# Irinotecan alleviates chemoresistance to anthracyclines through the inhibition of AARS1-mediated BLM lactylation and homologous recombination repair

**DOI:** 10.1038/s41392-025-02302-y

**Published:** 2025-07-10

**Authors:** Xinyuan Li, Chunlin Zhang, Yuhua Mei, Wenlong Zhong, Wei Fan, Li Liu, Zhenwei Feng, Xuesong Bai, Chuan Liu, Mingzhao Xiao, Weiyang He, Tianxin Lin, Xin Gou

**Affiliations:** 1 Department of Urology, The First Affiliated Hospital of Chongqing Medical University, Chongqing, PR China; 2 Chongqing Key Laboratory of Molecular Oncology and Epigenetics, Chongqing, PR China; 3 Department of Urology, Sun Yat-sen Memorial Hospital, Sun Yat-sen University, Guangzhou, Guangdong, PR China; 4 Guangdong Provincial Key Laboratory of Malignant Tumor Epigenetics and Gene Regulation, Sun Yat-sen Memorial Hospital, Guangzhou, Guangdong, PR China; 5 Department of Breast and Thyroid Surgery, The First Affiliated Hospital of Chongqing Medical University, Chongqing, PR China; 6 Department of Urology, The Second Affiliated Hospital of Chongqing Medical University, Chongqing, PR China

**Keywords:** Cancer therapy, Molecular medicine

## Abstract

Chemoresistance remains the major barrier to cancer treatment. Metabolic and epigenetic reprogramming are involved in this process; however, the precise roles and mechanisms are largely unknown. Here, we report that lactate-induced lactylation promotes chemoresistance to anthracyclines by regulating homologous recombination (HR) repair. Using the global lactylome, we revealed the landscape of differentially lactylated sites and proteins in cancer cells isolated from resistant and nonresistant tumors. Specifically, BLM, a crucial helicase in the HR repair process, is highly lactylated at Lys24 by AARS1 in response to chemotherapy. Mechanistically, hyperlactylation of BLM improves its stability by inhibiting MIB1-mediated ubiquitination and increasing its interaction with DNA repair factors, promoting DNA end resection and HR repair. Delactylation of BLM via the Lys24 mutation impairs HR repair and increases anthracycline chemosensitivity. Irinotecan shows synergistic effects and safety for alleviating anthracycline resistance by targeting BLM lactylation and suppressing HR repair in pancancer PDX models. A single-arm, phase I study (identifier NCT06766266) initiated by us suggested that the combination of irinotecan liposomes plus EPI is a feasible and safe treatment strategy for patients with anthracycline-resistant bladder cancer who experience recurrence. These findings exemplify how glycolytic reprogramming regulates HR repair through promoting protein lactylation and highlight the promising therapeutic potential of irinotecan for reversing anthracycline chemoresistance by suppressing BLM lactylation.

## Introduction

Despite considerable advances in cancer therapy, the development of chemoresistance remains one of the greatest obstacles to cancer treatment and causes significant mortality. Anthracyclines, such as epirubicin (EPI), pirarubicin, and doxorubicin, are aromatic type II polyketides that possess significant anticancer activities.^1^ Over the past few decades, anthracyclines have been used as effective treatments for a number of cancers, including bladder cancer,^2^ breast cancer,^3^ gastrointestinal cancer,^4^ leukemia,^5^ and lymphoma.^6^ Typically, the initial response to anthracyclines is excellent. However, many patients are at high risk of developing recurring or progressive disease within several years because of the development of resistance, resulting in increased mortality.^7,8^ Therefore, elucidating the molecular mechanisms underlying anthracycline chemoresistance is critical for the development of novel targeted therapies.

Complex and plastic metabolic patterns are important characteristics of tumor cells.^9^ Metabolic reprogramming satisfies the remarkable energy and biosynthesis requirements of tumor cells, facilitating their proliferation, invasion, and other malignant behaviors.^10,11^ Moreover, the metabolic properties and preferences of tumors change during cancer progression.^12,13^ Targeting cancer metabolism is an emerging research domain, and several inhibitors have advanced to anticancer clinical trials.^14^ However, the role of metabolic reprogramming in tumor resistance is not well understood. It remains unclear whether prolonged stimulation by chemotherapeutics alters metabolic patterns in tumor cells, ultimately reshaping their adaptability to drugs and, if so, the underlying mechanisms.

The precise regulation of protein function influences cellular activity and determines cell fate. Among multiple regulatory mechanisms, posttranslational modifications (PTMs) have garnered significant interest because they participate in almost all aspects of cellular, biological, and pathological processes by influencing protein stability, intervening in protein‒protein binding, regulating subcellular localization, and influencing transcriptional activity.^15,16^ Moreover, PTMs serve as a bridge connecting metabolic reprogramming and protein function regulation because multiple metabolites serve as substrates for PTMs, such as acetoacetyl-CoA (coenzyme A), crotonyl-CoA, and succinate.^16^ Lactate has been identified as the substrate of lactylation (Kla), which acts as a central node that links glycolytic metabolism and protein functions.^17,18^ Histone hyperlactylation directly influences gene expression and regulates the immune response,^19,20^ microglial inflammation,^21^, and tumorigenesis.^22^ Lactylation also regulates the functions of nonhistone proteins. For example, hyperlactylation of adenylate kinase 2 promotes the proliferation and metastasis of hepatocellular carcinoma cells.^23^ Lactylation of methyltransferase-like 3 enhances its RNA capture ability and facilitates m6A-mediated tumor immunosuppression.^12^ However, the mechanisms underlying the modulation of chemoresistance by PTMs remain unclear.

DNA damage, especially double-strand breaks (DSBs), occurs daily in cells in response to intrinsic or extrinsic damaging events.^24^ The DNA damage response (DDR) is a series of precise processes for repairing DNA lesions, including cell cycle checkpoint activation, DNA repair, apoptosis, and senescence.^24^ The normal function of the DDR is essential for maintaining genome stability and accurate gene transmission; however, when it is dysregulated, it is associated with aging and carcinogenesis.^24,25^ Genetic or epigenetic defects in DDR genes have been reported to promote the development of malignancies, particularly familial breast and ovarian cancers.^26–28^ There are four pathways responsible for repairing DSBs: nonhomologous end joining (NHEJ), homologous recombination (HR), alternative end-joining, microhomology-mediated end joining (MMEJ) and single-strand annealing (SSA).^25^ Multiple chemotherapeutic agents, including anthracyclines, exert their anticancer effects by inducing DNA intercalation and damage.^1,29^ An abnormally activated DDR confers increased resistance to DNA-damaging cancer therapies.^30^ Previous studies have demonstrated the crucial roles of HR in anthracycline-induced DSBs; however, the underlying molecular mechanisms remain largely elusive.^31,32^ BLM is a highly conserved human RecQ helicase that functions as an upstream sensor protein in the DNA damage signaling cascade and plays an important role in HR repair processes.^33^ BLM promotes HR by stimulating 5′ end resection and dissolution of the Holliday junction and inhibits unfavorable HR by melting the D-loop.^34,35^ Loss of BLM function leads to hypersensitivity of cancer cells to chemotherapy drugs, and BLM mutations are associated with susceptibility to malignant tumors.^36^

In the present study, we demonstrated that lactate-derived lactylation promoted EPI resistance by regulating HR. We found that lactate production and lactylation levels were increased in resistant cancer cells. Inhibiting lactylation increased the sensitivity of tumor cells to chemotherapies. Using the global lactylome, we first characterized the entire landscape of differentially lactylated proteins in resistant or nonresistant cancer cells. Mechanistically, we found that hyperlactylation of BLM at lysine 24 (K24) modulates HR processes and promotes chemoresistance. Mutation at the lactylated site alleviated chemoresistance. We further screened small molecule drugs and reported that irinotecan, a topoisomerase inhibitor mostly used for first-line treatment of colorectal and pancreatic cancer, effectively inhibits BLM lactylation and promotes cancer cell sensitivity to EPI treatment in pancancer models, such as bladder cancer, breast cancer, and hepatocellular carcinoma. Overall, this study reveals that BLM lactylation-dependent mechanisms underlie chemoresistance by regulating HR and suggests that inhibiting BLM lactylation represents a useful therapeutic strategy for treating chemoresistance.

## Results

### Lactate and lactylation levels are elevated in EPI-resistant tumor samples and cancer cells

**Fig. 1:**
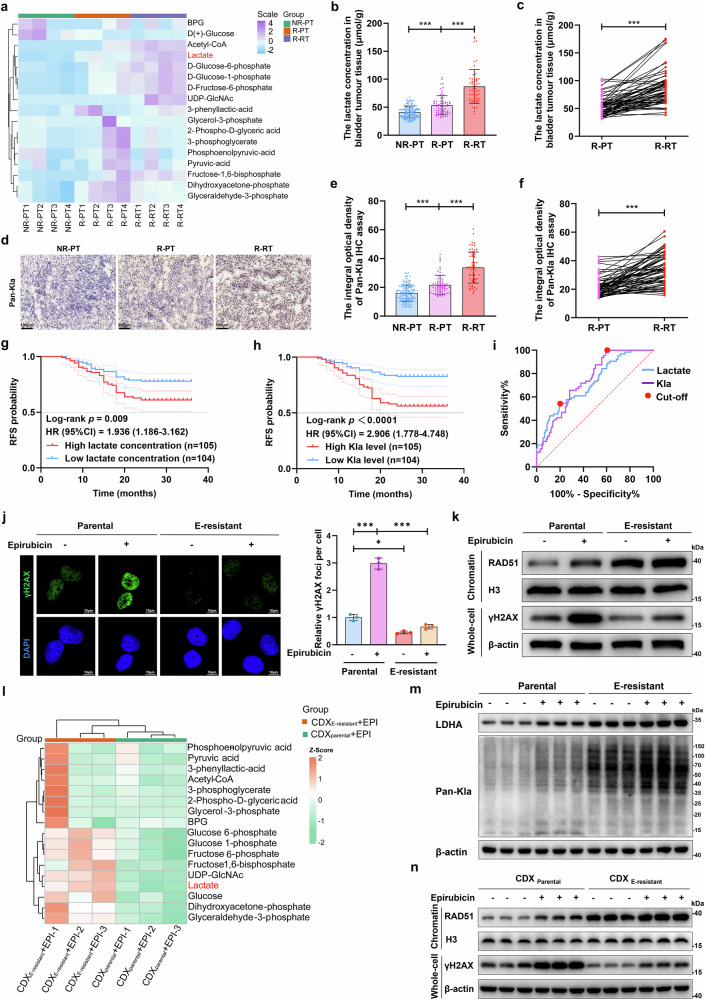
Lactate and lactylation levels were increased, and DNA repair was hyperactivated in EPI-resistant tumor tissues and cells. **a** Metabolic analysis of energy metabolites in primary tumors from patients with nonresistant NMIBC (NR-PT, *n* = 4), primary tumors from patients with resistant NMIBC (R-PT, *n* = 4), and recurrent tumors from patients with resistant NMIBC (R-RT, n = 4), highlighting the glycolytic metabolite lactate; NMIBC represents nonmuscle-invasive bladder cancer. **b** Lactate concentrations in NR-PT, R-PT, and R-RT bladder tumor samples from patients with nonresistant NMIBC (*n* = 145) and patients with resistant NMIBC (*n* = 64) compared via one-way ANOVA followed by Tukey’s test**. c** Lactate levels were compared via paired two-tailed Student’s t tests. **d** Representative pan-Kla immunohistochemical staining of NR-PT, R-PT, and R-RT bladder tumor tissues; scale bar: 100 μm. **e**, **f** The integral optical densities of pan-Kla staining in NR-PT (*n* = 145), R-PT (*n* = 64), and R-RT (*n* = 64) bladder tumor tissues were compared via one-way ANOVA followed by Tukey’s test and paired two-tailed Student’s t test. Kaplan‒Meier survival curves of the recurrence-free survival (RFS) of patients with NMIBC based on lactate concentrations (**g**) and pan-Kla levels (**h**) in primary tumor tissues; the median lactate concentration and the median integral optical density of pan-Kla were employed as the cutoff values; the statistical significance of RFS was determined via the Kaplan‒Meier method, and the *P* value was calculated via the log-rank test. **i** Receiver operating characteristic (ROC) analysis for recurrence prediction based on lactate concentrations and pan-Kla levels in primary bladder tumor tissues; red circles indicate cutoff values of 34.69 and 40. **j** The parental and EPI-resistant (E-resistant) UM-UC-3 cell lines either treated with 0.2 μM epirubicin (EPI) for 24 h or untreated; representative confocal microscopy images (left) and quantitative analyses (right) show the formation and disappearance of γH2AX (green) foci and the merging with DAPI staining (blue) of nuclei; scale bar: 10 μm. **k** Western blot analysis examining RAD51 accumulation in chromatin fractions and γH2AX expression in whole-cell extracts of parental and E-resistant UM-UC-3 cells before or 24 h after treatment with 0.2 μM EPI. **l** Heatmap showing energy metabolism metabolites in tumor cells from EPI-treated CDX_E-resistant_ and CDX_parental_ models (*n* = 3). **m** Western blot analysis of LDHA and pan-Kla in whole-cell extracts from CDX_E-resistant_ and CDX_parental_ models. **n** Western blot showing RAD51 chromatin accumulation and γH2AX expression in whole-cell extracts from CDX_E-resistant_ and CDX_parental_ models. ***p < 0.001 represents a significant difference between two groups

To investigate the metabolic characteristics of anthracycline-resistant tumors, we performed a metabolomic analysis of tumor samples consisting of nonresistant primary tumors (NR-PTs), resistant primary tumors (R-PTs), and resistant recurrent tumors (R-RTs) collected from patients with bladder cancer treated with EPI. We observed that the production of lactate was greater in R-PT samples than in NR-PT samples, with a further increase noted in R-RT tumors (Fig. 1a, Supplementary Fig. 1a). The metabolome results were validated by detecting the lactate content in larger samples in a prospective and single-blind clinical study (Table S1, Fig. 1b, c). As lactate serves as a substrate for lactylation, we measured panlactylation (Pan-Kla) levels in the tumors. We found that lactylation levels were increased in R-PT samples and further increased in R-RT tumors (Fig. 1d–f, Supplementary Fig. 1b, c). Higher lactate concentrations (HR: 1.936, 95% CI: 1.186–3.162, *p* = 0.009) and lactylation levels (HR: 2.906, 95% CI: 1.778–4.748, *p* < 0.001) were associated with inferior recurrence-free survival (RFS) in patients with bladder cancer treated with EPI (Fig. 1g, h). Receiver operating characteristic (ROC) curve analysis revealed that the mean area under the curve (AUC) for the prediction approaches based on lactate and lactylation levels in primary tumors was 0.720 (cutoff: 34.69, 95% CI: 0.646–0.793, *p* < 0.001) and 0.741 (cutoff: 40, 95% CI: 0.675–0.808, *p* < 0.001), respectively (Fig. 1i).

We then developed EPI-resistant bladder cancer cells (E-resistant, UM-UC-3) in vitro. The IC50 in the E-resistant cancer cells was approximately 10-fold greater than that in the parental cells (Supplementary Fig. 1d). Compared with parental cells, E-resistant cancer cells presented greater viability and more potent proliferation abilities in response to EPI treatment (Supplementary Fig. 1e, f). We observed that lactylation levels were increased in E-resistant cancer cells (Supplementary Fig. 1g). DNA damage and repair play pivotal roles in the antitumor effect of EPI. The results revealed that the accumulation of RAD51 on chromatin, a key machinery of DNA repair,^37^ was increased in E-resistant cancer cells, whereas γH2AX expression was reduced compared with that in parental cells (Fig. 1j, k). Consistently, comet assays revealed greater DNA damage in the EPI-treated parental cells than in the E-resistant cells (Supplementary Fig. 1h). These findings suggest that hyperactivation of the DNA repair process decreases DNA damage in EPI-resistant cancer cells; however, the lack of DNA repair allows the persistence of EPI-induced DNA damage in parental cancer cells.

Subsequently, we constructed cell line-derived xenograft nude mouse models using E-resistant and parental bladder cancer cells (CDX_E-resistant_ and CDX_Parental_) and confirmed that CDX_E-resistant_ cancer cells exhibited lower sensitivity to EPI treatment and greater proliferation ability (Supplementary Fig. 2a–d). Consistently, metabolome analysis indicated that lactate levels were increased in cancer cells isolated from EPI-treated CDX_E-resistant_ (CDX_E-resistant_ + EPI) tissues (Fig. 1l, Supplementary Fig. 2e). This change was accompanied by elevated lactate dehydrogenase A (LDHA) expression and lactylation levels (Fig. 1m). Consistent with the in vitro findings, we observed increased RAD51 chromatin accumulation and reduced γH2AX levels in CDX_E-resistant_ tissue-isolated tumor cells, indicating that hyperactivated DNA repair mitigated DNA damage (Fig. 1n). Collectively, these results indicate that metabolic reprogramming toward glycolysis and increased lactylation may participate in EPI resistance, which is likely related to hyperactivation of DNA repair in cancer cells.

### Inhibition of lactylation suppresses resistance to EPI in cancer cells

**Fig. 2:**
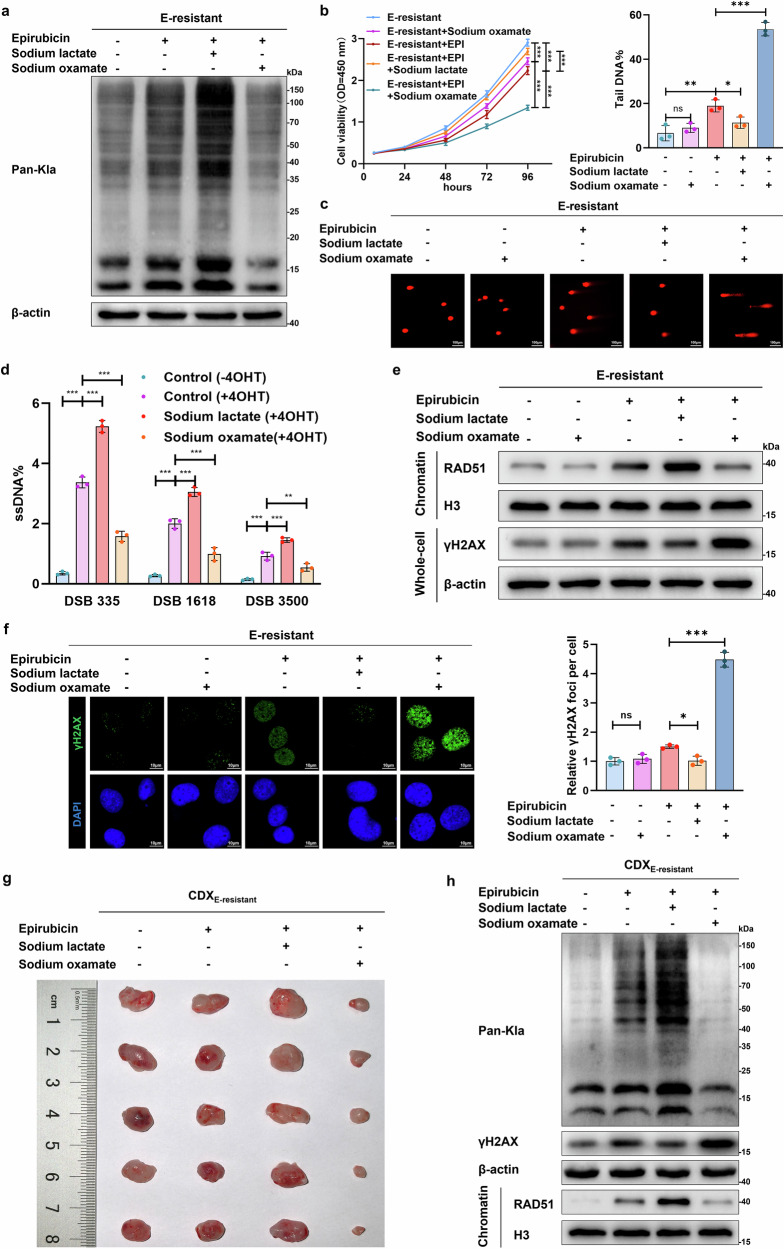
Lactate-derived lactylation induces HR and promotes EPI resistance. **a** Pan-Kla and β-actin levels were determined by western blot in E-resistant UM-UC-3 cells before or 24 h after treatment with 0.2 μM EPI, 20 mM sodium lactate, and/or 20 mM sodium oxamate as indicated. **b** Viability of E-resistant UM-UC-3 cells before or 24 h after treatment with 0.2 μM EPI, 20 mM sodium lactate, and/or 20 mM sodium oxamate; one-way ANOVA followed by Tukey’s test. **c** Representative images of comet assays performed with E-resistant UM-UC-3 cells treated with the same experimental setup as described in b; the percentage of DNA tails was compared by one-way ANOVA followed by Tukey’s test; scale bar: 100 μm. **d** AsiSI-ER U2OS system and qPCR-based quantification of single-stranded DNA (ssDNA) generated by DNA end resection; ssDNA levels were normalized to those of the control (-4OHT) group; one-way ANOVA followed by Tukey’s test. **e** The same experimental setup as in b, followed by western blot analysis of RAD51 accumulation in chromatin fractions and γH2AX expression in whole-cell extracts. **f** Same experimental setup as in b. Representative images (left) and quantitative analyses (right) showing the formation of EPI-induced γH2AX (green) foci merged with DAPI (blue); scale bar: 10 μm. **g** Nude mice were transplanted subcutaneously with E-resistant UM-UC-3 cells and treated with EPI (2 mg/kg), sodium lactate (100 mg/kg), or sodium oxamate (500 mg/kg); representative tumor images are shown (*n* = 5). **h** Western blot analysis of pan-Kla and γH2AX (whole-cell extracts) and RAD51 (chromatin fractions) from CDX models. Except for panels g and h, the graphs represent three replicates per condition (*n* = 3). ***p < 0.001, **p < 0.01, *p < 0.05 represent significant differences between two groups; ns represents no significant difference

To explore whether the upregulation of lactylation promotes the chemoresistance of cancer cells, the glycolysis inhibitor sodium oxamate was used to inhibit lactate production. As the lactate concentration decreased, the lactylation level decreased (Fig. 2a). Importantly, the inhibition of lactylation increased the sensitivity of E-resistant cancer cells to EPI treatment; however, sodium lactate increased lactylation levels and further promoted their resistance (Fig. 2b). Colony formation experiments revealed that the addition of sodium lactate restored cell proliferation in E-resistant cells in response to EPI treatment; however, the inhibition of lactylation via sodium oxamate increased cell sensitivity to EPI treatment and reversed EPI resistance (Supplementary Fig. 3a). The downregulation of lactylation efficiently promoted a lack of DNA repair, leading to the persistence of DNA damage in sodium oxamate-treated cancer cells, as shown by the prolonged comet tail length; however, lactate treatment increased the repair capacity and caused a decrease in DNA damage (Fig. 2c). qPCR assays were conducted to measure the DNA end-resection efficiency adjacent to DNA double-strand break sites, which is an important hallmark of HR repair. We found that lactylation promoted DNA end resection, which decreased in response to oxamate treatment (Fig. 2d). Additionally, RAD51 chromatin accumulation was decreased in sodium oxamate-treated cancer cells, accompanied by the upregulation of γH2AX expression; however, lactate increased the association of RAD51 with chromatin and inhibited γH2AX expression (Fig. 2e, f).

Consistently, in vivo validation demonstrated that inhibition of glycolysis reduced lactylation and suppressed HR repair processes, further mediating the accumulation of DNA damage and the reversal of EPI resistance in CDX_E-resistant_ models (Fig. 2g, h, and Supplementary Fig. 3b). In contrast, sodium lactate-induced lactylation strengthened HR repair and decreased sensitivity to EPI treatment, as evidenced by increased RAD51 accumulation on chromatin and downregulation of γH2AX expression. These results indicate that glycolysis reprogramming-induced lactylation facilitates HR repair and EPI resistance.

To determine whether EPI resistance was attributed to hyperactivation of HR repair, we constructed two additional EPI-resistant cancer cell lines, breast cancer (MDA-MB-231, Supplementary Fig. 3c) and hepatocellular carcinoma (Huh7, Supplementary Fig. 3d), and applied an HR inhibitor (B02) and an NHEJ inhibitor (Ku-57788) to EPI-resistant cancer cell lines. Notably, suppression of HR caused prominent upregulation of EPI-induced DSBs (Supplementary Fig. 3e–g) and reversed EPI resistance (Supplementary Fig. 3h–j), but Ku-57788 rarely affected resistance to EPI. Collectively, these results reveal that lactate-driven lactylation facilitates EPI resistance, likely via the regulation of HR repair processes; however, the underlying molecular mechanisms are unclear.

### Characterization of the global landscape of lactylated proteins in resistant and nonresistant cancer cells

**Fig. 3:**
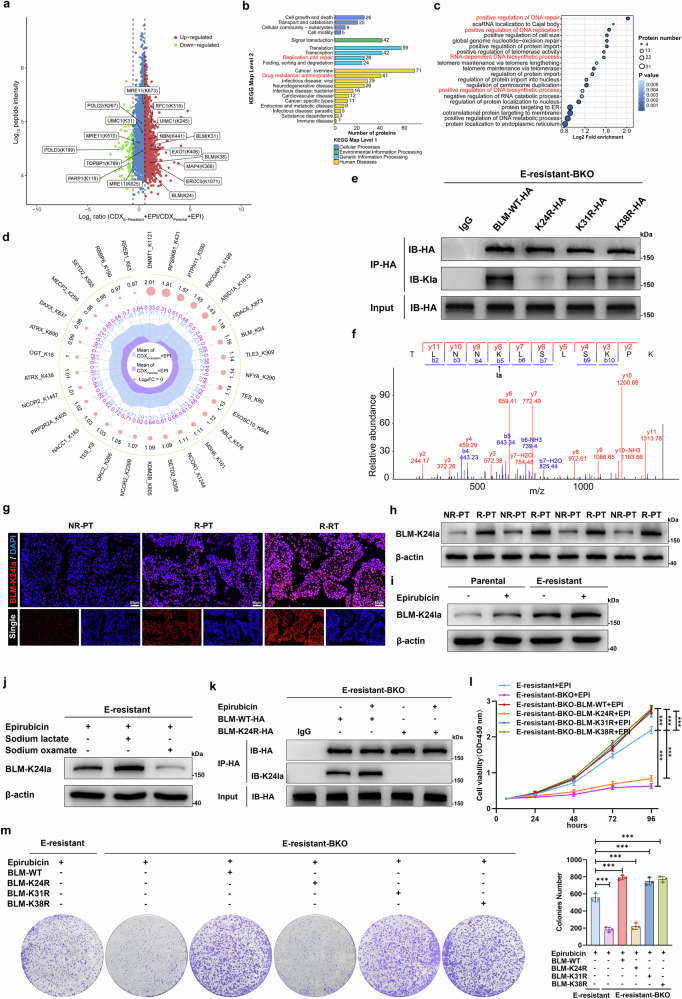
Hyperlactylation of BLM at Lys24 induces EPI resistance. **a** Scatterplot showing the quantification of lactylated sites in relation to peptide intensities in tumor cells separated from EPI-treated CDX_E-resistant_ and CDX_parental_ models, highlighting lactylated proteins and peptides in the DNA repair pathway. KEGG pathway (**b**) and GO biological process (**c**) analyses of upregulated lactylated proteins in EPI-treated CDX_E-resistant_ models. **d** Radar diagram showing the top 30 lactylated proteins enriched in the tumor resistance pathway in tumor cells separated from EPI-treated CDX_E-resistant_ models; the larger pink circles represent higher log_2_FC values; blue and purple numbers represent the quantification of protein lactylation in the EPI-treated CDX_E-resistant_ and CDX_parental_ models, respectively. **e** BLM-knockout E-resistant cells (E-resistant-BKO) were transfected with HA-tagged wild-type (WT), K24R, K31R, or K38R BLM; after transfection, HA-tagged BLM proteins were immunoprecipitated with an anti-HA antibody or an IgG control and analyzed via western blotting with anti-HA and anti-pan-Kla antibodies. **f** Illustration of BLM-K24 lactylation identified by MS. **g** Representative immunofluorescence images showing K24-lactylation-specific antibodies (BLM-K24la, red) merged with DAPI (blue) of nuclei in NR-PT, R-PT, and R-RT bladder tumor tissues; scale bar: 50 μm. BLM-K24 lactylation levels were determined by western blot with BLM-K24la antibody in NR-PT and R-PT bladder tumor samples (**h**) and in parental and E-resistant UM-UC-3 cells (**i**). **j** Western blot showing BLM-K24 lactylation levels in E-resistant cells treated with 0.2 μM EPI, 20 mM sodium lactate, and/or 20 mM sodium oxamate, as indicated. **k** HA-tagged BLM proteins were immunoprecipitated with anti-HA from E-resistant-BKO cells transfected with HA-tagged WT and K24R BLM plasmids; western blot analysis was performed to determine BLM-K24 lactylation levels. **l** Cell viability assays detecting the viability of E-resistant and E-resistant-BKO UM-UC-3 cells transfected with WT, K24R, K31R, or K38R BLM plasmids after treatment with 0.2 μM EPI; one-way ANOVA followed by Tukey’s test. **m** The same experimental setup as in k but displaying cell proliferation by colony formation assays; one-way ANOVA followed by Tukey’s test. The graphs represent three replicates per condition (*n* = 3). ***p < 0.001 represents a significant difference between two groups

To investigate the key lactylated proteins that modulate chemoresistance, we used the global lactylome and proteomics to explore differentially lactylated proteins in bladder cancer cells isolated from EPI-treated CDX_Parental_ and CDX_E-resistant_ tissues. Overall, 743 sites on 516 proteins exhibited differential lactylation, with 392 sites on 279 proteins showing upregulated lactylation levels (Fig. 3a). Gene Ontology (GO) and Kyoto Encyclopedia of Genes and Genomes (KEGG) analyses (Fig. 3b, c) revealed that hyperlactylated proteins were associated with tumor resistance and enriched in multiple DNA repair-related pathways. BLM, a helicase involved in the HR repair process, was lactylated at three potential sites (K24, K31, and K38). Furthermore, BLM was one of the lactylated proteins enriched in the tumor resistance pathway (Fig. 3d). Notably, we revealed that lactate-driven lactylation facilitated EPI resistance, likely through hyperactivated HR repair. We therefore investigated whether BLM lactylation regulates HR repair and accordingly induces EPI resistance.

We subsequently knocked out endogenous BLM in EPI-resistant cancer cells (E-resistant-BKO) and then rescued them with WT, K24R, K31R, or K38R BLM (Supplementary Fig. 4a). The results showed that the K24R mutation caused a reduction in BLM lactylation. In contrast, the K31R or K38R mutation did not affect BLM lactylation, indicating that K24 is a key lactylation site in BLM (Fig. 3e, f, and Supplementary Fig. 4b, c). To facilitate the specific recognition of BLM-K24 lactylation, a K24-lactylation-specific antibody (BLM-K24la) was generated. We found that BLM lactylation at the K24 site was increased in E-resistant bladder tumor tissues (Fig. 3g, h) and different E-resistant cell lines (Fig. 3i and Supplementary Fig. 4d, e). Treatment with sodium lactate further increased BLM-K24 lactylation (Fig. 3j). However, the BLM-K24R mutation abolished the signal recognized by the BLM-K24la antibody (Fig. 3k). Consistently, IP assays demonstrated the upregulation of BLM-K24 lactylation in E-resistant cancer cells, which was inhibited by sodium oxamate (Supplementary Fig. 4f–i).

To investigate whether BLM lactylation promotes EPI resistance, we overexpressed WT, K24R, K31R, and K38R BLM in E-resistant BKO cells and found that BLM knockout increased sensitivity to EPI treatment in different E-resistant cell lines, indicating the important role of BLM in EPI resistance. Notably, BLM-WT overexpression rescued viability and proliferation in E-resistant BKO cells in response to EPI treatment and reinduced chemoresistance; however, the K24R mutation impaired the effect of BLM (Fig. 3l, m, and Supplementary Fig. 4j, k). Consistent with the changes in lactylation, the K31R or K38R mutation scarcely affected the role of BLM in EPI resistance.

To validate the roles of BLM lactylation in other anthracycline-based drugs, we developed pirarubicin (THP)-resistant bladder cancer cells (T-resistant, Supplementary Fig. 4l). BLM-WT overexpression evidently rescued the viability and proliferation of T-resistant BKO cancer cells in response to THP treatment; however, K24R mutation impaired the effect of BLM (Supplementary Fig. 4m, n). These results show that BLM lactylation at K24 plays a crucial role in anthracycline resistance.

### Hyperlactylation of BLM regulates protein functions and promotes the HR repair process

**Fig. 4:**
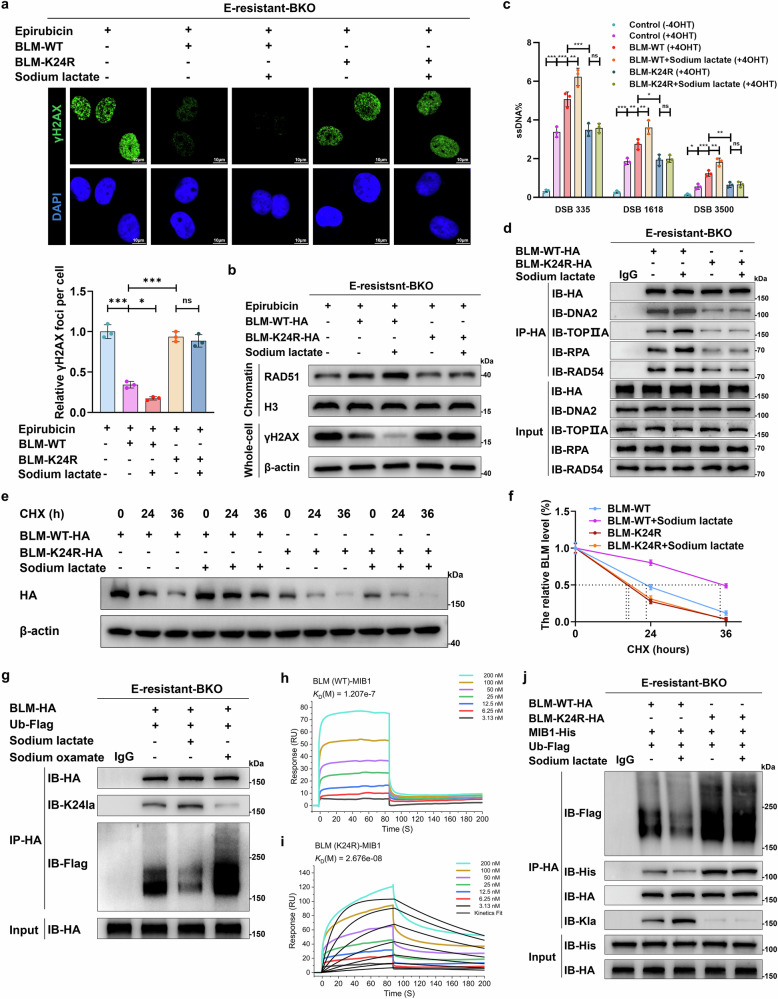
BLM-K24 lactylation facilitates HR and increases BLM protein stability. **a** E-resistant BKO UM-UC-3 cells were transfected with WT or K24R BLM plasmids; representative images (top) and quantitative analyses (bottom) show the formation of EPI-induced γH2AX (green) foci merged with DAPI (blue) before and 24 h after treatment with 0.2 μM EPI and 20 mM sodium lactate; scale bar: 10 μm. **b** Western blot analysis was performed to examine RAD51-chromatin associations and γH2AX expression in E-resistant BKO UM-UC-3 cells treated as described in a. **c** AsiSI-ER U2OS system and qPCR-based quantification analysis of ssDNA generated by DNA end resection in cells transfected with WT or K24R BLM plasmids before and 24 h after treatment with 20 mM sodium lactate; the ssDNA level was normalized to that of the control (-4OHT) group; one-way ANOVA followed by Tukey’s test. **d** E-resistant BKO UM-UC-3 cells transfected with HA-tagged WT or K24R BLM plasmids were treated with 20 mM sodium lactate or 20 mM sodium oxamate for 24 h; HA-tagged BLM proteins were immunoprecipitated with an anti-HA antibody or an IgG control and analyzed via western blotting with anti-HA and target antibodies as indicated. **e**, **f** Half-life detection and quantitative analysis of the WT and K24R BLM proteins in E-resistant-BKO UM-UC-3 cells treated with 40 μM cycloheximide and/or 20 mM sodium lactate for the indicated times. **g** E-resistant BKO cells stably expressing HA-BLM were cotransfected with Flag-ubiquitin (Ub) and treated with 20 mM sodium lactate or 20 mM sodium oxamate for 24 h, followed by immunoprecipitation with an anti-HA antibody or an IgG control; the blots were probed with the indicated antibodies. **h**, **i** The interaction between BLM and MIB1 proteins was tested via surface plasmon resonance (SPR) assay; the BIAcore diagram shows a greater affinity between K24R BLM and MIB1 than between WT BLM and MIB1, with fast dissociation kinetics for WT BLM and slow dissociation kinetics for K24R BLM. **j** E-resistant BKO UM-UC-3 cells cotransfected with HA-WT, HA-K24R BLM, His-MIB1, and/or Flag-Ub plasmids were treated with 20 mM sodium lactate or left untreated; HA-BLM proteins were immunoprecipitated with an anti-HA antibody or an IgG control and analyzed via western blotting with anti-HA and target antibodies as indicated. The graphs represent three replicates per condition (*n* = 3). ***p < 0.001, **p < 0.01, *p < 0.05 represent significant differences between two groups

We subsequently explored whether BLM-K24 lactylation induced anthracycline resistance by modulating HR repair processes. Compared with the BLM WT-overexpressing group, inhibition of lactylation via K24 site mutation impaired HR repair, resulting in increased DNA damage in E-resistant BKO cells. Notably, lactate strengthened the function of BLM-WT but scarcely affected the K24R mutant (Fig. 4a, b and Supplementary Fig. 5a, b). These results were corroborated by DNA end resection assays (Fig. 4c and Supplementary Fig. 5c) and comet assays (Supplementary Fig. 5d).

Mechanistically, we investigated the effects of BLM lactylation on protein function. However, electrophoretic mobility shift assays (EMSAs) revealed no significant difference in binding to DNA, including dsDNA and 3′-tailed DNA, between the WT and K24R BLM proteins (Supplementary Fig. 5e). Interestingly, the K24R mutation impaired BLM interactions with key DNA repair factors, including DNA2, TOPIIA, RPA, and RAD54. Lactate strengthened the interaction between WT BLM and these DNA repair proteins but had little effect on the K24R mutant (Fig. 4d and Supplementary Fig. 5f). Helicase assays also revealed that the K24R mutation suppressed DNA unwinding ability in the BLM-RPA-dependent in vitro DNA unwinding system (Supplementary Fig. 5g), probably because the K24R mutation impaired the interaction between BLM and RPA. In addition, molecular dynamics simulations indicated that the K24R mutation led to a dramatic change in the BLM protein structure, with a root mean square deviation (RMSD) of 1.40 nm (Supplementary Fig. 5h). These results suggest that BLM lactylation at K24 facilitates HR repair processes by modulating BLM function.

### BLM lactylation enhances protein stability by suppressing MIB1-mediated ubiquitination

Next, we explored the mechanisms by which BLM lactylation augments HR repair. Cycloheximide (CHX) assays revealed that, compared with that of BLM-WT, the stability of the K24R mutant was lower and scarcely influenced by sodium lactate (Fig. 4e, f). However, sodium lactate increased the stability of the BLM-WT protein. Notably, consistent with the changes in lactylation, the K31R or K38R mutation scarcely affected BLM protein stability (Supplementary Fig. 6a, b). These findings indicate that BLM-K24 lactylation increased its stability.

We subsequently revealed that BLM ubiquitination was suppressed in E-resistant cancer cells (Supplementary Fig. 6c) and that inhibition of BLM lactylation via a glycolysis inhibitor increased BLM ubiquitination; however, sodium lactate treatment had the opposite effect (Fig. 4g). The E3 ligase MIB1 has been identified as an effective ubiquitinase for BLM that modulates its stability.^38^ We confirmed that the overexpression of MIB1 increased BLM ubiquitination by interacting with BLM in E-resistant cancer cells (Supplementary Fig. 6d). We subsequently purified human MIB1, BLM-WT, and BLM-K24R proteins and conducted surface plasmon resonance (SPR) assays. The results revealed that the BLM-K24R mutant exhibited stronger binding to MIB1 than the BLM-WT protein did, with K_D_ values of 1.21e-7 M and 2.676e-8 M, respectively (Fig. 4h, i). Consistently, molecular docking assays demonstrated lower PIPER energies of docking between the BLM-K24R and MIB1 proteins than between the BLM-WT and MIB1 proteins (Supplementary Fig. 6e, f). These findings suggest that BLM lactylation attenuates its ability to bind to MIB1. Consistent with the results of the SPR and molecular docking assays, we detected that inhibition of BLM lactylation via K24 mutation enhanced the interaction between BLM and MIB1, thus promoting MIB1-mediated BLM ubiquitination (Fig. 4j and Supplementary Fig. 6g). Enhancing BLM-WT lactylation via sodium lactate inhibited the BLM-MIB1 interaction and BLM ubiquitination but scarcely regulated the BLM-K24R mutant. In addition, mutation of K31R or K38R had no effect on BLM ubiquitination (Supplementary Fig. 6h), which is consistent with the changes in lactylation and protein stability. These results demonstrate that BLM-K24 lactylation decreases its ability to bind to MIB1 and suppresses BLM ubiquitination and degradation accordingly.

### High expression of AARS1 catalyzes BLM lactylation and induces anthracycline resistance

**Fig. 5:**
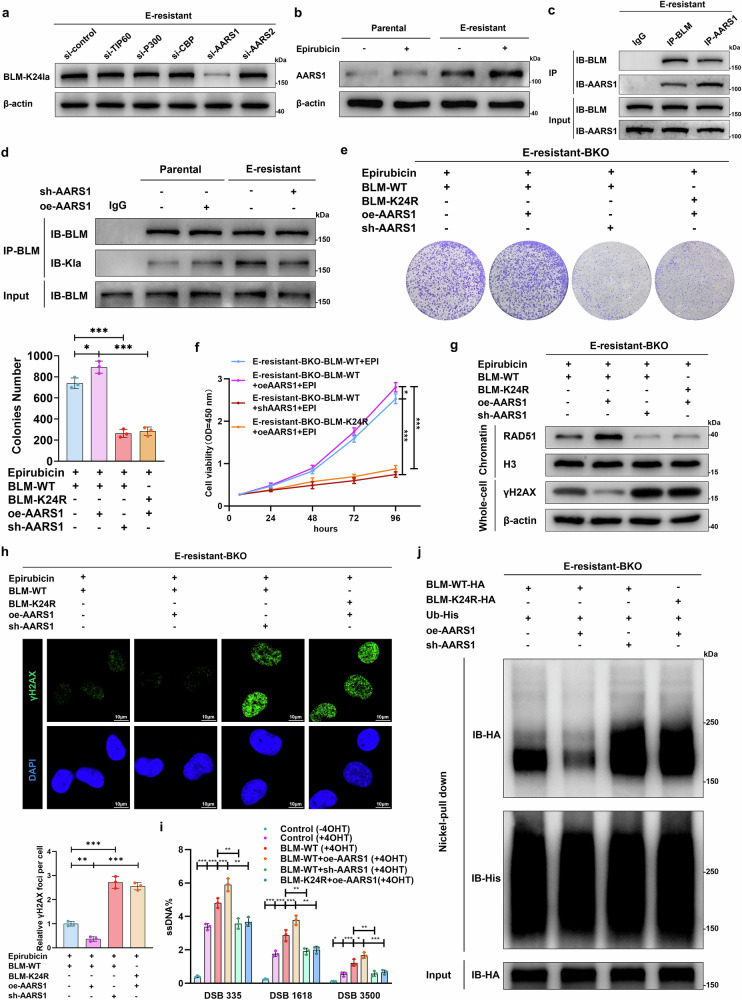
AARS1 catalyzes BLM lactylation and promotes HR and EPI resistance. **a** Western blot analyses were performed to examine BLM-K24 lactylation levels via a K24-lactylation-specific antibody in E-resistant UM-UC-3 cells treated with different siRNAs, as indicated. **b** AARS1 and β-actin levels were determined by western blot in parental and E-resistant UM-UC-3 cells before and 24 h after treatment with 0.2 μM EPI. **c** Endogenous BLM and AARS1 proteins were coimmunoprecipitated with anti-BLM, anti-AARS1, or IgG control and analyzed via western blotting with the indicated antibodies. **d** Parental and E-resistant cells with stable overexpression or knockdown of AARS1 were subjected to immunoprecipitation with an anti-BLM antibody or an IgG control; the blots were probed with the indicated antibodies. **e** E-resistant BKO UM-UC-3 cells with stable overexpression or knockdown of AARS1 were transfected with WT or K24R BLM plasmids and treated with 0.2 μM EPI; representative images show cell proliferation levels determined via colony formation assays and quantitative assays; one-way ANOVA followed by Tukey’s test. **f** Same experimental setup as in e but displaying the cell viability via cell viability assays. **g** Same experimental setup as that in **e** but for determining RAD51 accumulation in chromatin fractions and γH2AX expression in whole-cell extracts by western blot. **h** Representative images (top) and quantitative analyses (bottom) showing the formation of EPI-induced foci for γH2AX (green) merged with DAPI (blue) of nuclei; scale bar: 10 μm. **i** The AsiSI-ER U2OS system and qPCR-based quantification analysis were used for detecting ssDNA generated by DNA end resection in cells with stable overexpression or knockdown of AARS1; the ssDNA level was normalized on the basis of the results in the control (-4OHT) group; one-way ANOVA followed by Tukey’s test. **j** E-resistant BKO cells stably expressing AARS1 or AARS1 shRNA were cotransfected with His-Ub, HA-WT BLM, and/or HA-K24R BLM plasmids; the cells were subsequently lysed under denaturing conditions, and Ni-NTA beads were used to pull down His-tagged ubiquitin. The graphs represent three replicates per condition (*n* = 3). ***p < 0.001, **p < 0.01, *p < 0.05 represent significant differences between two groups

We screened five potential enzymes, TIP60, p300, CBP, AARS1, and AARS2, via short interfering RNAs (siRNAs). We found that the siRNA-mediated knockdown of AARS1, a novel lactate sensor and lactyltransferase,^39,40^ inhibited BLM lactylation in E-resistant cancer cells (Fig. 5a and Supplementary Fig. 7a). The expression of AARS1 was greater in the R-PT samples than in the NR-PT samples, with a further increase in the R-RT samples (Supplementary Fig. 7b–d). We found that higher AARS1 (HR: 1.679, 95% CI: 1.028–2.742, *p* = 0.038) was associated with inferior RFS in patients with bladder cancer treated with EPI (Supplementary Fig. 7e). ROC analysis revealed that the AUC for prediction approaches based on AARS1 expression in primary tumors was 0.636 (cutoff: 21.54, 95% CI: 0.556–0.715, *p* = 0.002) (Supplementary Fig. 7f). Consistently, an in vitro investigation revealed that AARS1 expression was increased in E-resistant bladder cancer cells and further upregulated in response to EPI treatment (Fig. 5b). However, the upregulation of AARS1 seems unrelated to the activation of glycolysis and increase in lactate levels because sodium lactate treatment could not further facilitate the upregulation of AARS1 expression (Supplementary Fig. 7g). Coimmunoprecipitation and molecular docking assays confirmed that BLM bound to AARS1, and the K24R mutation impaired the interaction between BLM and AARS1 (Fig. 5c and Supplementary Fig. 7h–j). Knockdown of AARS1 or inhibition of AARS1 lactyltransferase capacity via a crucial residue mutation decreased BLM lactylation, whereas overexpression of AARS1 promoted BLM lactylation (Fig. 5d and Supplementary Fig. 7k, l).

We subsequently investigated whether AARS1 modulates anthracycline resistance by catalyzing BLM lactylation. Inhibition of AARS1 expression restored EPI sensitivity in E-resistant cells overexpressing BLM-WT, whereas upregulation of AARS1 further promoted resistance to EPI treatment (Fig. 5e, f). Importantly, AARS1 scarcely affected sensitivity to EPI in BLM K24R-overexpressing cells. Moreover, we revealed that high expression of AARS1 promoted RAD51 chromatin accumulation and decreased γH2AX expression in E-resistant cancer cells overexpressing BLM-WT rather than the K24R mutant (Fig. 5g, h). However, downregulation of AARS1 via shRNA reduced the formation of RAD51 foci and increased γH2AX expression. Additionally, AARS1 facilitated BLM-mediated DNA end resection, whereas AARS1 knockdown inhibited this process (Fig. 5i). Mechanistically, AARS1 promoted the interaction of BLM with DNA repair factors, including DNA2, TOPIIA, and RPA, whereas AARS1 knockdown inhibited these interactions (Supplementary Fig. 7m). In addition, the upregulation of AARS1 inhibited BLM ubiquitination, whereas it was promoted in response to shRNA-AARS1 treatment (Fig. 5j and Supplementary Fig. 7n). Interestingly, compared with those in the WT group, the above effects of AARS1 were alleviated in the K24R mutant group. Taken together, these results indicate that high expression of AARS1 facilitates HR repair and induces anthracycline resistance by increasing BLM-K24 lactylation.

### Targeted inhibition of BLM-K24 lactylation via irinotecan increases the sensitivity of cancer cells to anthracyclines and reverses resistance

**Fig. 6:**
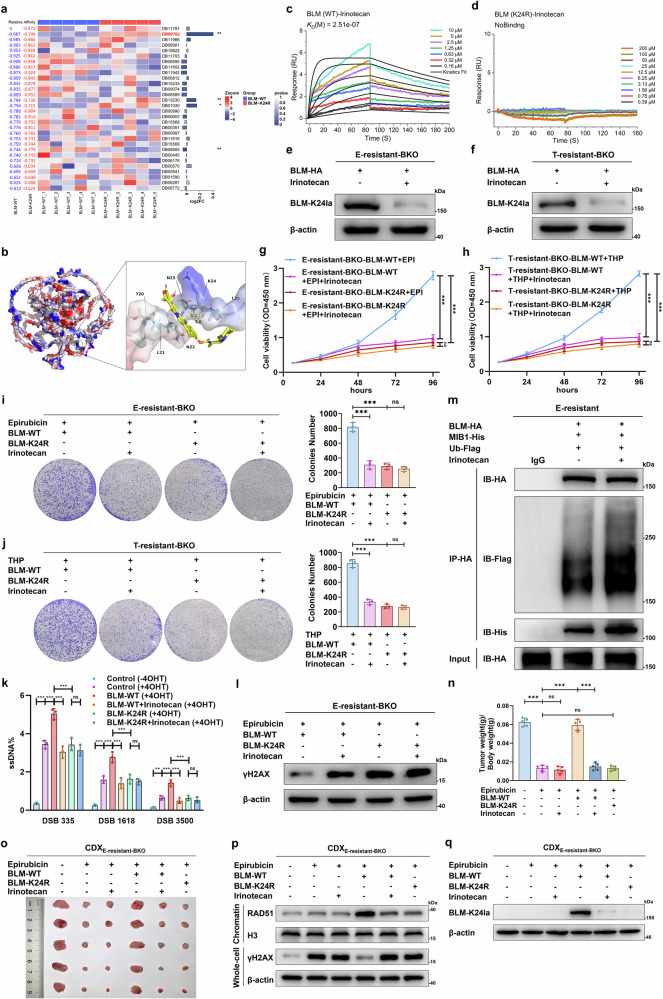
Irinotecan inhibits BLM-K24 lactylation and reverses anthracycline resistance. **a** Heatmap illustrating the relative affinities of the top 30 small-molecule drugs to the BLM-WT and BLM-K24R mutants determined via molecular docking; the left-side values represent the average affinities of five-time random docking between small-molecule drugs and the K24 regions of BLM-WT and the BLM-K24R mutant; the relative value was normalized to the average affinity of the small-molecule drug with the highest affinity to BLM-WT; the right-side log2FC and p values represent the changes in and significance of the average affinities of the small-molecule drugs toward WT BLM and the K24R BLM mutant. **b** Molecular docking diagram illustrating the docking pose of irinotecan small-molecule drug binding to the K24 region of the BLM protein; the dotted yellow lines represent two hydrogen bonds between irinotecan and the K24 residue with distances of 3.2 Å and 3.6 Å. **c** BIAcore diagram showing that the WT BLM protein bound to the irinotecan small-molecule drug with high affinity and slow dissociation kinetics. **d** The K24R BLM protein does not bind to the irinotecan small-molecule drug. Western blot analyses of BLM-K24 lactylation levels in E-resistant-BKO (**e**) and THP-resistant-BKO (**f**) UM-UC-3 cells transfected with the HA-BLM plasmid before and 24 h after treatment with 4 μM irinotecan. **g**, **h** Cell viability assays detecting the viability of E-resistant-BKO and THP-resistant-BKO UM-UC-3 cells transfected with WT or K24R BLM plasmids after treatment with 0.2 μM EPI, 0.3 μM THP, or 4 μM irinotecan; one-way ANOVA followed by Tukey’s test. **i**, **j** Representative images and quantitative analyses showing cell proliferation levels as assessed by colony formation assays in E-resistant-BKO and T-resistant-BKO UM-UC-3 cells treated with the same experimental setup described in g and h; one-way ANOVA followed by Tukey’s test. **k** The AsiSI-ER U2OS system and qPCR-based quantification analysis were used to detect ssDNA generated by DNA end resection in cells transfected with WT or K24R BLM plasmids after treatment with or without 4 μM irinotecan. **l** Same experimental setup as in (**g**) but determining γH2AX in whole-cell extracts by western blot. **m** E-resistant cells cotransfected with HA-BLM, His-MIB1, or Flag-Ub plasmids were treated with 4 μM irinotecan or left untreated; HA-BLM proteins were immunoprecipitated with an anti-HA antibody or an IgG control and analyzed via western blotting with antibodies as indicated. **n, o** Nude mice were transplanted subcutaneously with E-resistant BKO UM-UC-3 cells stably expressing WT BLM or K24R BLM and treated with EPI (2 mg/kg) and/or irinotecan (25 mg/kg) as indicated; tumor weights were measured as shown in M, and tumor images were acquired as shown in N (*n* = 5). **p**, **q** γH2AX and BLM-K24 lactylation in whole-cell extracts and RAD51 accumulation in chromatin fractions were examined by western blot in CDX models. Except for (**o**–**q**), the other graphs represent three replicates per condition (*n* = 3). ***p < 0.001, **p < 0.01, *p < 0.05 represent significant differences between two groups; ns represents no significant difference

These findings indicate that hyperlactylation of BLM at the K24 site plays an important role in inducing anthracycline resistance in cancer cells. Hence, we speculated that targeting BLM lactylation could rescue anthracycline resistance. Small-molecule drugs with high binding affinities and polar interactions with the BLM K24 region were screened from the DrugBank database via molecular docking and high-throughput screening. Among these, irinotecan (DB00762), a topoisomerase I (TOP I) inhibitor, showed the highest affinity for the BLM K24 region, as evidenced by a marked difference in the average affinity for BLM-WT and BLM-K24R (Fig. 6a, b). SPR assays further demonstrated a strong binding affinity between irinotecan and BLM-WT, with a K_D_ value of 2.51e-7 M (Fig. 6c). Notably, we did not observe a binding signal between irinotecan and the BLM-K24R mutant (Fig. 6d), whereas the binding affinities of irinotecan toward the K31R and K38R mutants remained unaffected by site mutagenesis, with K_D_ values of 7.76e-7 M and 9.11e-7 M, respectively (Supplementary Fig. 8a, b). Additionally, the 100 ns unrestricted molecular dynamics simulation assays revealed that irinotecan consistently bound to the K24 region of BLM throughout the simulation period (Supplementary Fig. 8c). The RMSD of the irinotecan–BLM protein complex remained stable at approximately 1 nm, indicating a stable interaction (Supplementary Fig. 8d). The molecular mechanics/generalized born surface area (MMGBSA) was subsequently used to calculate the binding free energy between irinotecan and the K24 region of BLM, which remained at approximately −25 kcal/mol, suggesting spontaneous and sustained binding of irinotecan to the K24 region of BLM (Supplementary Fig. 8e).

Next, we treated EPI-resistant and THP-resistant cancer cell lines, such as bladder cancer, breast cancer and hepatocellular carcinoma, with irinotecan. The treatment suppressed BLM-K24 lactylation (Fig. 6e, f and Supplementary Fig. 8f, g) and enhanced the sensitivity of these cells to EPI and THP (Fig. 6g–j and Supplementary Fig. 8h–k). The synergistic effects of irinotecan leading to the reversal of anthracycline resistance were impaired when the K24 site was mutated. To determine whether the synergistic effect is attributed mainly to the targeting of irinotecan for lactylation, we decreased TOP I expression in E-resistant cells by using siRNAs (Supplementary Fig. 8l) and confirmed that irinotecan treatment reduced BLM lactylation and suppressed cell viability (Supplementary Fig. 8m, n). Furthermore, combination treatment with irinotecan and EPI suppressed DNA end resection and HR repair in BLM-WT-overexpressing cancer cells (Fig. 6k), increasing the degree of DNA damage in this group (Fig. 6l). In addition, irinotecan promoted the interaction between BLM and MIB1 and increased their ubiquitination, resulting in impaired BLM stability (Fig. 6m and Supplementary Fig. 8o).

To validate whether targeting BLM K24 lactylation alleviated EPI resistance, we applied irinotecan in CDX_E-resistant-BKO_ models in vivo. We found that EPI inhibited tumor growth in the CDX_E-resistant BKO_ model and that overexpression of BLM-WT but not the K24R mutant rescued tumor growth in vivo. Synergistic treatment with irinotecan reversed the effect of BLM-WT and alleviated EPI resistance (Fig. 6n, o). Mechanistically, irinotecan treatment suppressed HR repair, restoring EPI-induced DNA damage in the BLM-WT-overexpressing group (Fig. 6p and Supplementary Fig. 9a). BLM-K24 lactylation was suppressed by irinotecan treatment (Fig. 6q). Furthermore, we constructed an EPI-resistant mouse bladder cancer cell line (MB49_E-resistant_) and cell line-derived allograft model using C57BL/6 J mice. Combination therapy with irinotecan reversed EPI resistance in the MB49_E-resistant_ cell line-derived allograft model; however, irinotecan scarcely affected the sensitivity to EPI in the BLM-knockout (MB49_E-resistant-BKO_) cell line-derived allograft model (Supplementary Fig. 9b–d). These collective results suggest that targeted inhibition of BLM-K24 lactylation via irinotecan reverses anthracycline resistance.

### Combination treatment with irinotecan and EPI shows synergistic effects and safety for anthracycline-resistant tumors in PDX models

**Fig. 7:**
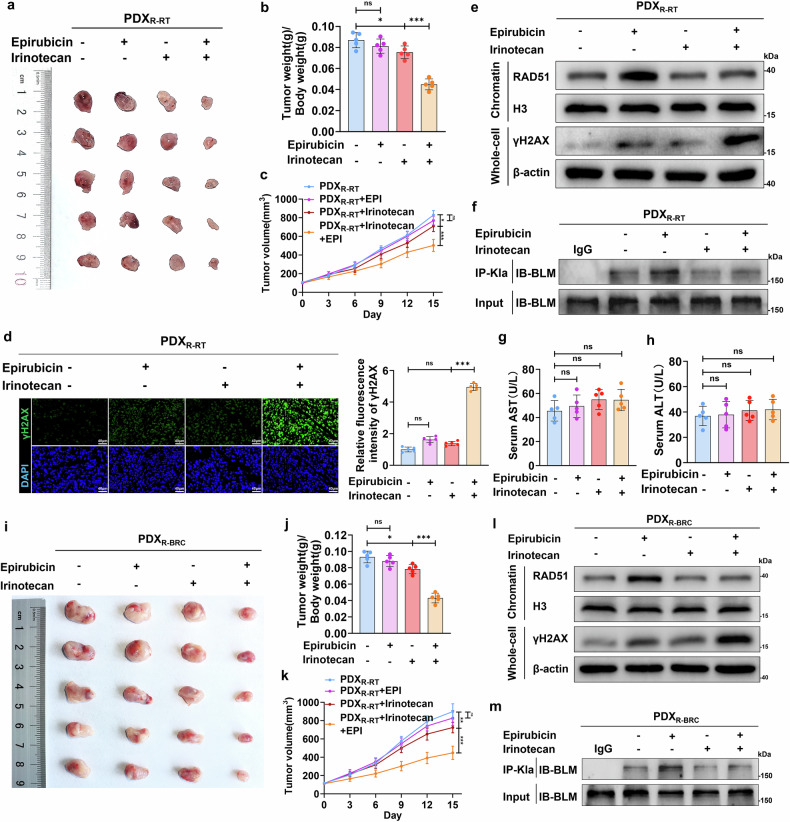
Combination therapy with irinotecan reverses EPI resistance by suppressing BLM lactylation and HR in pancancer PDX models. NSG mice were transplanted subcutaneously with NMIBC patient-derived xenografts (R-RT tumor tissues) and treated with EPI (2 mg/kg) and/or irinotecan (25 mg/kg) as indicated (*n* = 5); tumor images were acquired as shown in (**a**); tumor weights were measured (**b**), and volumes were calculated (**c**); one-way ANOVA followed by Tukey’s test. **d** Representative immunofluorescence images (left) and quantitative analyses (right) showing the nuclear expression and localization of γH2AX (green) colocalized with DAPI (blue) in PDX models; scale bar: 40 μm. **e** γH2AX expression and RAD51 chromatin accumulation were examined by western blot in PDX models. **f** Lactylated proteins were immunoprecipitated with anti-pan-Kla or IgG control in PDX models and analyzed by western blotting with anti-BLM. **g**, **h** Concentrations of ALT and AST in the serum of mice from different groups as indicated. NSG mice were transplanted subcutaneously with EPI-resistant breast cancer patient-derived xenografts and treated with EPI (2 mg/kg) and/or irinotecan (25 mg/kg) as indicated (*n* = 5); tumor images were acquired as shown in (**i**); tumor weights were measured (**j**), and volumes were calculated (**k**); one-way ANOVA followed by Tukey’s test. **l** γH2AX expression in whole-cell extracts and RAD51 accumulation in chromatin fractions were examined by western blot in PDX models. **m** Lactylated proteins were immunoprecipitated with anti-pan-Kla or IgG control in PDX models and analyzed via western blotting with anti-BLM. ***p < 0.001, **p < 0.01, *p < 0.05 represent significant differences between two groups; ns represents no significant difference

Next, we attempted to provide preclinical evidence for the efficacy of irinotecan treatment in patients with EPI failure. We isolated tumor tissues from patients with EPI-resistant bladder cancer and used them to develop patient-derived xenograft (PDX) NSG mouse models. Consistently, we found that irinotecan treatment increased the potency of the EPI-induced antitumor effects and showed remarkable synergistic effects in the EPI-treated PDX_R-RT_ models (Fig. 7a–c). Moreover, combination treatment with irinotecan and EPI upregulated γH2AX expression and decreased RAD51 chromatin accumulation in PDX tumors (Fig. 7d, e), accompanied by a decrease in BLM lactylation (Fig. 7f). No significant differences were noted in alanine transaminase (ALT) or aspartate aminotransferase (AST) levels in the serum of irinotecan-treated mice (Fig. 7g, h), indicating that irinotecan treatment did not increase hepatotoxicity. In addition, we also applied irinotecan in PDX mouse models via the use of EPI-resistant breast cancer tissues. We found that irinotecan treatment promoted EPI-induced antitumor effects in EPI-treated PDX_R-BRC_ models (Fig. 7i–k), which were accompanied by decreased HR repair and BLM lactylation levels (Fig. 7l, m). Collectively, these findings suggest the possibility of synergistic irinotecan treatment in anthracycline-resistant tumors. However, more preclinical and clinical trials are needed to corroborate the benefits and safety of this therapeutic strategy.

### The combination of irinotecan liposomes plus EPI is feasible and safe for anthracycline-resistant recurrent bladder cancer in a phase I study

Following our preclinical findings, we initiated a single-arm, phase I clinical trial to evaluate the safety and feasibility of combination therapy using irinotecan liposomes and EPI in patients with anthracycline-resistant recurrent bladder cancer (ClinicalTrials.gov identifier NCT06766266). The primary aim was to assess the dose-limiting toxicity (DLT) of irinotecan liposomes, and the secondary aim was to evaluate adverse events (AEs) and RFS. The trial was approved by the Ethics Committee of The First Affiliated Hospital of Chongqing Medical University (approval number 2024-549-02), and written informed consent was obtained from all participants or their legal guardians. The detailed inclusion and exclusion criteria are provided in Supplementary Fig. 10.

**Fig. 8:**
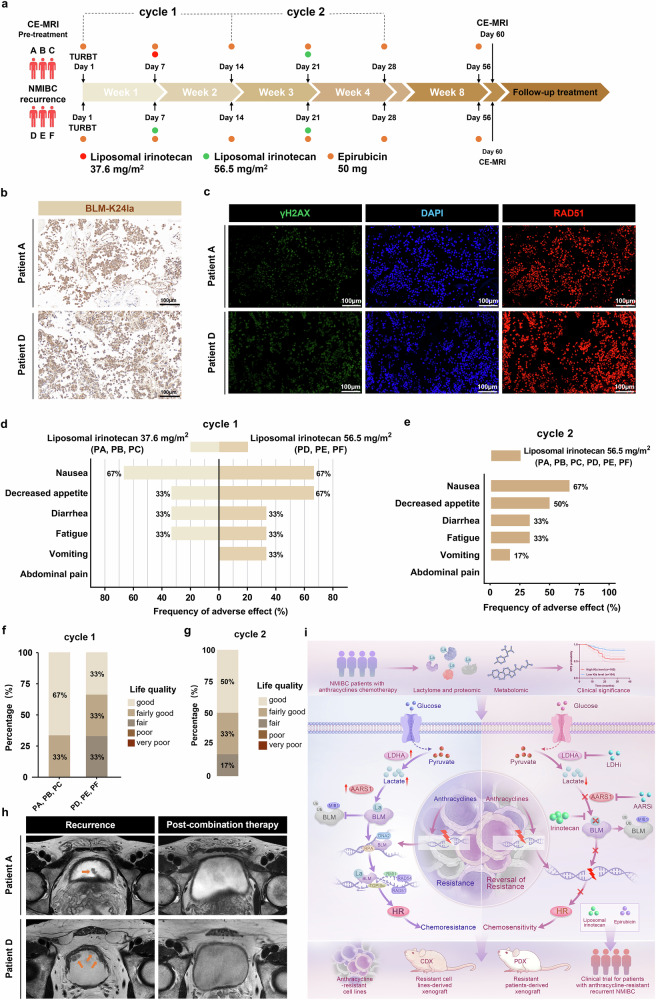
Safety, feasibility, and clinical response of combination treatment with irinotecan liposomes and EPI. **a** Diagram of the clinical trial design for the combination of irinotecan liposomes plus EPI for patients with anthracycline-resistant recurrent NMIBC. Participants will complete immediate intravesical instillation of EPI (50 mg) within 24 h after TURBT surgery. During the first month after surgery, participants will receive two cycles of combination treatment with irinotecan liposomes (administered intravenously once every two weeks for 1 month, with increasing doses of 37.6 mg/m^2^ and 56.5 mg/m^2^) and EPI (intravesical instillation, once a week for 1 month, 50 mg). All participants subsequently continued standard intravesical EPI chemotherapy (50 mg, once a month) until 6 months postsurgery. CE-MRI: contrast-enhanced magnetic resonance imaging, NMIBC: nonmuscle-invasive bladder cancer, TURBT: transurethral resection of a bladder tumor. **b** Representative BLM-K24la immunohistochemical staining of bladder tumor tissues from participants with anthracycline-resistant recurrent NMIBC; scale bar: 100 μm. **c** Representative immunofluorescence images showing nuclear expression and localization of γH2AX (green) and RAD51 (red) colocalized with DAPI (blue) in recurrent bladder tumor tissues; scale bar: 100 μm. **d**, **e** Frequency of adverse events (AEs) after each cycle of combination treatment. During the first cycle, the first three participants (Patients A, B, and C) received a reduced dose of irinotecan liposomes (37.6 mg/m^2^) without experiencing dose-limiting toxicity and subsequently escalated to the full dose (56.5 mg/m^2^) in the second cycle because of good tolerance; in addition, 3 participants (Patients D, E, and F) subsequently received the full dose of irinotecan liposomes during both treatment cycles. **f**, **g** Quality of life evaluation of participants after each cycle of combination treatment. **h** Representative CE-MRI images showing the bladder status of patients A and D during the recurrence period and after combination therapy; orange arrows indicate recurrent bladder tumors. **i** Model of how glycolytic reprogramming-induced BLM lactylation facilitates anthracycline resistance by activating HR repair (Drawn using GNU Image Manipulation Program)

To date, six patients with recurrent bladder cancer have been enrolled and received irinotecan liposomes (administered intravenously) in combination with standard intravesical EPI chemotherapy during the first month after surgical resection, according to a dose-escalation protocol, and subsequently, standard intravesical EPI chemotherapy was continued until 6 months after surgery. (Fig. 8a and Table S2). Hyperlactylation of BLM at Lys24 was confirmed in recurrent tumor tissues from enrolled patients through histologic analysis (Fig. 8b). In addition, decreased DNA damage was observed in recurrent tumor tissues, as evidenced by the downregulation of γH2AX expression shown in Fig. 8c. Patients A, B, and C received a reduced dose of irinotecan liposomes (37.6 mg/m^2^) during the first cycle without experiencing DLT and were subsequently escalated to the full dose (56.5 mg/m^2^) in the second cycle. Patients D, E, and F received the full dose of irinotecan liposomes during both treatment cycles. DLTs were assessed after each combination treatment cycle, and no DLTs have been reported in participants completing reduced and full doses of irinotecan liposomes until now (Tables S3 and S4). The most common AEs were grade 1–2 gastrointestinal toxicities, including nausea, decreased appetite, diarrhea, and fatigue. No grade 3–5 AEs have been reported to date (Table S3). During the first cycle, the incidence of AEs was similar between patients receiving reduced and full doses (Fig. 8d). Across all participants, the most frequent AEs following two cycles of combination treatment were nausea (67%), decreased appetite (50%), diarrhea (33%) and fatigue (33%) (Fig. 8e), none of which significantly impacted quality of life (Fig. 8f, g). Additionally, no abnormal changes in physiological parameters, including blood counts, cardiac enzyme profiles, or biochemical indicators, were observed (Table S4). In this interim analysis, no patients experienced disease recurrence, resulting in a recurrence-free rate (RFR) of 100%. Clinical benefits were evident in dynamic contrast-enhanced magnetic resonance imaging (Fig. 8h). Collectively, these preliminary results suggest that irinotecan liposomes combined with intravesical EPI chemotherapy constitute a safe and feasible therapeutic strategy for patients with anthracycline-resistant recurrent bladder cancer.

## Discussion

Anthracycline-containing chemotherapy substantially improves survival outcomes in patients with various cancers, including breast cancer, bladder cancer, and thymic carcinoma. However, the development of resistance to these agents poses a major therapeutic challenge. Metabolic reprogramming is essential for tumor occurrence and development, but its role in chemoresistance remains largely unknown. In the present study, we first characterized the metabolic and lactylome landscape in anthracycline-resistant and nonresistant cancer cells. We observed increased lactate and lactylation levels in anthracycline-resistant cancer cells and proposed a novel mechanism by which hyperlactylation of BLM by AARS1 promoted anthracycline resistance by activating HR repair in cancer cells. Targeted inhibition of BLM lactylation via synergistic irinotecan treatment was safe and effectively suppressed anthracycline resistance in preclinical models and a phase I clinical trial. Overall, we demonstrate the important role of glycolytic reprogramming and lactylation in anthracycline resistance and propose that irinotecan liposomes combined with EPI chemotherapy constitute a safe and feasible therapeutic strategy to reverse resistance in patients who are resistant to anthracyclines (Fig. 8i).

Upregulated glycolysis is a hallmark of malignant tumors and enables cancer cells to generate abundant energy in a short time. Lactate, a byproduct of glycolysis, has gradually been discovered to play an important role in promoting tumorigenesis by facilitating the proliferation of cancer cells, suppressing cancer stem cell differentiation, and accelerating neovascularization.^41^ Through dynamic crosstalk with diverse cellular components, lactate shapes the tumor microenvironment and modulates tumor progression.^42^ Furthermore, targeting lactate metabolism has become a promising therapeutic strategy in cancer. However, its role in chemoresistance remains unclear. Recently, lactate-induced lactylation has expanded our understanding of the function of lactate, which links metabolic reprogramming and epigenetic modifications.^43^ Like other PTMs, lactylation modulates the functions of histone and nonhistone proteins and influences cellular processes.^44^ Lactylated H3K18 was distributed mostly within the promoter regions of genes that are induced during hepatic stellate cell activation and liver fibrosis.^45^ The decline in IDH3β, a critical enzyme in the tricarboxylic acid cycle, increases histone H3 and H4 lactylation, which promotes expression of the transcription factor PAX6. Elevated PAX6 levels, in turn, further suppress IDH3β expression through a positive feedback loop, ultimately exacerbating neurodegeneration and cognitive impairment in patients with Alzheimer’s disease.^46^ Lactylation of PDHA1 and CPT2 led to their inactivation and inhibited oxidative phosphorylation by limiting acetyl-CoA supply from both pyruvate oxidation (via PDHA1) and fatty acid β-oxidation (via CPT2).^47^ AARS1 and AARS2 were recently demonstrated to sense lactate and mediate lactyl transfer and are considered panlactyltransferases that catalyze lactylation independently of lactyl-CoA.^48,49^ AARS1-mediated lactylation of p53 impairs its liquid-liquid phase separation and DNA-binding function, driving tumorigenesis.^40^ AARS2 associates with and lactylates cyclic GMP–AMP synthase (cGAS), which impairs cGAS-mediated self-DNA sensing. This dysfunction leads to compromised immune surveillance and promotes immune-related diseases.^50^ In the present study, AARS1 expression and glycolysis are upregulated in EPI-resistant cancer cells, leading to increased lactylation levels. Using the lactylome, we characterized differentially lactylated proteins in resistant and nonresistant cancer cells. KEGG and GO analyses revealed that multiple hyperlactylated proteins were enriched in pathways related to DNA repair. Furthermore, BLM hyperlactylation promoted chemoresistance by modulating HR repair. These results confirmed the important roles of metabolic reprogramming and epigenetic modifications in regulating cancer cell behavior.

HR repair is an evolutionarily conserved process for repairing damaged DNA that occurs daily in cells.^24^ Normal HR repair is essential for genome stability and supports faithful DNA replication. Dysfunction of HR repair is related to the development of multiple diseases, including tumorigenesis. HR repair comprises a series of subprocesses and involves the participation of multiple proteins, including RAD51, the MRE11-RAD50-NBS1 (MRN) complex, and BLM.^51^ Mutations or modifications in proteins have been shown to influence cancer risk. A recent study reported that lactylation of MRE11 and NBS1 leads to resistance to platinum drugs and poly (ADP-ribose) polymerase (PARP) inhibitors via activation of the HR repair process in colon cancer and gastric cancer.^52,53^ According to the lactylome results of the present study, no significant differences in MRE11 and NBS1 lactylation were noted between EPI-resistant and nonresistant cells. This finding may be attributed to distinct therapies or differences in cancer cells, and other differentially lactylated proteins that modulate EPI resistance in bladder cancer cells may exist. BLM, a highly conserved human RecQ helicase, functions as an upstream sensor protein in the DNA damage signaling cascade and plays an important role in HR repair processes.^33^ Recent data demonstrated that chemoresistance to camptothecin or oxaliplatin was primarily mediated by BLM-dependent enhancement of RAD54-mediated chromatin remodeling activity and that small molecule-induced conformational alteration of RAD54 disrupted the RAD54-BLM interaction and resensitized colon cancer cells to chemotherapy.^54^ In this study, we found that BLM lactylation was upregulated in EPI-resistant cancer cells and that hyperlactylation of K24 (as opposed to K31/K38 residues) enhanced BLM stability and its interactions with RAD54 and other key proteins in the HR process, thereby strengthening its essential role in HR repair. The K24R mutation or the small compound irinotecan, which targets BLM K24 lactylation, impairs HR repair and alleviates EPI resistance in bladder cancer cells.

Irinotecan is a potent and broad-spectrum anticancer drug that binds to the Top1-DNA complex and prevents religation of the DNA strand. The activity of irinotecan and its combination in metastatic bladder cancer shows nonoverlapping toxicity profiles and potent synergistic effects.^55^ Combinational treatment with irinotecan, EPI, and entinostat led to strong synergy between entinostat and cisplatin-resistant small-cell lung cancer.^56^ Furthermore, irinotecan improved progression-free survival in patients with metastatic biliary tract cancer after resistance and progression to gemcitabine plus cisplatin chemotherapy.^57^ For patients with advanced or metastatic pancreatic ductal adenocarcinoma after gemcitabine-based therapy, irinotecan demonstrates promising efficacy and manageable safety.^58^ In the present study, we demonstrated that irinotecan increases EPI sensitivity in EPI-resistant bladder cancer cells by targeting BLM K24 lactylation both in vitro and in vivo. These findings raise the preliminary possibility that combined treatment with irinotecan and EPI could be used for EPI-resistant bladder cancer. However, further conclusive evidence is necessary to support this strategy.

There are several limitations in the current study. First, our results provide convincing evidence that irinotecan suppressed BLM-K24 lactylation and reversed anthracycline resistance in different in vitro and in vivo models. However, the effects and mechanisms of irinotecan in reversing anthracycline resistance should be further validated in BLM-knockout PDX models and allograft models. Second, we elucidated the role of BLM lactylation in modulating chemoresistance by influencing HR repair; however, the roles of other differentially lactylated proteins remain largely unknown, and further exploration of these mechanisms is needed. Third, we demonstrated that BLM lactylation modulated its function and stability; however, lactylation-mediated protein structural changes are worth examining in future studies.

## Materials and methods

### Mice

The experiments and procedures were supported by the Ethics Committee of the First Affiliated Hospital of Chongqing Medical University. Five-week-old female nude BALB/c, NSG, and C57BL/6 J mice were purchased from AAALCA-accredited Model ORGANISMS (Shanghai, CHN). These mice were bred at the Animal Center of Chongqing Medical University at standardized specific pathogen-free facilities under a constant temperature of 23 °C and a photoperiod of 12 h light/12 h dark cycle and were provided ad libitum access to standard mouse chow and fresh water.

### PDX models

EPI-resistant bladder and breast tumor tissues were used to develop PDX NSG mouse models. The tumor tissues were immersed in RPMI 1640 medium and finely minced into fragments of approximately 2 mm^3^. Subsequently, the tumor fragments were subcutaneously transplanted into the flank of the right thigh of six-week-old NSG mice. Mice bearing tumors 100–120 mm^3^ in size were randomly assigned to various groups (*n* = 5/group) as indicated. The tumor volume was monitored and calculated every three days via the following standard formula: (width^2^ × length)/2. The peripheral blood samples of each mouse were collected via the right orbital vein for the detection of ALT and AST via the ALT and AST Activity Assay Kit (Solarbio, CHN) according to the manufacturer’s instructions. After euthanasia, the tumor tissues were collected for weighing, photography, histological examination, and other evaluations.

### CDX models

The CDX mouse models used were 6-week-old nude mice (*n* = 5/group) and C57BL/6 J mice (*n* = 5/group). A total of 10^6^ UM-UC-3 human bladder cancer cells or 5 × 10^5^ MB49 mouse bladder cancer cells resuspended in 100 μL of normal saline were subcutaneously injected into the flank of the right thigh of each mouse. The mice were administered EPI (2 mg/kg), sodium lactate (100 mg/kg), sodium oxamate (500 mg/kg), and/or irinotecan (25 mg/kg) when the tumor volume reached 100–120 mm^3^ (day 0).

### Cell culture and treatment

The human urothelial carcinoma cell line UM-UC-3, the human osteosarcoma cell line U2OS, the human breast carcinoma cell line MDA-MB-231 and HEK-293T cells were obtained from ATCC. The human hepatoma cell line Huh7 was obtained from the National Collection of Authenticated Cell Cultures. All the cell lines were routinely cultured in DMEM supplemented with 10% heat-inactivated fetal bovine serum (FBS; Gibco), 100 U/mL penicillin, and 0.1 mg/mL streptomycin at 37 °C in an atmosphere containing 5% CO_2_. All the cell lines were verified via short tandem repeat profiling. The Cell Culture Contamination Detection Kit (Thermo, USA) was used to confirm that the cells were free of *Mycoplasma* contamination. The development of E-resistant UM-UC-3 cells involved subjecting parental UM-UC-3 cells to incremental doses of EPI ranging from 10 nM to 100 nM until the E-resistant UM-UC-3 cells survived in DMEM containing 100 nM EPI. The process spanned approximately six months. In addition, the cells were treated with 0.2 μM EPI, 20 mM sodium lactate, 20 mM sodium oxamate, 27.4 μM B02, 14 nM KU-57788, or 4 μM irinotecan.

### Patient samples

All protocols used in this study were approved by the Medical Ethics Committee of the First Affiliated Hospital of Chongqing Medical University (2020-−36) and complied with all ethical regulations. Human tumor tissues were obtained from the First Affiliated Hospital of Chongqing Medical University after written informed consent was obtained from the participants. Primary and recurrent bladder tumor specimens were obtained from patients who underwent transurethral resection of bladder tumors and regular intravesical EPI chemotherapy or radical cystectomy between July 2020 and September 2023 (Table S1). The obtained samples were small in volume and therefore had no impact on the clinical diagnosis and treatment. All collected tumor samples were subjected to metabolomics, western blotting, immunohistochemistry, immunofluorescence, in vivo modeling, and other evaluations.

### Clinical trial

Eligible patients reported recurrence of bladder cancer after standard intravesical chemotherapy with anthracyclines, with diagnoses confirmed histologically. The other inclusion criteria for the trial were as follows: (1) recurrent tumor diagnosed as urothelial carcinoma with a clinical stage of cTa–cT1N0M0; (2) age ≥18 years; (3) no prior history of systemic chemotherapy; (4) Eastern Cooperative Oncology Group Performance Status (ECOG PS) of 0–1; and (5) provision of written informed consent. The exclusion criteria included the following: (1) severe cardiac, cerebral, hepatic, gastrointestinal, renal, or infectious diseases; (2) severe malnutrition; (3) mental illness or cognitive impairment that hinders the ability to comprehend or communicate effectively; and (4) the presence of malignancies in other organs. All patients considered for enrollment were fully informed about the study procedures, potential therapeutic risks and benefits, available treatment alternatives, and their rights as participants.

This single-arm, phase I trial followed a 3 + 3 dose-escalation scheme to evaluate the safety and tolerability of intravenous irinotecan liposomes (Hengrui Pharma) in combination with intravesical EPI chemotherapy. Participants will complete immediate intravesical instillation of EPI (50 mg) within 24 h after transurethral resection of bladder tumor (TURBT) surgery. During the induction phase (the first month postoperatively), the first three participants (Patients A, B, and C) received a reduced dose of irinotecan liposomes (37.6 mg/m^2^, once every two weeks) in combination with standard intravesical EPI chemotherapy (50 mg, once a week) during the first cycle. If the combination was well tolerated, the irinotecan liposome dose was escalated to the full dose (56.5 mg/m^2^, once every 2 weeks) in the second cycle. The remaining three participants (Patients D, E, F) received the full dose of irinotecan liposomes (56.5 mg/m^2^, once every two weeks) along with standard EPI (50 mg, once a week) in both treatment cycles. All participants subsequently continued standard intravesical EPI chemotherapy (50 mg, once a month) until 6 months postsurgery. The participants will visit the clinic once every two weeks for checkups and tests in the first month after surgery, and maintain regular and consistent follow-up. The clinical trial was approved by the Ethics Committee of The First Affiliated Hospital of Chongqing Medical Hospital (approval number 2024-549-02) and registered at ClinicalTrials.gov (NCT06766266).

### Lentiviral infection and the CRISPR/Cas9 system

To develop ER-AsiSI U2OS cells, U2OS cells were infected with lentivirus containing ER-AsiSI cDNA, and 8 μg/mL polybrene was added. Stable ER-AsiSI U2OS cells were screened with 5 μg/mL puromycin for two weeks. To generate stably overexpressing cell lines, UM-UC-3, MDA-MB-231, and Huh7 cell lines were infected with lentiviruses containing BLM, BLM-K24R, BLM-K31R, BLM-K38R, EP300, CBP, AARS1, or AARS1-5A cDNA fragments with HA or Flag tags along with the addition of polybrene at a concentration of 8 μg/mL. The cells were subsequently screened with 5 μg/mL puromycin or 500 µg/mL G418 for two weeks. To establish BLM-knockout (BKO) cell lines, the CRISPR/Cas9 system was used as previously described.^59^ Briefly, the pSpCas9 BB-2A-Puro (PX459) vector containing the individual guide sequences targeting BLM was transfected into UM-UC-3, MDA-MB-231, or Huh7 cells, followed by screening with 5 μg/mL puromycin for five days. All stable cells were detected by immunoblotting. Their oligonucleotide sequences are listed in Table S5.

### Plasmids and cell transfection

Human cDNA sequences corresponding to Ub (UniProt ID: P0CG47), MIB1 (UniProt ID: Q86YT6), TIP60 (UniProt ID: Q92993), and AARS1 (UniProt ID: P49588) were synthesized via PCR-based amplification from a human cDNA library and then subcloned and inserted into the pcDNA3.1 expression vector (cloning sites: KpnI/XhoI or KpnI/BamHI) containing a Flag or His tag to drive transient expression. The plasmids were validated via DNA sequencing. For cell transfection, the plasmids were combined with polyetherimide in serum-free medium, followed by incubation for 15 min. Subsequently, the mixture was added to the cells.

### Metabolite profiling

The isolated tumor tissues and cancer cells were washed twice with precooled normal saline. To remove proteins and extract metabolites, the tissues were smashed on ice, suspended in 400 μL of precooled methanol/acetonitrile solvent mixture (MeOH/ACN = 1:1, V/V, Merck, GER), vortexed for 30 s, and then centrifuged at 12,000 × *g* at 4 °C for 20 min. Then, the supernatant was incubated at −20 °C for 1 h and dried by rotary evaporation. The samples were subsequently redissolved in 100 μL of 50% ACN solvent (ACN/H_2_O = 1:1, V/V) and vortexed for 30 s. After recentrifuging, the supernatant was subjected to LC‒MS analysis (AB Sciex QTRAP 6500 LC‒MS/MS platform) for metabolite detection and untargeted metabolomic profiling as previously described.^60^

### Identification of proteins with lysine lactylation by LC‒MS/MS analysis

Bladder cancer cells isolated from CDX_Parental_ + EPI and CDX_E-resistant_ + EPI tissues were snap-frozen in liquid nitrogen and supplemented with urea lysis buffer for protein extraction. After sonication (Scientz, CHN) three times on ice, the supernatant was quantified via a BCA Kit (Beyotime, CHN). Subsequently, equal amounts of protein from each group were mixed dropwise with trichloroacetic acid (TCA, Sigma–Aldrich, CHN) at a final concentration of 20% and then precipitated at 4 °C for 2 h. Then, the pellet was collected and washed twice with precooled acetone (Sigma–Aldrich). The pellet was resuspended in 200 mM triethylammonium bicarbonate buffer (TEAB, Sigma–Aldrich, CHN), treated with trypsin (trypsin/protein = 1:50, W/W, Promega, USA), and digested overnight for peptide preparation. The resulting mixture was subsequently incubated with 5 mM dithiothreitol (Sigma–Aldrich, CHN) at 37 °C for 1 h and supplemented with 11 mM iodoacetamide (Sigma–Aldrich, CHN) for 30 min at room temperature (RT).

For enrichment of the lactylated peptides, anti-L-lactyl-lysine antibody-conjugated agarose beads (PTM-1404, PTM Bio, CHN) were washed twice with precooled PBS. The prepared peptides were then suspended in NP-40 buffer (Beyotime, CHN) and mixed with prewashed anti-L-lactyllysine antibody beads, followed by overnight incubation at 4 °C with gentle rotation. Next, the beads were harvested by centrifugation at 1000 × *g* for 1 min at 4 °C and rewashed with NP-40 buffer and deionized water to collect the enriched lactylated peptides. The beads bound to the enriched lactylated peptides were eluted twice via TCA at a concentration of 0.1%, followed by purification via C_18_ ZipTips (Sigma–Aldrich, CHN).

For lactylation analysis, LC‒MS/MS was conducted via a Bruker timeTOF Pro mass spectrometer combined with a NanoElute ultrahigh-performance system (Bruker, GER). Mobile phase A was an aqueous solution containing 0.1% formic acid and 2% acetonitrile. Mobile phase B was an acetonitrile-aqueous solution containing 0.1% formic acid. The liquid phase gradient conditions were as follows: 0–42 min, 7–24% B; 42.0–54.0 min, 24–32% B; 54.0–57.0 min, 32–80% B; and 57.0–60.0 min, 80% B. The flow rate was maintained at 450 nL/min. The peptides were separated by an ultrahigh-performance liquid phase system, injected into a capillary ion source for ionization and then analyzed by timsTOF Pro (Bruker) mass spectrometry. The ion source voltage was set to 2.0 kV, and the parent ions of the peptide segment and its secondary fragments were detected and analyzed via high-resolution TOF. The secondary mass spectrometry scan range was set to 100–1700 m/z. PASEF mode was used for data acquisition. A secondary spectrum with the charge number of parent ions in the range of 0-−5 was collected in PASEF mode 10 times after primary mass spectrometry collection. The dynamic exclusion time of series mass spectrometry scanning was set to 30 s to avoid repeated scanning of parent ions.

In addition, the raw MS data from each group of peptides were identified and quantified via MaxQuant (v1.6.15.0) software. The search parameters were set as follows: the database was Homo_sapiens_9606_SP_20220107.fasta (20376 sequence); an antidatabase was added to calculate the false positive rate caused by random matching; a common contamination database was added to the database to eliminate the influence of contaminated proteins; the enzyme digestion method was set as Trypsin/P; the number of missing cuts was set to 4; the minimum length of the peptide was set to 7 amino acid residues; the maximum number of peptide modifications was set to 5; and the mass error tolerance for primary parent ions was set at 20 ppm for the first search and main search and 20 ppm for secondary fragment ions. Carbamidomethylation was considered a fixed modification. Methionine oxidation, N-terminal acetylation, and lysine lactylation were considered variable modifications. The false discovery rate did not exceed 1%.

### Measurement of lactate concentration

Bladder tumor tissues and cancer cells were lysed in extraction buffer by sonication or homogenization at 4 °C and then centrifuged at 12,000 × *g* for 10 min, followed by collection of the supernatant. The absorbance values of the supernatant and standard solutions were measured at 570 nm via a colorimetric lactate content assay kit (Solarbio, CHN) according to the manufacturer’s protocol. The lactate concentration was calculated via a standard curve.

### DNA end resection assay

For the DNA end-resection assay, ER-AsiSI U2OS cells were transfected with plasmids containing BLM or BLM-K24R cDNA fragments and then treated with 20 mM sodium lactate or 20 mM sodium oxamate for 24 h. The cells were treated with 2 μM 4-OHT or DMSO for 4 h. Subsequently, genomic DNA was extracted via the DNAzol reagent (Invitrogen, USA) according to the manufacturer’s protocol and subjected to BsrGI enzyme (New England Biolabs, USA)-mediated digestion at 37 °C overnight. A subsequent qPCR-based method was used to analyze single-stranded DNA (ssDNA) generation. In the qPCR mixture (25 μL), 2 μL of DNA was added as a template, along with 12.5 μL of 2× Taqman Universal PCR Master Mix (Applied Biosystems, USA) containing 0.2 μM of each primer and 0.2 μM probe. The primer and probe sequences used for qPCR are listed in Table S5. The percentage of ssDNA was quantified via the following equation: ssDNA% = 100/(2^(ΔCt–1)^ + 0.5). The efficiency of end-resection was evaluated by determining the percentage of single-stranded DNA (ssDNA) generation relative to the cutoff efficiency, as previously described.^61^

### Expression and purification of recombinant proteins

Bacterial expression constructs (pET28a-sumo) containing the indicated genes were transformed into *Escherichia coli* BL21 (DE3), which were induced to express protein via 0.2 mM isopropyl β-D-thiogalactopyranoside (IPTG) at 20 °C with 160 rpm rotation for 20 h. Then, the harvested bacterial pellet was resuspended in 35 mL of lysis buffer (20 mM Tris-HCl, pH = 7.5, 500 mM NaCl, 5% glycerol, 20 μg/mL lysozyme, 1 mM PMSF, 1 mM DTT, and 1 mM EDTA) and subjected to ultrasonic cell disruption via an ultrasonic homogenizer. The lysate was clarified by ultracentrifugation at 15,000 × *g* for 30 min at 4 °C. The supernatant was filtered through a 0.45 μm membrane and subsequently loaded onto an AKTA pure chromatography system for protein purification.

### Surface plasmon resonance assay

SPR experiments were conducted on a Biacore T200 instrument (Cytiva, Sweden) via a Series S Sensor Chip CM5 (Lot #10344853). The running buffer (10 × PBS, 0.05% Tween-20, and 1% DMSO) was degassed prior to use. Ligand immobilization was performed by activating the CM5 chip with a 1:1 mixture of 0.4 M EDC and 0.1 M NHS (Amine Coupling Kit, Cytiva) at a flow rate of 10 μL/min. The target protein was injected in 10 mM sodium acetate (pH = 4.0) until 5000 RU was achieved. Blocking was performed using ethanolamine at 10 μL/min. Small-molecule analytes were dissolved in PBST (1% DMSO) and serially diluted. Binding kinetics were measured at 25 °C via multicycle kinetics (30 μL/min, 90 s association, 300 s dissociation). Three startup cycles and blank injections were performed prior to analysis. Sensorgrams were double-referenced, and kinetic parameters (*k*_on_, *k*_off_) and equilibrium dissociation constants (*K*_D_) were calculated via Biacore Evaluation Software (Cytiva) with global fitting to a 1:1 Langmuir binding model.

### Electrophoretic mobility shift assay

Purified human WT and K24R BLM proteins were incubated at the indicated concentrations with 5′-end-labeled 50-bp dsDNA and 3′-tailed-labeled DNA substrates (Table S5) in reaction buffer (25 mM Tris-HCl, pH = 7.5, 5 mM MgCl_2_, 1 mM DTT, 100 mg/mL BSA, 2 mM ATP) containing 100 mM KCl at room temperature for 30 min. Prior to loading, the gels were run at 120 V for 30 min, and each well was flushed with 0.25x TBE. The reaction mixtures were subsequently combined with loading buffer and separated via electrophoresis on 5% native polyacrylamide gels in 0.25x TBE buffer. Images were acquired via an infrared fluorescence imaging system (LI-COR Odyssey 9120).

### Helicase assay

Reactions were performed using 75 nM nucleotides (5′-end-labeled 50-bp dsDNA and 3′-tailed-labeled DNA, Table S5) in standard buffer (20 mM Na-HEPES, pH = 7.5, 2 mM ATP, 0.1 mM DTT, 100 mg/mL BSA, 0.05% Triton-X100, and 5 mM MgCl_2_) at 37 °C for 30 min. BLM protein was used at concentrations ranging from 10 to 20 nM, and RPA protein was used at 20 nM. Reactions were assembled on ice and initiated by transfer to 37 °C. To terminate the reactions, termination buffer containing 2 mg/mL proteinase K, 50 mM EDTA, and 1% SDS was added, and the mixture was incubated for 30 min. In addition, a 20-fold molar excess of identical but unlabeled oligonucleotide was included in the termination buffer to prevent spontaneous reannealing of the unwound substrate strands. The reaction products were resolved via electrophoresis on 12% native polyacrylamide gels in 1x TAE buffer.

### Comet assay

For the comet assay, 10^4^ cells were embedded in 0.7% agarose with a low melting point covered with 1% agarose with a normal melting point on the glass side. Next, additional cell-free 0.7% agarose with a low melting point was used to cover a layer containing pretreated cells embedded in 0.7% agarose with a low melting point. After cooling at 4 °C for 30 min, the three layers of agarose on the glass side were immersed in lysis buffer for 2 h at 4 °C. The slides were subsequently incubated with electrophoresis buffer for 30 min to unwind the DNA, and electrophoresis was performed at 25 V for 25 min. The slides were stained in the dark with PI for 10 min. For quantification, the head and tail DNA were captured via a fluorescence microscope, and the percentage of tail DNA was calculated via the CASP software.

### Immunohistochemistry

Bladder cancer tissue samples were fixed in 4% formaldehyde (Biosharp, CHN) and embedded in paraffin. The paraffin blocks were subsequently prepared as 4 μm thick sections on slides, followed by deparaffinization with xylene and rehydration using a series of decreasing ethanol concentrations. The slides were subsequently immersed in sodium citrate (Beyotime, CHN) and subjected to heat treatment at 95 °C for antigen retrieval. Then, the slides were covered with 3% H_2_O_2_ for 10 min and blocked with 5% goat serum for 20 min, followed by incubation with anti-pan-Kla primary antibody (PTM BIO, CHN) at 4 °C overnight. The slides were then incubated with horseradish peroxidase (HRP)-conjugated secondary antibodies for 20 min and stained with diaminobenzidine. Slides were scanned via the Panoramic SCAN (3DHISTECH, HUN), and the integral optical density of Pan-Kla was further analyzed via ImageJ software.

### Western blot analysis

Proteins were extracted via RIPA buffer (Beyotime, CHN) containing 1% protease inhibitor (Abcam, UK), followed by sonication or homogenization at 4 °C. Subcellular protein fractionation was performed with a subcellular protein fractionation kit (Thermo, USA). The protein concentration was quantified via a BCA Kit (Beyotime, CHN). Equal amounts of proteins were separated by SDS‒PAGE and subsequently transferred onto polyvinylidene fluoride membranes (Millipore, USA). After being blocked with 5% skim milk, the membranes were incubated with the indicated primary antibodies overnight at 4 °C and then incubated with species-matched secondary antibodies (1:5000; Proteintech) at room temperature for 1 h. The membranes were visualized via WesternBright ECL (Advansta, USA) and Fusion FX (VILBER, FRS). The primary antibodies used in this study are listed in Table S6.

### Immunoprecipitation and nickel pulldown assays

Pretreated cells were lysed in NP-40 buffer (Beyotime, CHN) containing a protease inhibitor cocktail (Selleck, CHN) at 4 °C for 30 min. Then, the cell supernatant was incubated with 2.5 μg of species-matched immunoglobulin G (IgG) or equal amounts of anti-BLM, anti-HA, or anti-Flag antibodies at 4 °C. After overnight incubation, the supernatant was subsequently supplemented and incubated with 30 μL of protein A/G magnetic beads (MCE, CHN) at 4 °C for 4 h. Next, the magnetic beads were washed three times with NP-40 lysis buffer and then boiled in 1× SDS loading buffer (Beyotime, CHN) for further western blot analyses. To reduce the masking of IgG bands (heavy and light chains), anti-mouse and anti-rabbit IgG Veriblot secondary antibodies (ab131368, ab131366, Abcam) were used to detect immunoprecipitated proteins at a dilution of 1:5000.

Nickel pulldown assays were conducted as previously described.^38^ The cells were cotransfected with constructs encoding HA-BLM (or HA-K24R BLM) and His-ubiquitin. After 48 h of transfection, the cells were lysed in denaturing buffer (6 M urea, 300 mM NaCl, and 10 mM imidazole in 100 mM sodium phosphate, pH = 7.4) and incubated with Ni-NTA agarose beads at 4 °C for 2 h. The purified complexes were subsequently washed and subjected to western blot analysis.

### Quantitative real-time PCR

Total RNA was extracted from E-resistant cells via RNAiso Plus (Takara, JPN). A total of 1 μg of purified RNA was reverse transcribed via ABScript Neo RT Master Mix for qPCR with gDNA Remover (ABclonal, CHN). Quantitative real-time PCR was conducted with 2X Universal SYBR Green Fast qPCR Mix (ABclonal, CHN). TOP I gene expression was normalized to that of β-actin.

### BLM protein stability assay

Cells transfected with BLM-HA or BLM-K24R-HA plasmids were administered 40 μM cycloheximide with or without 20 mM sodium lactate. After 0, 12, 24, and 36 h of treatment, total protein was extracted via RIPA buffer, and the extract was subjected to western blot analysis via an anti-HA antibody for the quantitative detection of protein degradation.

### Cell viability assay

Cell viability was assessed via a Cell Counting Kit-8 (Dojindo, JPN). A total of 4000 cells/well were seeded into 96-well plates. Subsequently, EPI, irinotecan, sodium lactate, sodium oxamate, or DMSO was administered at the indicated dose in the medium for 6 h, 24 h, 48 h, 72 h, or 96 h. After the cells were incubated with 10% CCK-8 reagent in complete DMEM for 2 h, the absorbance was measured at 450 nm via a microplate reader (TECAN, USA).

To measure the half maximal inhibitory concentration (IC50) values of EPI in parental and E-resistant cells, the cells were seeded into 96-well plates at a density of 12,000 cells/well and permitted to adhere for 12 h. Subsequently, the cells were administered incremental doses of EPI at 50 nM, 100 nM, 250 nM, 500 nM, 1 μM, and 2.5 μM. After 48 h of incubation, the cells were incubated with 10% CCK-8 reagent in complete medium for 2 h, and the absorbance was measured at 450 nm via a microplate reader. The survival rate of the EPI-exposed cells was calculated and normalized to the absorbance of the control group.

### Immunofluorescence

Immunofluorescence assays were conducted as previously described.^62^ The cells were fixed in 4% paraformaldehyde (Beyotime, CHN) for 10 min and permeabilized with 0.25% Triton X-100 at room temperature for 5 min, followed by blocking with Immunol staining blocking buffer (Beyotime, CHN) for 15 min. Next, the cells were incubated with anti-RAD51 and anti-γH2AX primary antibodies at 4 °C overnight, followed by incubation with secondary antibodies for 1 h. Then, the cells were stained with DAPI (Beyotime, CHN) for 10 min. Following deparaffinization, rehydration, antigen retrieval, and blocking, the tissue slides were incubated with anti-ki-67, anti-γH2AX, and/or anti-RAD51 primary antibodies at 4 °C overnight. The slides were then incubated with secondary antibodies for 1 h. After being stained with DAPI for 10 min, images were captured with a laser confocal microscope (Leica Microsystems AG).

### Colony formation assay

The cells were treated with EPI, irinotecan, sodium lactate, sodium oxamate or DMSO at the indicated doses and incubated for 10–14 days until visible colonies formed. Colonies were fixed with 4% formaldehyde for 10 min and stained with 0.1% crystal violet (Beyotime) for 20 min. The number of colonies was counted via ImageJ software.

### Molecular docking

The PDB structure of BLM (P54132) was obtained from the AlphaFold protein structure database (https://alphafold.ebi.ac.uk/). A set of small-molecule drugs classified as ATC Code level 1 as class L was assembled from the DrugBank database. These chemical structures of the compounds were represented in SDF format, and their geometries were optimized in the MMFF94s force field via the RDkit program (https://www.rdkit.org, open-source cheminformatics). The three-dimensional spatial coordinates of the K-24 residues were identified via the GetBox plugin within PyMOL software. The Swiss PDB Viewer program was used to process the structure of the BLM-K24R mutant.^63^ The AutoDock Tools (version 1.5.6) program was used to add hydrogen to the BLM structure, calculate the Gasteiger charge, and incorporate a nonpolar hydrogen of the protein. Semiflexible molecular docking was performed via AutoDock Vina software (version 1.1.2),^64^, and each small molecule in the compound set was docked with the BLM WT and BLM K24R structures five times. The docking poses of the small-molecule drugs with the target protein were visualized via PyMOL software.

### Molecular dynamics simulation

Molecular dynamics simulations were performed to investigate the binding stability of irinotecan and the BLM protein via GROMACS (gmx2020.6_GPU) software. The ORCA program was used to optimize the DFT level of irinotecan on the basis of r2SCAN-3c. Water was used as the SMD solvent. The single-point energy was calculated via the B3LYP/GD3 DEF2-TZVP def2/J RIJCOSX base set. The topological structures of irinotecan were created via the Antechamber tool on the basis of the GAFF2 force field, and its RESP charges were fitted via Multiwfn.^65^ The AMBER99SB-ILDN force field was utilized to obtain the topological structures of the WT and K24R BLM proteins. The TIP3P water model was used for each simulation system. Counterions were added to neutralize the total charge of the system. Energy minimization was performed via the steepest descent method with a maximum of 1000 kJ/mol/nm and then minimized to 500 kJ/mol/nm via conjugate gradient optimization. Next, the system was first equilibrated for 5 ns under the isothermal-isochoric (NVT) system and then for 1 ns under the isothermal-isobaric (NPT) system by using a leap-frog integrator, reaching the corresponding simulated temperature (300.15 K) and pressure (0.1 MPa). The balanced system was simulated under the NPT system for 100 ns of all-atom molecular dynamics. A 10 ns unlimited simulation was performed for the BLM protein. The simulated temperature (300.15 K) and pressure (1.0 bar) were controlled via a V-rescale thermostat and a Parrinello Rahman regulator with time constants of 0.1 and 2 ps, respectively. The LINCS algorithm was used for all key constraints in the system. Long-range electrostatic interactions were calculated via the particle‒mesh Ewald method with a time step of 2 fs. The binding free energy between irinotecan and the K24 region of the BLM protein was calculated via the MMGBSA method via the gmx_MMPBSA tool.^66^

### Quantification and statistical analysis

GraphPad Prism 9.5.1 (GraphPad, USA) and SPSS version 26.0 (SPSS, USA) were used for all the statistical analyses. The data are presented as the means ± SDs of at least three independent experiments. Two-tailed unpaired Student’s t tests or paired t tests were performed to compare the statistical significance between 2 groups. One-way ANOVA with Tukey’s post hoc test was used to compare the statistical significance among multiple groups. Pearson’s correlation coefficient was calculated for linear correlation analysis. Differences were considered statistically significant at *p < 0.05, **p < 0.01, and ***p < 0.001.

## Supplementary information

Revised Supplementary_Materials

Data S1

Data S2

Study protocol

## Data Availability

Lactylome and proteomics data have been deposited to the ProteomeXchange Consortium via the PRIDE partner repository (http://www.ebi.ac.uk/pride) with the dataset identifier PXD062720 and are publicly available as of the date of publication. The raw metabolomic data are available in the Figshare repository (10.6084/m9.figshare.29279096) and the Data S2 document. The original western blot images used to generate the figures throughout the manuscript can be found within the Data S1 document. All additional datasets included in the manuscript will be shared by the lead contact upon request.
