# Supplementary material for: Irinotecan alleviates chemoresistance to anthracyclines through the inhibition of AARS1-mediated BLM lactylation and homologous recombination repair
Source: Signal Transduct Target Ther. 2025 Jul 10;10:214. doi: 10.1038/s41392-025-02302-y (PMC12241633; doi:10.1038/s41392-025-02302-y)
Supplement: Supplementary file 1 — Revised Supplementary_Materials [file 41392_2025_2302_MOESM1_ESM.docx]

Supplementary Materials for

Irinotecan alleviates chemoresistance to anthracyclines through inhibition of AARS1-mediated BLM lactylation and homologous recombination repairs

Xinyuan Li, Chunlin Zhang, Yuhua Mei, Wenlong Zhong, Wei Fan, Li Liu, Zhenwei Feng, Xuesong Bai, Chuan Liu, Mingzhao Xiao, Weiyang He, Tianxin Lin, and Xin Gou.

Correspondence to: [gouxincq@163.com](mailto:gouxincq@163.com); [lintx@mail.sysu.edu.cn](mailto:lintx@mail.sysu.edu.cn); [weiyang361@163.com](mailto:weiyang361@163.com)

**This PDF file includes:**

Figures. S1 to S10

Tables S1 to S6

**Other Supplementary Materials for this manuscript include the following:**

Data S1 [The original western-blot images]

Data S2 [The raw metabolomics data]

Study Protocol





**Figure. S1. Lactate and lactylation levels are upregulated in EPI-resistant tumor samples and cells. a** The intensity of lactate in NR-PT, R-PT, and R-RT bladder tumor samples from patients with non-resistant NMIBC (n = 4) and patients with resistant NMIBC (n = 4); one-way ANOVA followed by Tukey's test. **b, c** Pan-Kla, LDHA and β-actin levels were determined by western blot in NR-PT, R-PT, and R-RT bladder tumor samples from patients with non-resistant NMIBC and patients with resistant NMIBC. **d** Half maximal inhibitory concentration (IC50) of EPI in Parental and E-resistant UM-UC-3 cell lines were determined by cell viability assays, with the resistance index (RI) of 10.14. **e** Cell viability assays detecting the viability of Parental and E-resistant UM-UC-3 cells before and after treatment with 0.2 μM EPI; one-way ANOVA followed by Tukey's test. **f** Same experimental setup as in e but displaying cell proliferation by colony-formation assays; one-way ANOVA followed by Tukey’s test. **g** Western blot analysis was performed to examine LDHA, pan-Kla, and β-actin in whole-cell extracts of Parental and E-resistant UM-UC-3 cells treated with the same experimental setup as described in e. **h** Representative images of comet assays performed with Parental and E-resistant UM-UC-3 cells treated with the same experimental setup as described in e; the percentage of DNA tail was compared by one-way ANOVA followed by Tukey's test; scale bar: 100 μm. Except for panels A-C, graphs represent three replicates per condition (n = 3). ****p <* 0.001, ***p <* 0.01, **p <* 0.05 represent significant differences between two groups.

**

Figure. S2. Tumor growth is inhibited and lactate level is upregulated in CDX_E-resistant_ models.** Nude mice were transplanted subcutaneously with Parental and E-resistant UM-UC-3 cells and treated with EPI (2 mg/kg) or untreated (n = 5); tumor images were acquired as shown in (**a**); tumor weights were measured (**b**) and volumes were calculated (**c**); one-way ANOVA followed by Tukey’s test. **d** Representative immunofluorescence images show expression of ki-67 (green) and merged with DAPI (blue) of nuclei in CDX models (n = 3); scale bar: 50 μm. **e** The intensity of lactate in tumor cells separated from EPI-treated CDX_E-resistant_ and CDX_parental_ models (n = 3); unpaired two-tailed Student’s t test. ****p <* 0.001, ***p <* 0.01, **p <* 0.05 represent significant differences between two groups.

**

Figure. S3. Inhibition of lactate production and homologous recombination reverses EPI resistance in pan-cancer. a** E-resistant UM-UC-3 cells were treated with 0.2 μM EPI, 20 mM sodium lactate, and (or) 20 mM sodium oxamate as indicated; representative images show cell proliferation levels determined using colony-formation assays and quantitative assays; one-way ANOVA followed by Tukey’s test. **b** Nude mice were transplanted subcutaneously with E-resistant UM-UC-3 cells and treated with EPI (2 mg/kg), sodium lactate (100 mg/kg) and (or) sodium oxamate (500 mg/kg); tumor weights were measured; one-way ANOVA followed by Tukey’s test. IC50 of EPI in Parental and E-resistant MDA-MB-231 breast cancer cell lines (**c**) and Huh7 hepatocellular carcinoma cell lines (**d**) were determined using cell viability assays, with the resistance index (RI) of 11.79 and 9.75 respectively. Western blot analyses were conducted to examine γH2AX and β-actin using whole-cell extracts from E-resistant UM-UC-3 (**e**), MDA-MB-231 (**f**), and Huh7 (**g**) cells before and 24 h after treatment with 0.2 μM EPI, 27.4 μM B02, and (or) 14 nM KU-57788. **h-j** Same experimental setup as in e-g but displaying cell viability by cell viability assays; one-way ANOVA followed by Tukey’s test. Graphs represent three replicates per condition (n = 3). ****p <* 0.001, ***p <* 0.01, **p <* 0.05 represent significant differences between two groups.

**

Figure. S4. BLM-K24 lactylation facilitates anthracycline-based drug resistance. a** Western blot analysis of BLM expression in E-resistant UM-UC-3 cells and their BLM-knockout (BKO) counterparts reconstituted with either BLM-WT or the K24R/K31R/K38R mutants. Illustration of BLM-K31 (**b**) and BLM-K38 (**c**) lactylation identified by MS. BLM-K24 lactylation levels were determined by western blot with K24-lactylation specific antibody in parental and E-resistant MDA-MB-231 (**d**) and Huh7 (**e**) cells before and 24 h after treatment with 0.2 μM EPI. **f** Parental and E-resistant UM-UC-3 cells were used for immunoprecipitation using anti-BLM antibody or IgG control; blots were probed with anti-BLM and anti-pan-Kla antibodies. **g** Parental-BKO and E-resistant-BKO UM-UC-3 cells transfected with HA-tagged BLM plasmids were treated with 0.2 μM EPI or untreated; HA-tagged BLM proteins were immunoprecipitated with anti-HA antibody; western blot determining BLM lactylation using anti-pan-Kla and anti-HA antibodies. **h** E-resistant UM-UC-3 cells treated with 0.2 μM EPI, 20 mM sodium lactate, and (or) 20 mM sodium oxamate were used for immunoprecipitation using anti-BLM antibody or IgG control; blots were probed with anti-BLM and anti-pan-Kla antibodies. **i** E-resistant-BKO UM-UC-3 cells transfected with HA-tagged BLM plasmids were administrated with 0.2 μM EPI, 20 mM sodium lactate, and (or) 20 mM sodium oxamate; HA-tagged BLM proteins were immunoprecipitated with anti-HA antibody; western blot detecting BLM lactylation using anti-pan-Kla and anti-HA antibodies. **j, k** Cell viability assays determining viability of E-resistant cells and E-resistant-BKO cells transfected with WT, K24R, K31R, and K38R BLM plasmids; cells were treated with 0.2 μM EPI; one-way ANOVA followed by Tukey’s test (J indicates the MDA-MB-231 cell line and K indicates Huh7). **l** IC50 of pirarubicin (THP) in Parental and THP-resistant UM-UC-3 cells was determined by cell viability assays, with the resistance index (RI) of 10.81. **m** Cell viability assays examining viability of T-resistant and T-resistant-BKO UM-UC-3 cells transfected with WT and K24R BLM plasmids after treatment with 0.3 μM THP; one-way ANOVA followed by Tukey’s test. **n** Same experimental setup as in e but displaying cell proliferation by colony-formation assays; one-way ANOVA followed by Tukey’s test. Graphs represent three replicates per condition (n = 3). ****p <* 0.001 and **p <* 0.05 represent significant differences between two groups.





**Figure. S5. BLM-K24 lactylation regulates protein functions and facilitates HR repair.** Western blot analyses were performed to examine the RAD51 chromatin accumulation and γH2AX expression in E-resistant-BKO MDA-MB-231 (**a**) and Huh7 (**b**) cells treated with 0.2 μM EPI and 20 mM sodium lactate for 24h or untreated. **c** AsiSI-ER U2OS system and qPCR-based quantification analysis were used to detect ssDNA generated by DNA end resection in cells transfected with WT, K24R, or K24A BLM plasmids before and 24 h after treatment with 20 mM sodium lactate; the ssDNA level was normalized according to the results in BLM-WT (+4OHT) group; one-way ANOVA followed by Tukey’s test. **d** Representative images of comet assays performed with E-resistant UM-UC-3 cells transfected with WT or K24R BLM plasmids after treatments with 0.2 μM EPI, 20 mM sodium lactate, and (or) 20 mM sodium oxamate for 24 h; scale bar: 100 μm. **e** BLM–DNA complex migration distance was determined using the electrophoretic mobility shift assays (EMSA); 1.5 nM of 5′-end-labeled 50-bp dsDNA and 3′-tailed-labeled DNA were separately incubated at room temperature for 30 min with 0.6 mM WT and K24R BLM proteins in a binding buffer; PC and NC indicate the positive control (human GATA1) and negative control. **f** E-resistant-BKO UM-UC-3 cells transfected with HA-tagged WT or K24R BLM plasmids were treated with 20 mM sodium lactate or 20 mM sodium oxamate for 24 h; HA-tagged BLM proteins were immunoprecipitated with anti-HA antibody or IgG control; blots were probed with anti-HA and target antibodies as indicated. **g** BLM-mediated unwinding reactions were performed using 5′-end-labeled 50-bp DNA and 3′-tailed-labeled DNA fragments; reactions contained 10-20 nM WT and K24R BLM, 20 nM RPA and 5 mM MgCl_2_. **h** Molecular dynamics simulation using the GROMACS software package with the AMBER99SB-ILDN force field detected that K24R mutation altered BLM protein structure with the Root Mean Square Deviation (RMSD) of 1.40 nm. Graphs represent three replicates per condition (n = 3). ****p <* 0.001, ***p <* 0.01, **p <* 0.05 represent significant differences between two groups; ns represents no significant difference.





**Figure. S6. BLM-K24 lactylation suppresses MIB1-mediated BLM ubiquitination and increases protein stability. a, b** Half-life detection and quantitative analysis of the WT, K31R, and K38R BLM proteins in E-resistant-BKO UM-UC-3 cells treated with 40 μM cycloheximide for indicated times. **c** Parental and E-resistant UM-UC-3 cells cotransfected with HA-BLM and Flag-Ub plasmids were used for immunoprecipitation using anti-HA antibody or IgG control; blots were probed with anti-HA and anti-Flag antibodies. **d** E-resistant UM-UC-3 cells were cotransfected with HA-BLM, Flag-Ub, and (or) His-MIB1 plasmids; HA-tagged BLM proteins were immunoprecipitated with anti-HA antibody and analyzed by western blot with antibodies as indicated. **e, f** Molecular docking diagram showing the docking pose of WT and K24R BLM interacting with MIB1; the PIPER energies of the top 30 docking poses between BLM and MIB1 were compared using unpaired two-tailed Student’s t test. **g** E-resistant-BKO UM-UC-3 cells were cotransfected with His-Ub, HA-WT BLM, and (or) HA-K24R BLM plasmids; cells were lysed under denaturing conditions and Ni-NTA beads were used to pull down His-tagged ubiquitin; blots were probed with the indicated antibodies. **h** E-resistant-BKO UM-UC-3 cells were cotransfected with His-MIB1, Flag-Ub, and HA-tagged BLM plasmids as indicated; HA-tagged BLM proteins were immunoprecipitated with anti-HA antibody and determined using western blot with anti-Flag, anti-His, and anti-HA antibodies. Graphs represent three replicates per condition (n = 3). ****p <* 0.001 represents a significant difference between two groups.





**Figure. S7. Upregulation of AARS1 catalyzes BLM-K24 lactylation and regulates BLM protein functions in EPI-resistant tumor. a** Western blot analyses detecting the expression of TIP60, P300, CBP, AARS1 and AARS2 using whole-cell extracts of E-resistant UM-UC-3 cells treated with different siRNAs. **b** Representative immunofluorescence images show BLM-K24la (red), AARS1 (green) and merged with DAPI (blue) of nuclei in NR-PT, R-PT, and R-RT bladder tumor tissues; scale bar: 50 μm. **c, d** AARS1 and β-actin levels were determined by western blot in NR-PT, R-PT, and R-RT bladder tumor samples from patients with non-resistant NMIBC and resistant NMIBC. **e** Kaplan-Meier survival curves of RFS of patients with NMIBC based on the mean fluorescence intensity of AARS1 in primary tumor tissues; the median value was employed as the cutoff value; statistical significance in RFS was determined using the Kaplan–Meier method and *P*-value was calculated by log-rank test. **f** Receiver operating characteristic (ROC) analysis for recurrence prediction based on the mean fluorescence intensity of AARS1 in primary bladder tumor tissues; the red circle indicates cutoff value of 21.54. **g** AARS1 and β-actin levels were determined by western blot in E-resistant UM-UC-3 cells before and 24 h after treatment with 20 mM sodium lactate. **h** E-resistant-BKO UM-UC-3 cells were cotransfected with Flag-AARS1 and HA-tagged WT or K24R BLM plasmids; Flag-tagged AARS1 protein was immunoprecipitated with anti-Flag antibody and analyzed by western blot with antibodies as indicated. **i, j** Molecular docking diagram showing the docking pose of WT and K24R BLM interacting with AARS1; the PIPER energies of the top 30 docking poses between BLM and AARS1 were compared using unpaired two-tailed Student’s t test. **k** Western blot analyses detecting the expression of AARS1 in E-resistant-BKO UM-UC-3 cells with stable overexpression or knockdown of AARS1. **l** E-resistant-BKO UM-UC-3 cells were cotransfected with HA-BLM and Flag-tagged WT AARS1 or 5A mutation (M46A, R77A, N216A, D239A and G241A) plasmids; HA-BLM protein was immunoprecipitated with anti-HA antibody and analyzed by western blot with antibodies as indicated. **m** E-resistant-BKO cells stably expressing AARS1 or AARS1 shRNA were cotransfected with HA-WT BLM or HA-K24R BLM plasmids; cells were used for immunoprecipitation using anti-HA antibody or IgG control; blots were probed with antibodies as indicated. **n** E-resistant-BKO cells stably expressing AARS1 or AARS1 shRNA were cotransfected with Flag-Ub, HA-WT BLM and (or) HA-K24R BLM plasmids; HA-tagged BLM protein was immunoprecipitated with anti-HA antibody and analyzed by western blot with anti-HA and anti-Flag antibodies. Graphs represent three replicates per condition (n = 3). ****p <* 0.001 represents a significant difference between two groups.

**

Figure. S8 Combination treatment with irinotecan enhances sensitivity to EPI in EPI-resistant tumors by inhibiting BLM-K24 lactylation. a, b** The interactions of irinotecan with K31R and K38R BLM proteins were tested by surface plasmon resonance (SPR) assays; BIAcore diagram showing BLM mutants bound to the irinotecan small-molecular drug with high affinities and slow dissociation kinetics. The 100 ns unrestricted molecular dynamics simulation assays showing the conformations (**c**) and RMSD (**d**) of the BLM–irinotecan complex during the simulation period. **e** Binding free energy between irinotecan and the K24 region of BLM protein was calculated using Molecular Mechanics/Generalized Born Surface Area (MMGBSA) method in gmx_MMPBSA software. E-resistant-BKO MDA-MB-231 cells (**f**) and E-resistant-BKO Huh7 cells (**g**) transfected with HA-BLM plasmid were treated with 4 μM irinotecan for 24 h or untreated; western blot analyses detecting BLM-K24 lactylation using K24-lactylation-specific antibody. **h** Cell viability assays detecting the viability of E-resistant-BKO MDA-MB-231 cells transfected with WT or K24R BLM plasmids in response to treatments with 0.2 μM EPI and (or) 4 μM irinotecan; one-way ANOVA followed by Tukey’s test. **i** Same experimental setup as in h but displaying cell proliferation by colony-formation assays; one-way ANOVA followed by Tukey’s test. **j** Same experimental setup as in h but determining the cell viability of E-resistant-BKO Huh7 cells by cell viability assays; one-way ANOVA followed by Tukey’s test. **k** Representative images and quantitative analyses show cell proliferation levels by colony-formation assays in E-resistant-BKO Huh7 cells treated with the same experimental setup as described in h; one-way ANOVA followed by Tukey’s test. **l** Western blot analysis of TOP Ⅰ expression in whole-cell extracts of E-resistant-BKO UM-UC-3 cells treated with siRNAs. **m** E-resistant-BKO UM-UC-3 cells transfected with HA-BLM were treated with siRNA-TOP Ⅰ and (or) 4 μM irinotecan for 24 h; cells were used for immunoprecipitation using anti-HA antibody or IgG control; blots were probed with antibodies as indicated. **n** Cell viability assays detecting the viability of E-resistant UM-UC-3 cells after treatments with siRNA-TOP Ⅰ, 0.2 μM EPI and (or) 4 μM irinotecan; unpaired two-tailed Student’s t test. **o** E-resistant-BKO UM-UC-3 cells cotransfected with His-Ub and HA-BLM were treated with 4 μM irinotecan for 24 h or untreated; cells were lysed under denaturing conditions and Ni-NTA beads were used to pull down His-tagged ubiquitin; blots were probed with the indicated antibodies. Graphs represent three replicates per condition (n = 3). ****p <* 0.001 represents a significant difference between two groups; ns represents no significant difference.


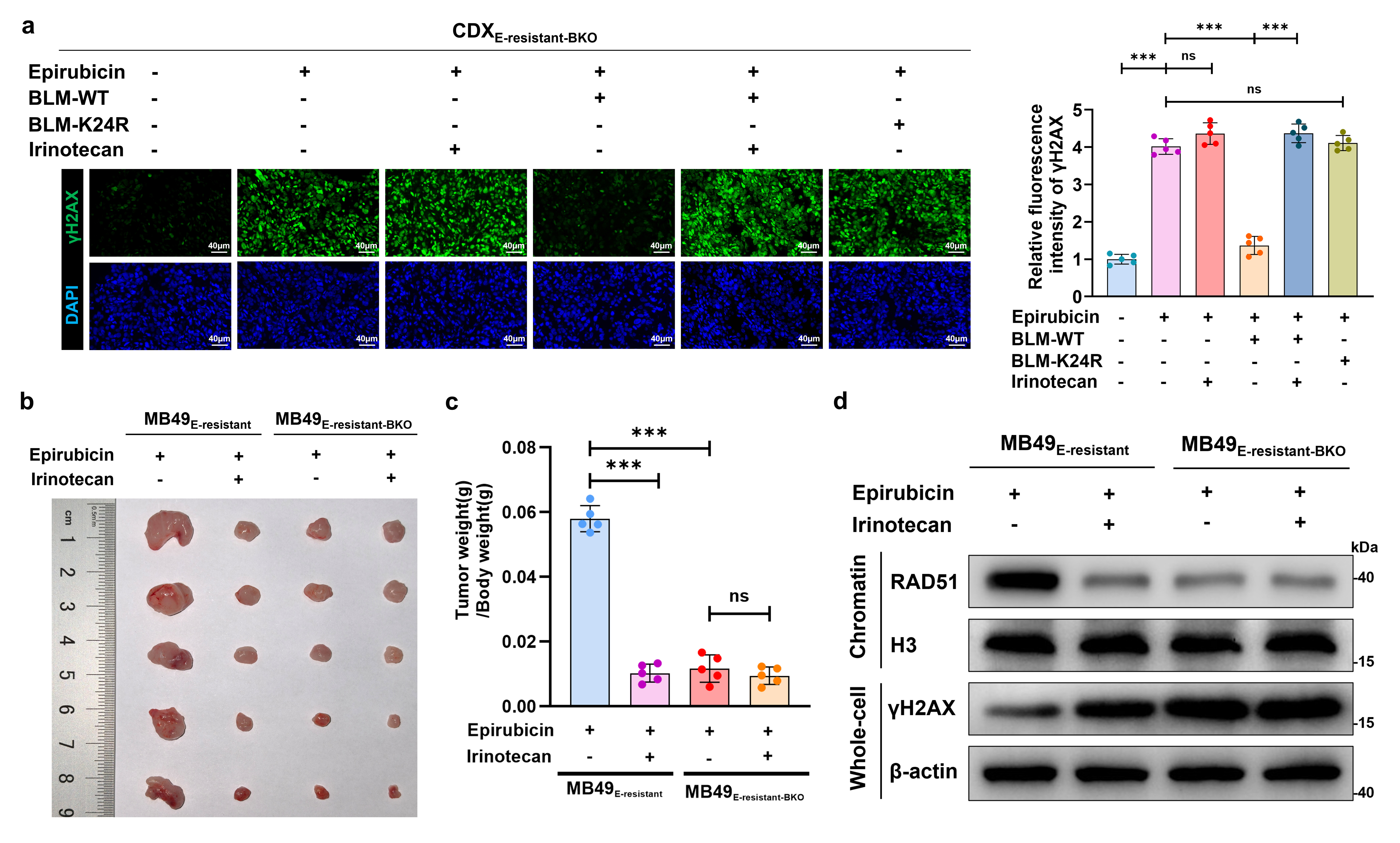
**Figure. S9** **Irinotecan treatment suppresses HR repair and reverses EPI resistance by regulating BLM.** **a** Representative immunofluorescence images (left) and quantitative analyses (right) show nuclear expression and localization of γH2AX (green) co-localized with DAPI (blue) in CDX models; scale bar: 40 μm. C57BL/6J mice were transplanted subcutaneously with EPI-resistant mouse bladder cancer cells (MB49_E-resistant_) and BLM-knockout MB49_E-resistant_ (MB49_E-resistant_-BKO) and treated with EPI (2 mg/kg) and (or) irinotecan (25 mg/kg) as indicated (n = 5); tumor images were shown in (**b**) and tumor weights were measured in (**c**). **d** Western blot analysis of γH2AX in whole-cell extracts and RAD51 in chromatin fractions from CDX models. ****p <* 0.001 represents a significant difference between two groups; ns represents no significant difference.


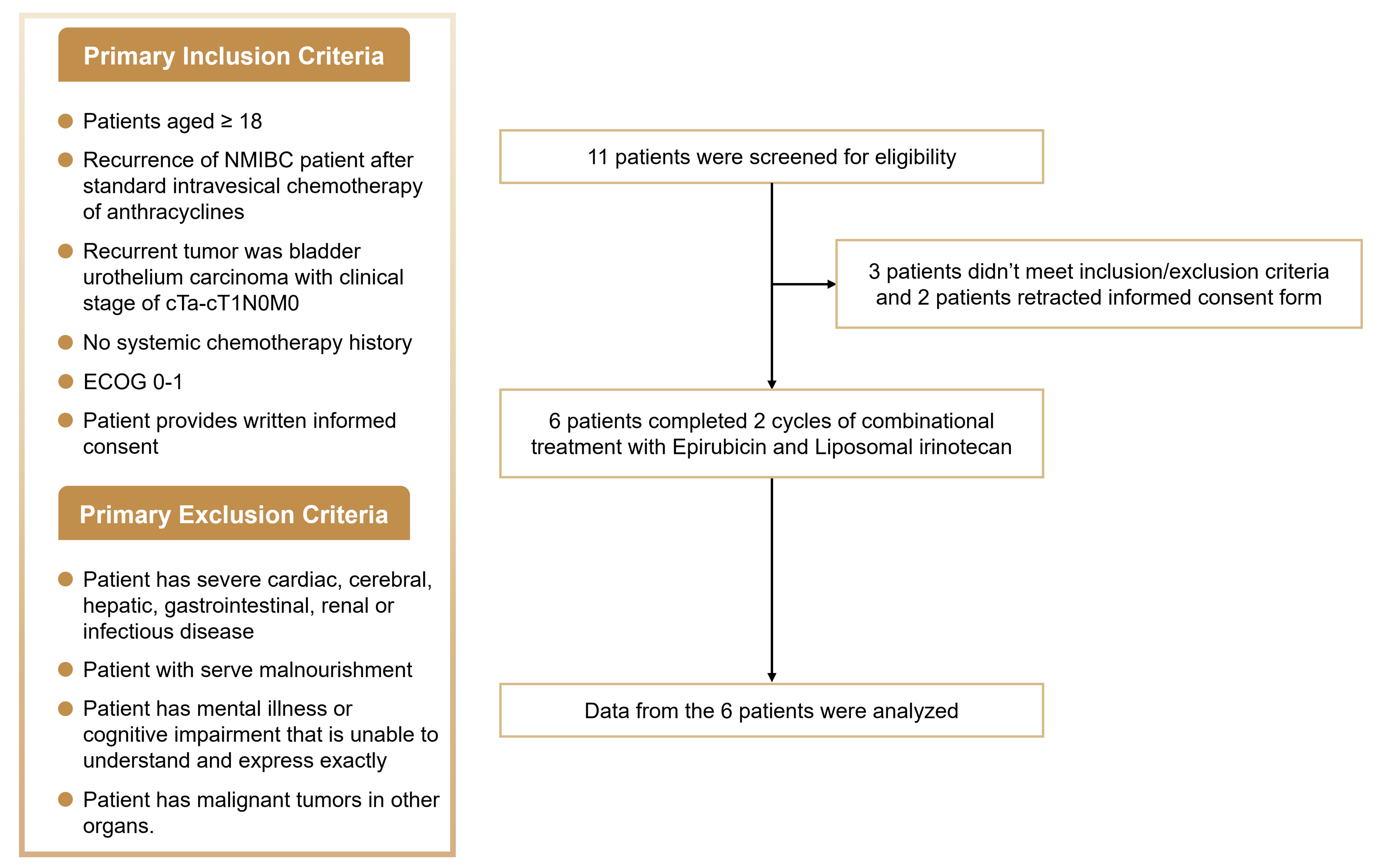


**Figure. S10 Trial design and participant enrollment.** Flow of participants enrolled in the study.

Table S1. The clinicopathologic factors of NR-PT, R-PT and R-RT patients

| **Parameter** | **NR-PT** | **R-PT** | ***p*-value** | **R-RT** | ***p*-value**  **(R-PT vs. R-RT)** |
| --- | --- | --- | --- | --- | --- |
| **Number** | 145 | 64 | — | 64 | — |
| **Gender** |  |  | 0.808 |  | 1.000 |
| Male | 111 | 48 |  | 48 |  |
| Female | 34 | 16 |  | 16 |  |
| **Age (year)** |  |  | 0.149 |  | 0.579 |
| < 65 | 70 | 24 |  | 21 |  |
| ≥ 65 | 75 | 40 |  | 43 |  |
| **Tumor size (MaxD cm)** |  |  | 0.078 |  | 0.215 |
| *<* 2 | 87 | 30 |  | 37 |  |
| ≥ 2 | 58 | 34 |  | 27 |  |
| **Grade** |  |  | **0.034** |  | **0.020** |
| High | 28 | 21 |  | 34 |  |
| Low | 117 | 43 |  | 30 |  |
| **T stage** |  |  | **0.018** |  | ***<* 0.001** |
| Ta | 104 | 35 |  | 17 |  |
| T1 | 41 | 29 |  | 32 |  |
| T2 | 0 | 0 |  | 15 |  |
| **Lymph node status** |  |  | — |  | — |
| Negative | 145 | 64 |  | 64 |  |
| Positive | 0 | 0 |  | 0 |  |
| **Distant metastasis site** |  |  | — |  | 0.496 |
| Negative | 145 | 64 |  | 62 |  |
| Positive | 0 | 0 |  | 2 |  |

Note: NR-RT: the primary tumor of non-recurrent patient, MaxD: maximum diameter, R-PT: the primary tumor of recurrent patient, R-RT: the recurrent tumor of recurrent patient. The bold number represents the *p*-value with significant difference.

**Table S2. The clinicopathologic information and clinical response of participants in this trial**

| **Patient ID** | **Sex** | **ECOG** | **Primary**  **chemotherapy** | **Surgical history** | **Recurrent**  **tumor**  **pathological type** | **Recurrent**  **tumor grade** | **Recurrent**  **tumor TNM stage** | **Recurrent**  **tumor number** | **Combinational**  **treatment** | **Post-treatment outcome** |
| --- | --- | --- | --- | --- | --- | --- | --- | --- | --- | --- |
| A | Male | 0 | Epirubicin | Yes,  TURBT | BUC | Low | T1N0M0 | Single | Epirubicin (50 mg)  Liposomal irinotecan  (37.6-56.5 mg/m^2^) | No recurrence |
| B | Male | 1 | Epirubicin | Yes,  TURBT | BUC | Low | T1N0M0 | Single | Epirubicin (50 mg)  Liposomal irinotecan  (37.6-56.5 mg/m^2^) | No recurrence |
| C | Male | 0 | Pirarubicin | Yes,  TURBT | BUC | Low | TaN0M0 | Single | Epirubicin (50 mg)  Liposomal irinotecan  ((37.6-56.5 mg/m^2^) | No recurrence |
| D | Male | 0 | Epirubicin | Yes,  TURBT | BUC | Low | T1N0M0 | Multiple | Epirubicin (50 mg)  Liposomal irinotecan  (56.5 mg/m^2^) | No recurrence |
| E | Male | 0 | Epirubicin | Yes,  TURBT | BUC | Low | TaN0M0 | Single | Epirubicin (50 mg)  Liposomal irinotecan  (56.5 mg/m^2^) | No recurrence |
| F | Male | 1 | Epirubicin | Yes,  TURBT | BUC | Low | T1N0M0 | Single | Epirubicin (50 mg)  Liposomal irinotecan  (56.5 mg/m^2^) | No recurrence |

Note: BUC: bladder urothelial carcinoma, TURBT: transurethral resection of bladder tumor.

**Table S3. Summary of DLT and AEs for all participants completing two cycles of combinational treatment**

| **AE (According to CTCAE 5.0)** | **Any Grade, No. (%)** | **Grade 1, No. (%)** | **Grade 2, No. (%)** | **Grade 3, No. (%)** | **Grade 4, No. (%)** |
| --- | --- | --- | --- | --- | --- |
| **Any** | **4 (67)** | **4 (67)** | **1 (17)** | **0** | **0** |
| **DLT** | **0** | **0** | **0** | **0** | **0** |
| Nausea | 4 (67) | 3 (50) | 1 (17) | 0 | 0 |
| Vomiting | 1 (17) | 1 (17) | 0 | 0 | 0 |
| Fatigue | 2 (33) | 2 (33) | 0 | 0 | 0 |
| Diarrhea | 2 (33) | 2 (33) | 0 | 0 | 0 |
| Abdominal pain | 0 | 0 | 0 | 0 | 0 |
| Decreased appetite | 3 (50) | 2 (33) | 1 (17) | 0 | 0 |

Note: AEs: adverse events, DLT: dose-limiting toxicity.

**Table S4. Summary of DLT and physiological parameters for participants completing two cycles of combinational treatment**

| **AE (According**  **to CTCAE 5.0)** | **Any Grade, No. (%)** | **Grade 1, No. (%)** | **Grade 2, No. (%)** | **Grade 3, No. (%)** | **Grade 4, No. (%)** |
| --- | --- | --- | --- | --- | --- |
| **Any** | **5 (83)** | **5 (83)** | **0** | **0** | **0** |
| **DLT** | **0** | **0** | **0** | **0** | **0** |
| RBC counts decreased | 1 (17) | 1 (17) | 0 | 0 | 0 |
| Anemia | 1 (17) | 1 (17) | 0 | 0 | 0 |
| WBC counts decreased | 0 | 0 | 0 | 0 | 0 |
| Neutropenia | 0 | 0 | 0 | 0 | 0 |
| Thrombocytopenia | 0 | 0 | 0 | 0 | 0 |
| Hypokalemia | 0 | 0 | 0 | 0 | 0 |
| Hypocalcemia | 0 | 0 | 0 | 0 | 0 |
| Hypoalbuminemia | 0 | 0 | 0 | 0 | 0 |
| ALT increased | 1 (17) | 1 (17) | 0 | 0 | 0 |
| AST increased | 1 (17) | 1 (17) | 0 | 0 | 0 |
| GGT increased | 1 (17) | 1 (17) | 0 | 0 | 0 |
| ALP increased | 1 (17) | 1 (17) | 0 | 0 | 0 |
| Total bilirubin increased | 2 (33) | 2 (33) | 0 | 0 | 0 |
| Urca decreased | 0 | 0 | 0 | 0 | 0 |
| Creatinine increased | 0 | 0 | 0 | 0 | 0 |
| Myoglobin increased | 0 | 0 | 0 | 0 | 0 |
| CKMB increased | 0 | 0 | 0 | 0 | 0 |
| Troponin T increased | 0 | 0 | 0 | 0 | 0 |

Note: ALT: alanine aminotransferase, ALP: alkaline phosphatase, AST: aspartate aminotransferase, CKMB: creatine kinase isoenzymes B, DLT: dose-limiting toxicity, GGT: gamma glutamyl transferase, RBC: red blood cell, WBC: white blood cell.

**Table S5. The sgRNA, siRNA, shRNA, primer and probe sequences used in this study**

| **sgRNA** | | **Target Sequence** |
| --- | --- | --- |
| BLM-sgRNA-1 | | GGAACGAACTGCTTCAGCAG |
| BLM-sgRNA-2 | | GTAGTCAGTAAACAGTCCCC |
| BLM-sgRNA-3 | | TCACTTGATGGCCCTATGGA |
| **shRNA** | | **Target Sequence** |
| AARS1-shRNA-1 | | CCCATTGAAAGACTTTATGTT |
| AARS1-shRNA-2 | | GGTGGATGACAGCAGTGAAGA |
| AARS1-shRNA-3 | | GGACATCATTAATGAAGAAGA |
| **siRNA** | | **Sequence** |
| TOP Ⅰ-siRNA-1 | | GCUUAACCCUAGUUCACGA |
| TOP Ⅰ-siRNA-2 | | GAUUGCUAACUUCAAGAUA |
| TOP Ⅰ-siRNA-3 | | GAUAGACUCAAUACUGGUA |
| TIP60-siRNA-1 | | ACGGAAGGTGGAGGTGGTTAA |
| TIP60-siRNA-2 | | GTACGGCCGTAGTCTCAAGAA |
| P300-siRNA-1 | | TGACACAGGCAGGCTTGAC |
| P300-siRNA-2 | | TCTACTCTCAAATCCGGCG |
| CBP-siRNA-1 | | TAGTAACTCTGGCCATAGC |
| CBP-siRNA-2 | | CGCCGACAACTAAGGAGTT |
| AARS1-siRNA-1 | | GTTTGGCATTCCCATTGAA |
| AARS1-siRNA-2 | | GCAGTGAGATCCACTACGA |
| AARS2-siRNA-1 | | GGAACCTGGTCTTCATGCA |
| AARS2-siRNA-2 | | CGAGACCTTTCCCATCATA |
| **Primer and Probe** | | **Sequence** |
| DSB-335 bp | Primer-F | GAATCGGATGTATGCGACTGATC |
|  | Primer-R | TTCCAAAGTTATTCCAACCCGAT |
|  | Probe | 6FAM-CACAGCTTGCCCATCCTTGCAAACC-TAMRA |
| DSB-1618 bp | Primer-F | TGAGGAGGTGACATTAGAACTCAGA |
|  | Primer-R | AGGACTCACTTACACGGCCTTT |
|  | Probe | 6FAM-TTGCAAGGCTGCTTCCTTACCATTCAA-TAMRA |
| DSB-3500 bp | Primer-F | TCCTAGCCAGATAATAATAGCTATACAAACA |
|  | Primer-R | TGAATAGACAGACAACAGATAAATGAGACA |
|  | Probe | 6FAM-ACCCTGATCAGCCTTTCCATGGGTTAAG-TAMRA |
| TOP Ⅰ | Primer-F | TCCGGCATGATAACAAGGTTACT |
|  | Primer-R | TCTCGATACTGGTTCCGGATCTT |
| β-actin | Primer-F | CCTTCCTGGGCATGGAGTC |
|  | Primer-R | TGATCTTCATTGTGCTGGGTG |
| 5’-labeled dsDNA | *5’-3’ | GACGTCATAGACGATTACATTGCTAGGACATGCTGTCTAGAGACTATCGC |
|  | 3’-5’ | CTGCAGTATCTGCTAATGTAACGATCCTGTACGACAGATCTCTGATAGCG |
| 3’-labeled tailed DNA | 5’-3’ | TTGCTAGGACATGCTGTCTAGAGACTATCGC |
|  | *3’-5’ | CTGCAGTATCTGCTAATGTAACGATCCTGTACGACAGATCTCTGATAGCG |

**Table S6. Antibodies, chemicals, critical commercial assays and experimental models used in this study**

| **REAGENT or RESOURCE** | **SOURCE** | **IDENTIFIER** |
| --- | --- | --- |
| **Antibodies** | | |
| Rabbit anti-L-lactyl lysine | PTM BIO | Cat# PTM-1401RM |
| Rabbit anti-BLM-K24la | PTM BIO | Cat#CO1220 |
| Mouse anti-β-actin | Proteintech | Cat# 66009-1-Ig; RRID: AB_2687938 |
| Mouse anti-RAD51 | Proteintech | Cat# 67024-1-Ig; RRID: AB_2882339 |
| Rabbit anti-γH2AX | Abcam | Cat# ab81299; RRID: AB_1640564 |
| Rabbit anti-BLM | Abcam | Cat# ab2179; RRID: AB_2290411 |
| Rabbit anti-AARS1 | Abclonal | Cat#A15017; RRID: AB_2761897 |
| Rabbit anti-H3 | Abmart | Cat# P30266; RRID: AB_2936509 |
| Mouse anti-HA | Abmart | Cat# M20003; RRID: AB_2864345 |
| Rabbit anti-HA | Cell Signaling | Cat# 3724; RRID: AB_1549585 |
| Rabbit anti-Flag | Cell Signaling | Cat# 14793; RRID: AB_2572291 |
| Rabbit anti-His | Cell Signaling | Cat# 12698; RRID: AB_2744546 |
| Rabbit anti-DNA2 | Thermo Fisher | Cat# PA5-77943, RRID: AB_2735727 |
| Rabbit anti-TOPⅡA | Abcam | Cat# ab52934; RRID: AB_883143 |
| Rabbit anti-RPA | Abcam | Cat# ab79398; RRID: AB_1603759 |
| Rabbit anti-Ki67 | Abcam | Cat# ab15580; RRID: AB_805388 |
| HRP-conjugated Veriblot for IP secondary antibody | Abcam | Cat# ab131368; RRID: AB_2895114 |
| HRP-conjugated Veriblot for IP secondary antibody | Abcam | Cat# ab131366; RRID: AB_2892718 |
| HRP-conjugated anti-mouse IgG | Proteintech | Cat# SA00001-1; RRID: AB_2722565 |
| HRP-conjugated anti-rabbit IgG | Proteintech | Cat# SA00001-2; RRID: AB_2722564 |
| Alexa Fluor 488 anti-mouse IgG | Cell Signaling | Cat# 4408; RRID: AB_10694704 |
| Alexa Fluor 488 anti-rabbit IgG | Cell Signaling | Cat# 4412; RRID: AB_1904025 |
| Alexa Fluor 647 anti-mouse IgG | Cell Signaling | Cat# 4410; RRID: AB_1904023 |
| Alexa Fluor 647 anti-rabbit IgG | Cell Signaling | Cat# 4414; RRID: AB_10693544 |
| **Bacterial and virus strains** | | |
| TOP10 | Tsingke | Cat# TSC-C12 |
| DH5α | Tsingke | Cat# TSC-C14 |
| STBL3 | Thermo Fisher | Cat# C737303 |
| **Chemicals, peptides, and recombinant proteins** | | |
| Sodium lactate | Sigma-Aldrich | Cat# L7022 |
| Sodium oxamate | MedChemExpress | Cat# HY-W013032A |
| Epirubicin | MedChemExpress | Cat# HY-13624A |
| Irinotecan | MedChemExpress | Cat# HY-16562 |
| PEI | Sigma-Aldrich | Cat# 919012 |
| Polybrene | Sigma-Aldrich | Cat# TR-1003 |
| Puromycin | Sigma-Aldrich | Cat# 540222 |
| 4-Hydroxytamoxifen (4-OHT) | Sigma–Aldrich | Cat# H6278 |
| MG132 | Sigma–Aldrich | Cat# M7449 |
| Cycloheximide | Sigma–Aldrich | Cat# 5087390001 |
| G418 | Beyotime | Cat# ST081-1ml |
| BsrGI | New England Biolabs | Cat# R3575S |
| **Critical commercial assays** | | |
| Dulbecco’s modified Eagle’s medium | Gibco | Cat# 11965092 |
| RPMI 1640 | Gibco | Cat# 11875093 |
| Penicillin/Streptomycin | Beyotime | Cat# C0222 |
| Foetal bovine serum | Pricella | Cat# 164210-50 |
| Cell Culture Contamination Detection Kit | Invitrogen | Cat# C7028 |
| BCA protein assay Kit | Thermo Fisher | Cat# 23227 |
| DAB kit | Thermo Fisher | Cat# 34002 |
| 4% Paraformaldehyde | Biosharp | Cat# BL539A |
| CCK-8 Kit | DOJINDO | Cat# CK04 |
| RIPA | Beyotime | Cat# P0013B |
| PMSF | Beyotime | Cat# ST506 |
| SDS-PAGE Protein loading buffer (5X) | Beyotime | Cat# P0015 |
| SDS-PAGE Protein loading buffer (1X) | Beyotime | Cat# P0015A |
| NP-40 buffer | Beyotime | Cat# P0013F |
| Protease inhibitor cocktail | Selleck | Cat# B14001 |
| Plasmid Extraction Kit | OMEGA | Cat# D6945 |
| protein A/G magnetic beads | MedChemExpress | Cat# HY-K0202 |
| Nuclear and Cytoplasmic Protein Extraction Kit | Thermo Fisher | Cat# 78835 |
| Subcellular Protein Fractionation Kit | Thermo Fisher | Cat# 78840 |
| Subcellular Protein Fractionation Kit | Thermo Fisher | Cat# 87790 |
| Comet Assay Kit | KeyGen BioTECH | Cat# KGA1302-100 |
| DNAzol reagent kit | Invitrogen | Cat# 10503027 |
| 2×Taqman Universal PCR Master Mix | Applied Biosystems | Cat# 4304437 |
| PVDF membranes | Millipore | Cat# ISEQ00010 |
| Lactate Content Assay Kit | Solarbio | Cat# BC2235 |
| ALT Assay Kit | Solarbio | Cat# BC1555 |
| AST Assay Kit | Solarbio | Cat# BC1565 |
| RNAiso Plus | Takara | Cat# 9109 |
| ABScript Neo RT Master Mix for qPCR with gDNA Remove | ABclonal | Cat# RK20433 |
| 2X Universal SYBR Green Fast qPCR Mix | ABclonal | Cat# RK21203 |
| **Deposited data** | | |
| Raw and Analyzed data (that is not included in Data S1) | Upon Request | N/A |
| Raw data/gels data used to generate figures | Unedited blot and gel images | N/A |
| Lactylome and proteomics datasets | Proteomics Identification Database | <http://www.ebi.ac.uk/pride>  Identifier: PXD062720 |
| Metabolomics datasets | Figshare | <https://doi.org/10.6084/m9.figshare.29279096> |
| **Experimental models: Cell lines** | | |
| Human: U2OS | ATCC | Cat# HTB-96 |
| Human: HEK-293T | ATCC | Cat# CRL-3216 |
| Human: UM-UC-3 | ATCC | Cat# CRL-1749 |
| Human: MDA-MB-231 | ATCC | Cat# HTB-26 |
| Human: Huh-7 | National Collection of Authenticated Cell Cultures | Cat# SCSP-526 |
| **Experimental models: Organisms/strains** | | |
| Mouse: M-NSG | MODEL ORGANISMS | N/A |
| Mouse: BALB/c Nude | MODEL ORGANISMS | N/A |
| Mouse: C57BL/6J | MODEL ORGANISMS | N/A |
| **Software and algorithms** | | |
| GraphPad Prism 9.5.1 | GraphPad Software | N/A |
| LAS X Life Science Microscope Software | Leica | N/A |
| Fiji-Image J | NIH | N/A |

Data S1. (separate file)

The original western-blot images.

Data S2. (separate file)

The raw metabolomics data.

Study Protocol
