# Supplementary material for: Irinotecan alleviates chemoresistance to anthracyclines through the inhibition of AARS1-mediated BLM lactylation and homologous recombination repair
Source: Signal Transduct Target Ther. 2025 Jul 10;10:214. doi: 10.1038/s41392-025-02302-y (PMC12241633; doi:10.1038/s41392-025-02302-y)

The original western-blot images

Figure1K

RAD51

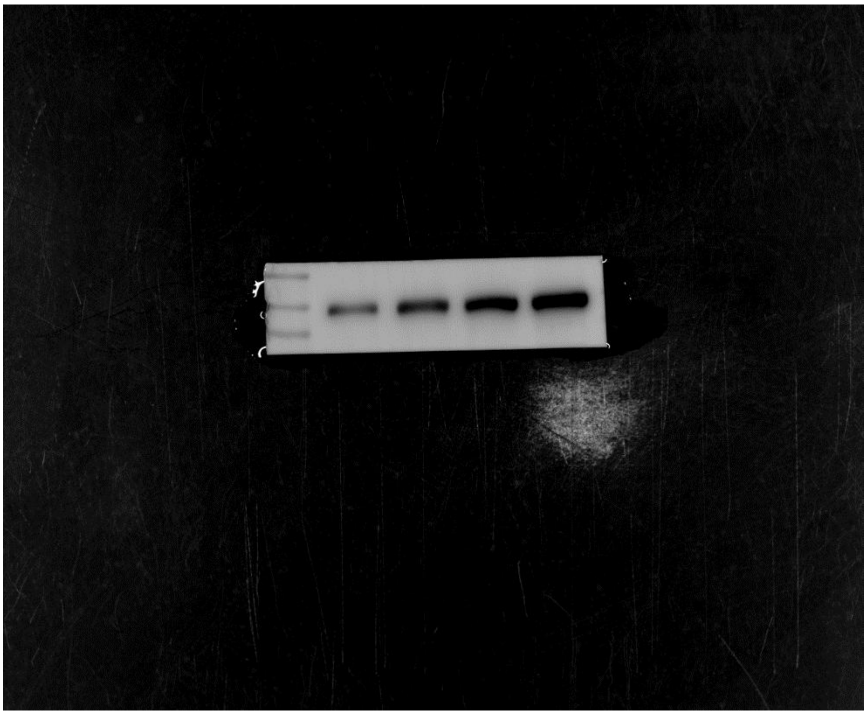

H3

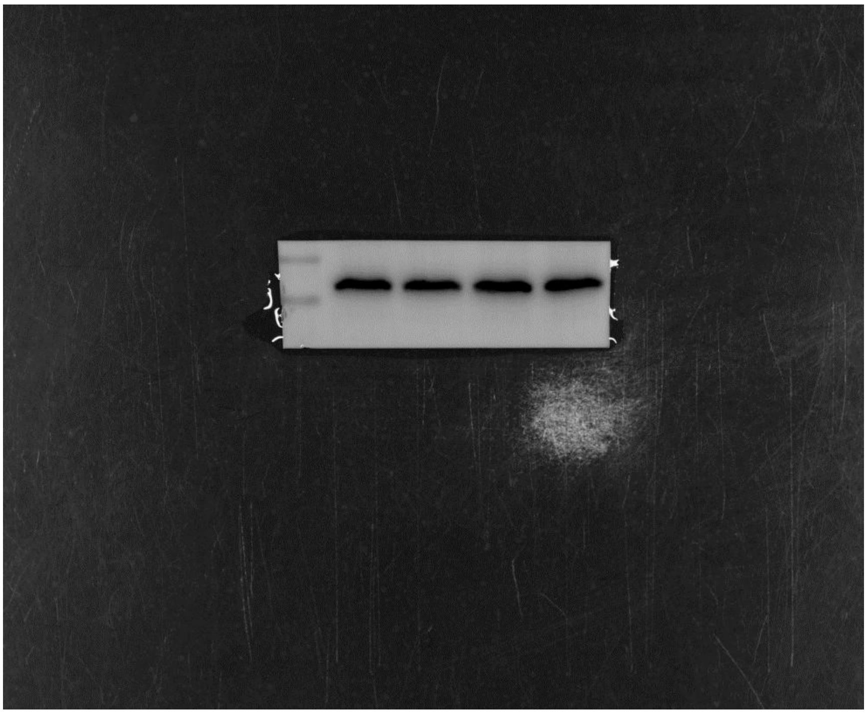

γH2AX

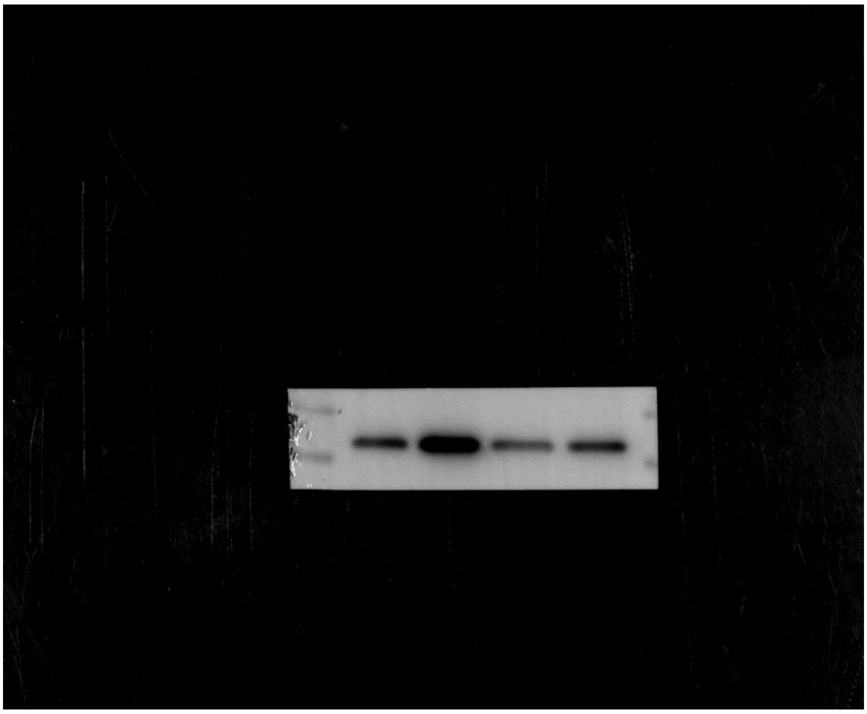

β-actin

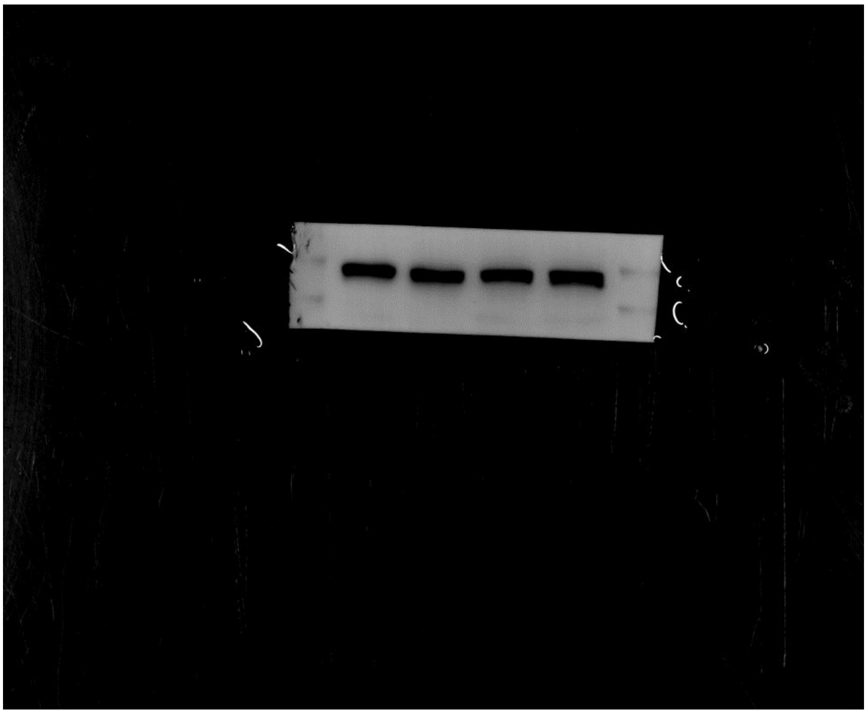

Figure1M

LDHA

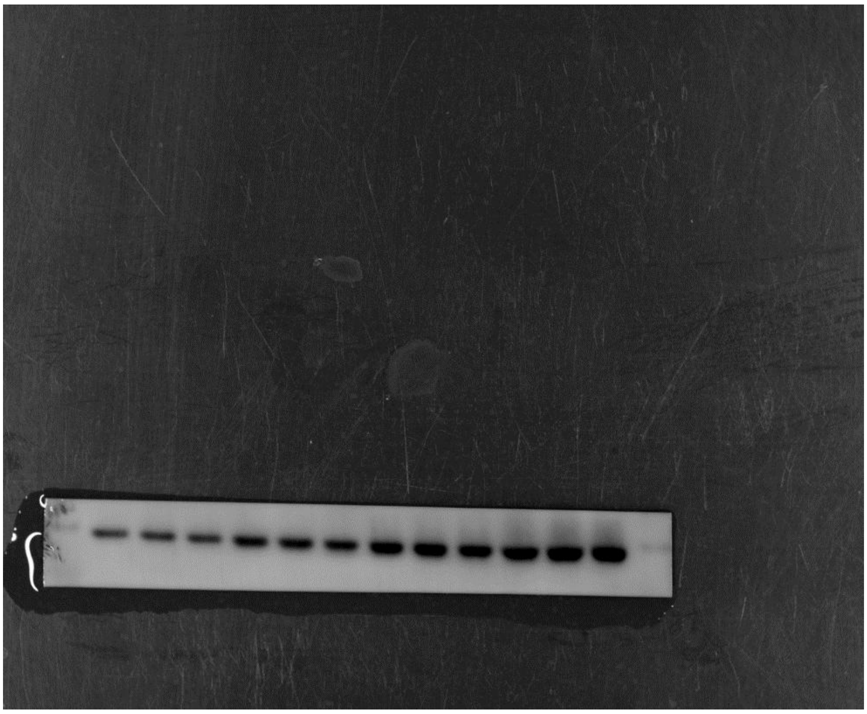

Pan-Kla

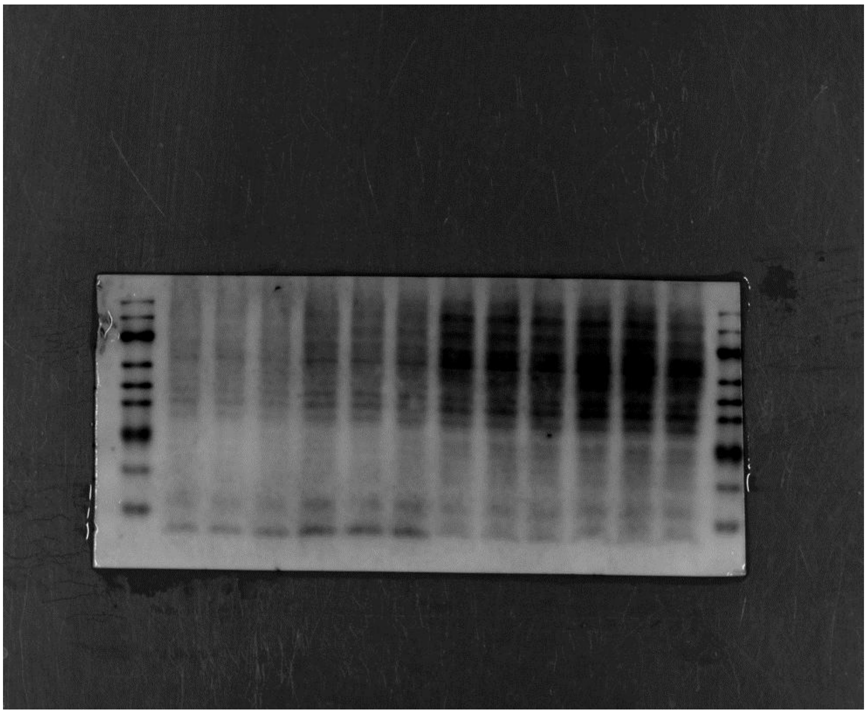

β-actin

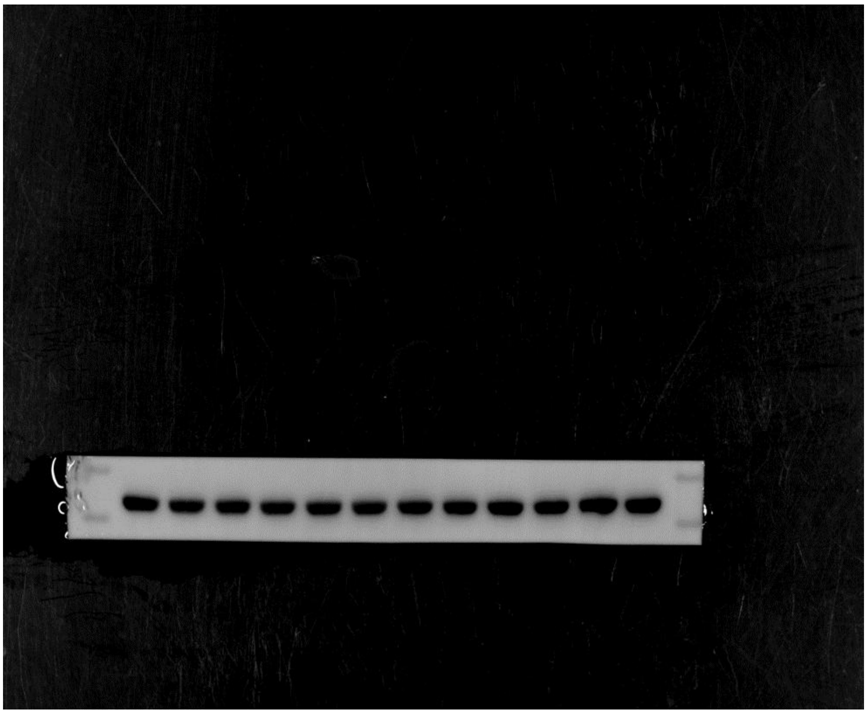

Figure1N

RAD51

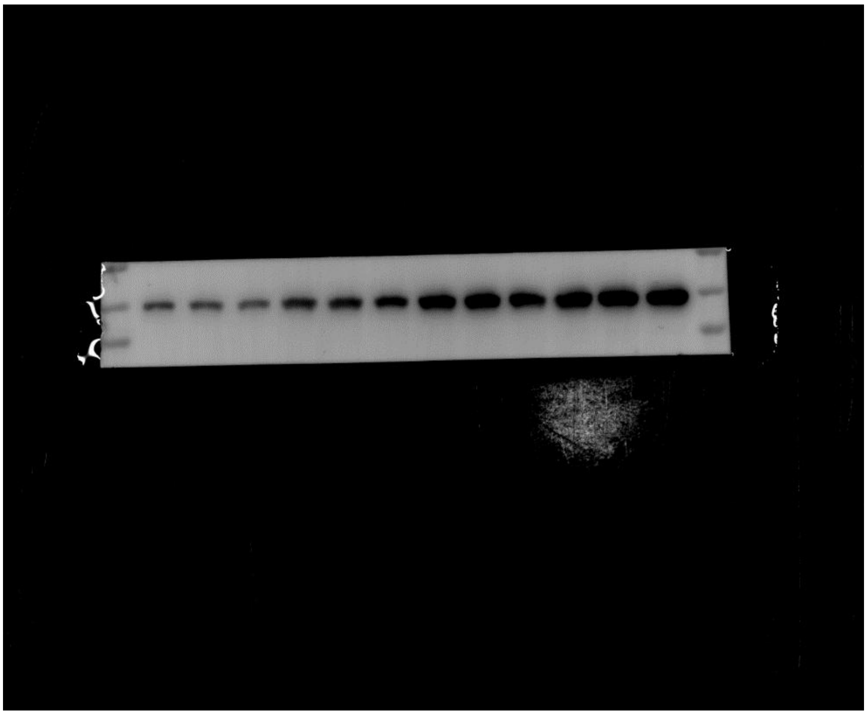

H3

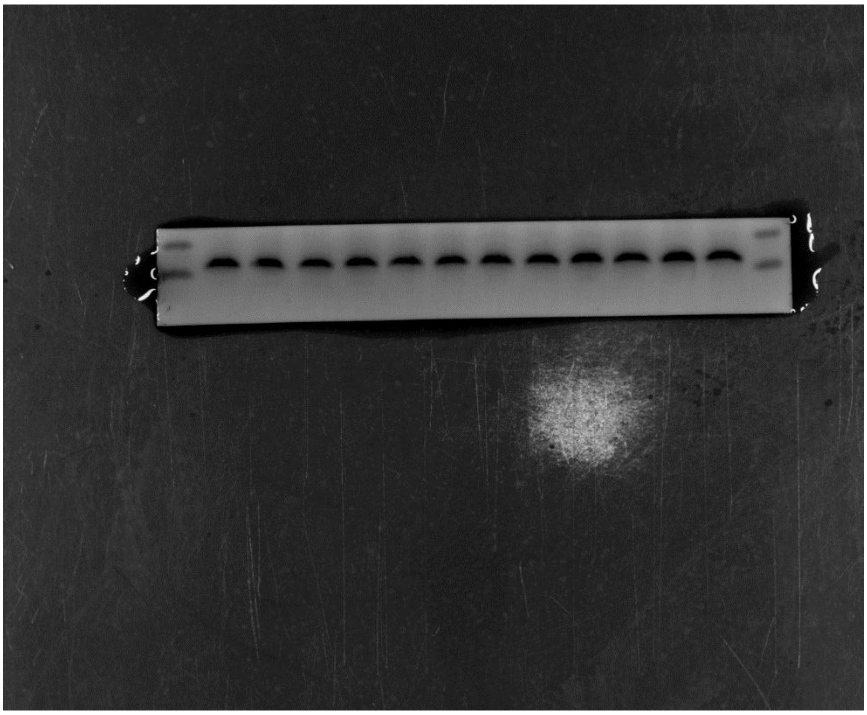

γH2AX

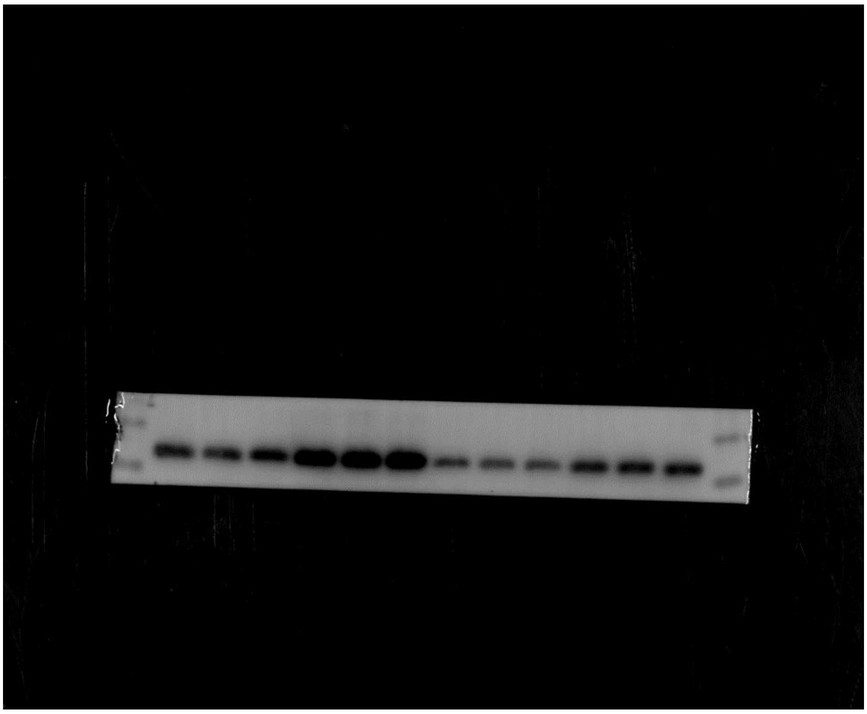

β-actin

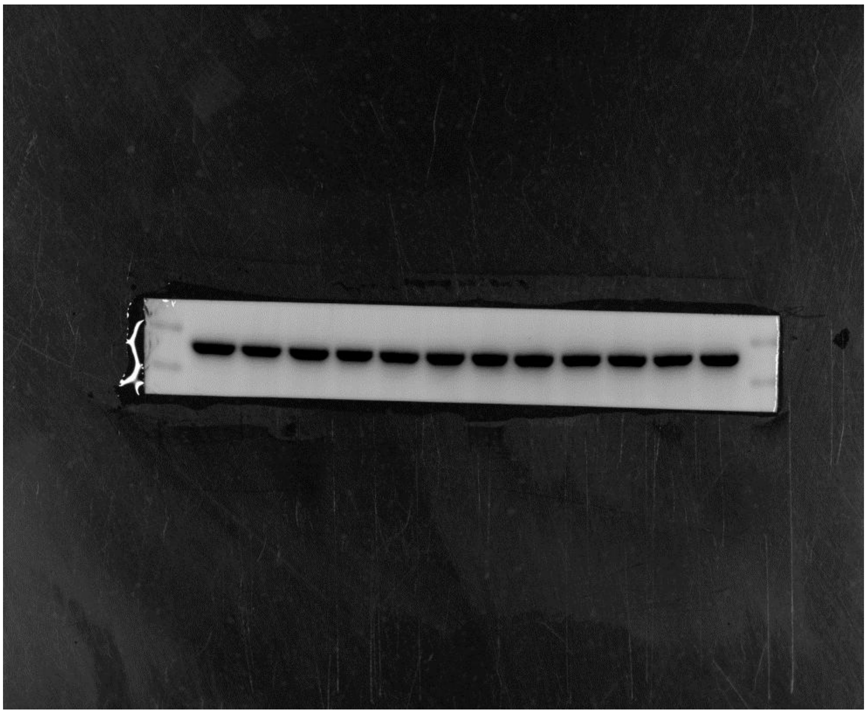

Figure2A

Pan-Kla

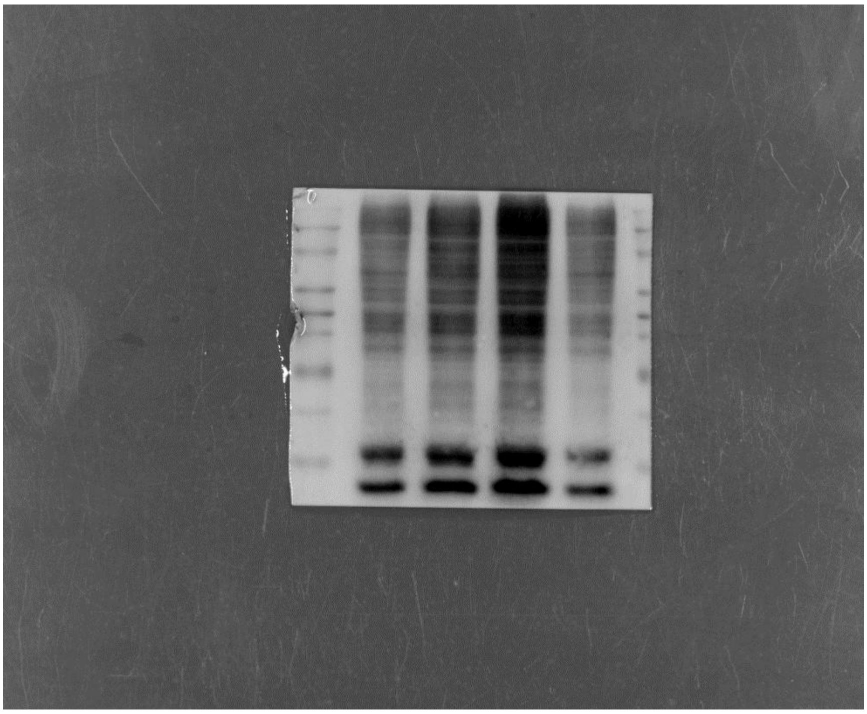

β-actin

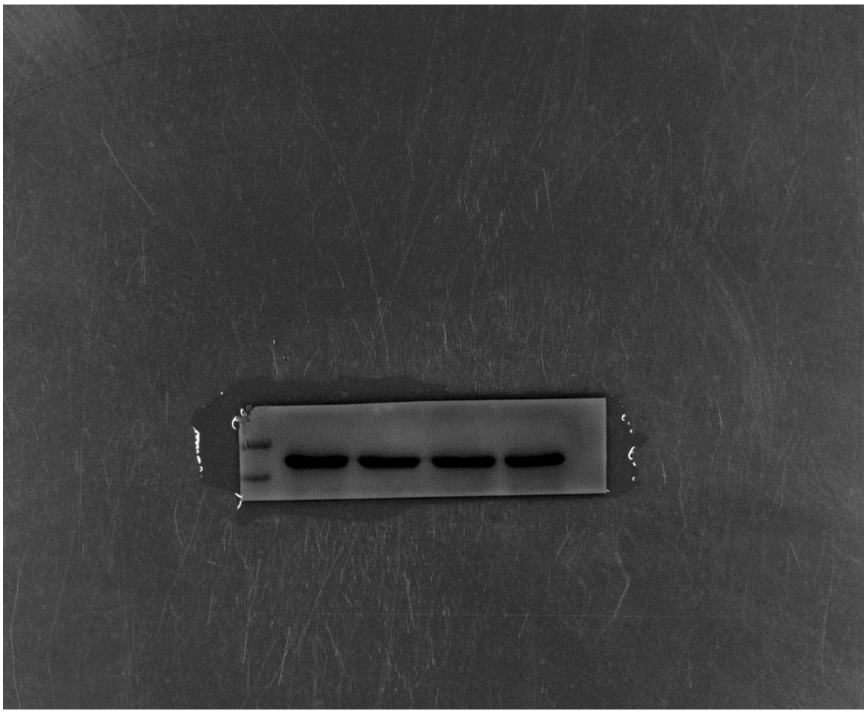

Figure2E

RAD51

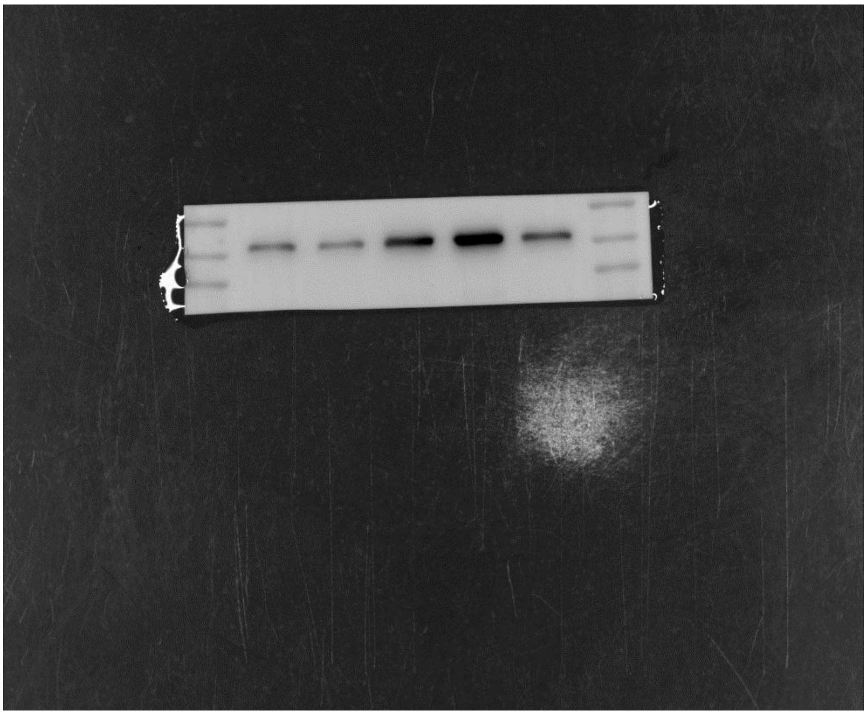

H3

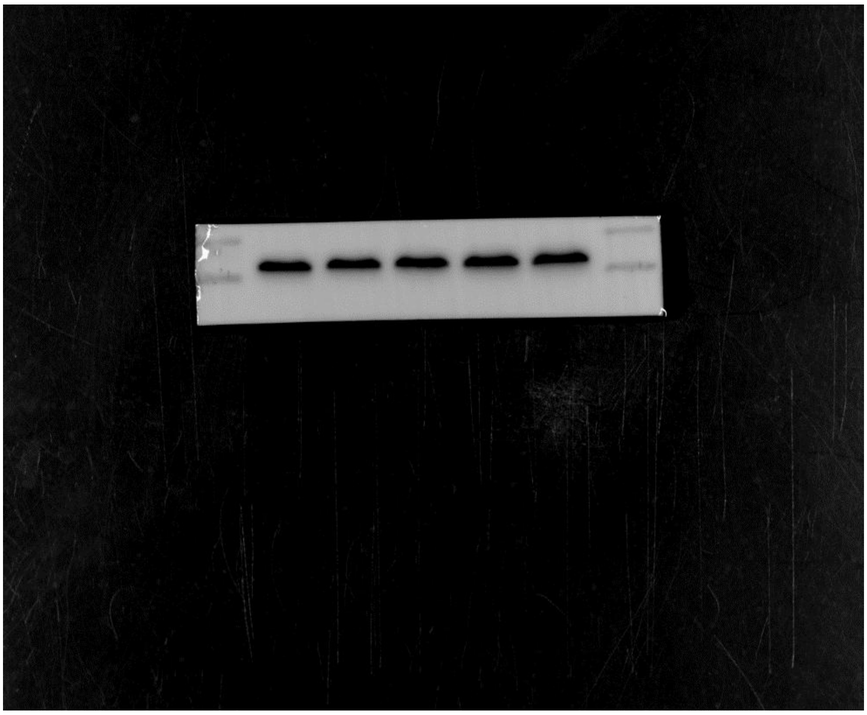

γH2AX

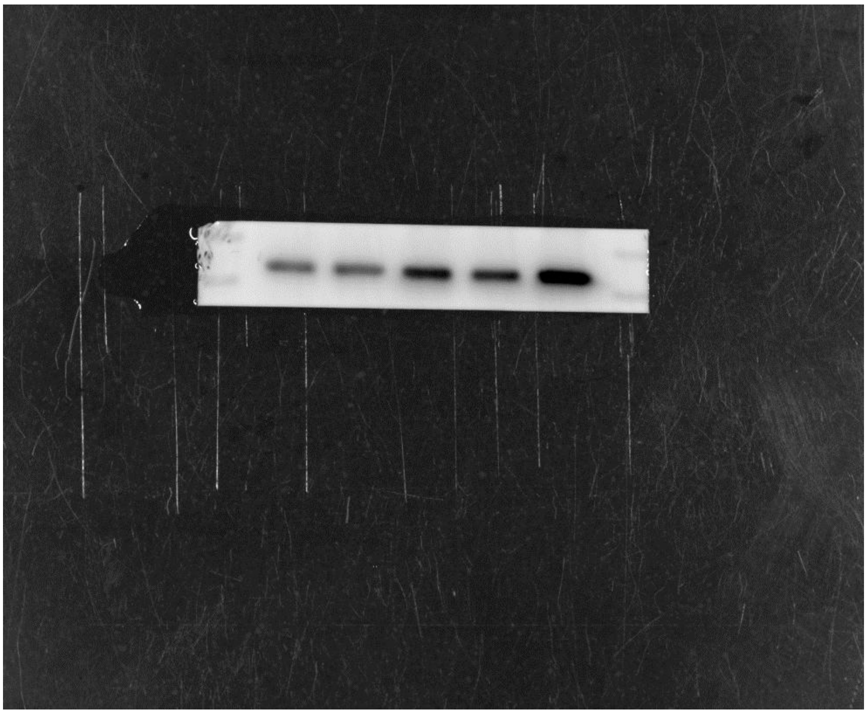

β-actin

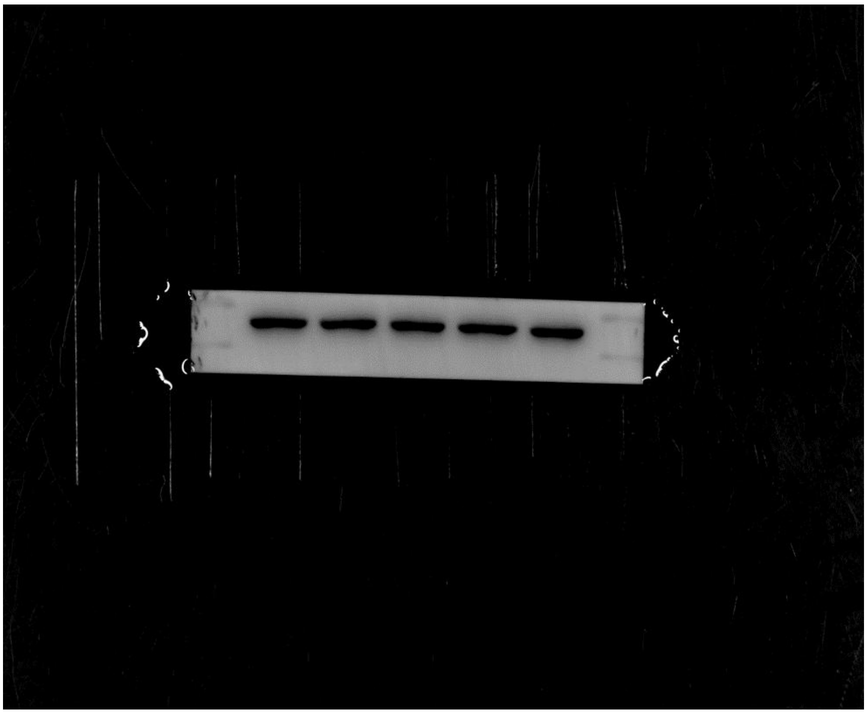

Figure2H

Pan-Kla

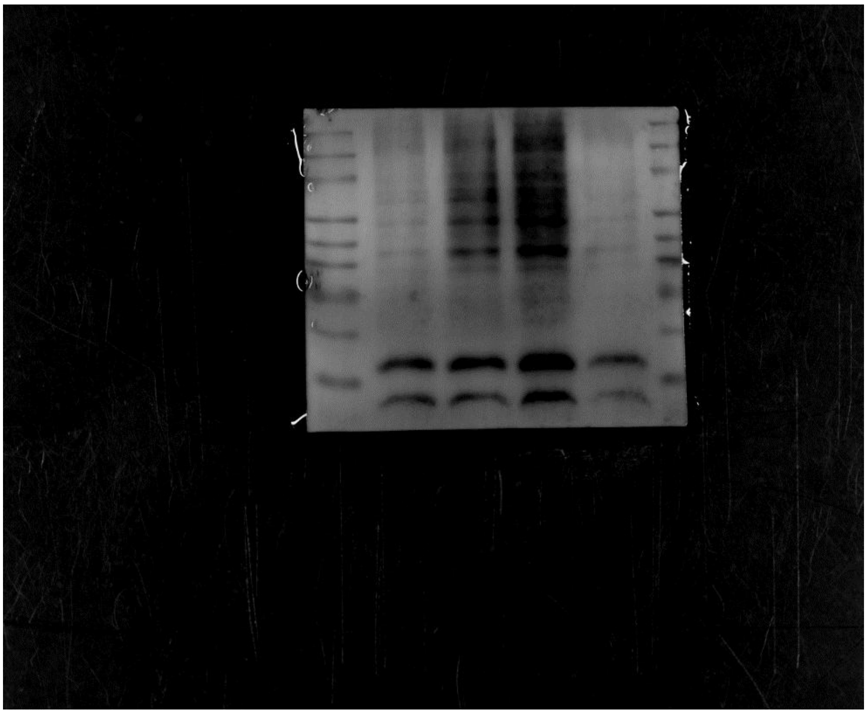

γH2AX

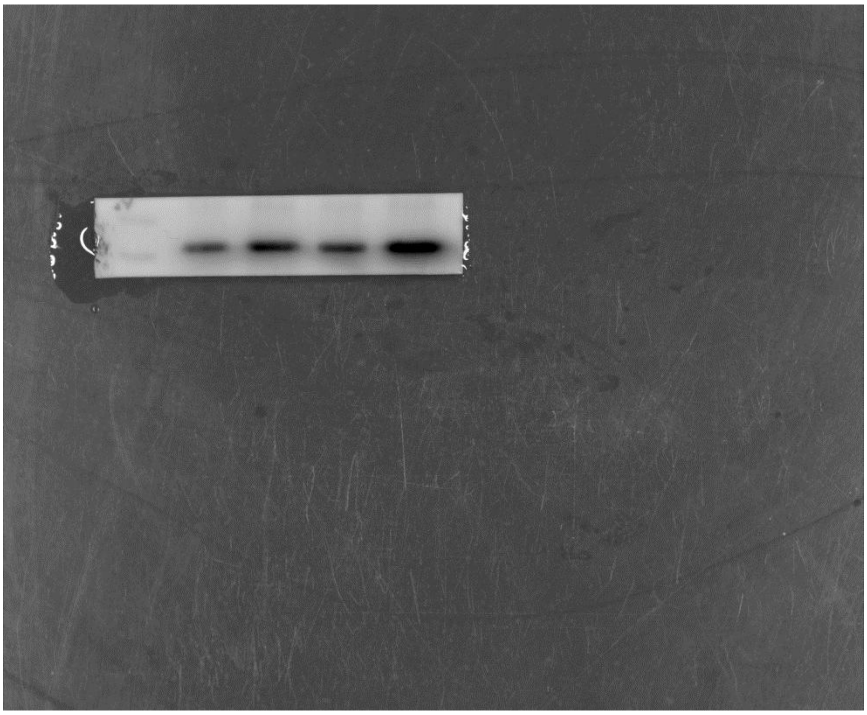

β-actin

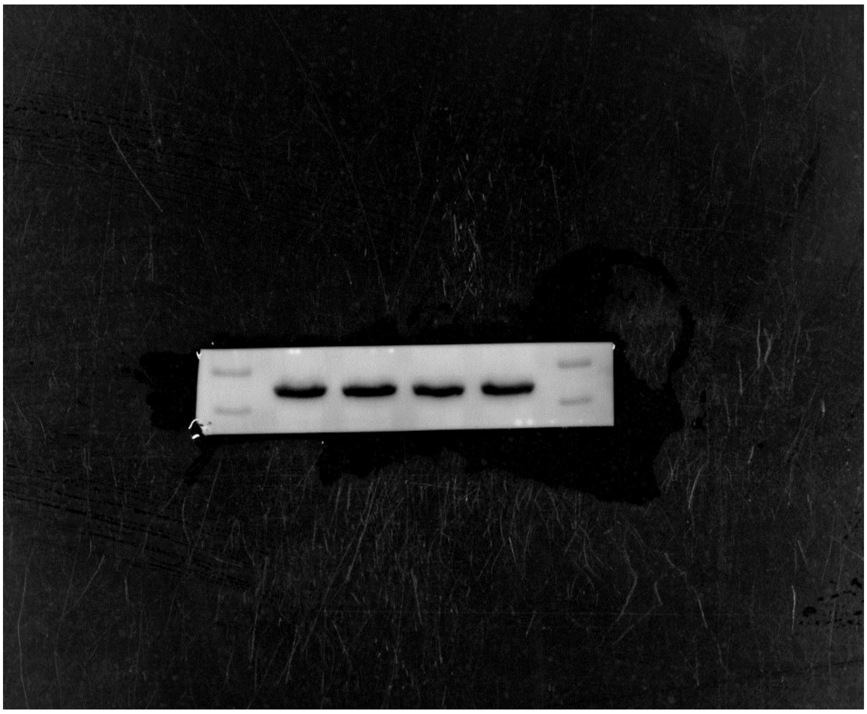

RAD51

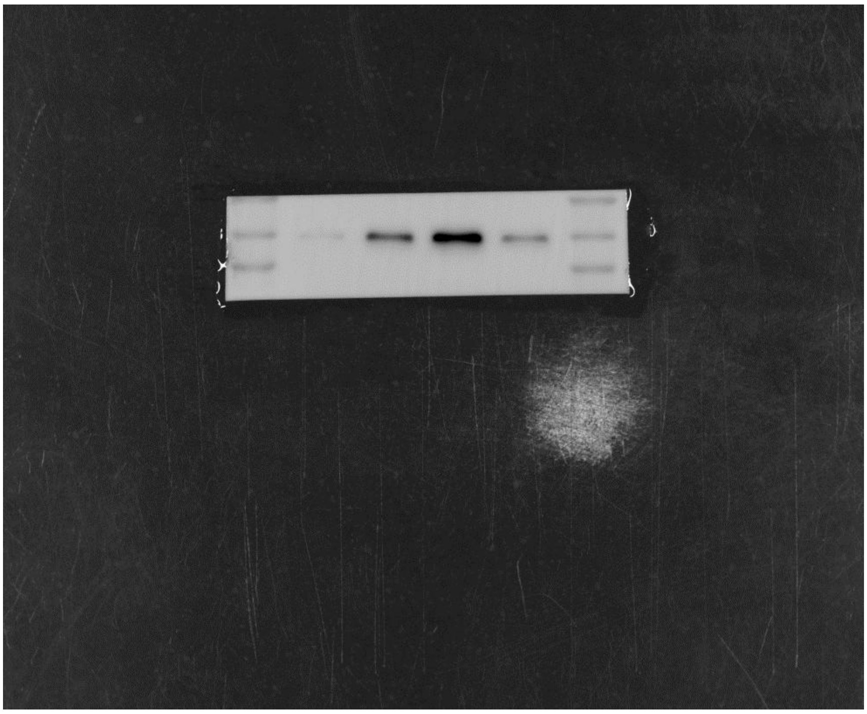

H3

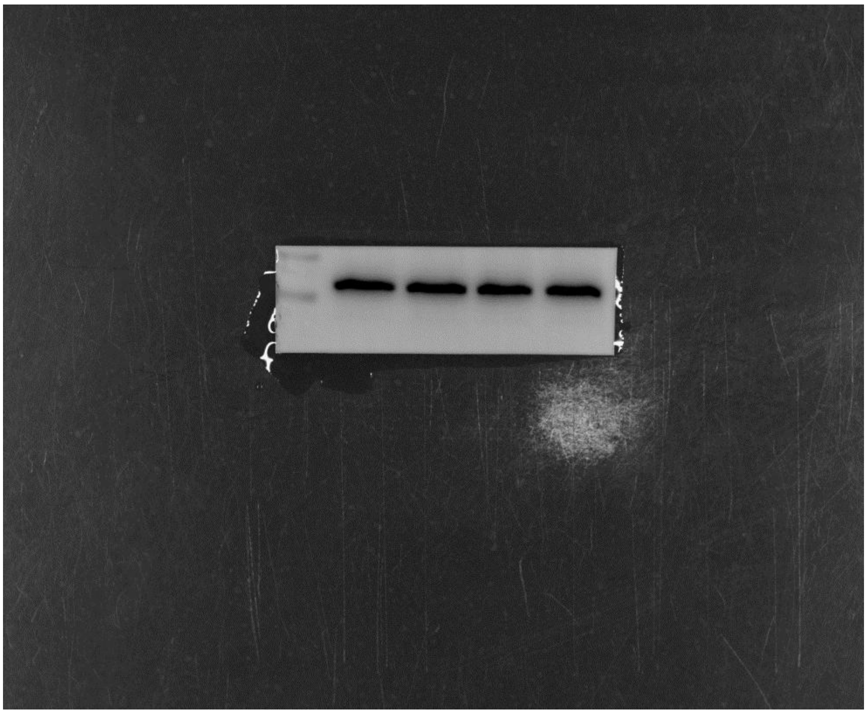

Figure3E

IP-HA

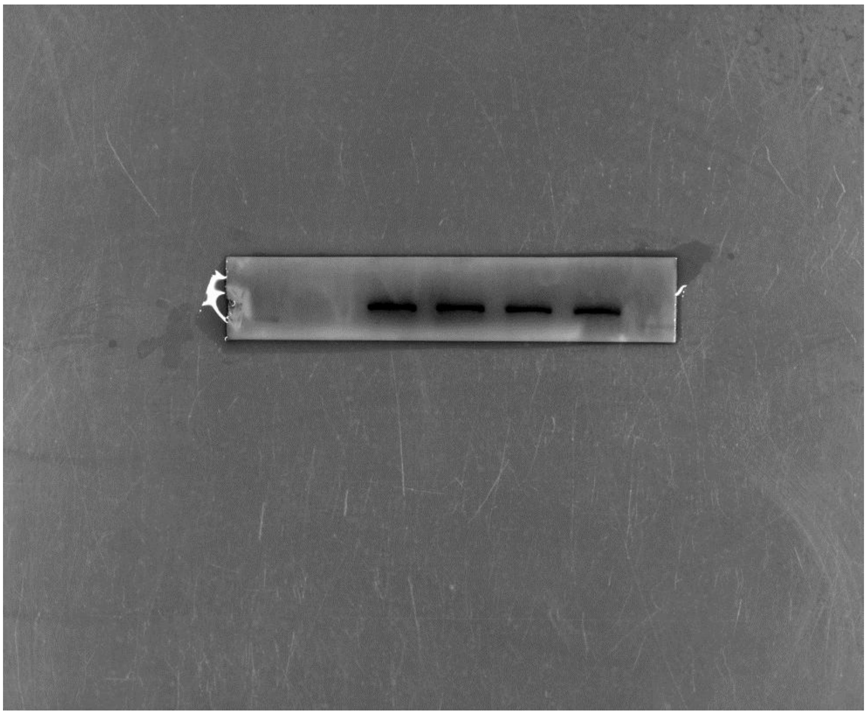

IP-Kla

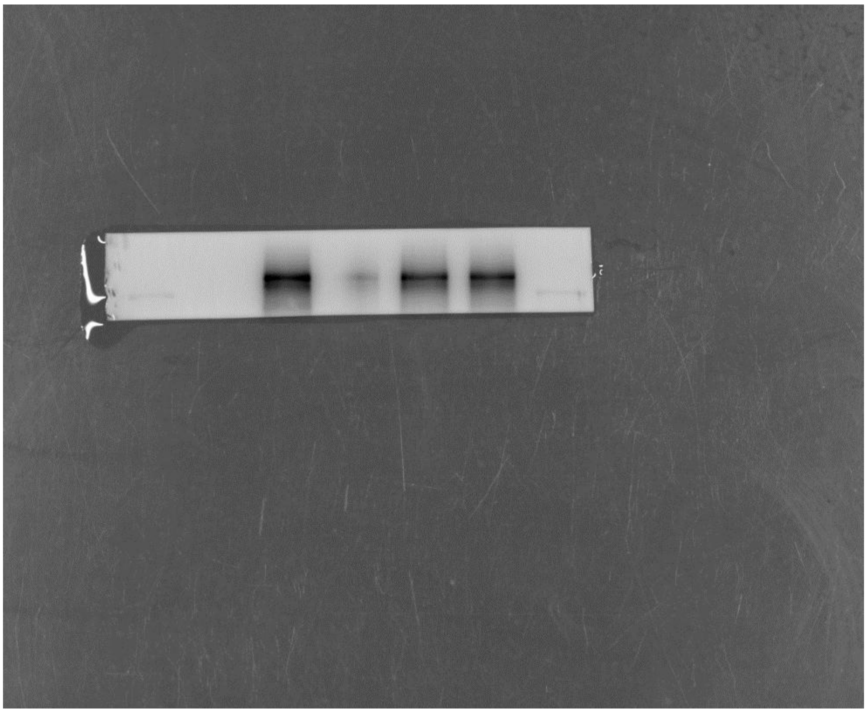

Input-HA

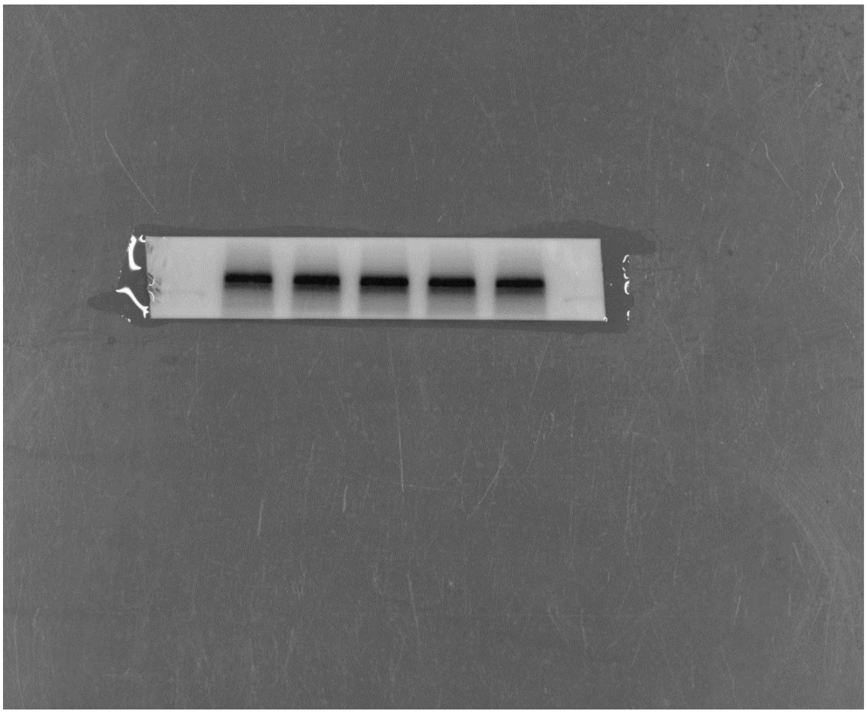

Figure3H

BLM-K24la

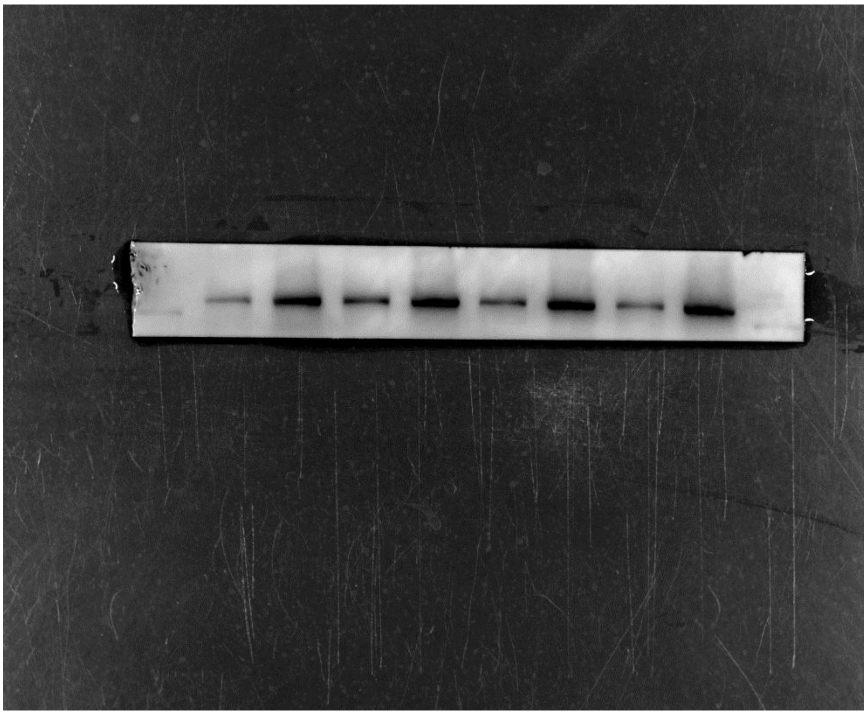

β-actin

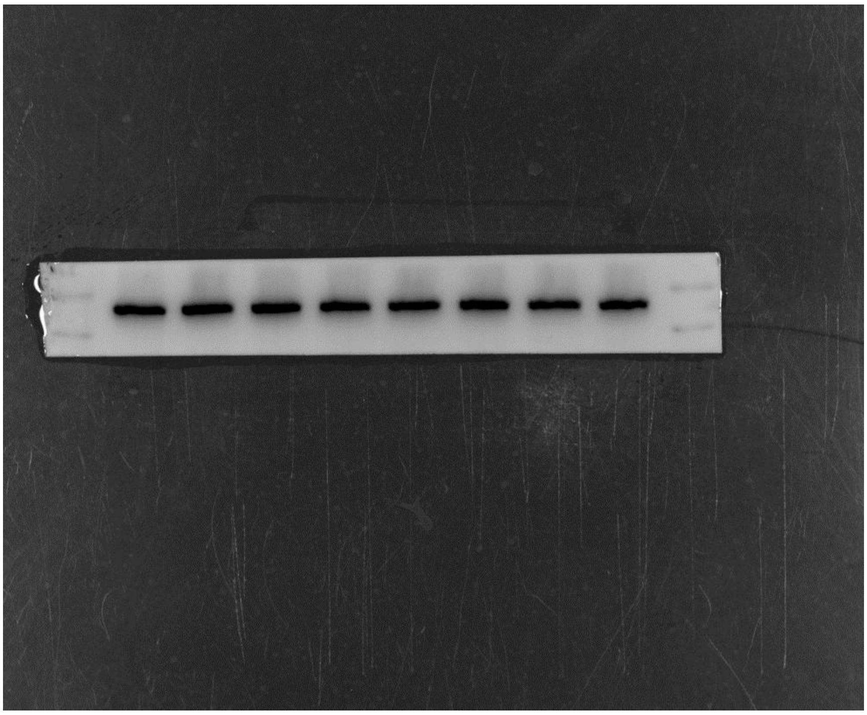

Figure3I

BLM-K24la

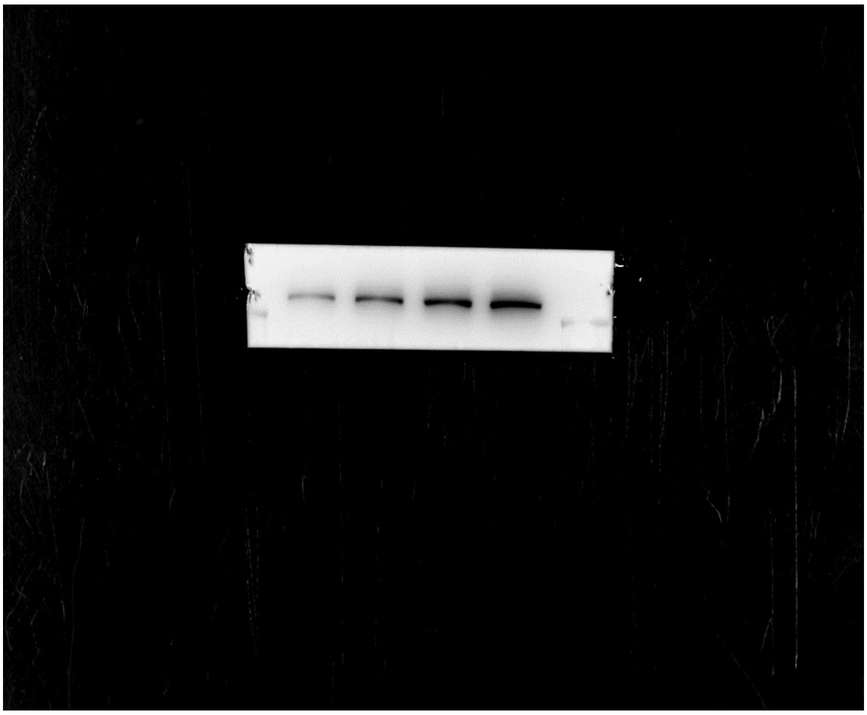

β-actin

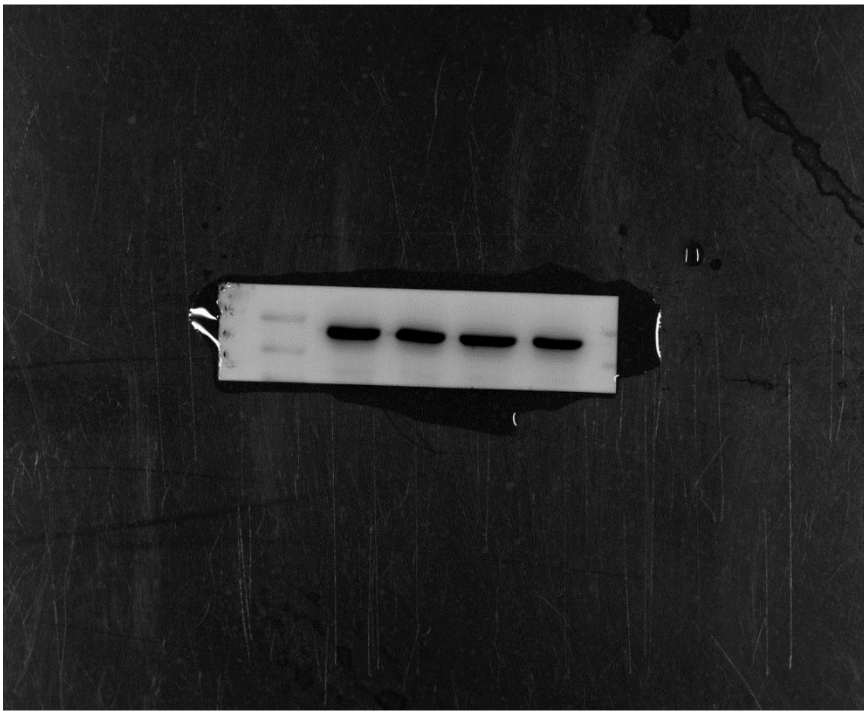

Figure3J

BLM-K24la

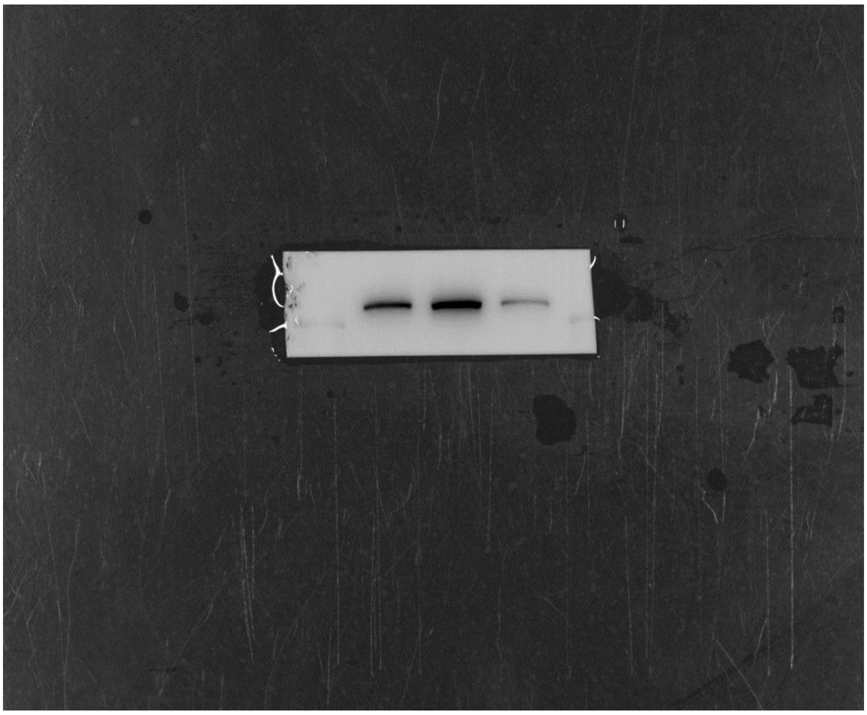

β-actin

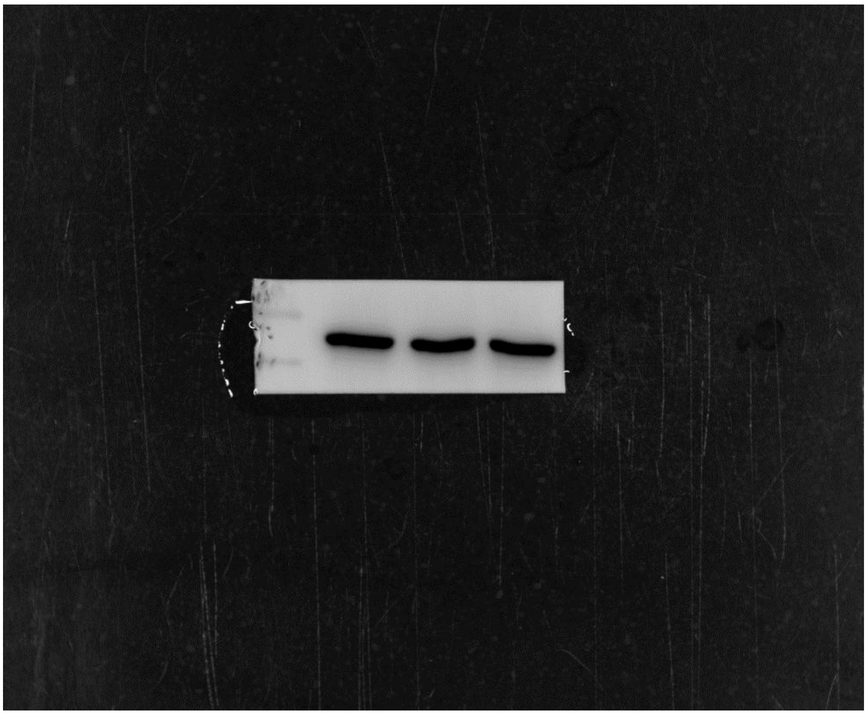

Figure3K

IP-HA

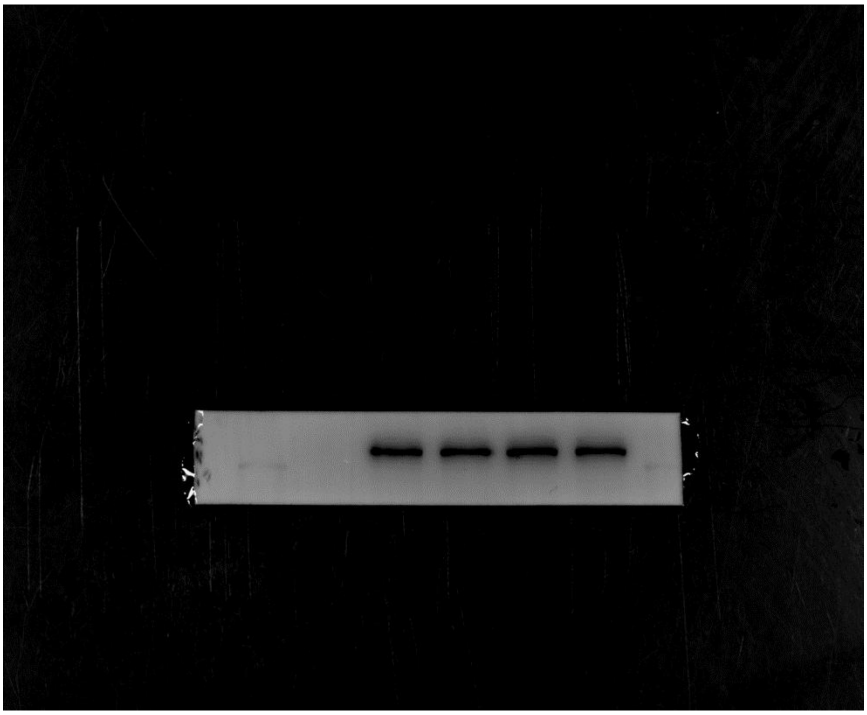

IP-K24la

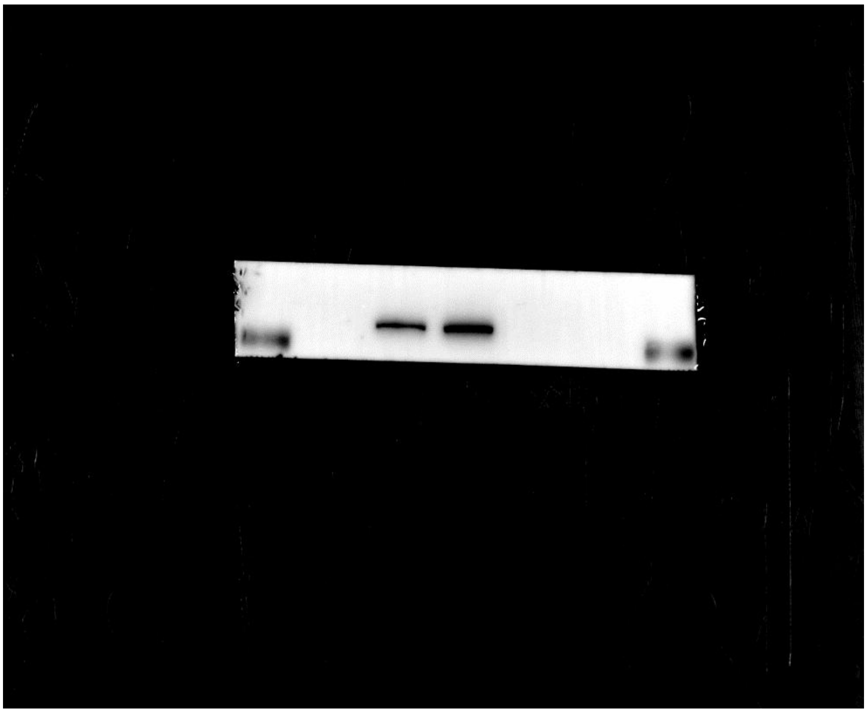

Input-HA

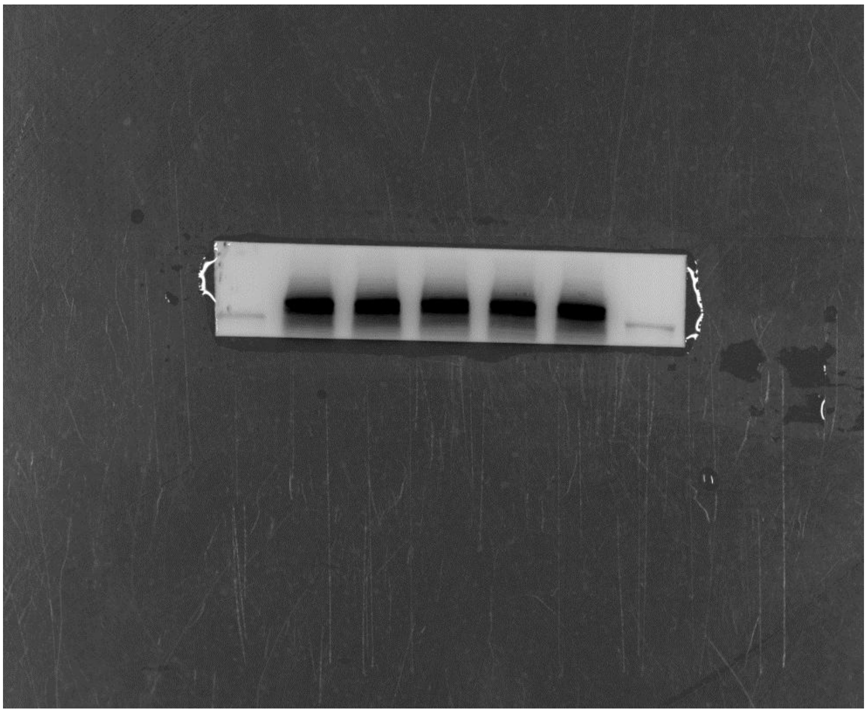

Figure4B

RAD51

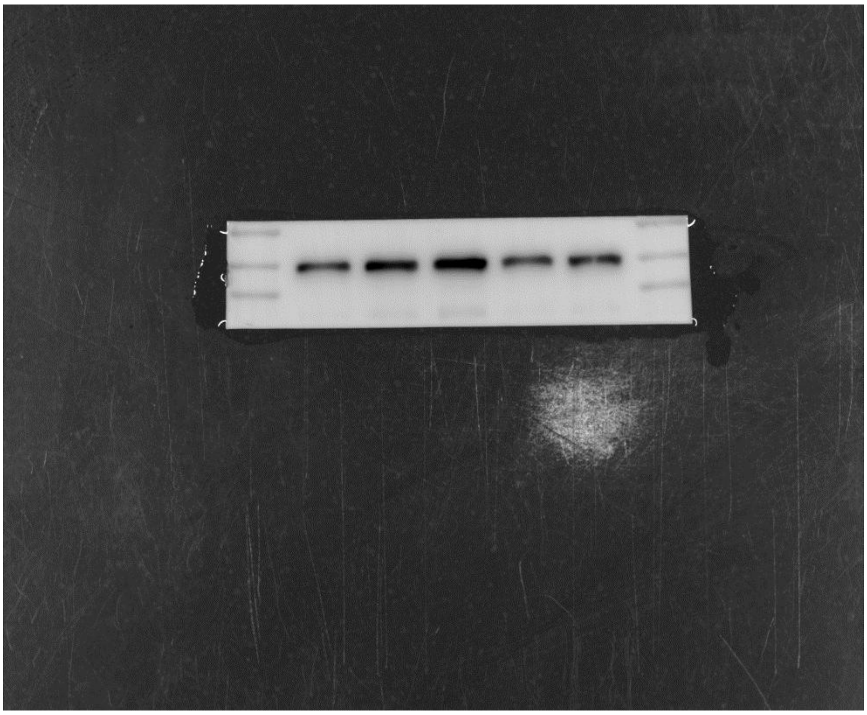

H3

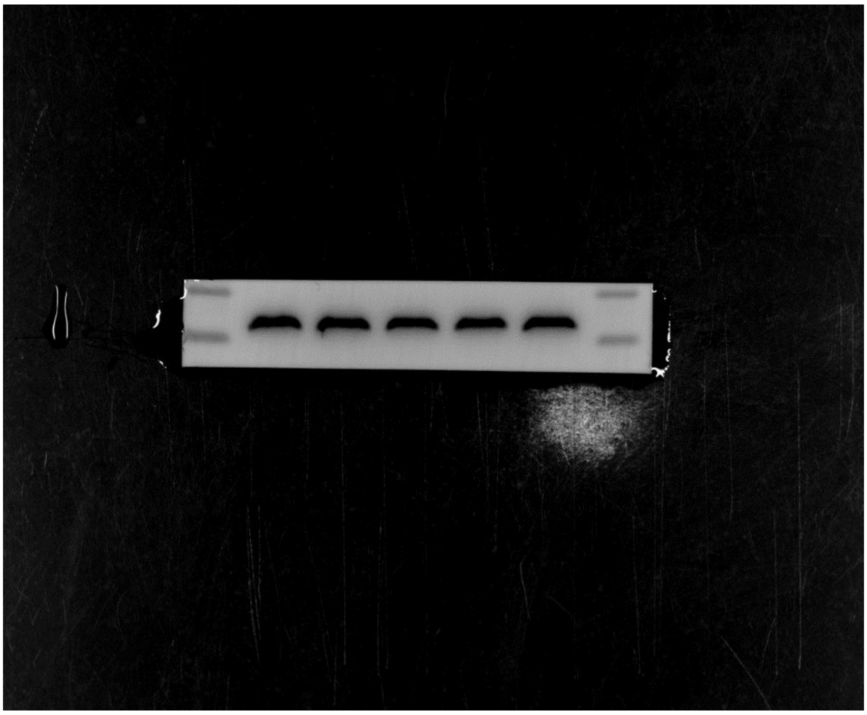

γH2AX

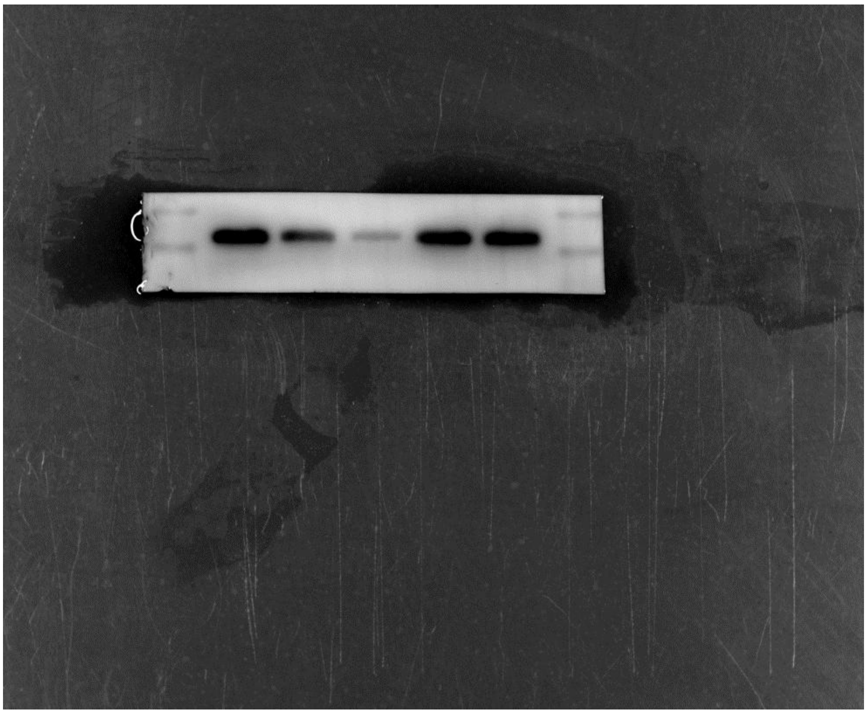

β-actin

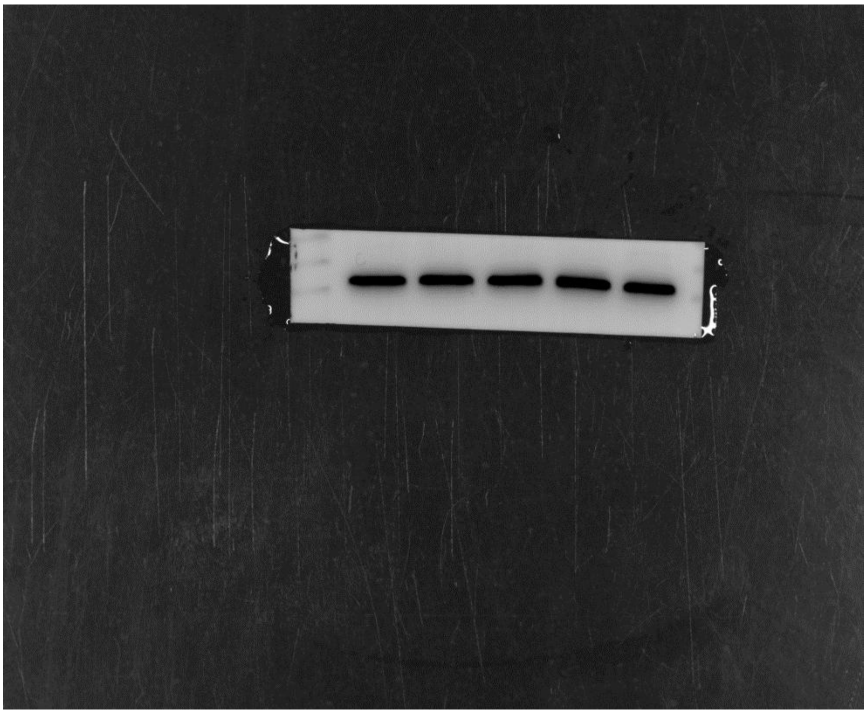

Figure4D

IP-HA

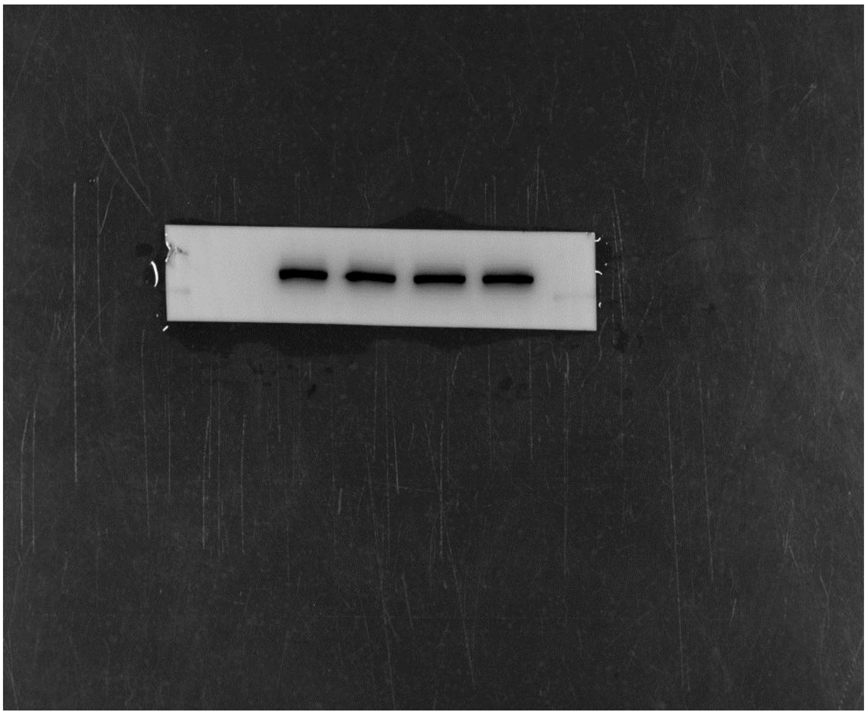

IP-DNA2

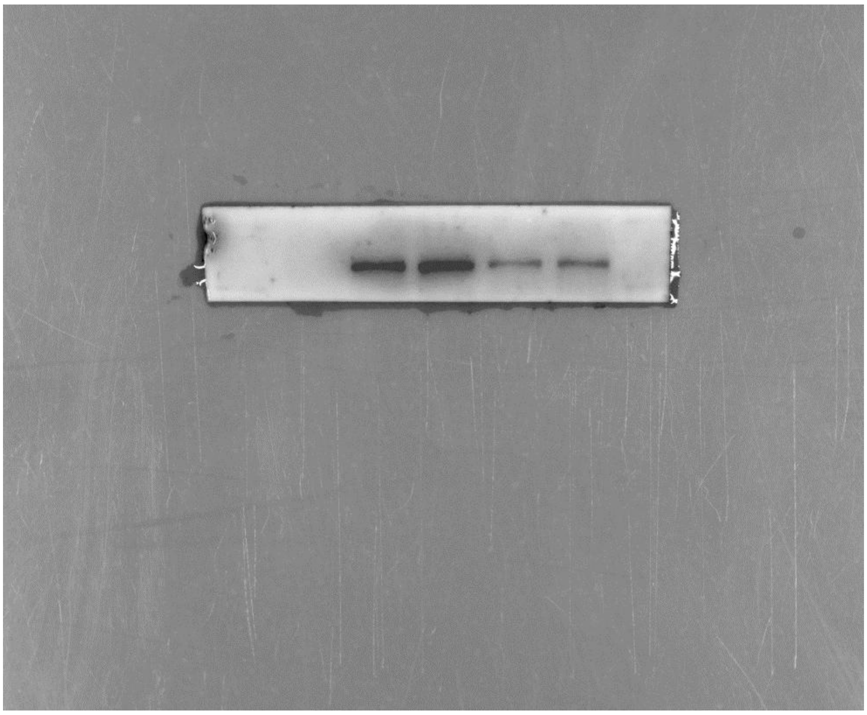

IP-TOPIIA

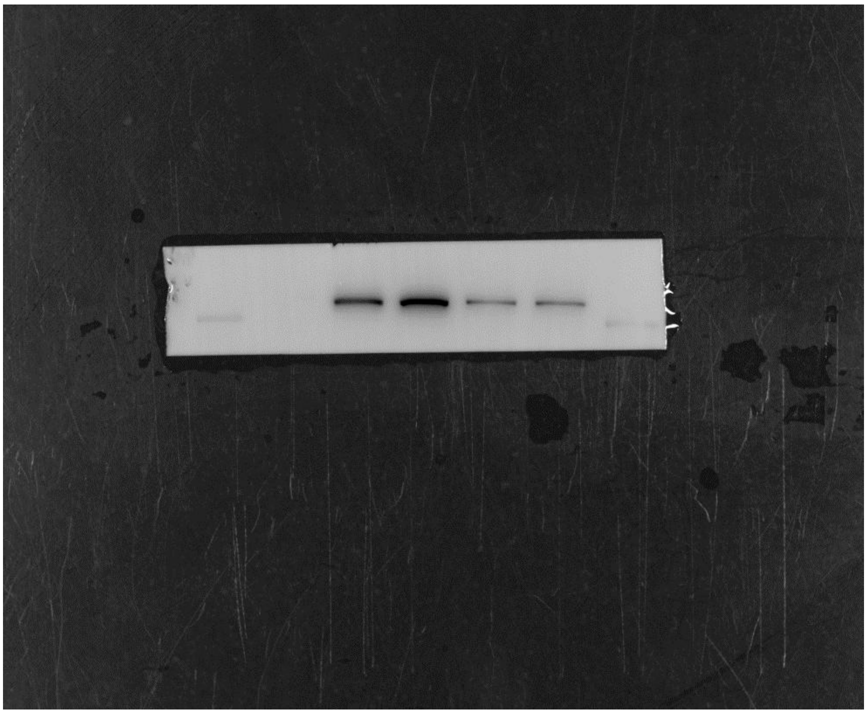

IP-RPA

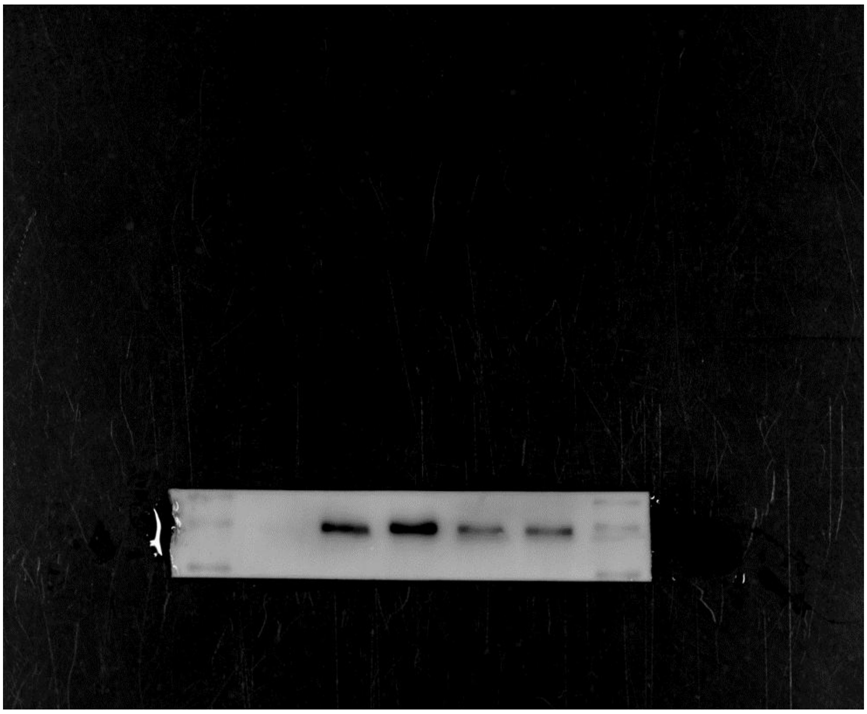

IP-RAD54

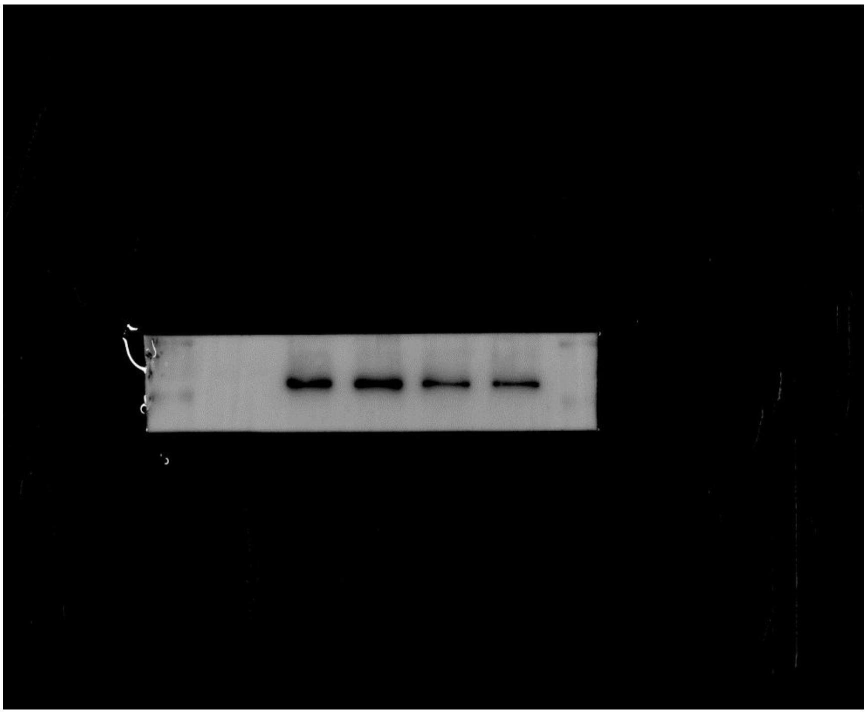

Input-HA

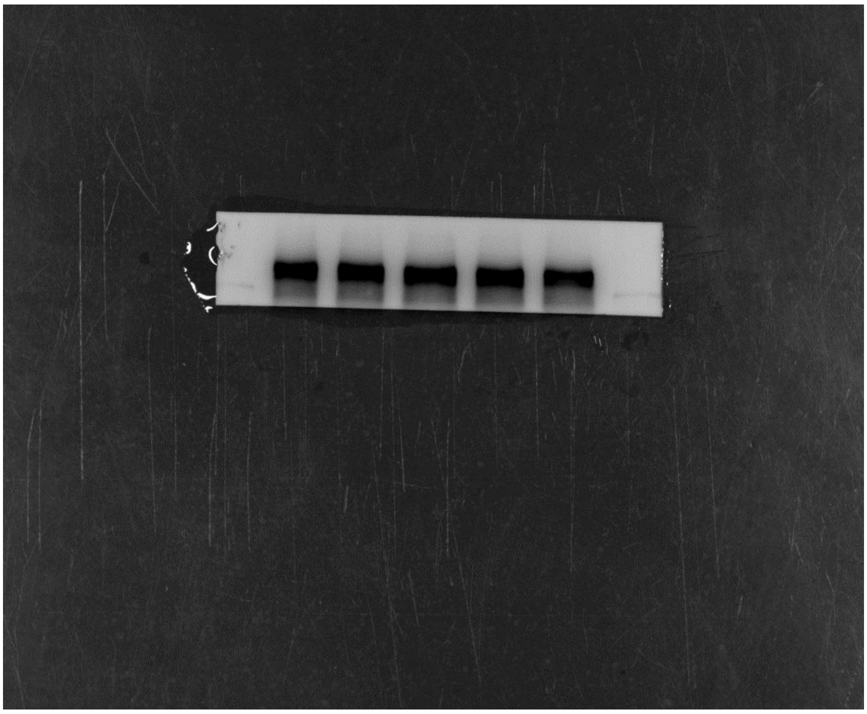

Input-DNA2

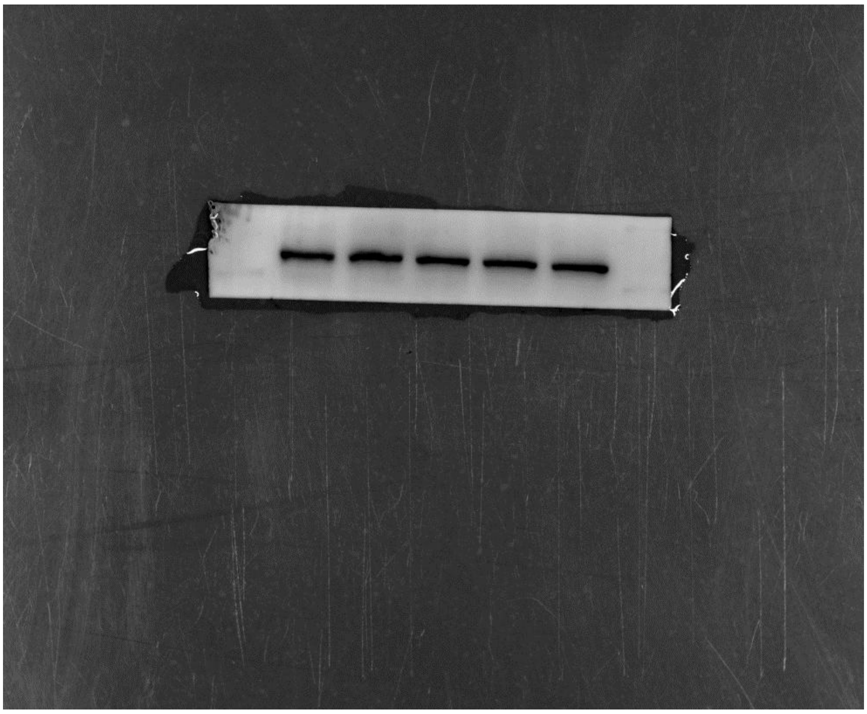

Input-TOPIIA

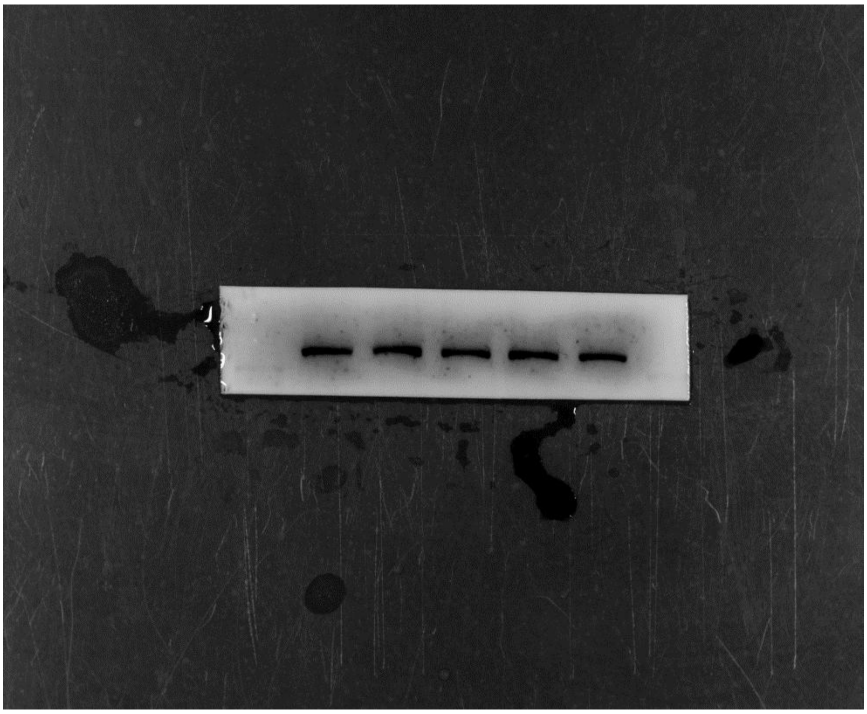

Input-RPA

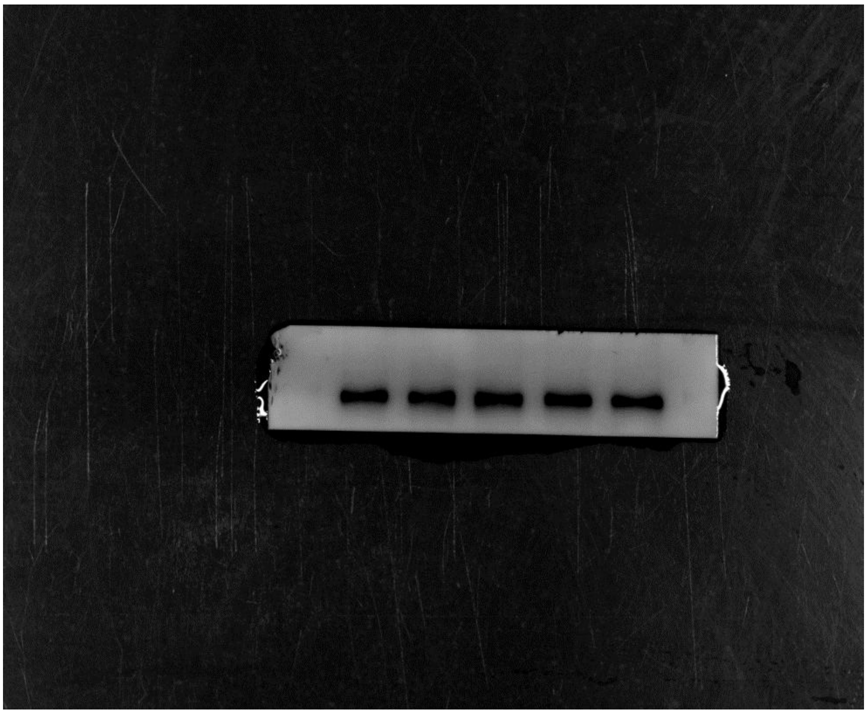

Input-RAD54

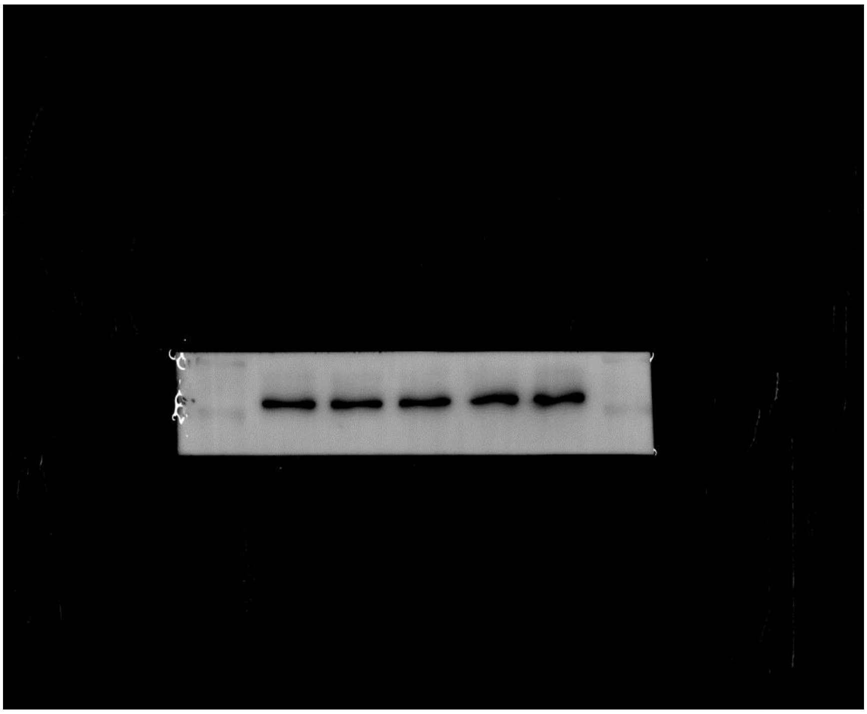

Figure4E

HA

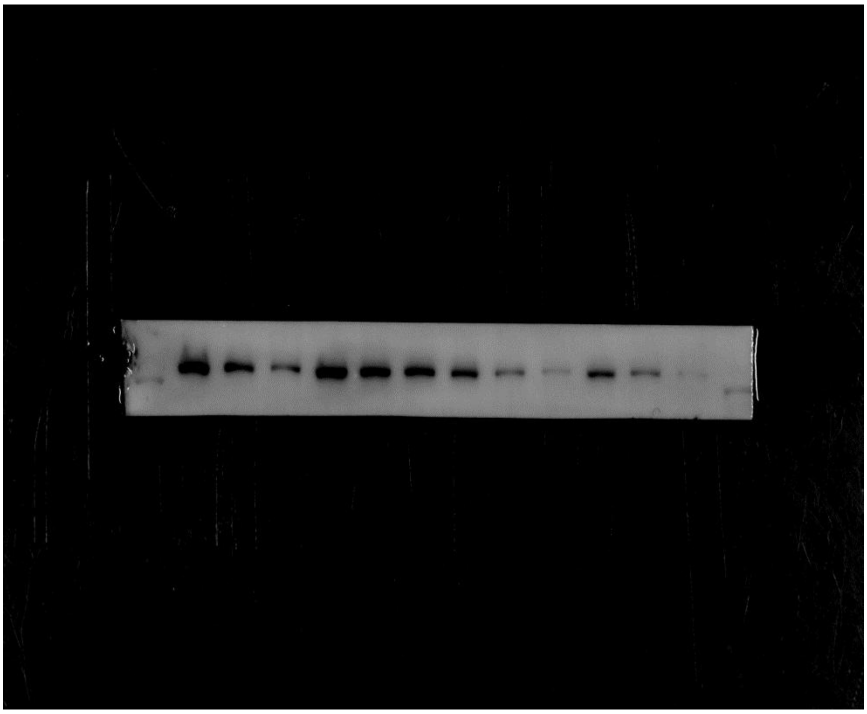

β-actin

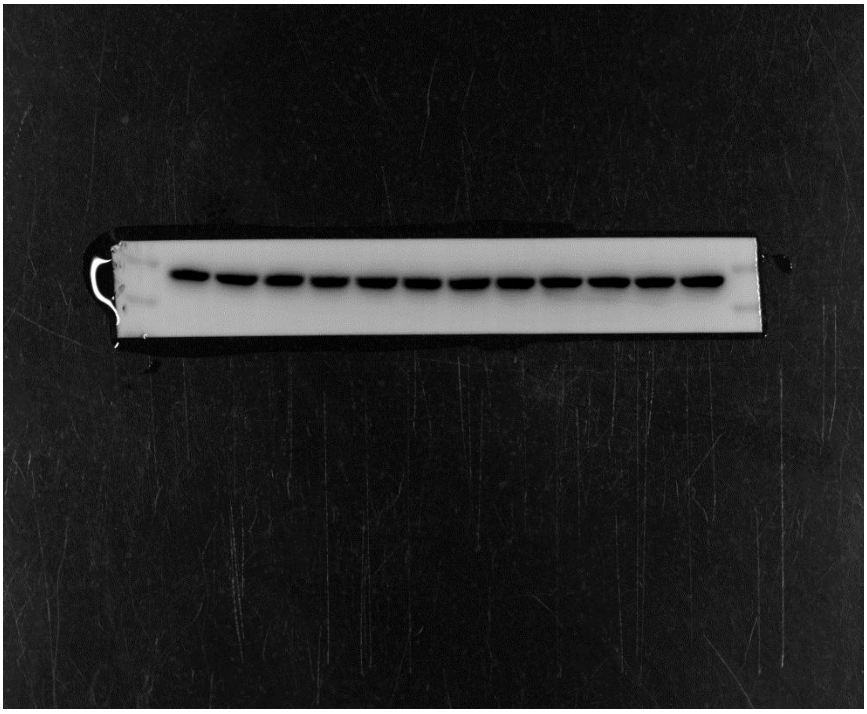

Figure4G

IP-HA

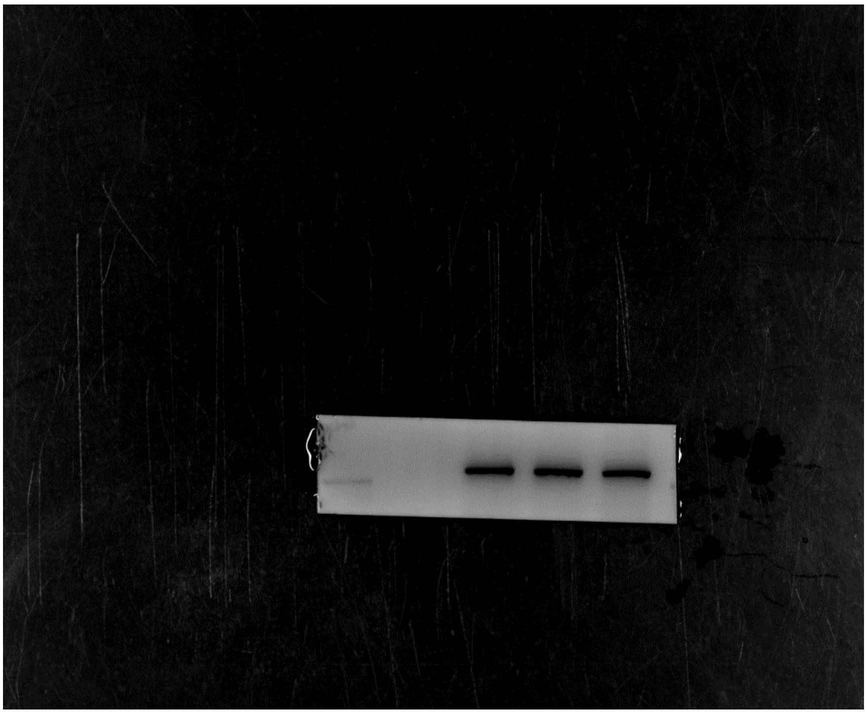

IP-K24la

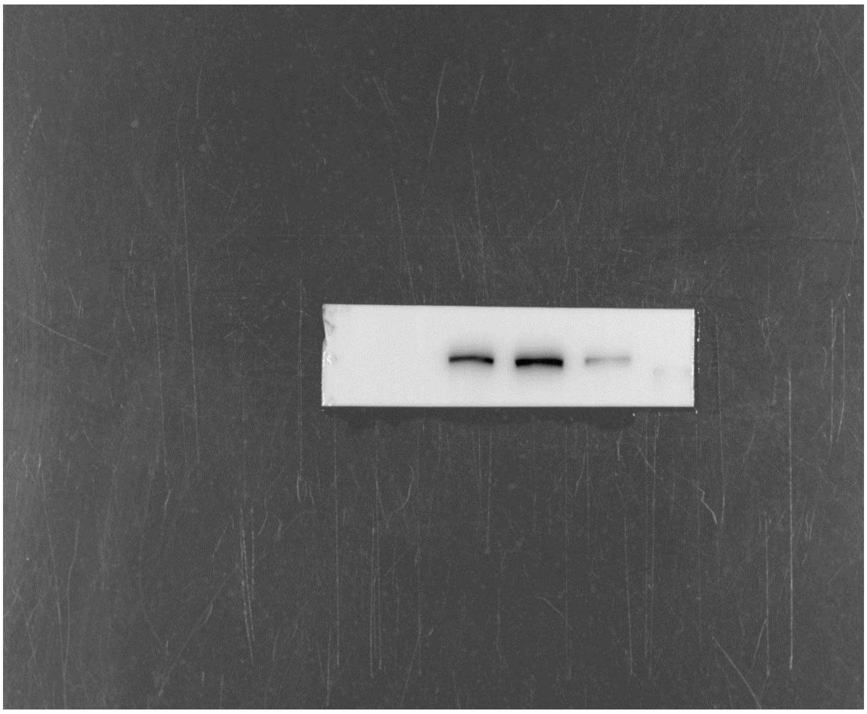

IP-Flag

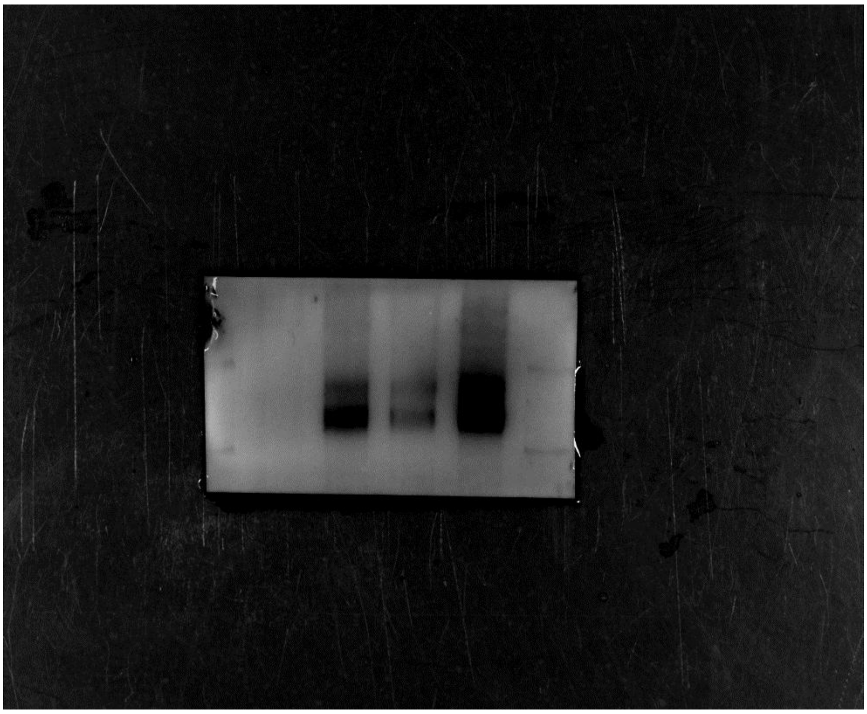

Input-HA

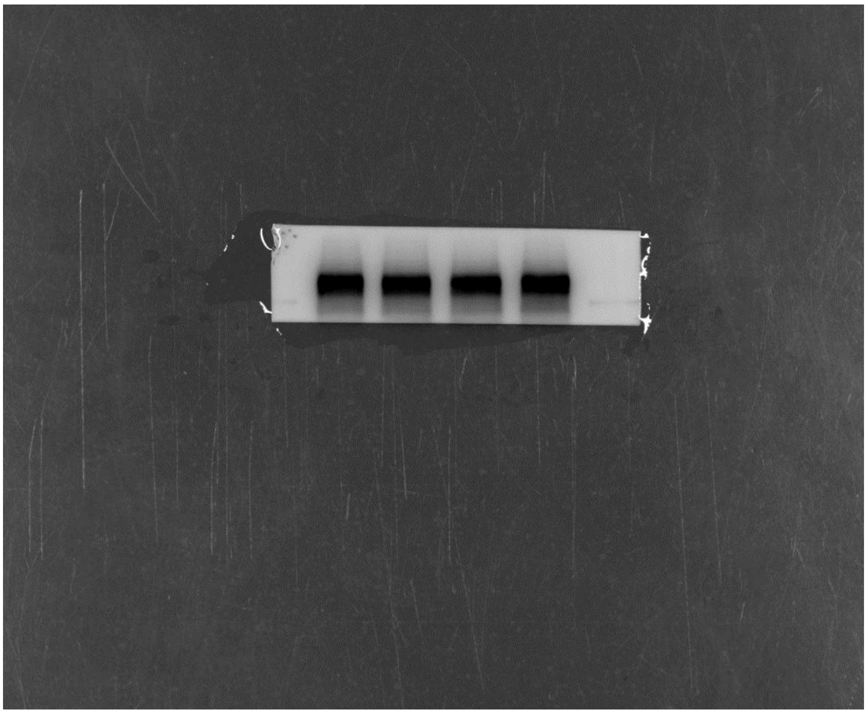

Figure4J

IP-Flag

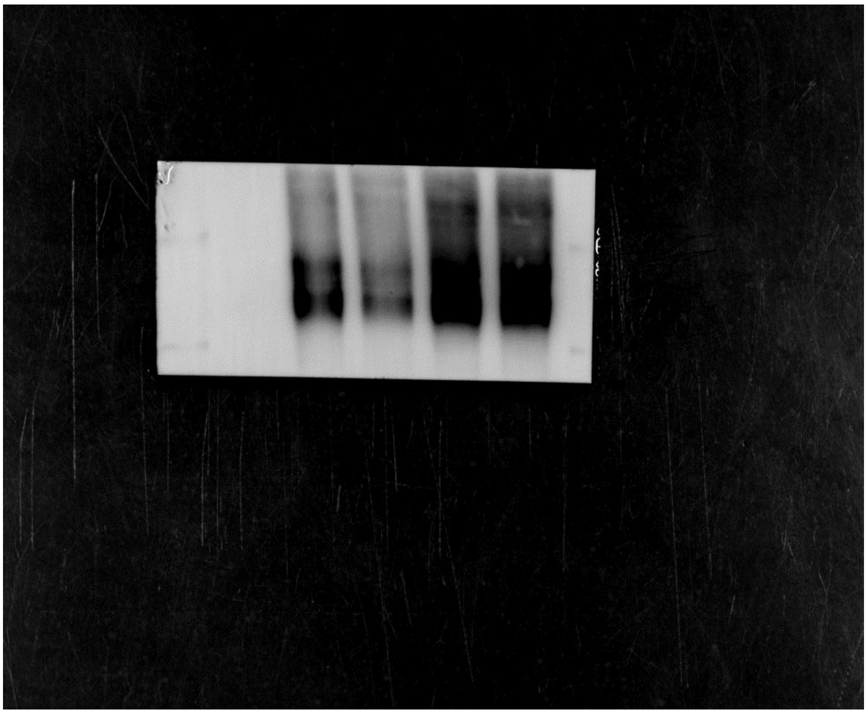

IB-His

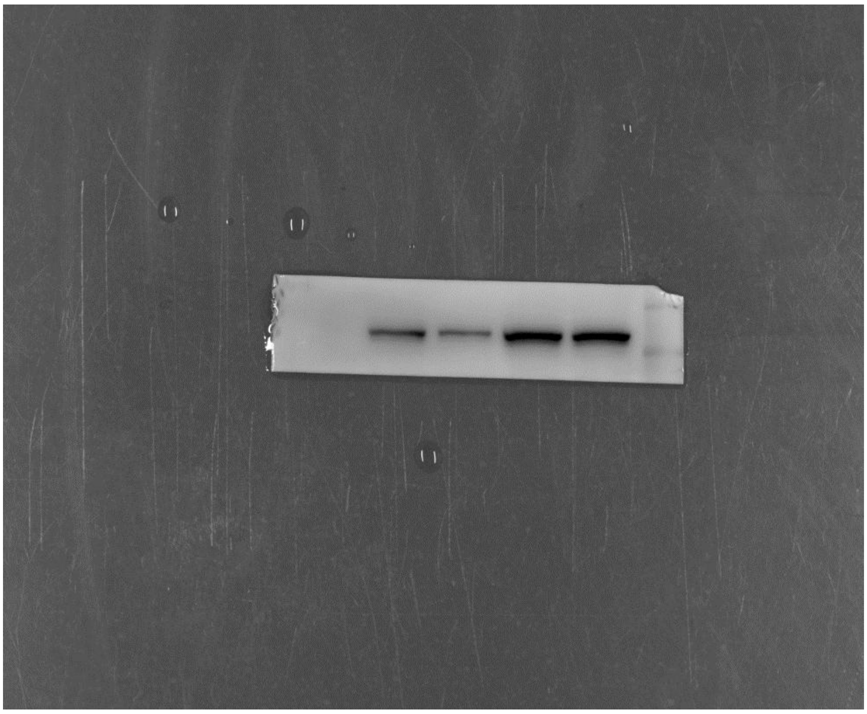

IP-HA

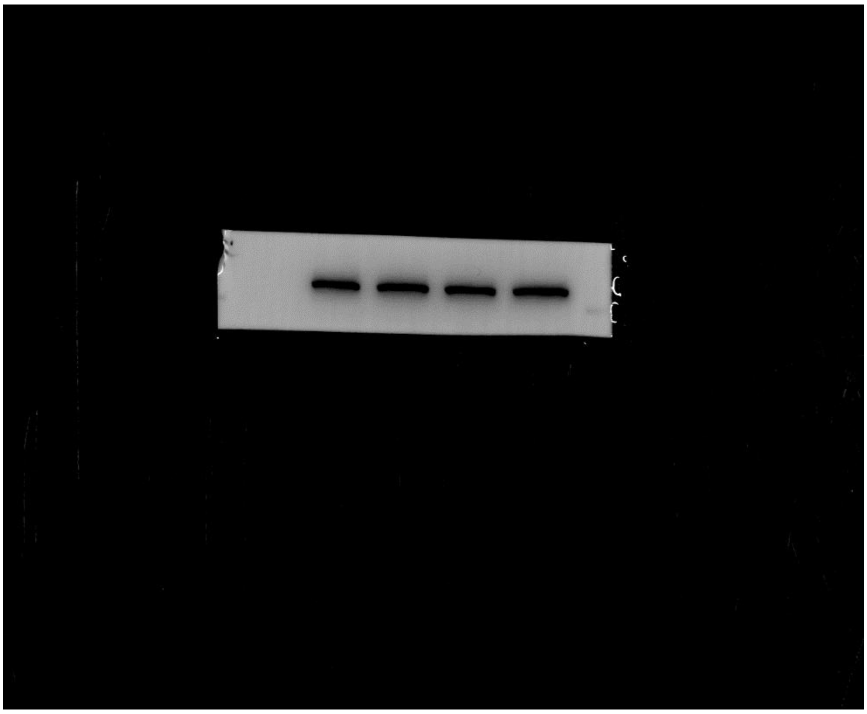

IP-Kla

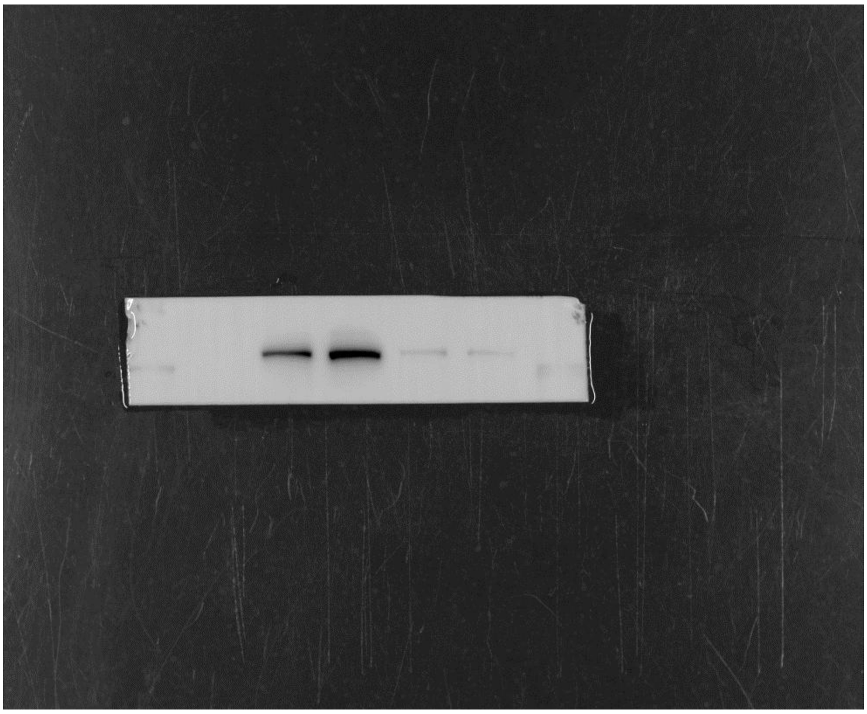

Input-His

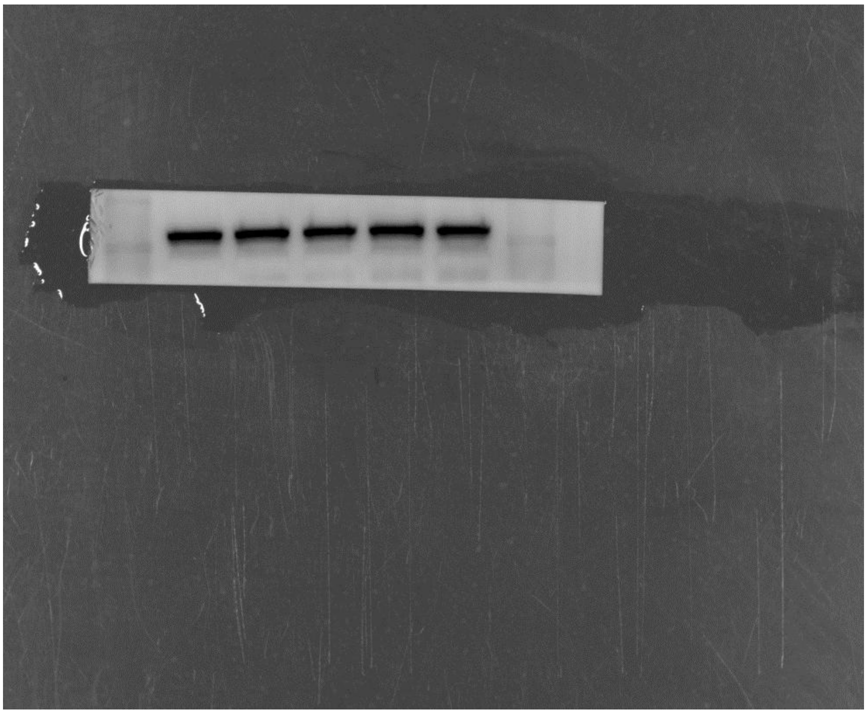

Input-HA

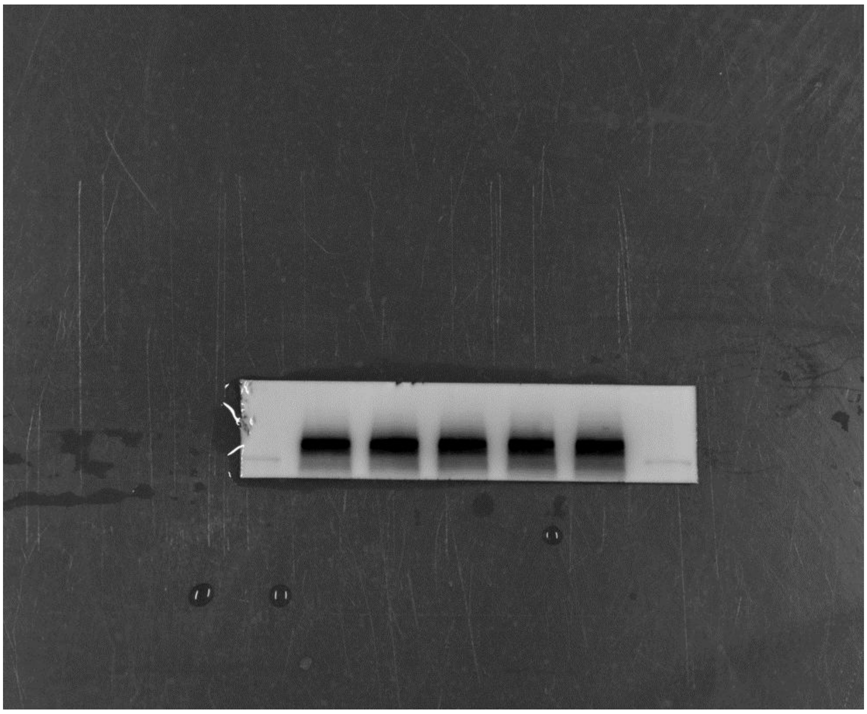

Figure5A

BLM-K24R

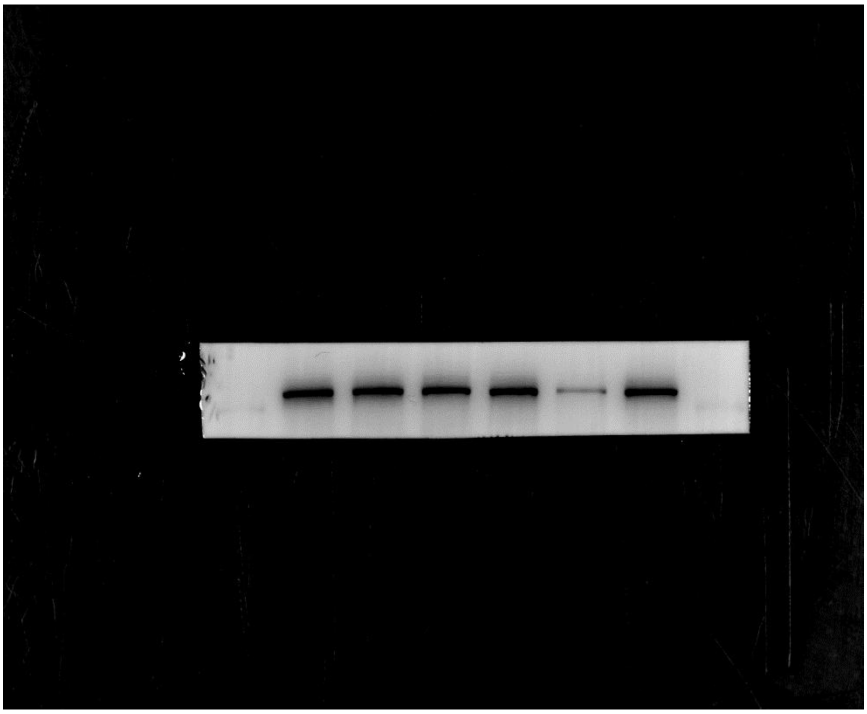

β-actin

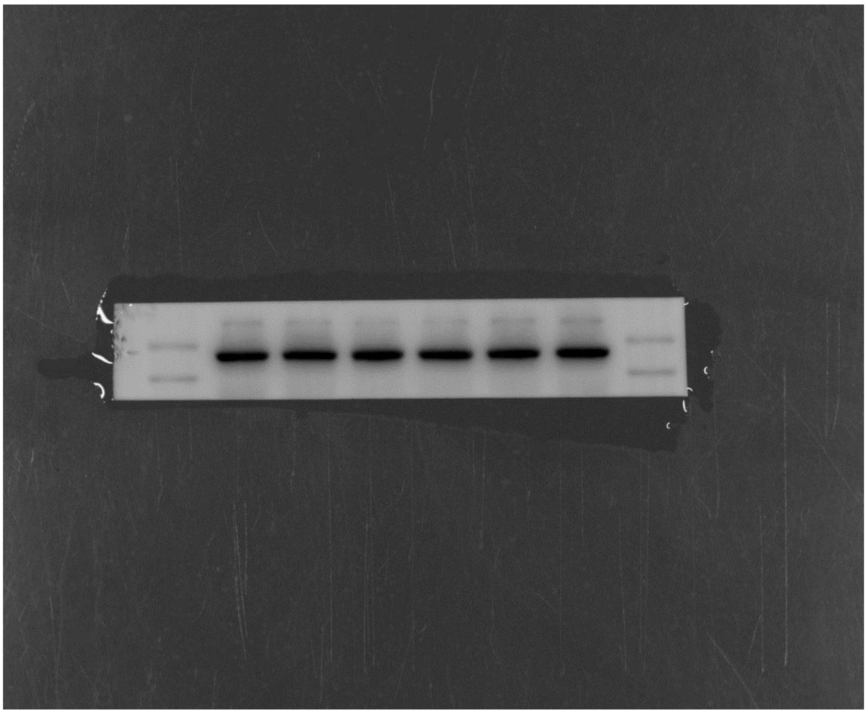

Figure5B

AARS1

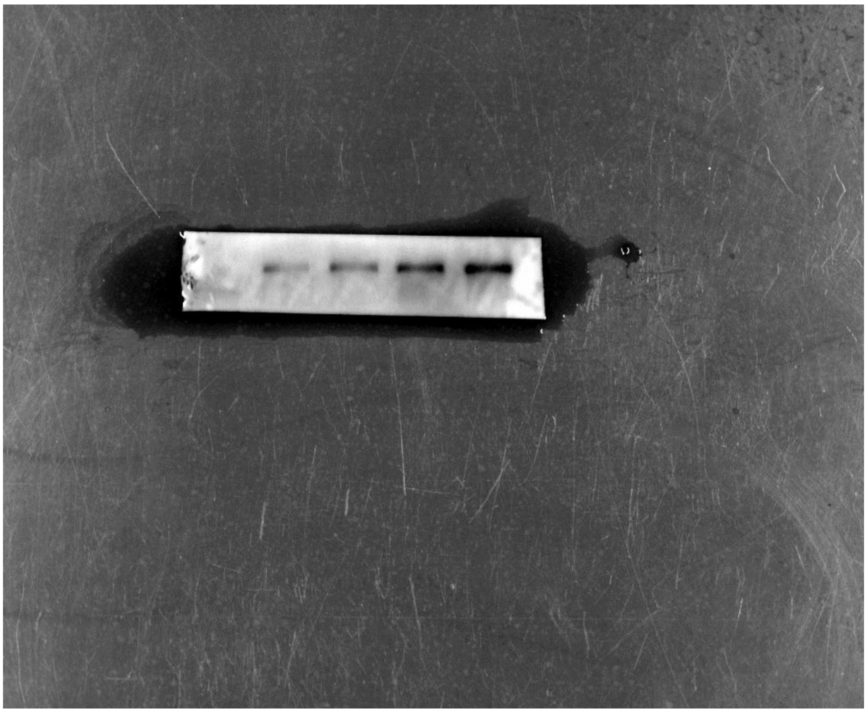

β-actin

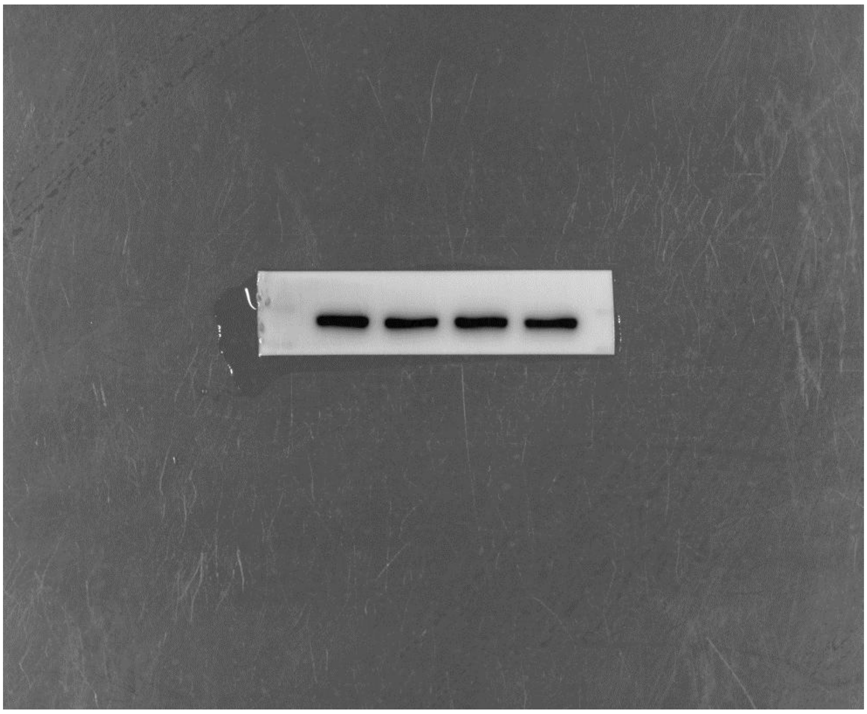

Figure5C

IP-BLM

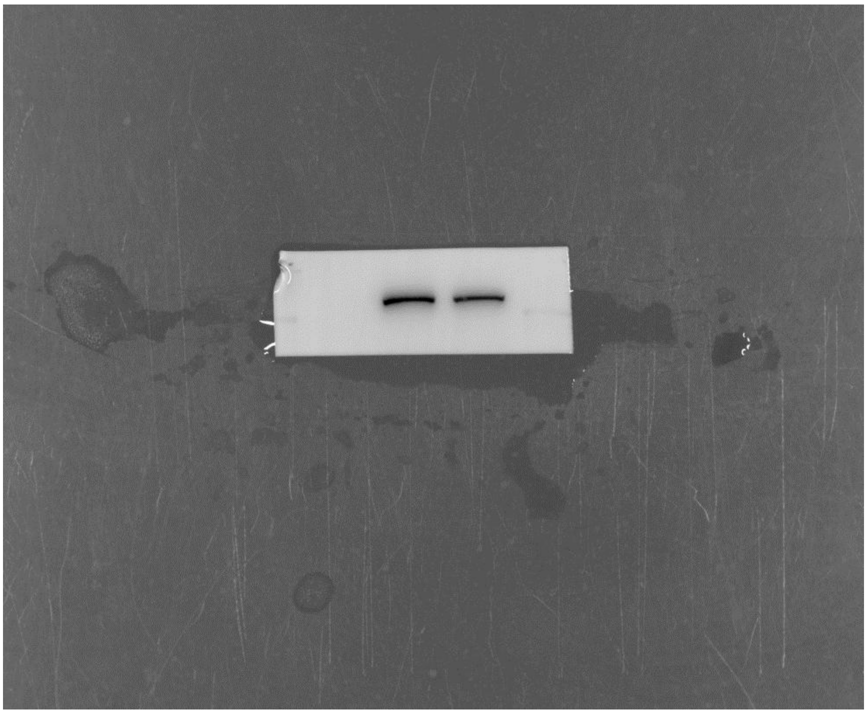

IP-AARS1

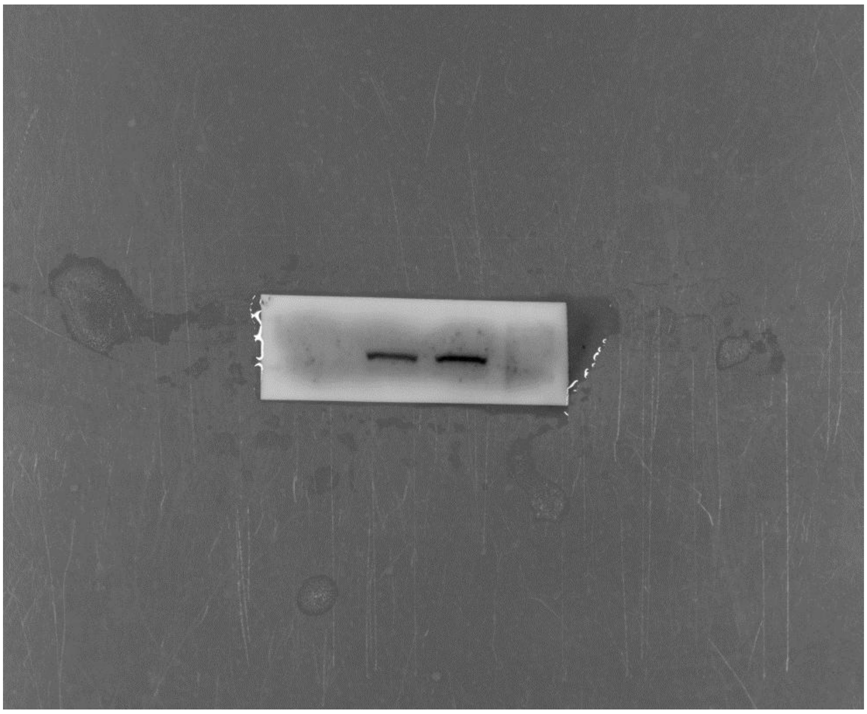

Input-BLM

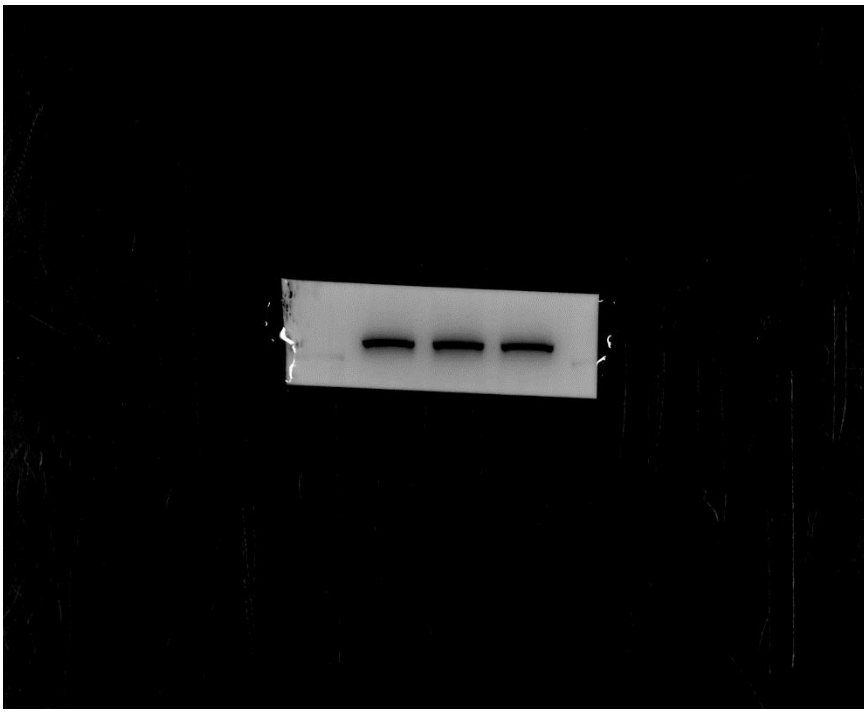

Input-AARS1

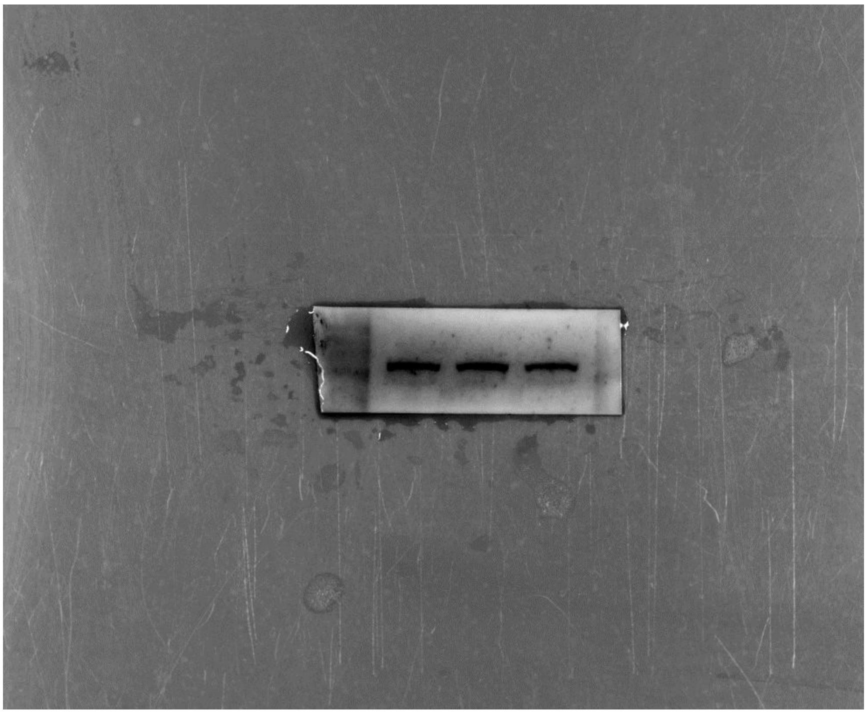

Figure5D

IP-BLM

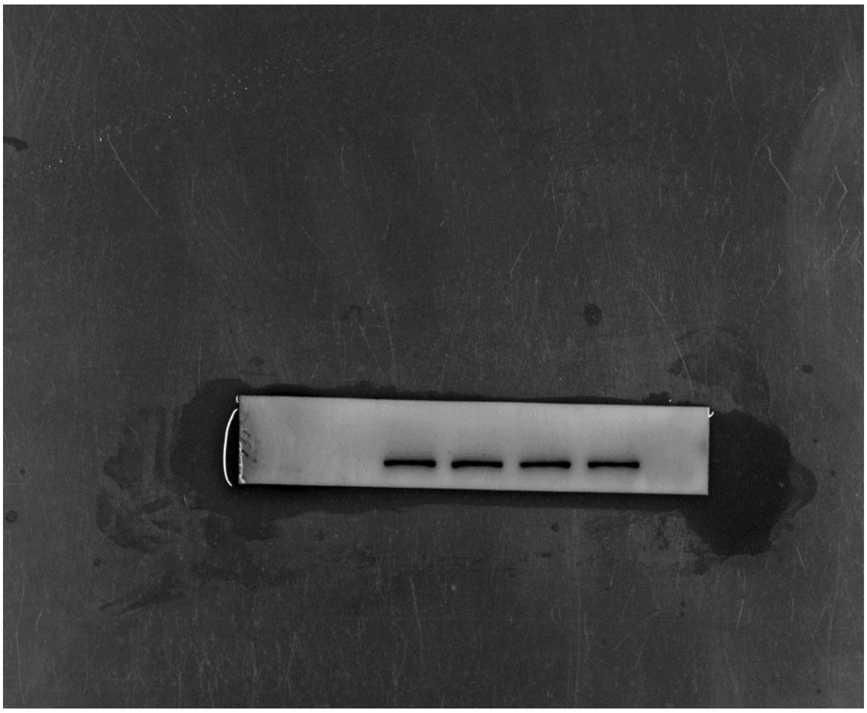

IP-Kla

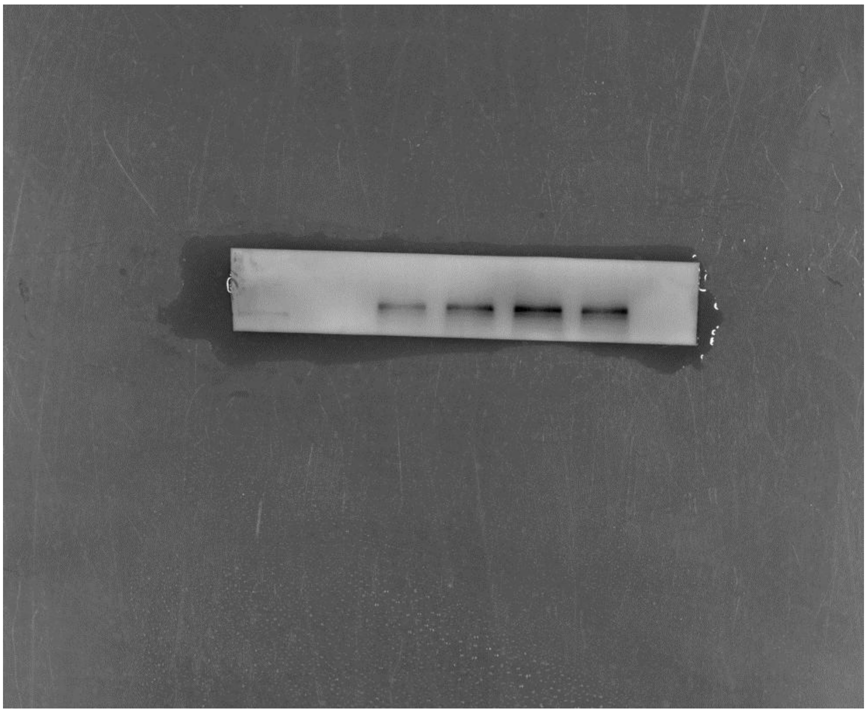

Input-BLM

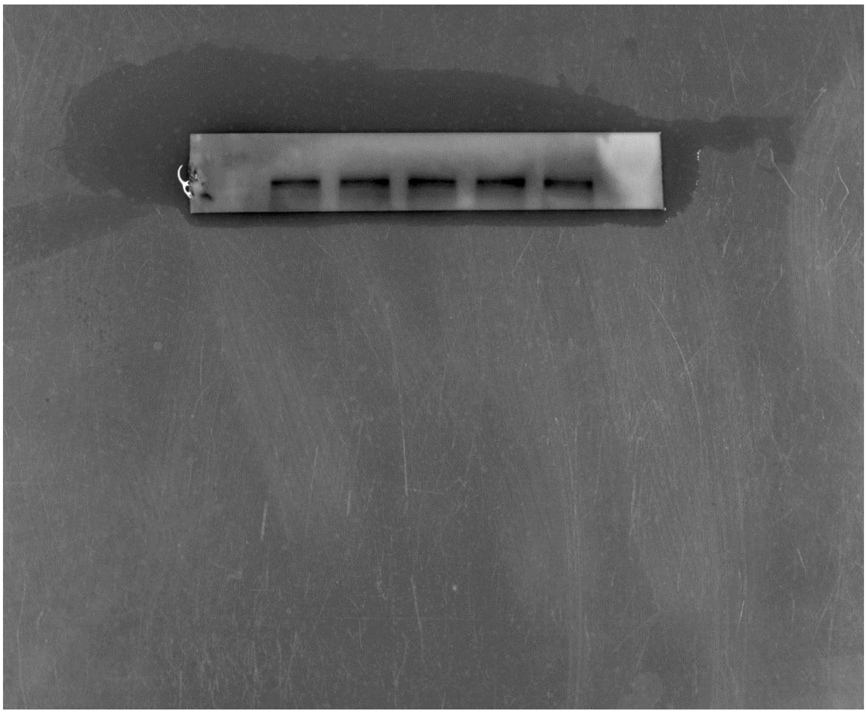

Figure5G

RAD51

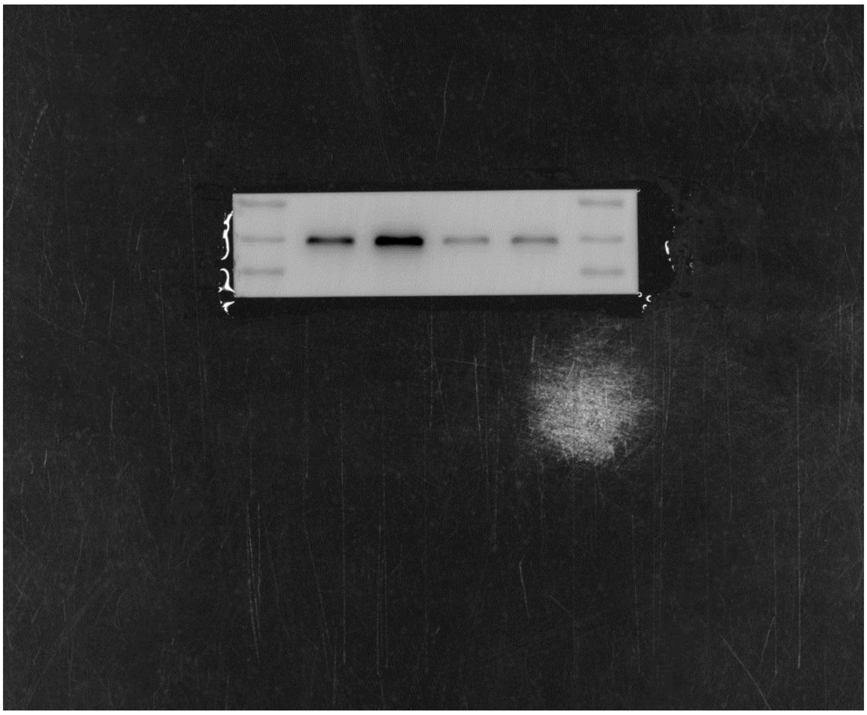

H3

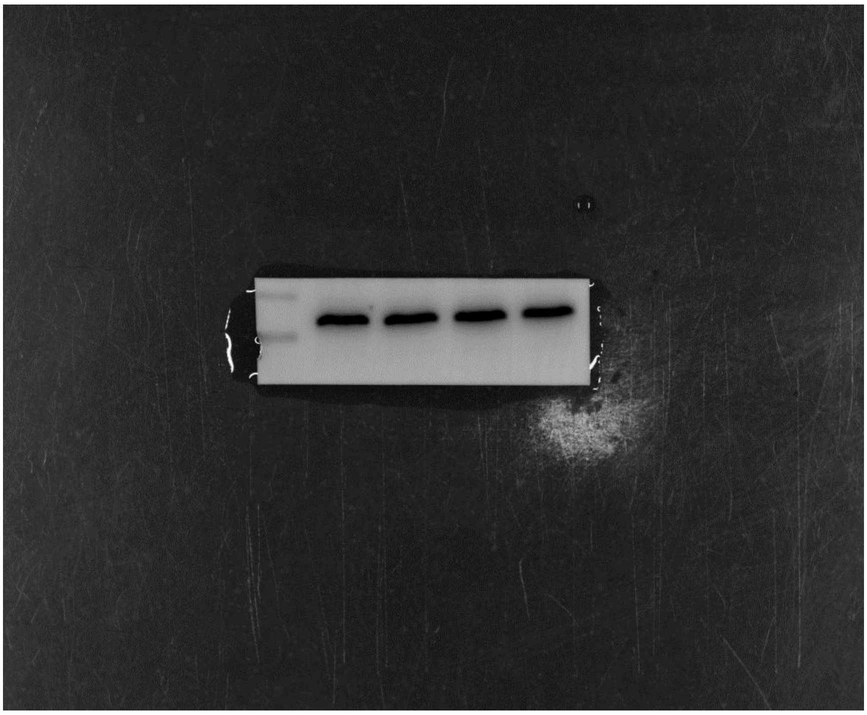

γH2AX

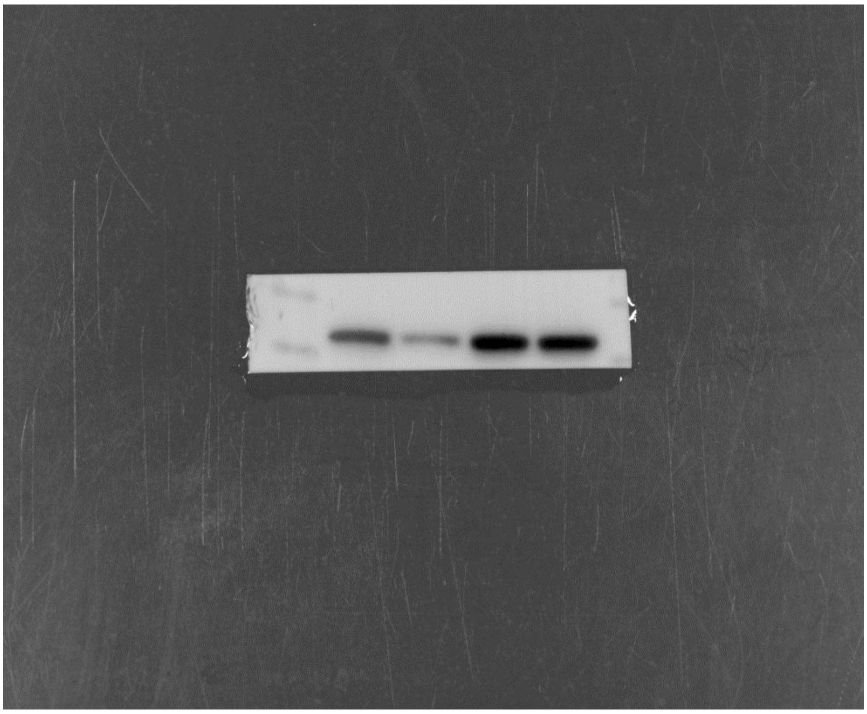

β-actin

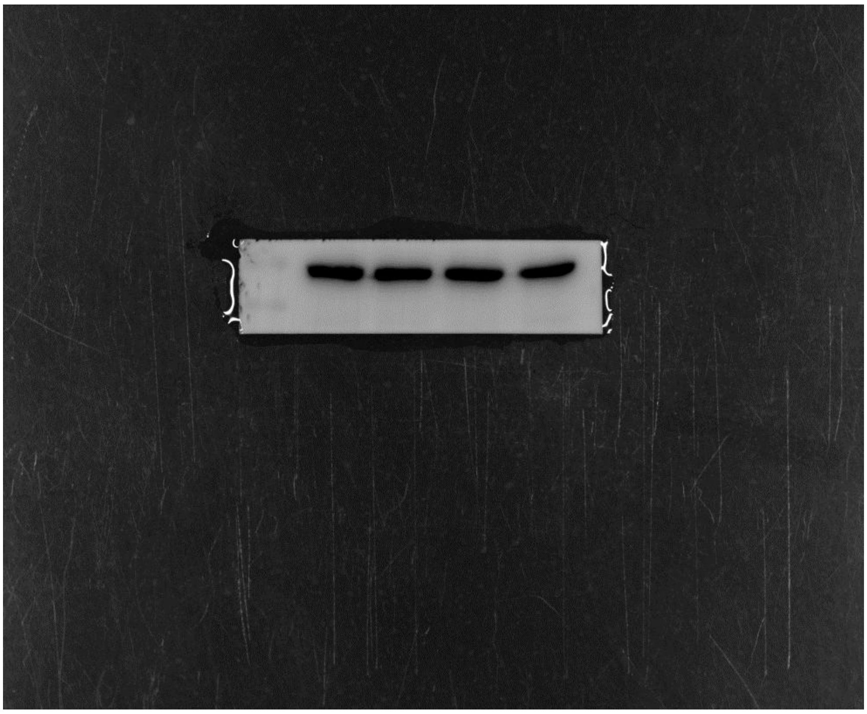

Figure5J

HA

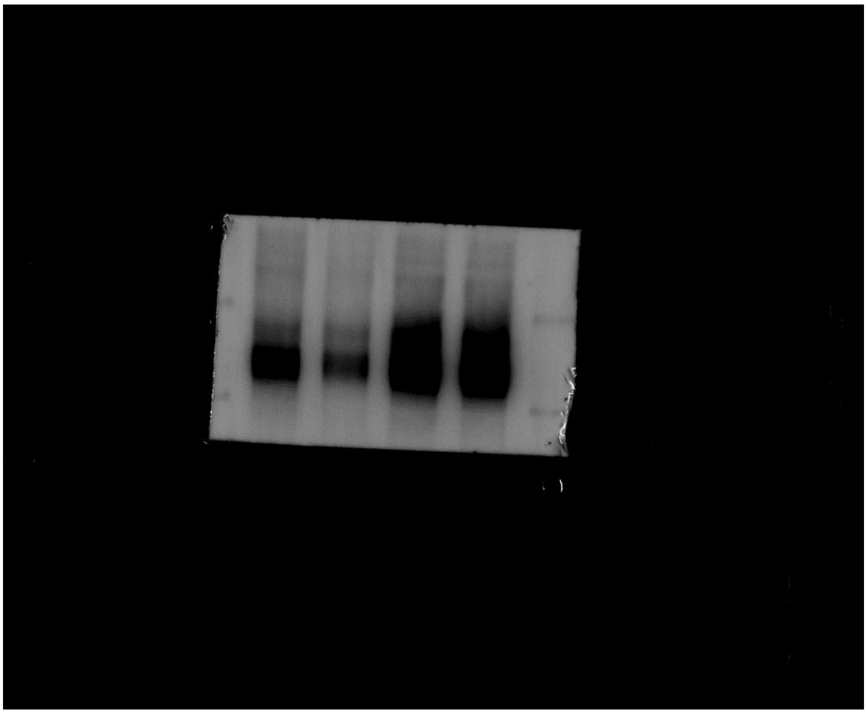

His

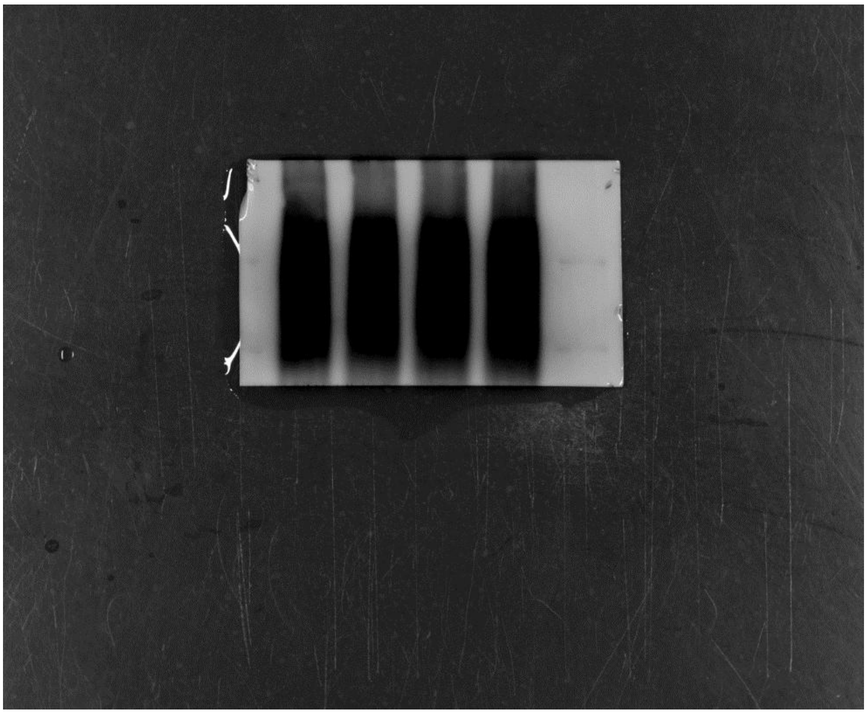

Input-HA

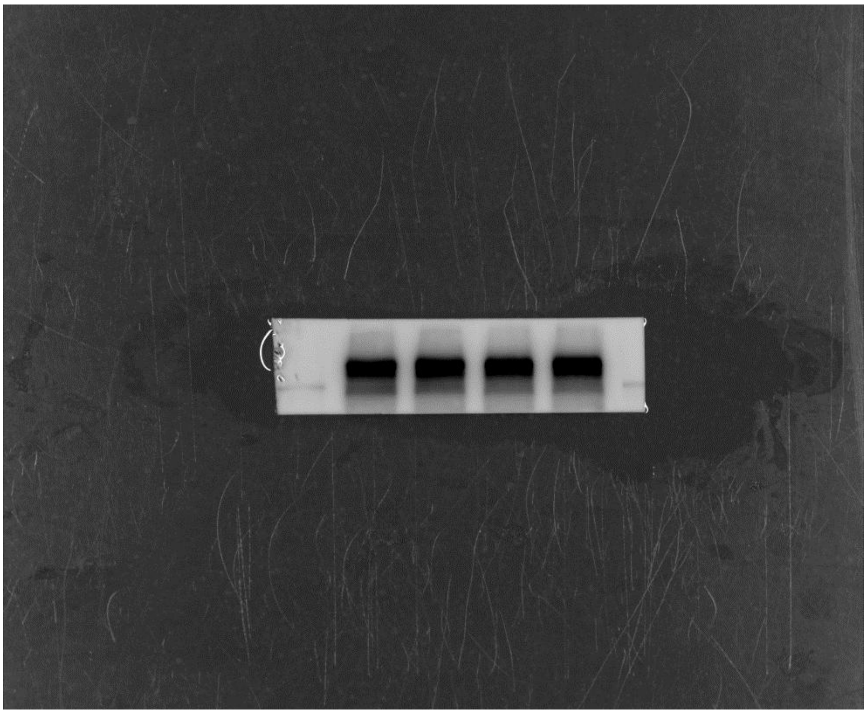

Figure6E

BLM-K24la

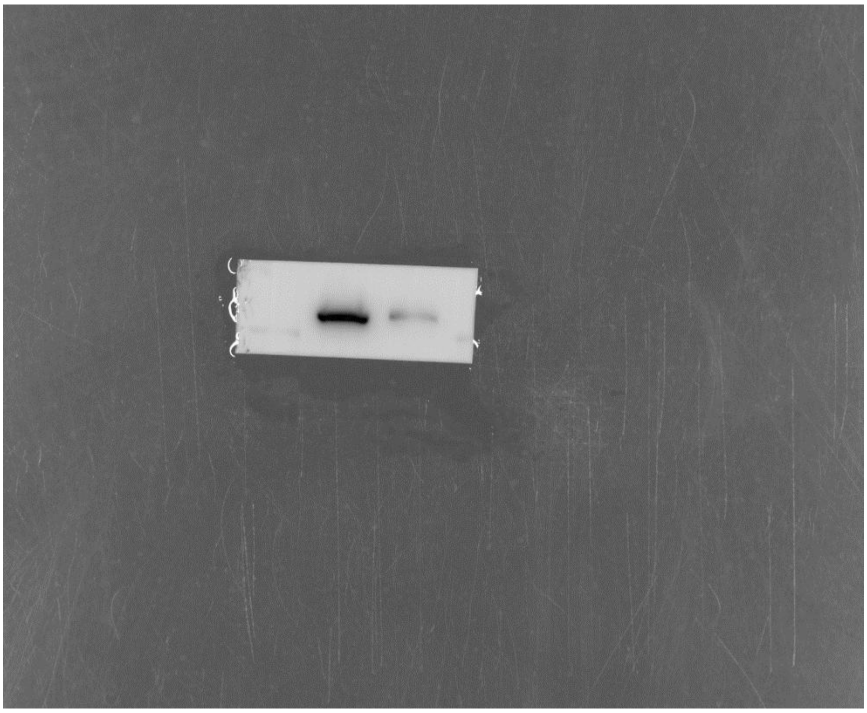

β-actin

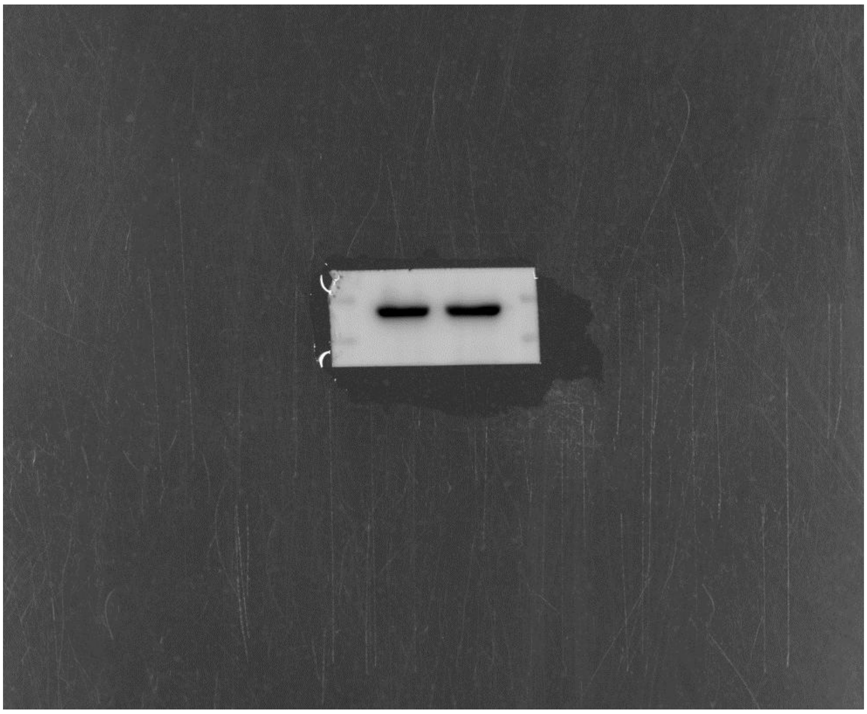

Figure6F

BLM-K24la

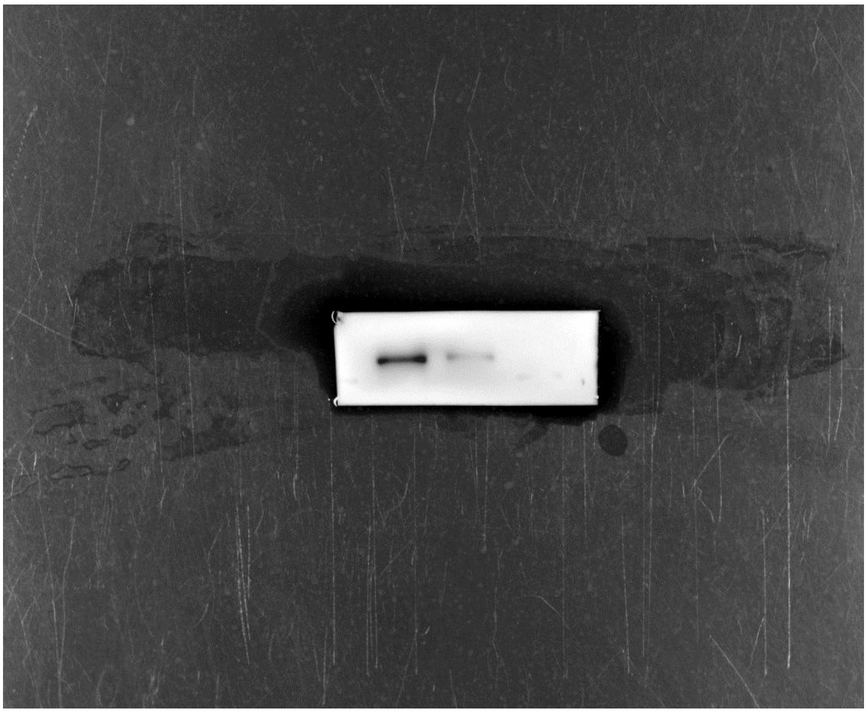

β-actin

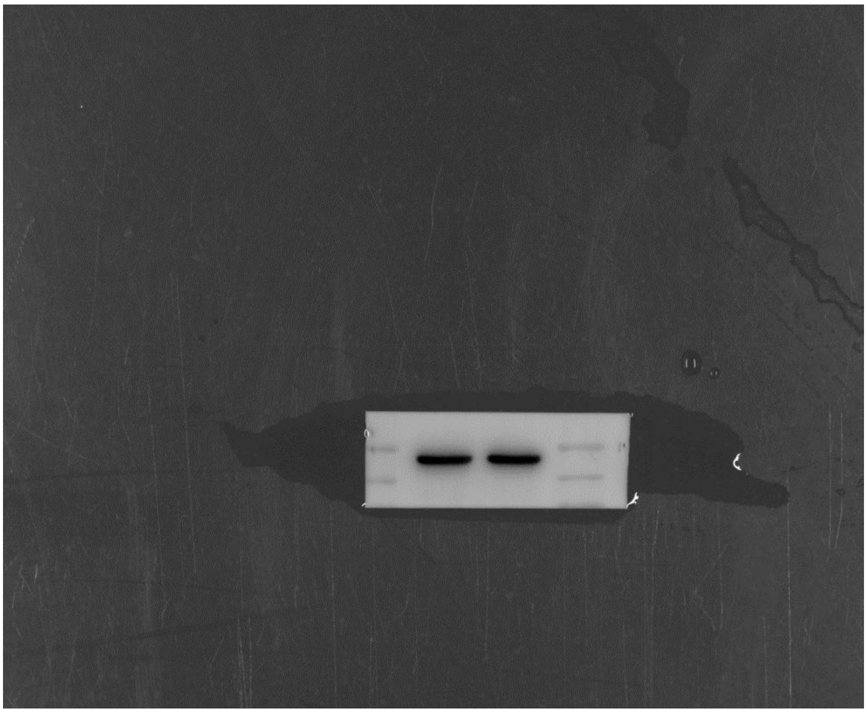

Figure6M

IP-HA

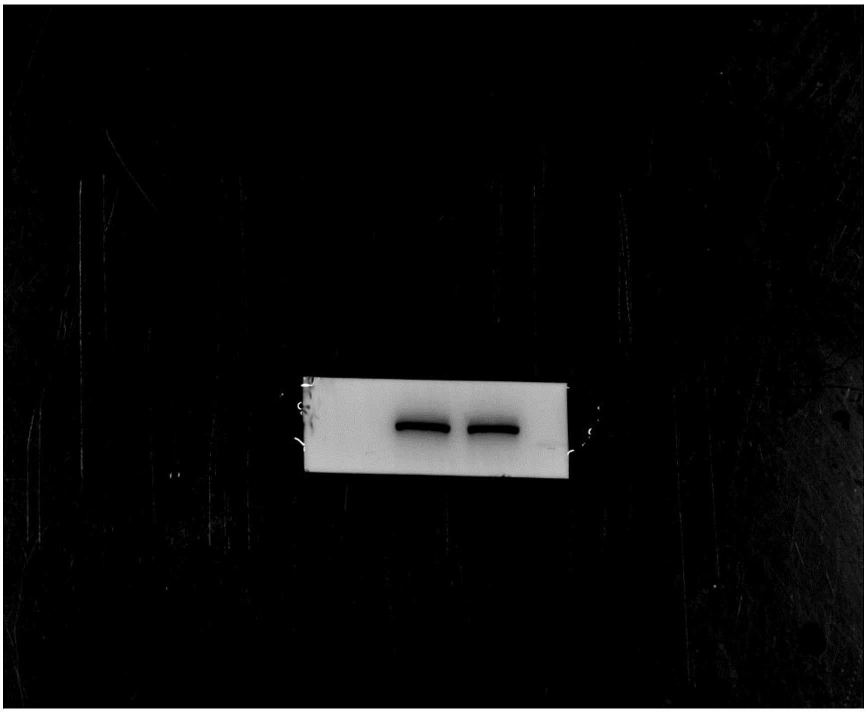

IP-Flag

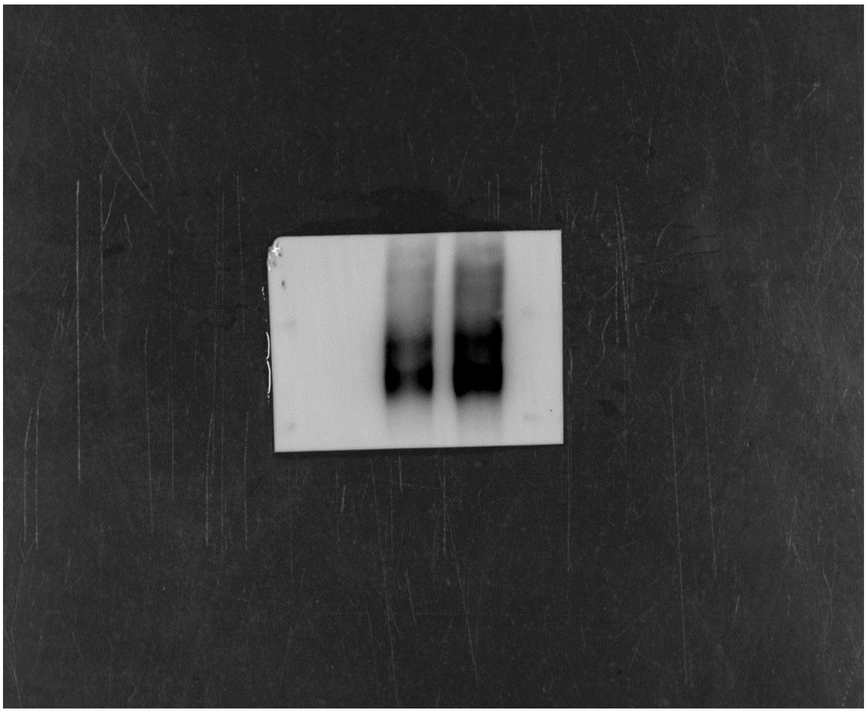

IP-His

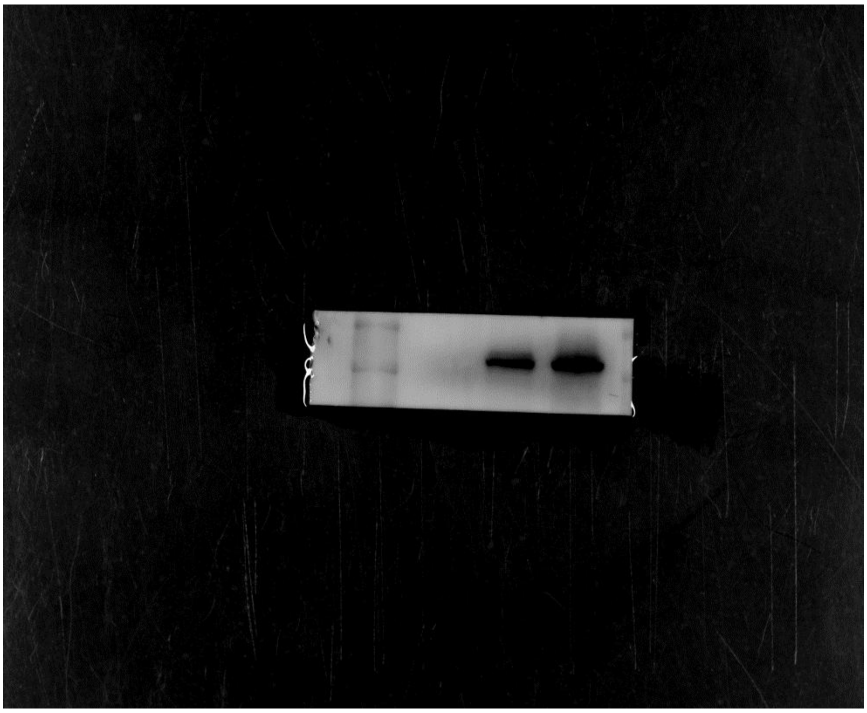

Input-HA

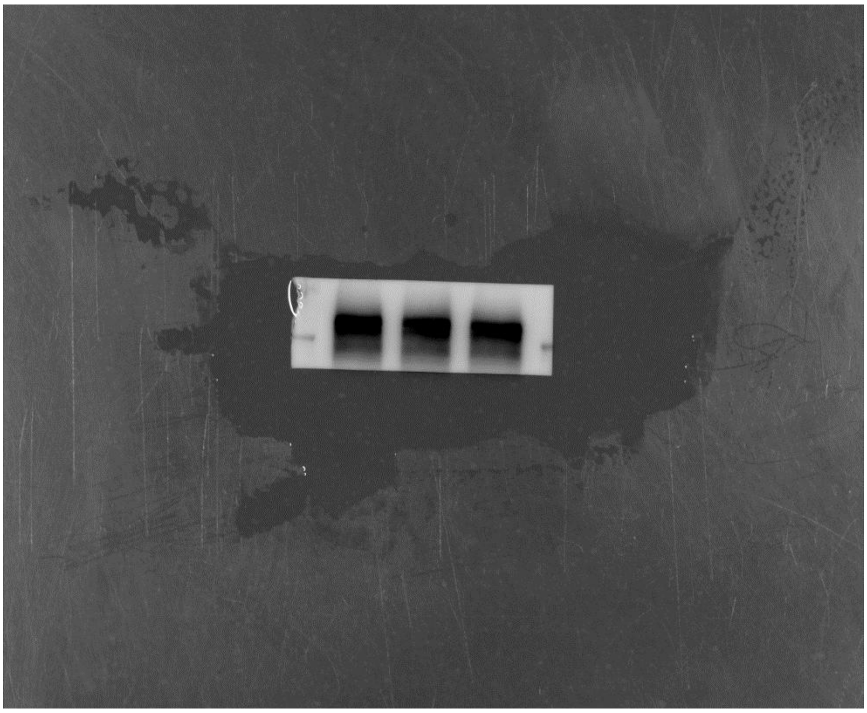

Figure6L

γH2AX

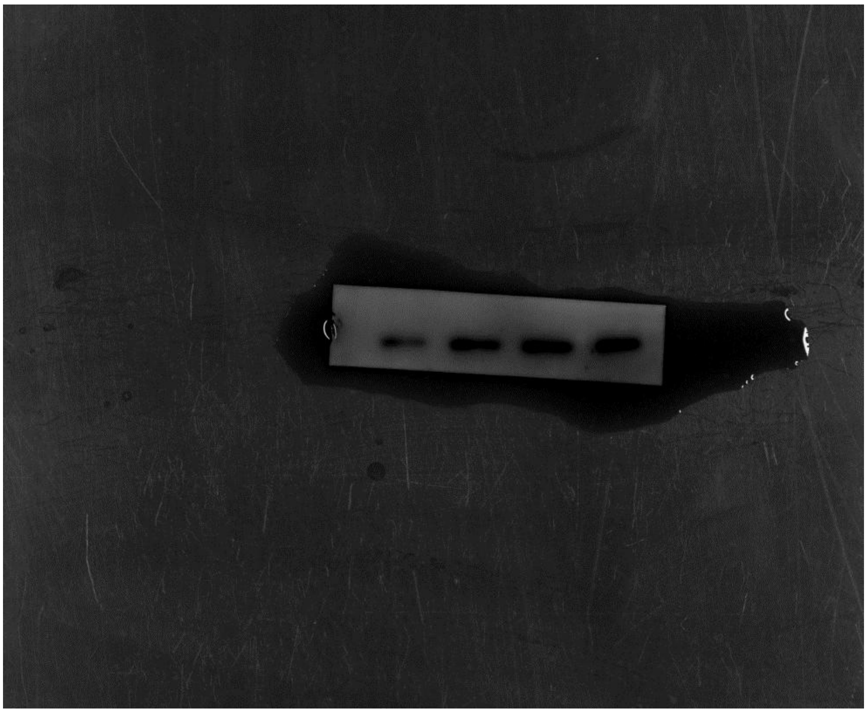

β-actin

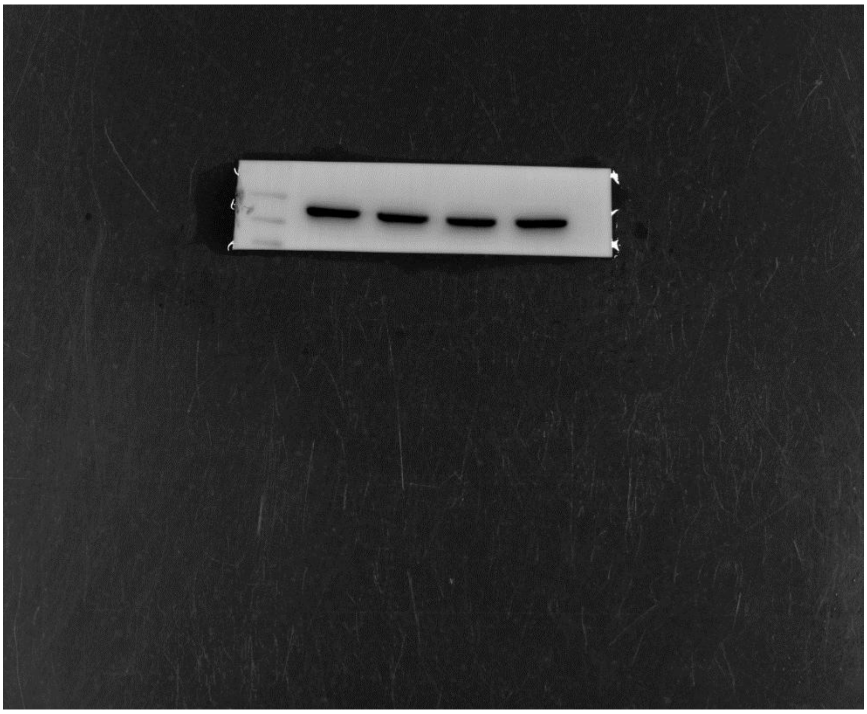

Figure6P

RAD51

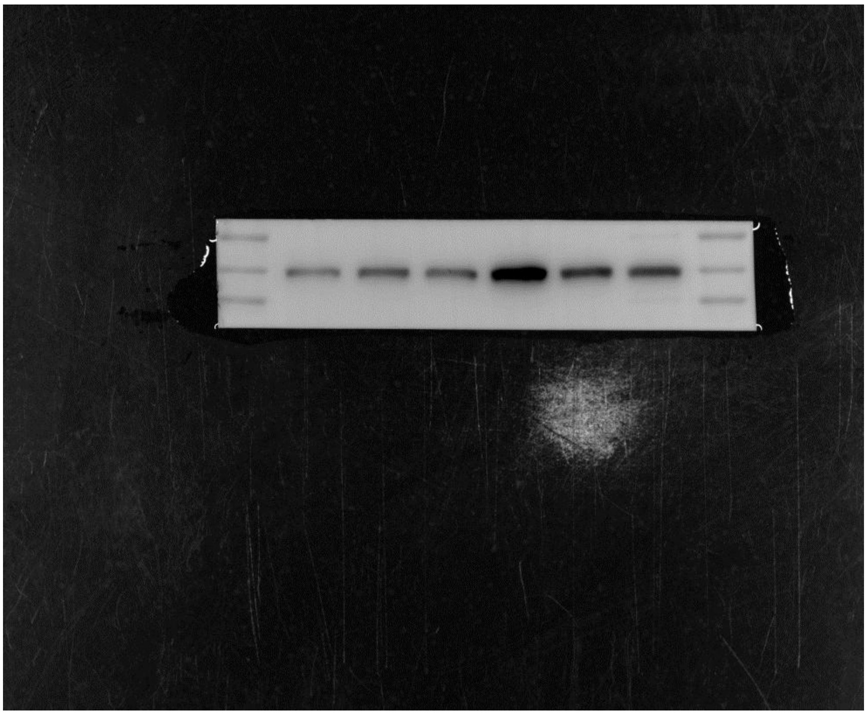

H3

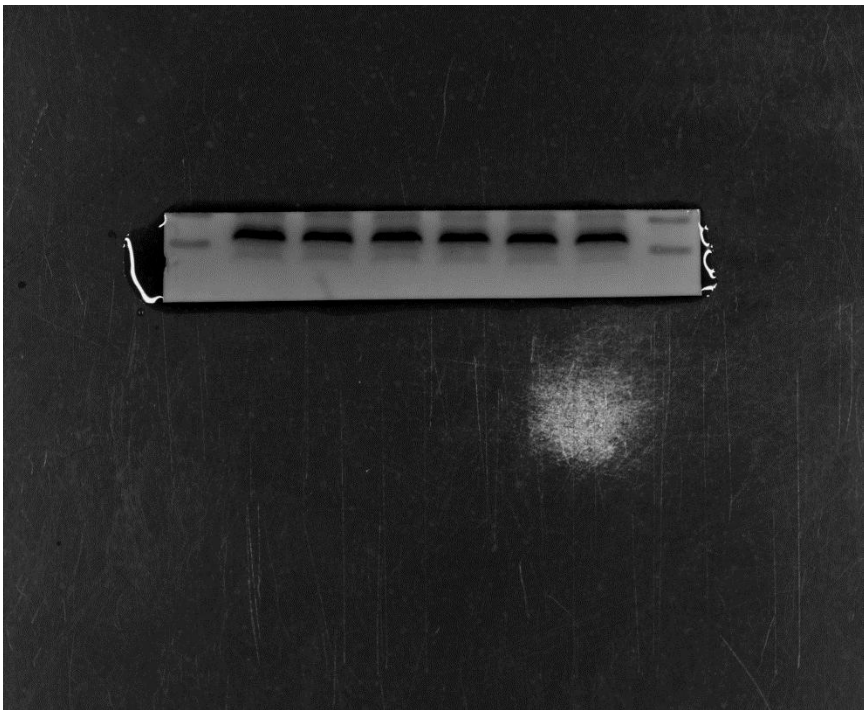

γH2AX

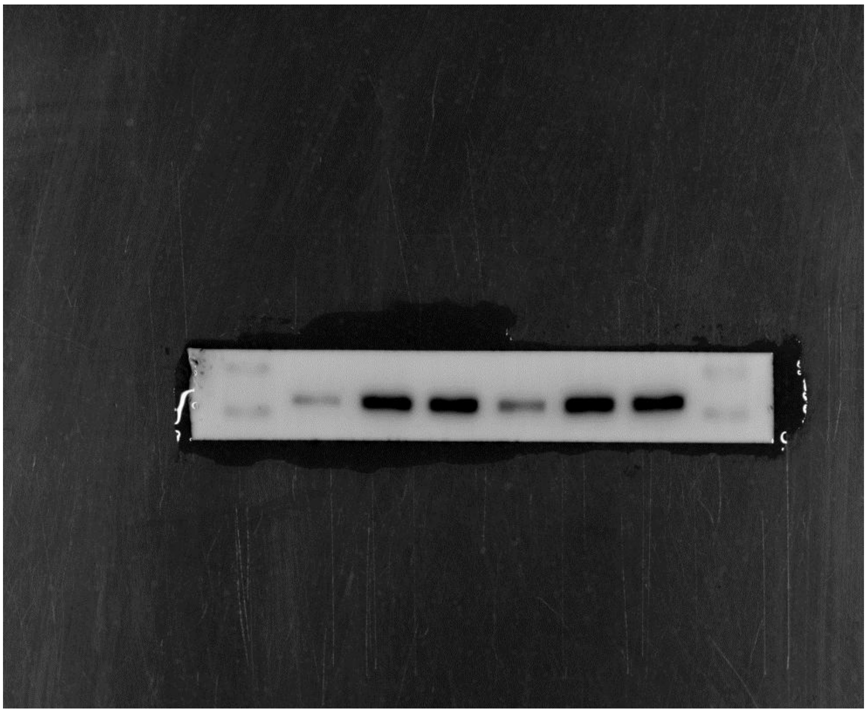

β-actin

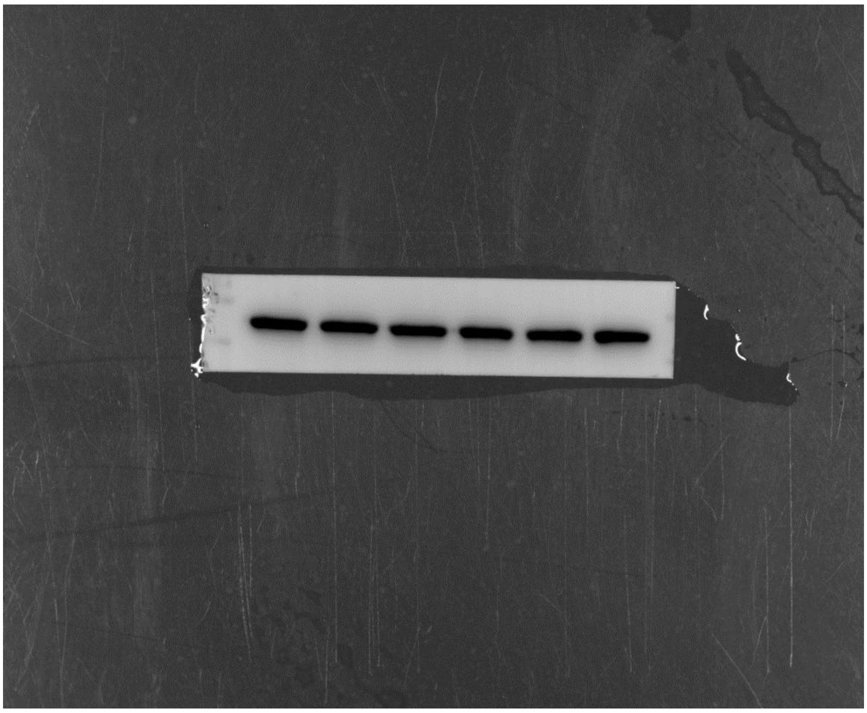

Figure6Q

BLM-K24la

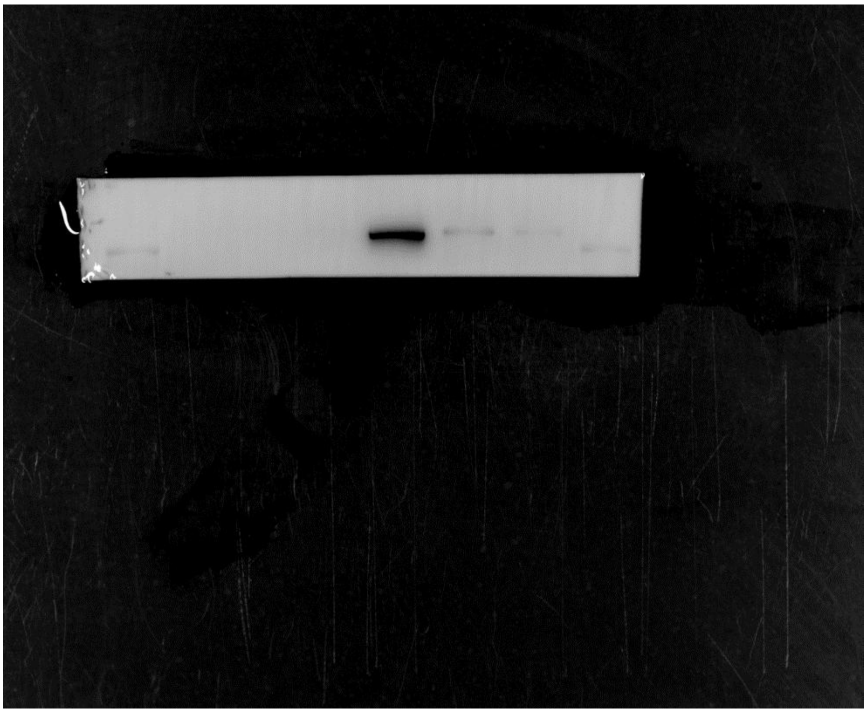

β-actin

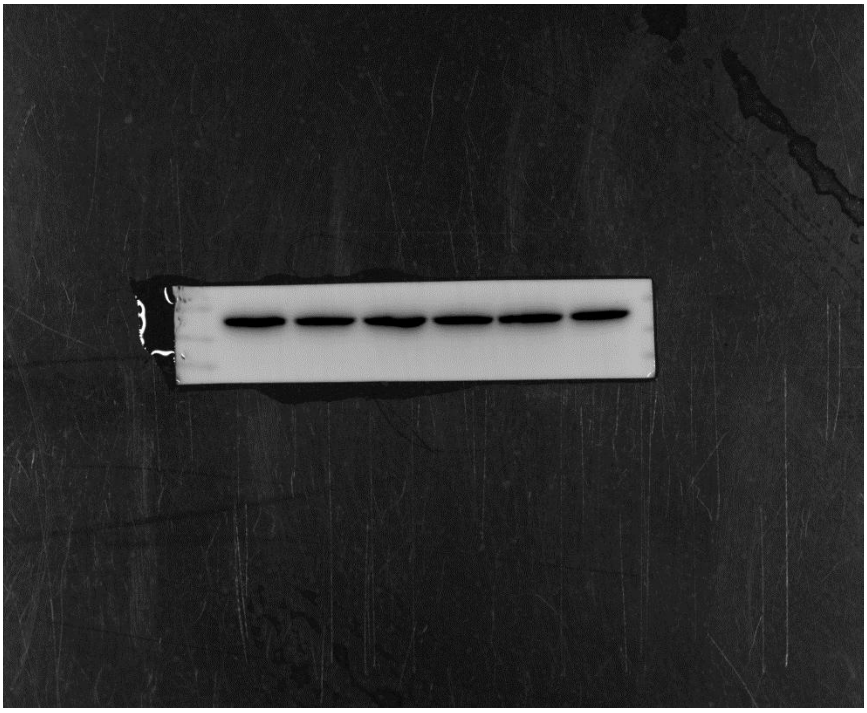

Figure7E

RAD51

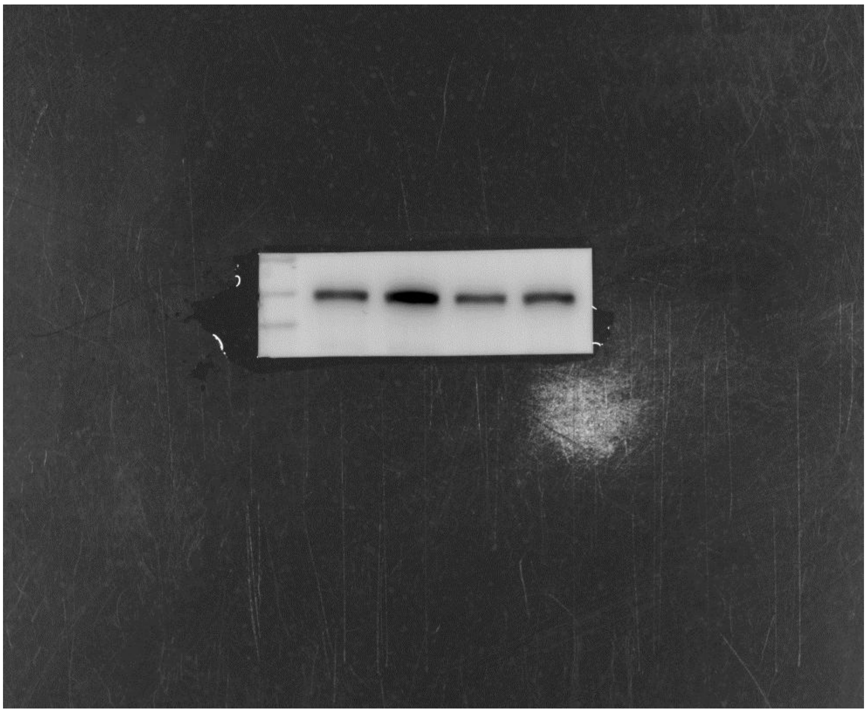

H3

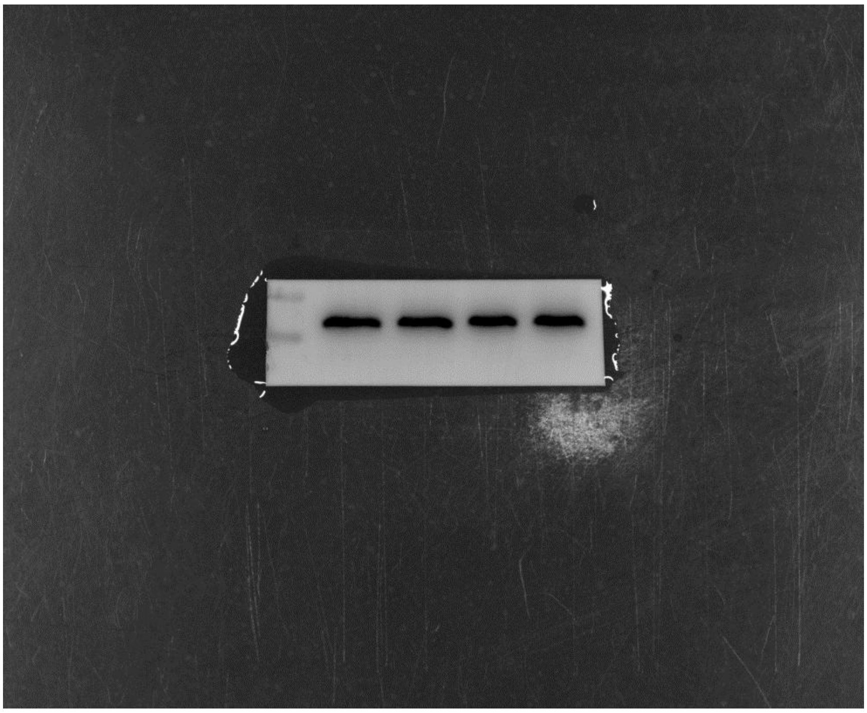

γH2AX

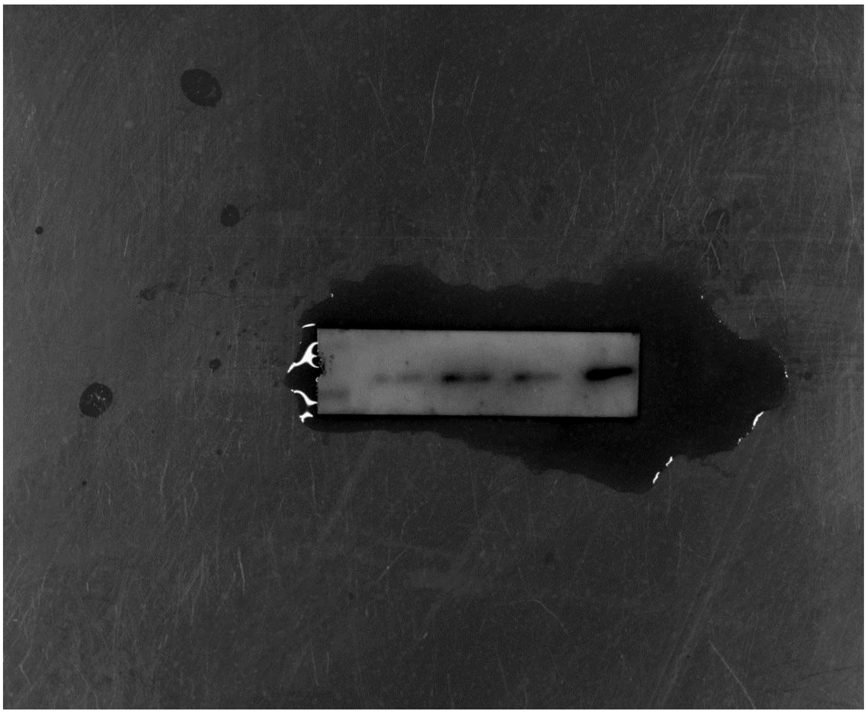

β-actin

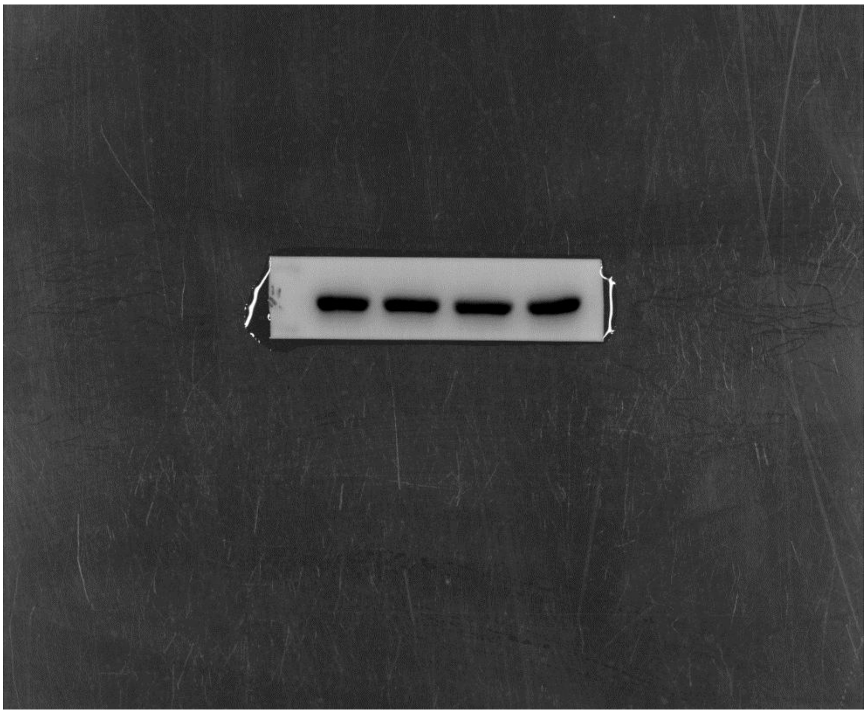

Figure7F

IP-BLM

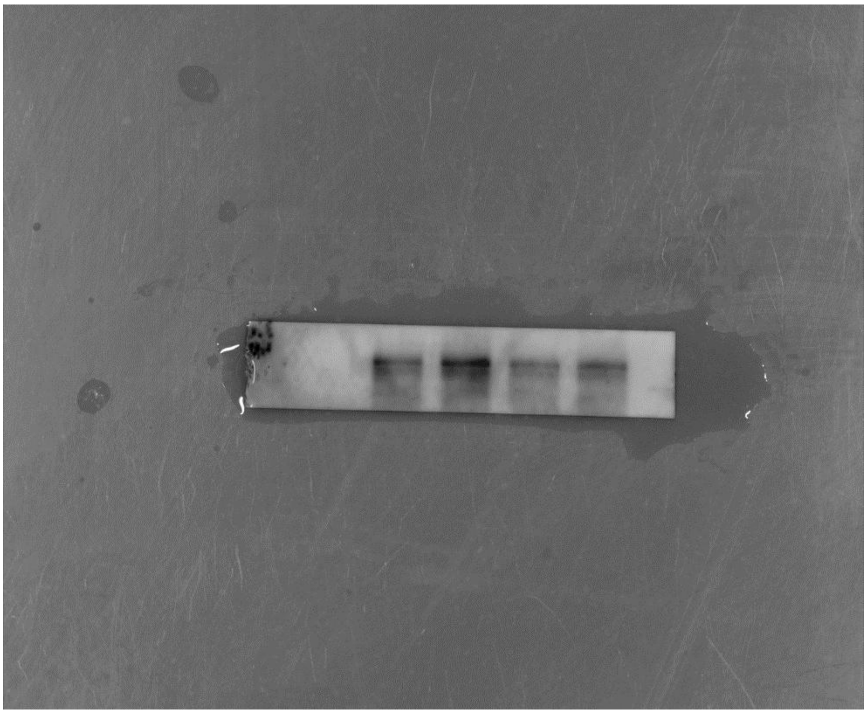

Input-BLM

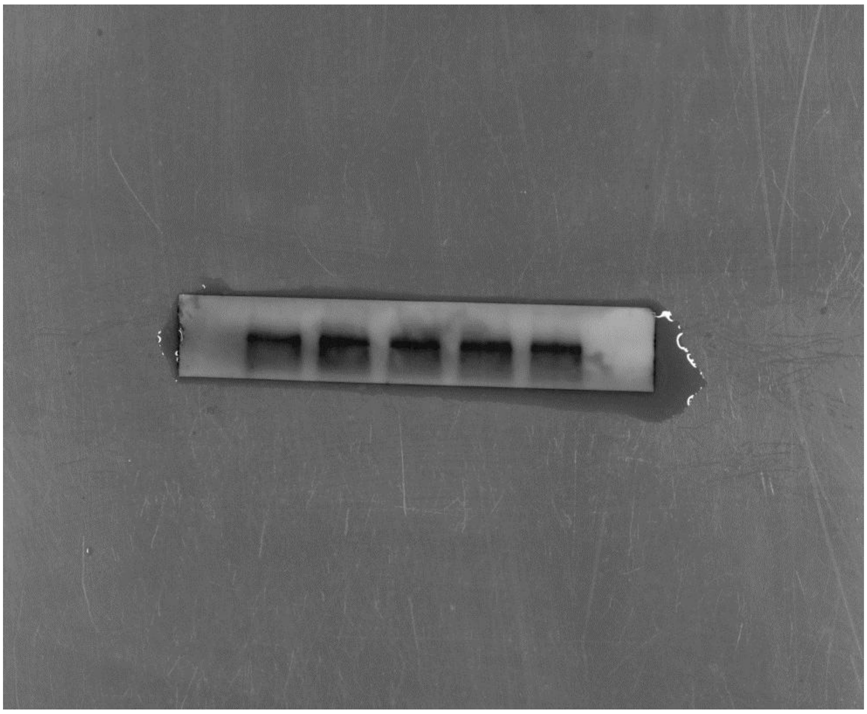

Figure7L

RAD51

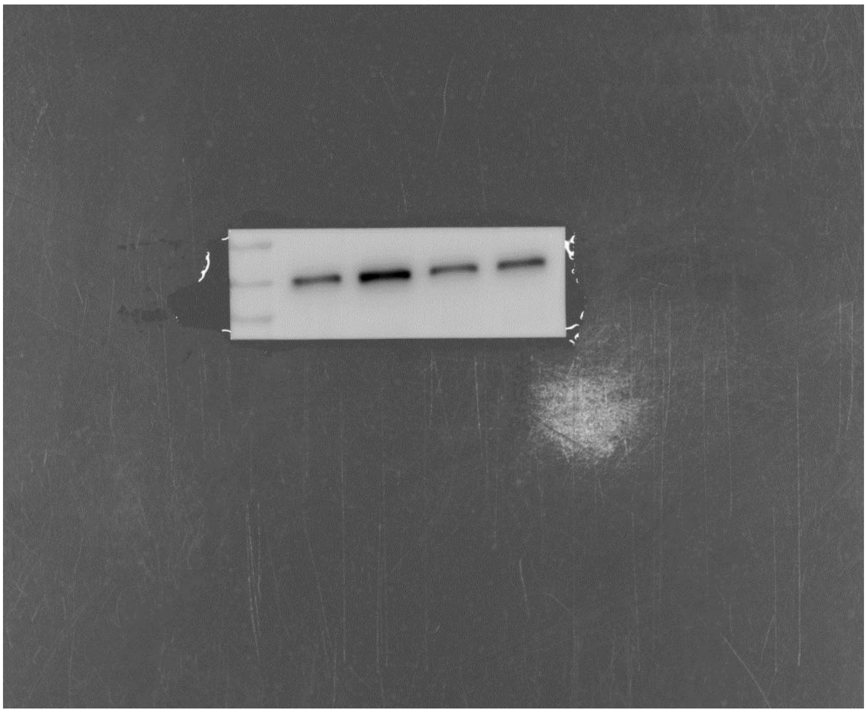

H3

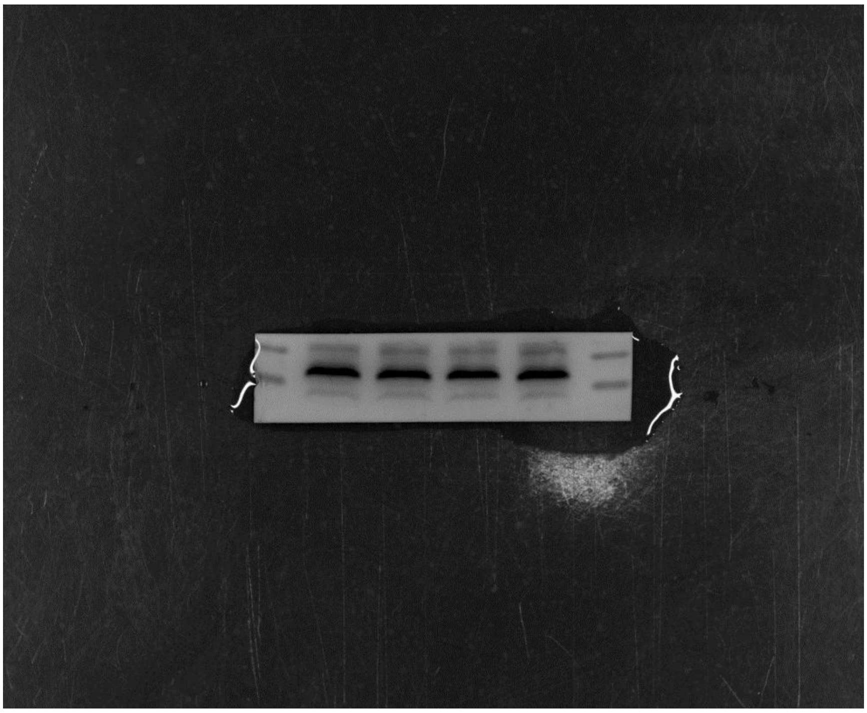

γH2AX

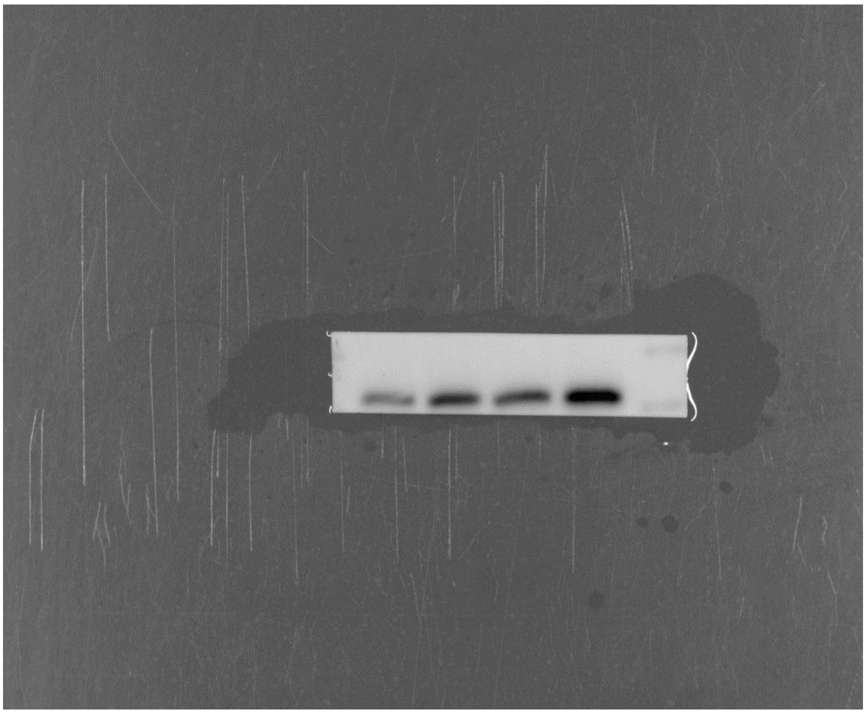

β-actin

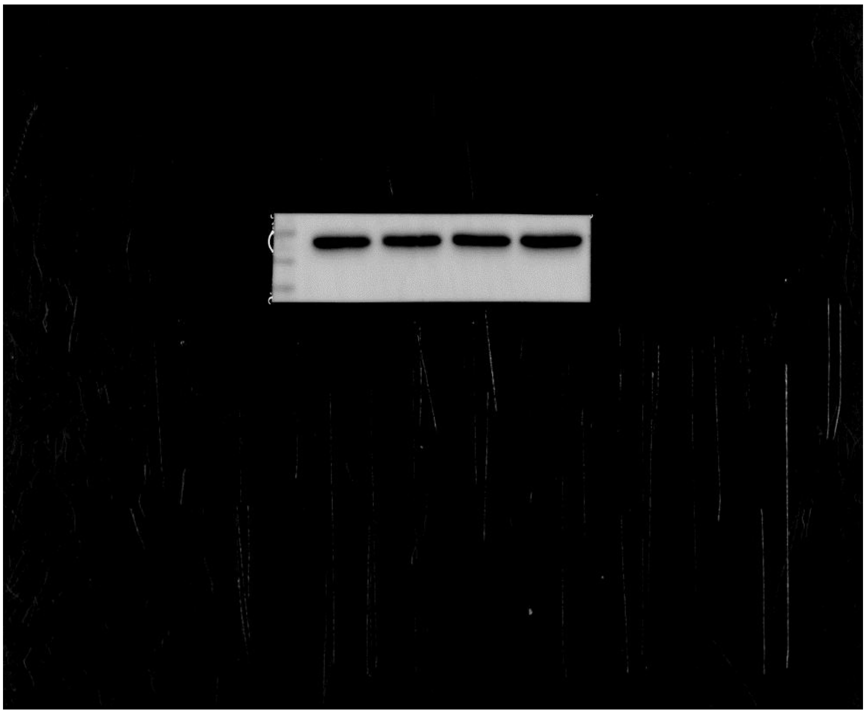

Figure7M

IP-BLM

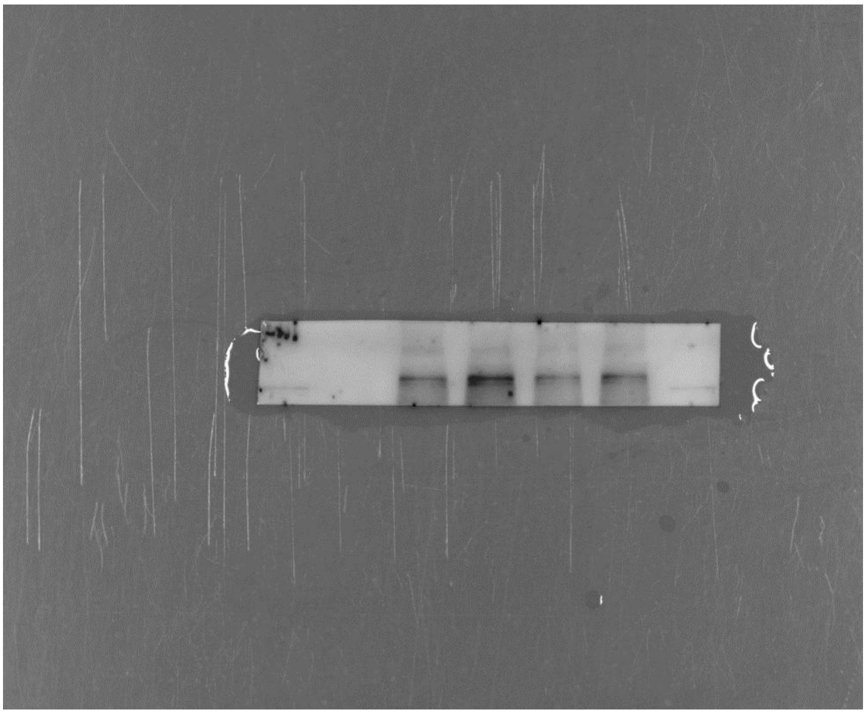

Input-BLM

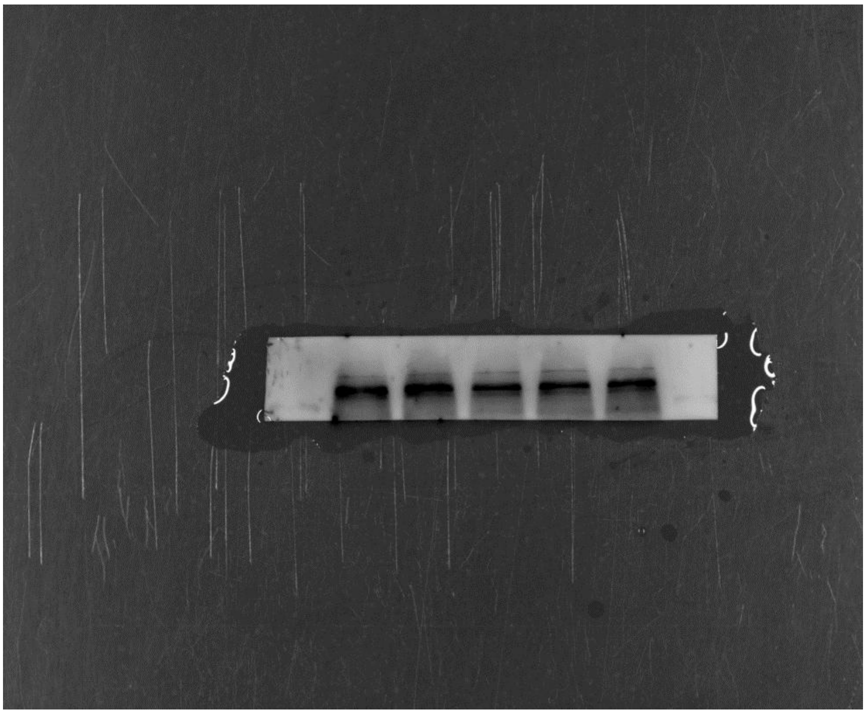

FigureS1B

LDHA

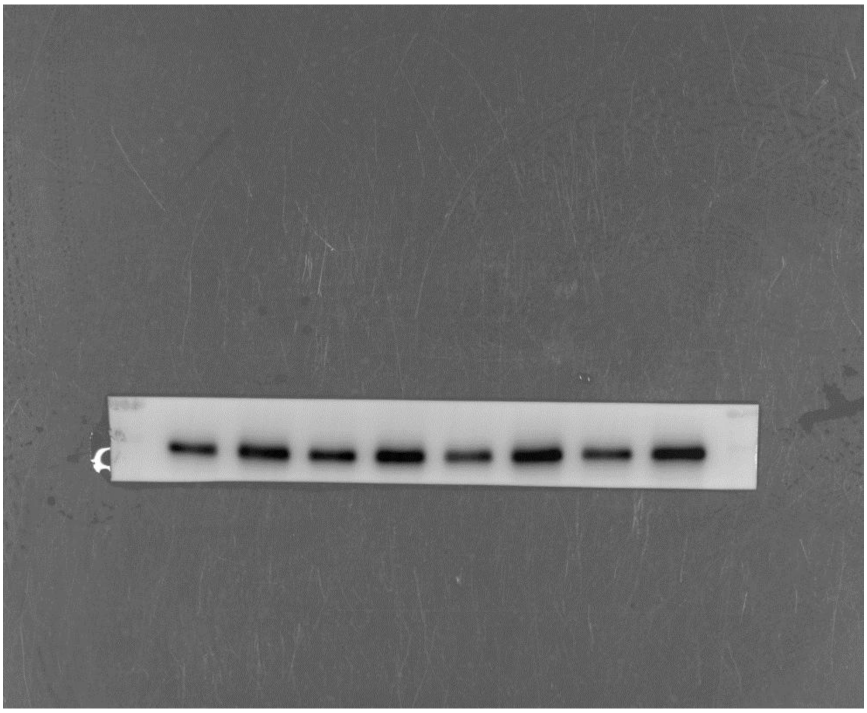

Pan-Kla

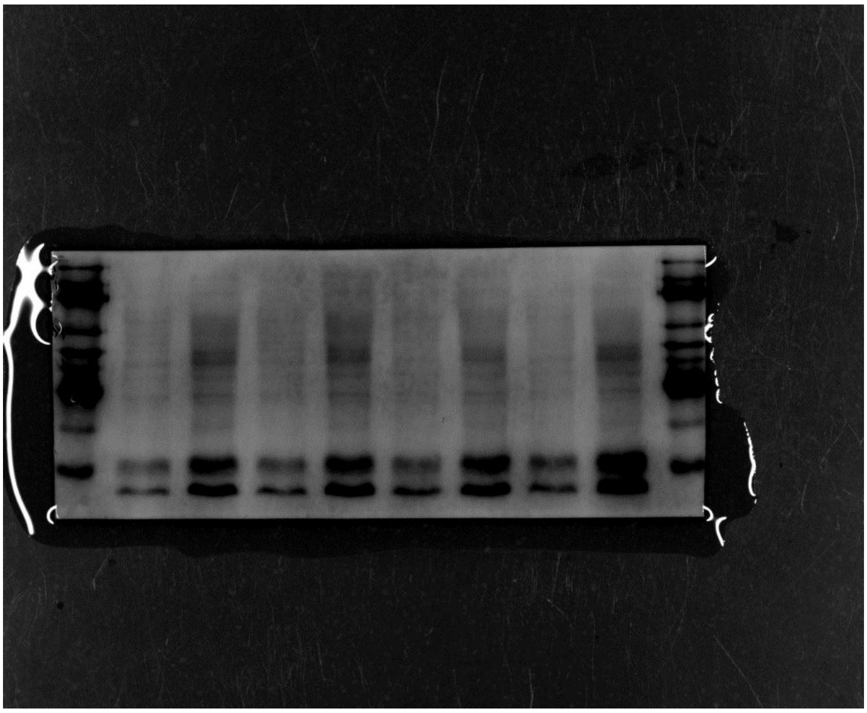

β-actin

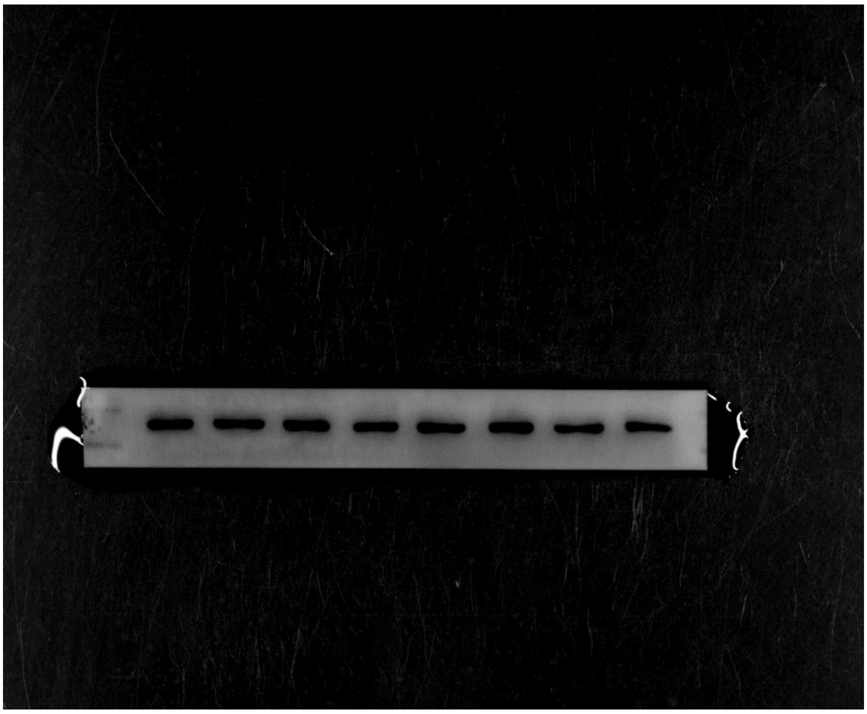

FigureS1C

LDHA

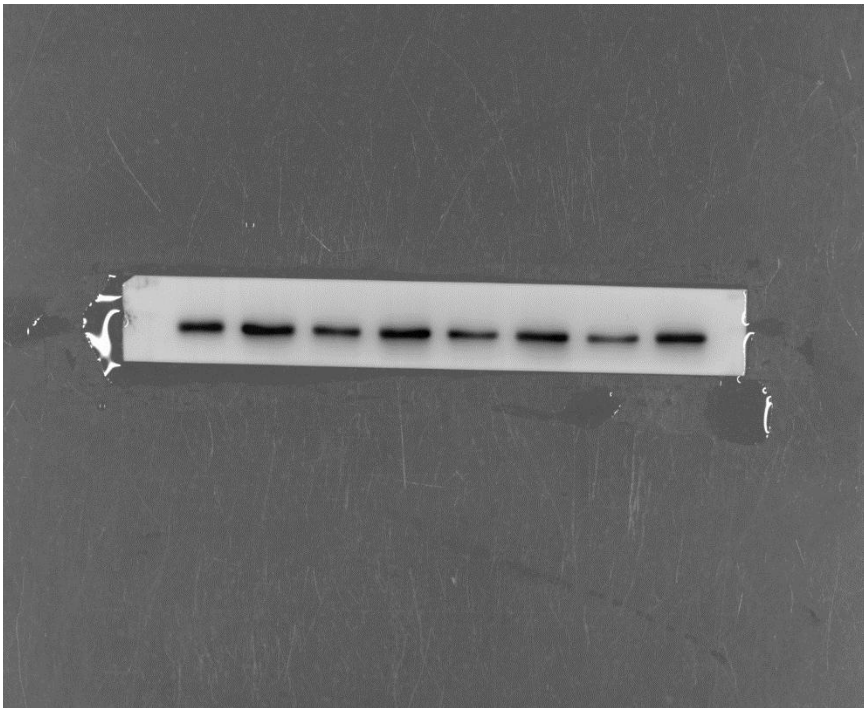

Pan-Kla

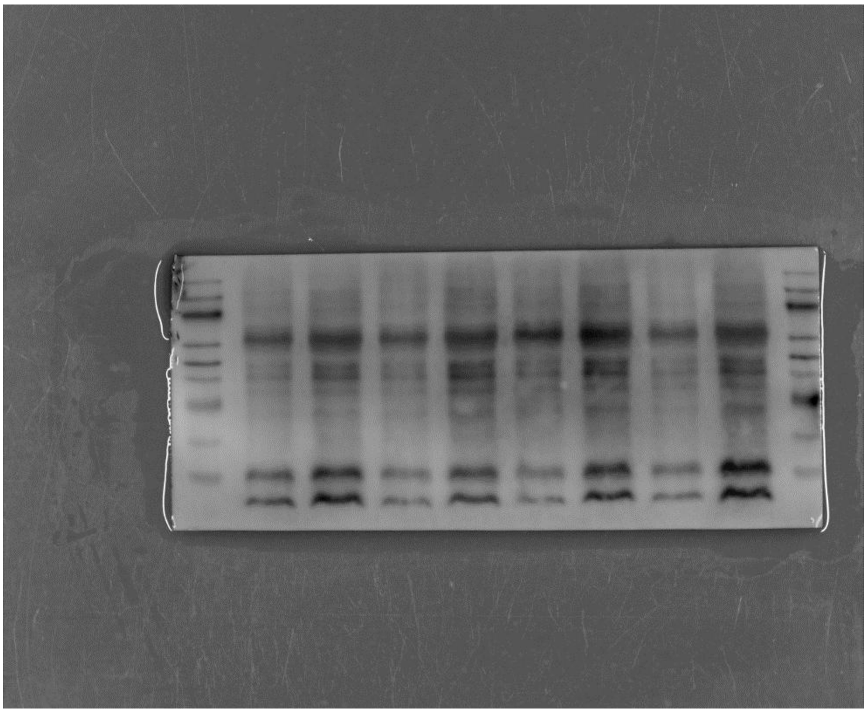

β-actin

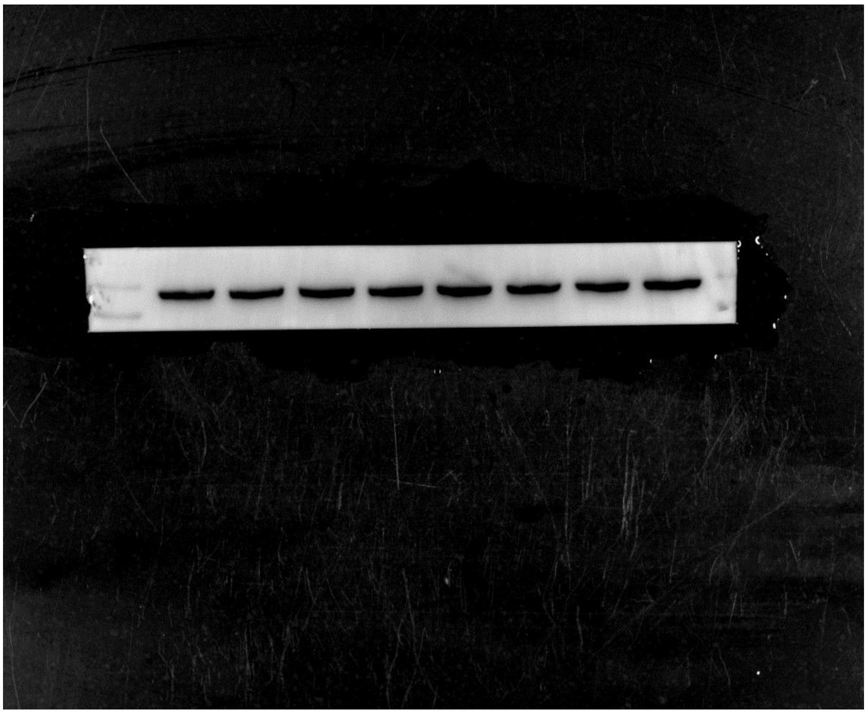

FigureS1G

LDHA

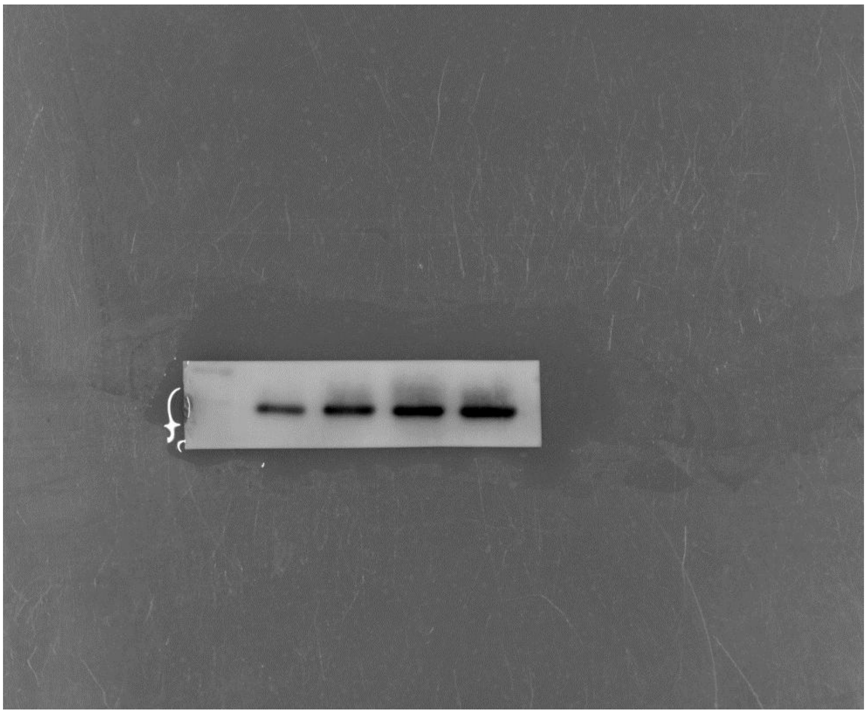

Pan-Kla

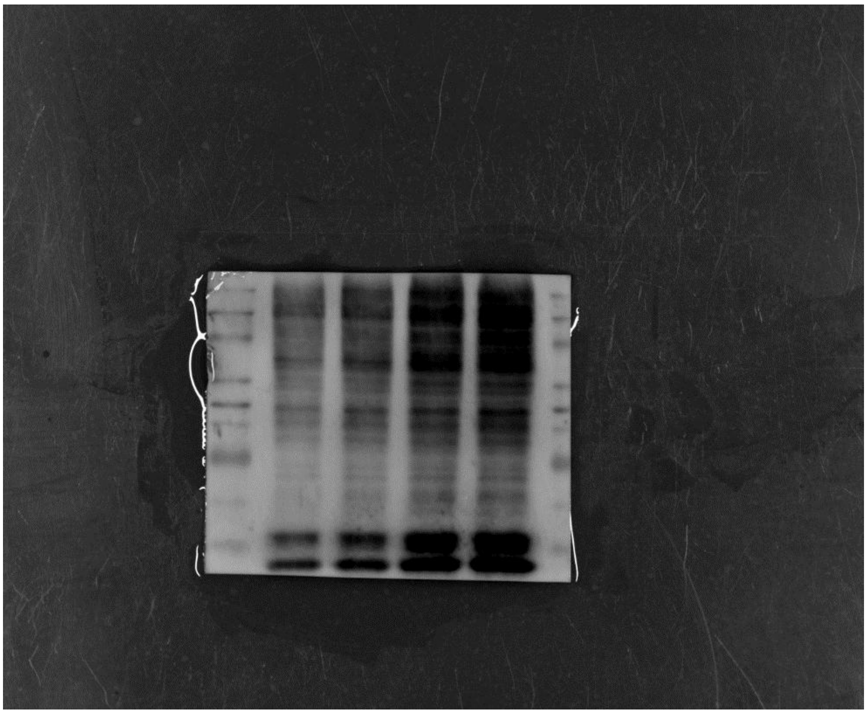

β-actin

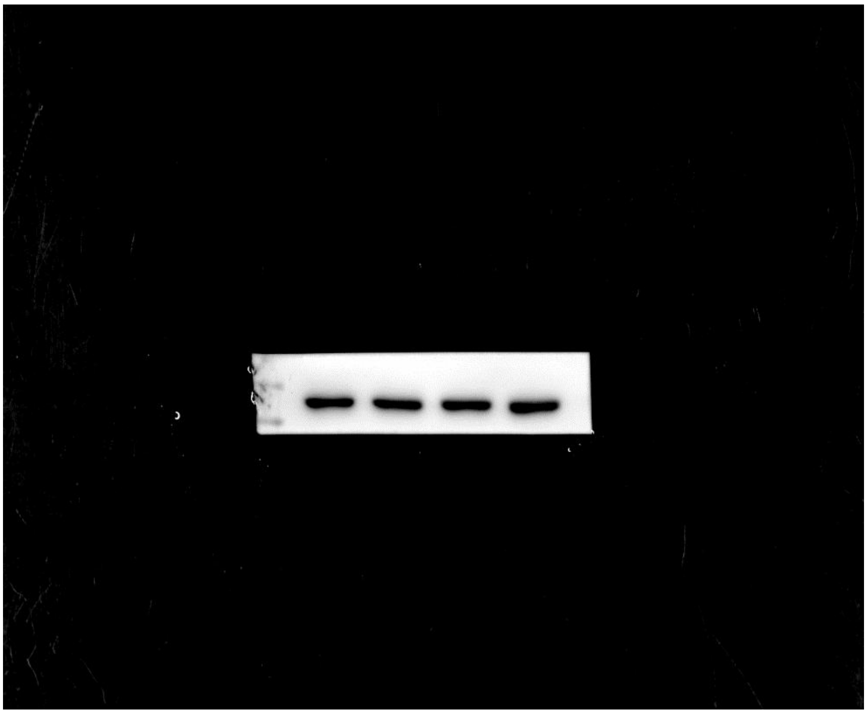

FigureS3E

γH2AX

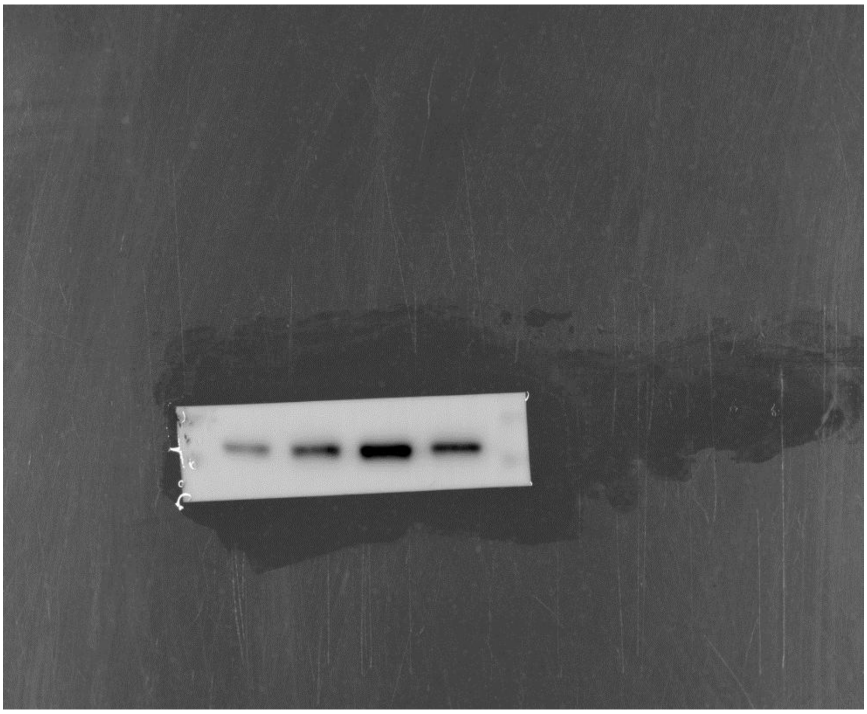

β-actin

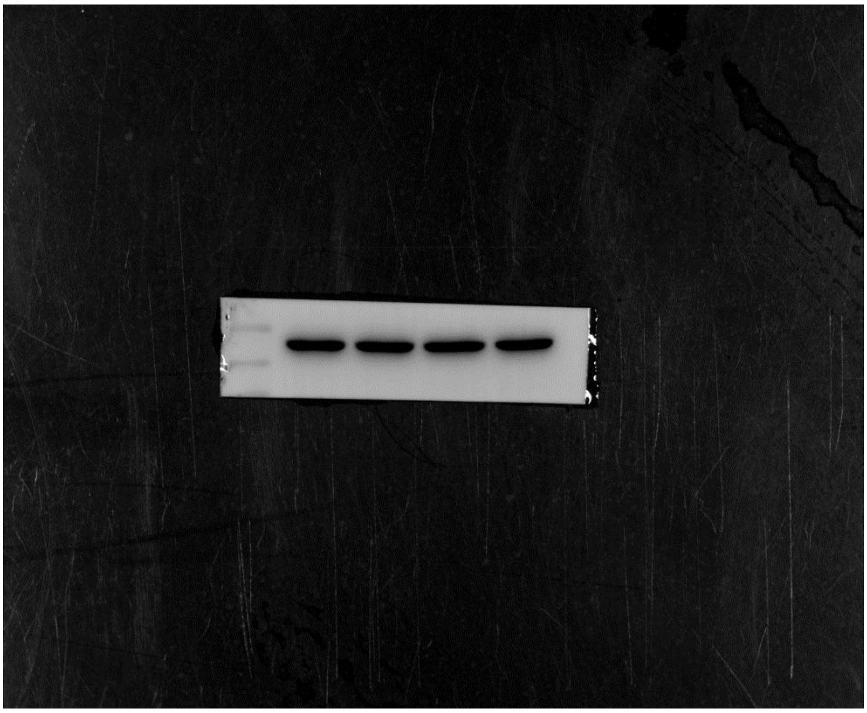

FigureS3F

γH2AX

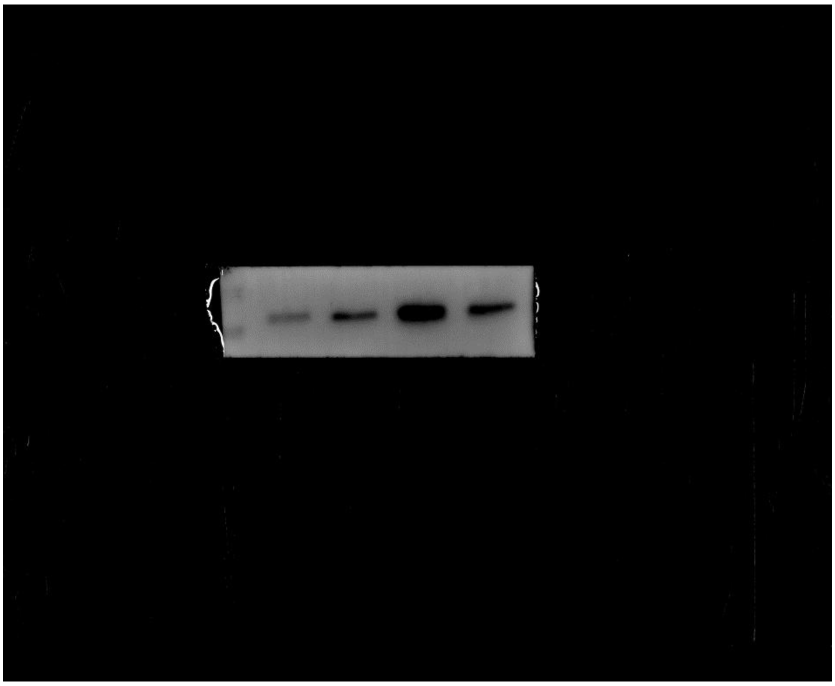

β-actin

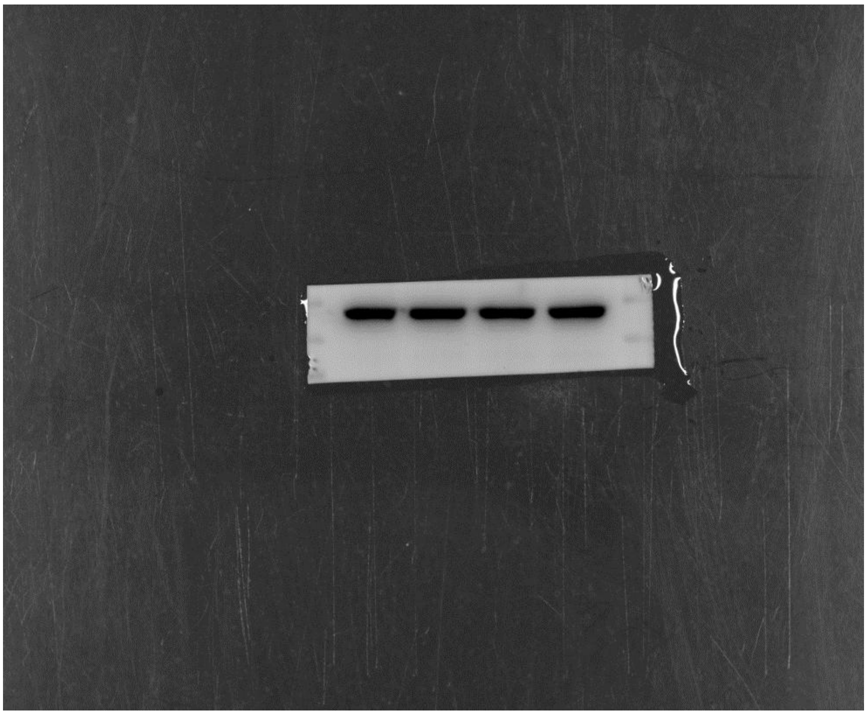

FigureS3G

γH2AX

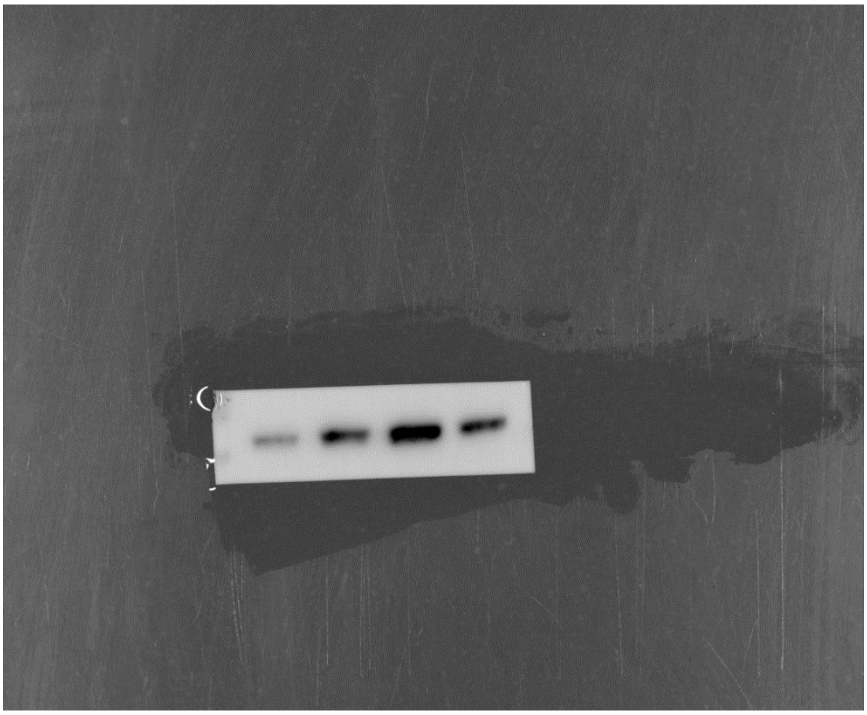

β-actin

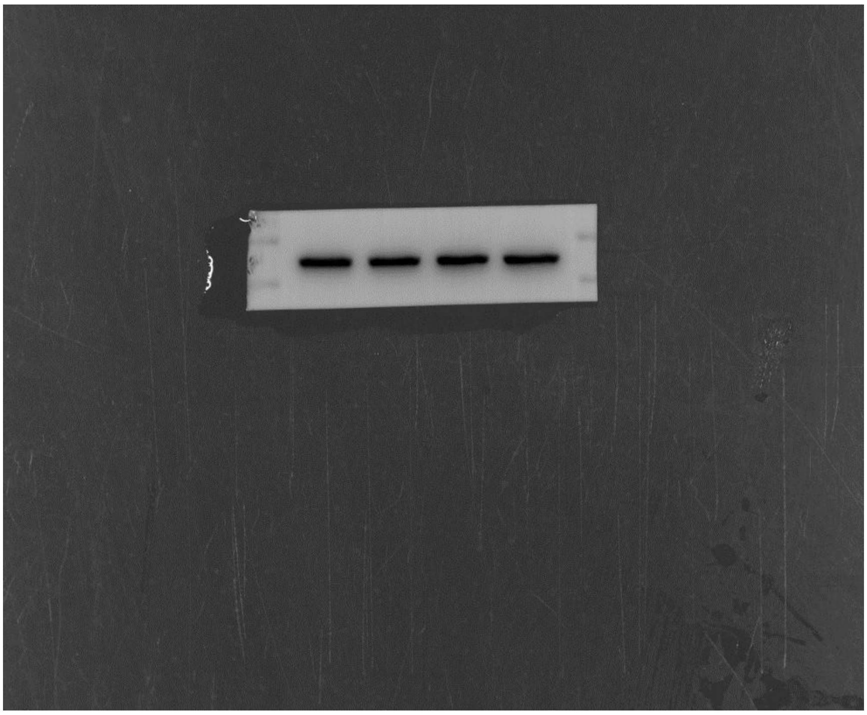

FigureS4A

BLM

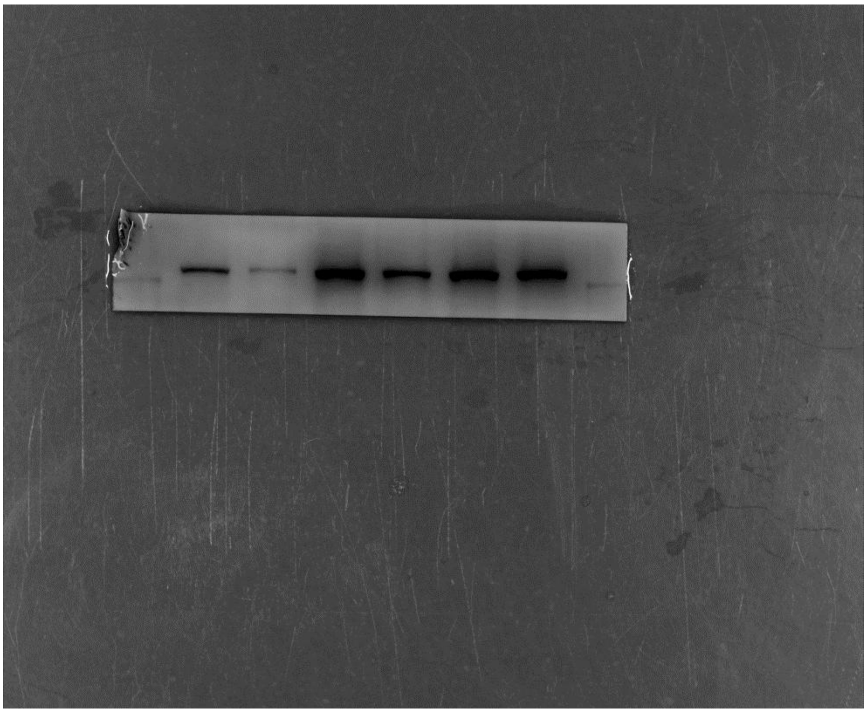

β-actin

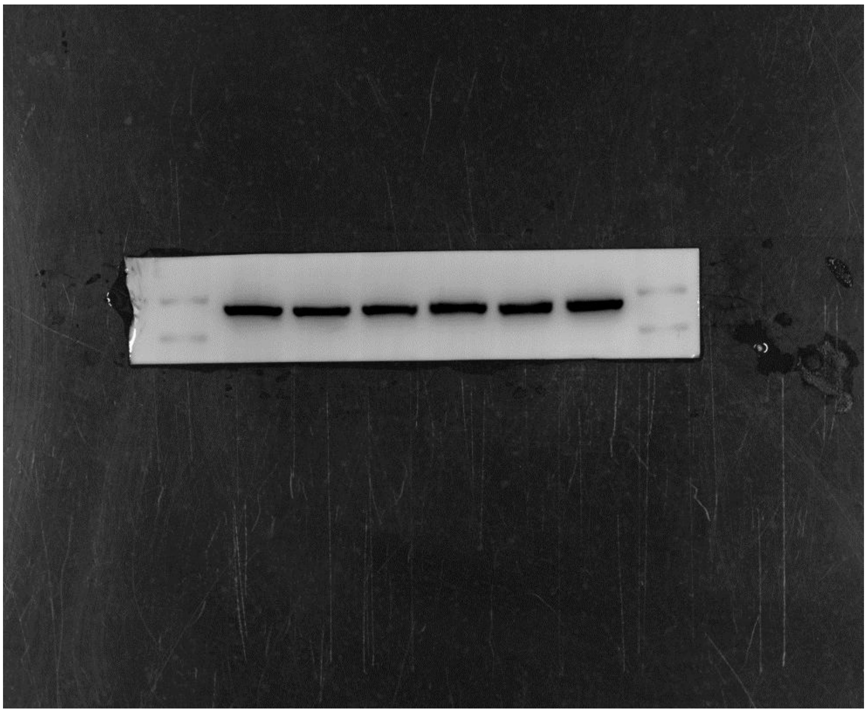

FigureS4D

BLM-K24la

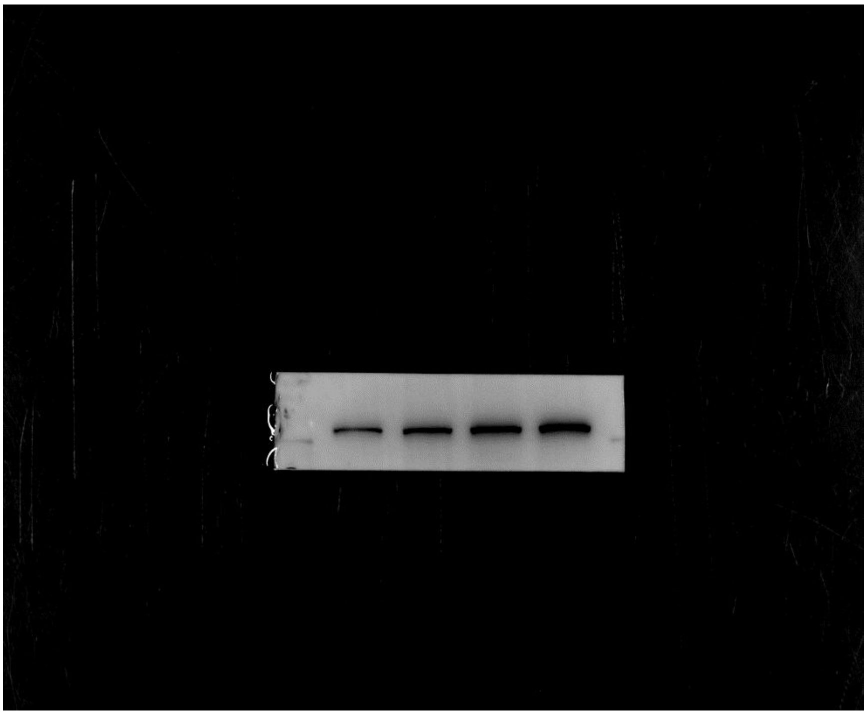

β-actin

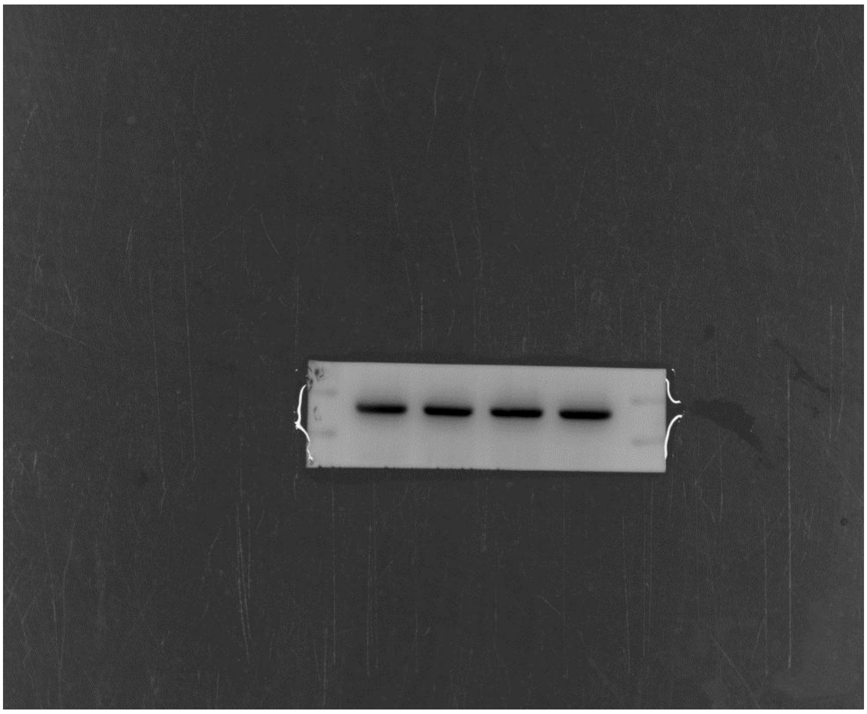

FigureS4E

BLM-K24la

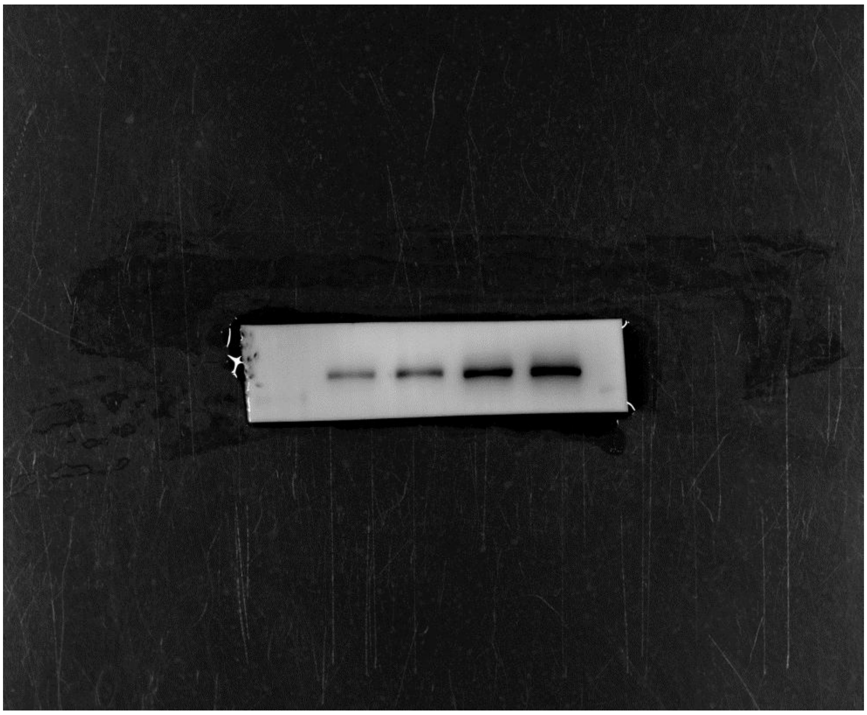

β-actin

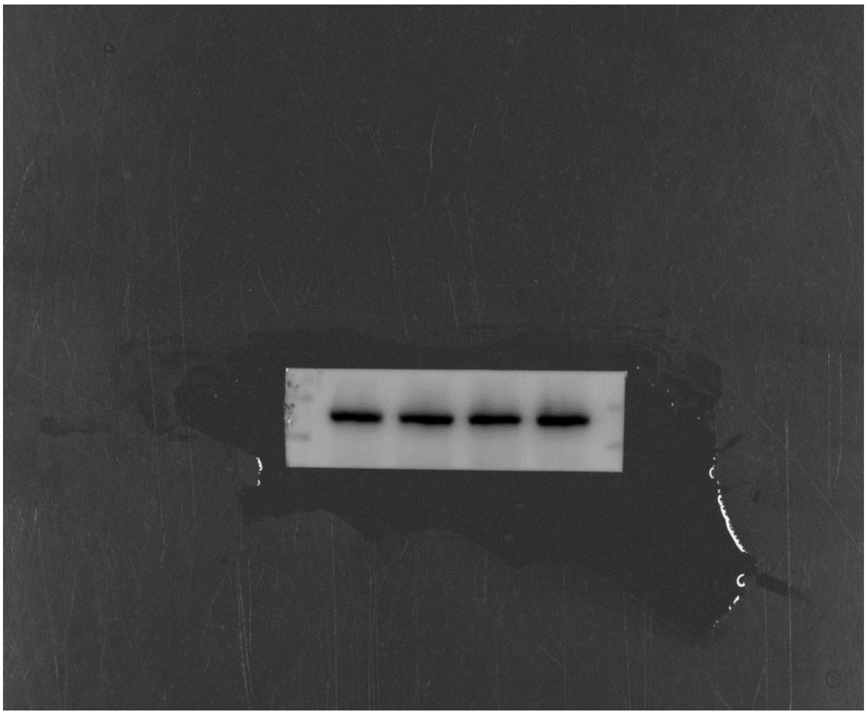

FigureS4F

IP-BLM

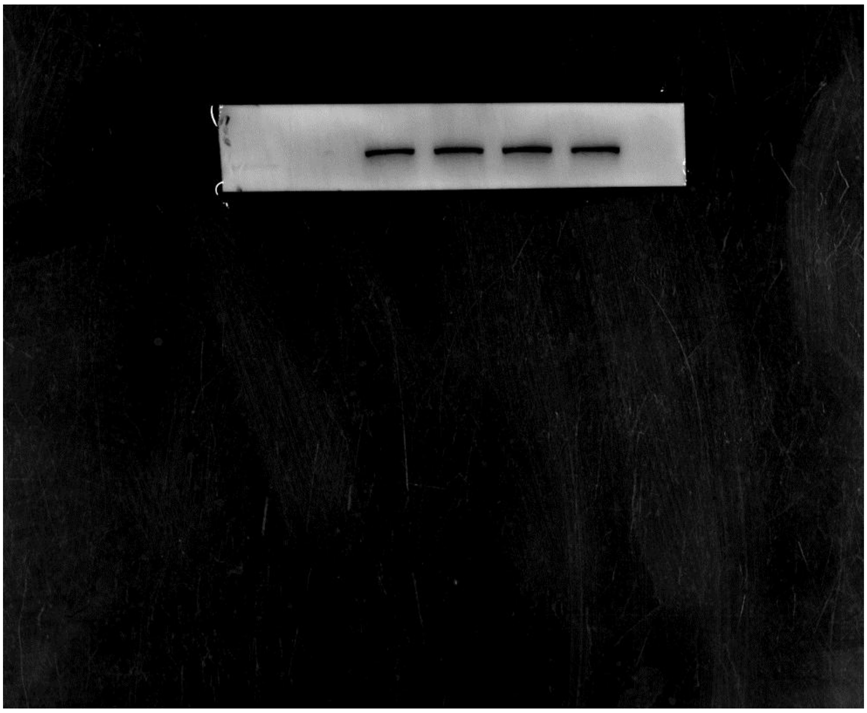

IP-Kla

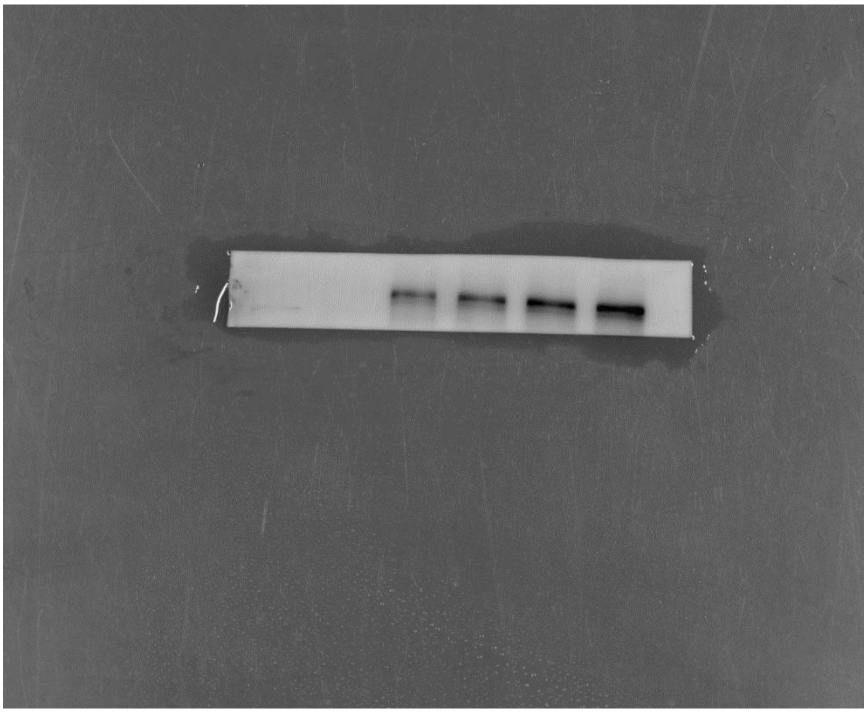

Input-BLM

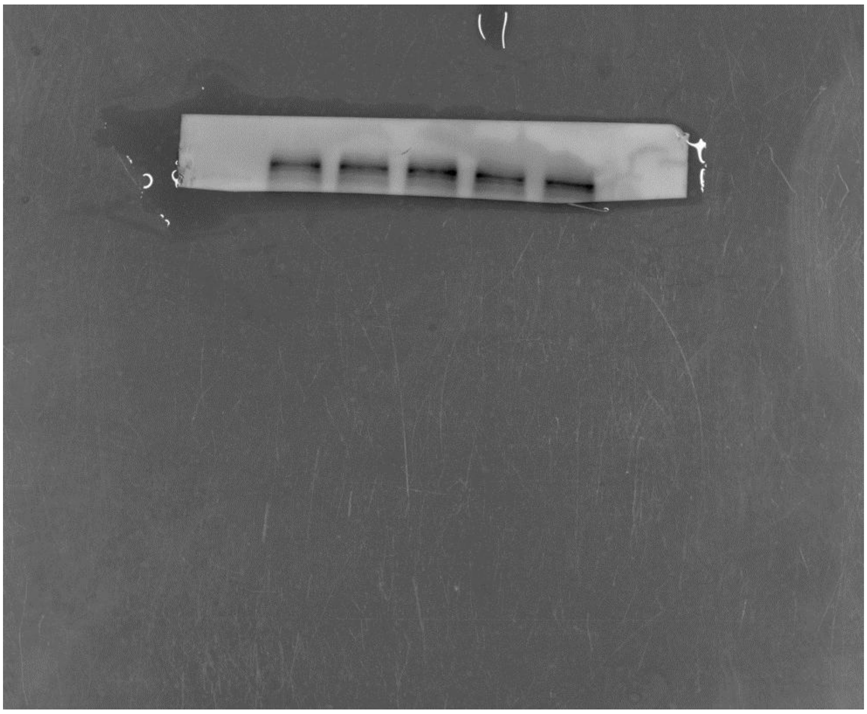

FigureS4G

IP-HA

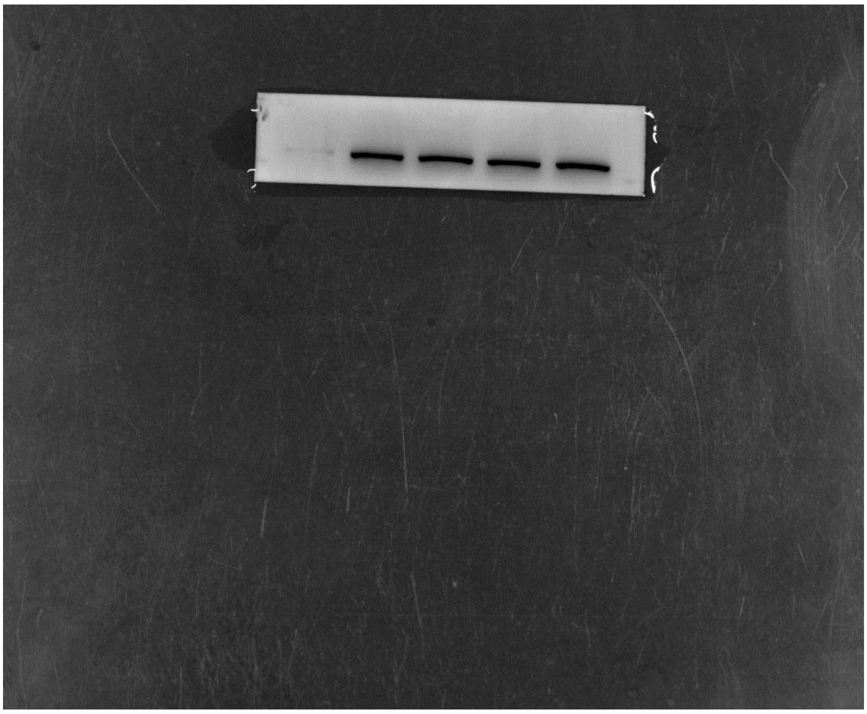

IP-Kla

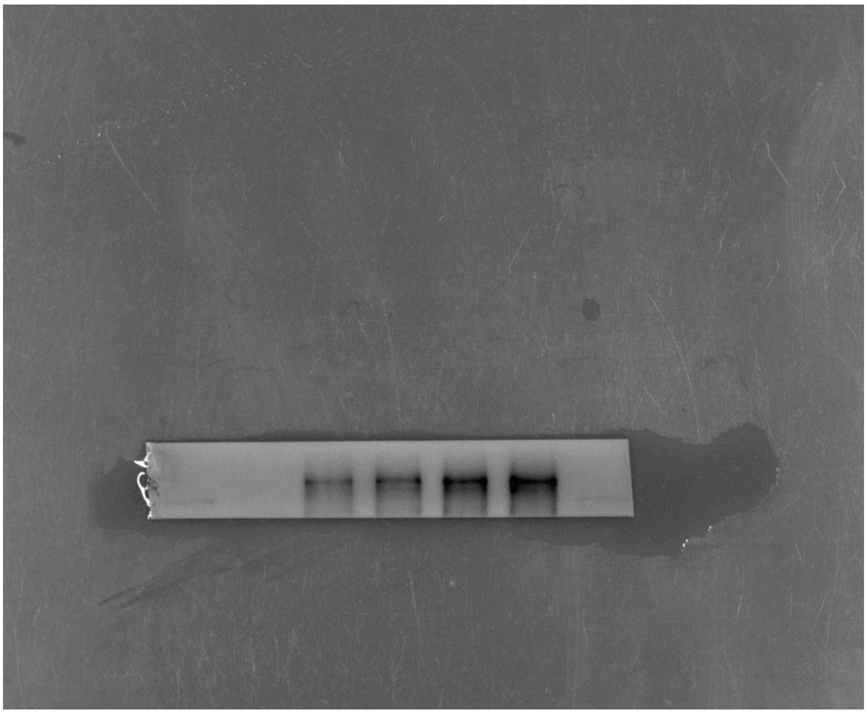

Input-HA

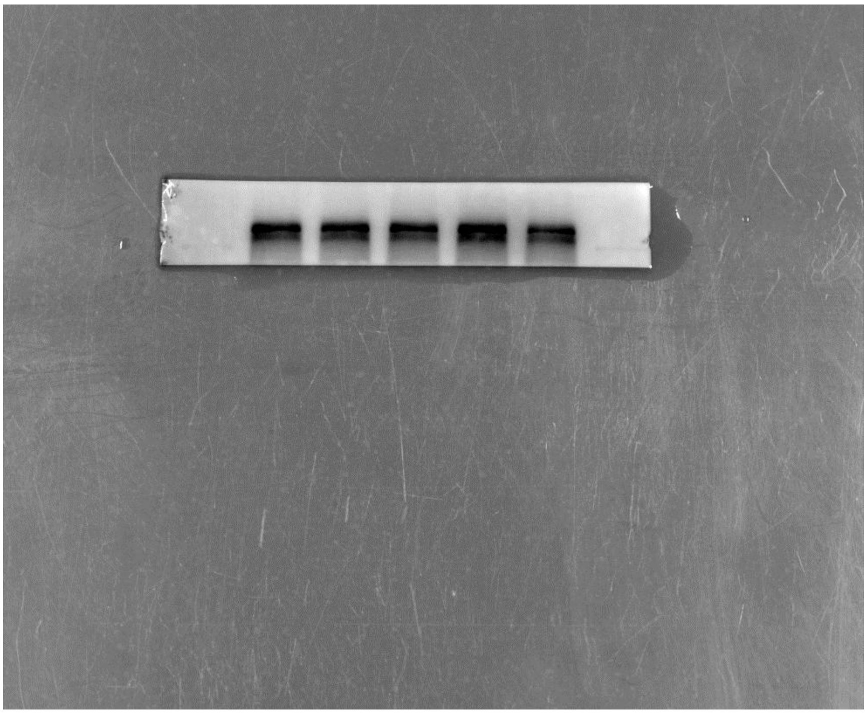

FigureS4H

IP-BLM

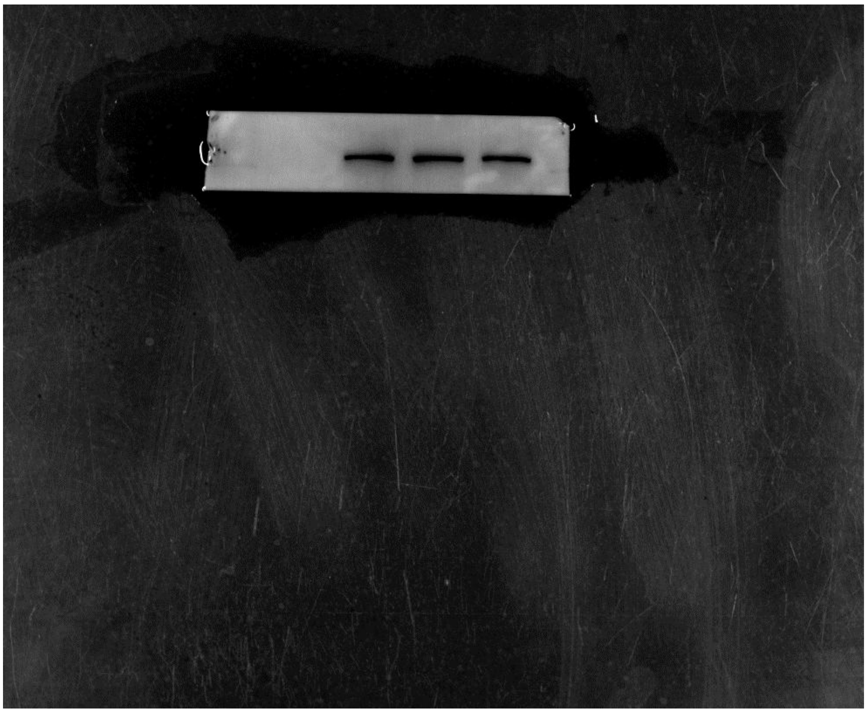

IP-Kla

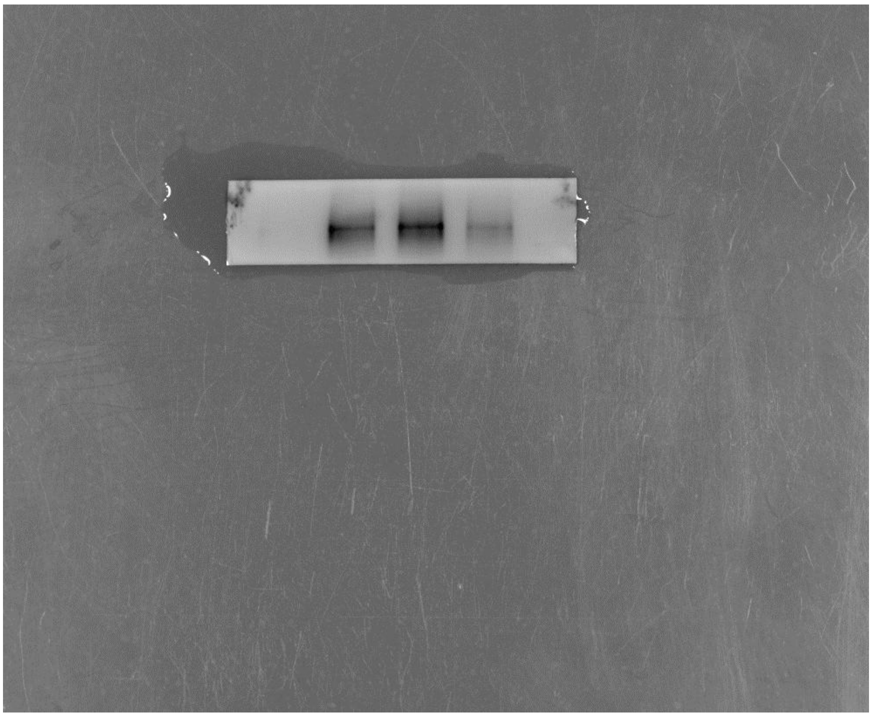

Input-BLM

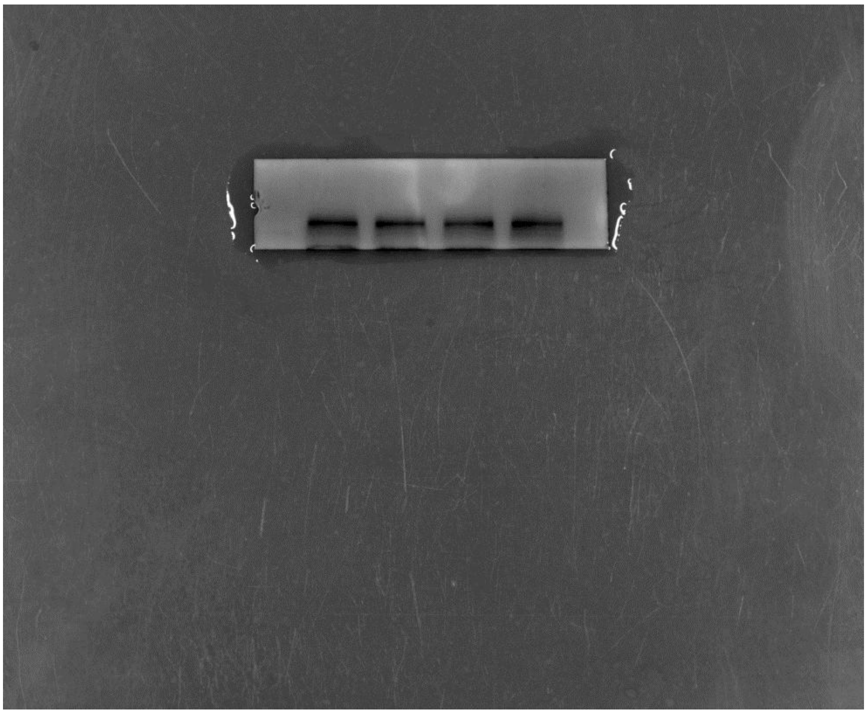

FigureS4I

IP-HA

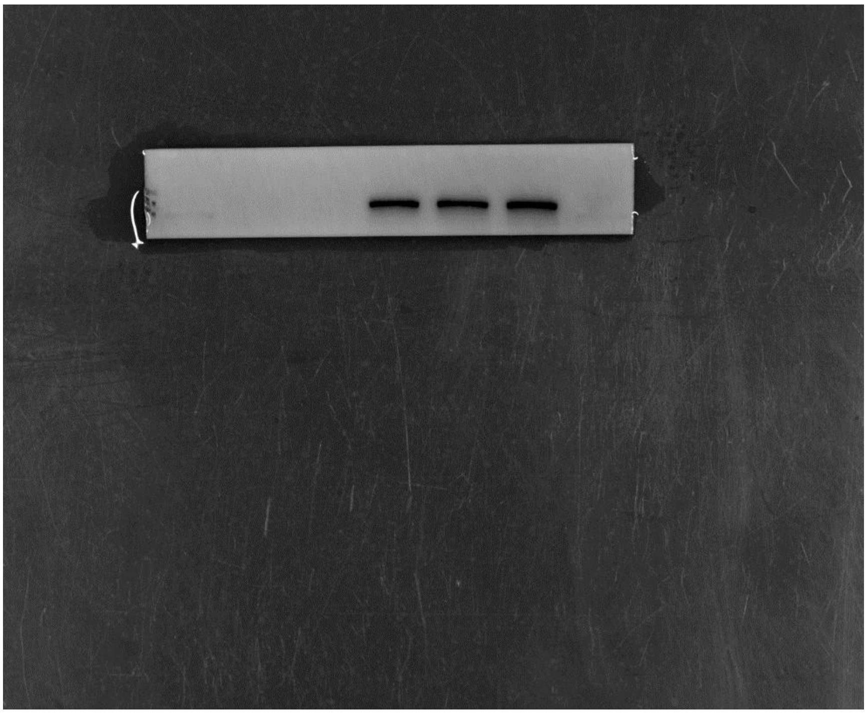

IP-Kla

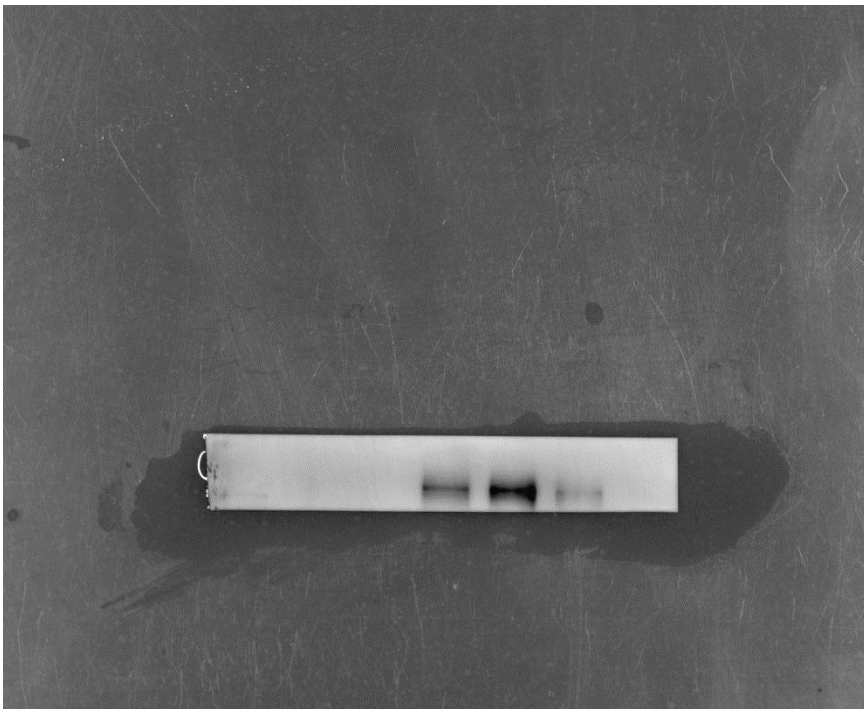

Input-HA

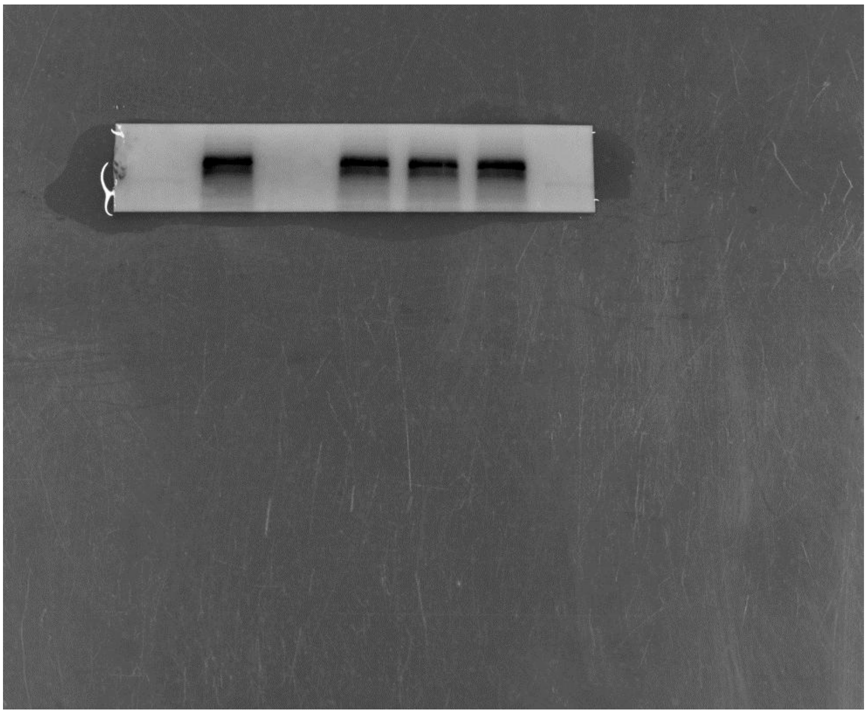

FigureS5A

RAD51

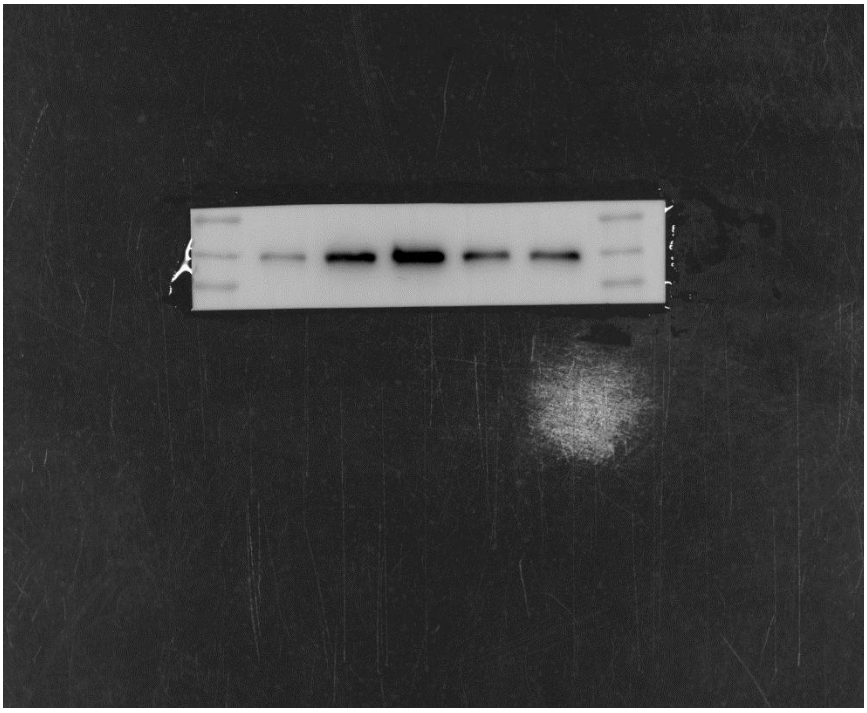

H3

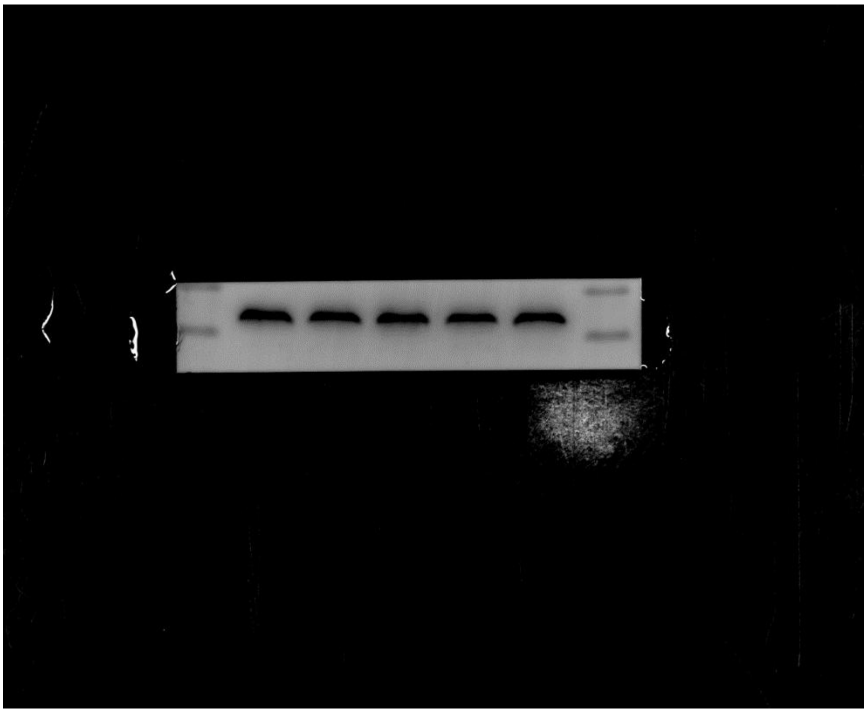

γH2AX

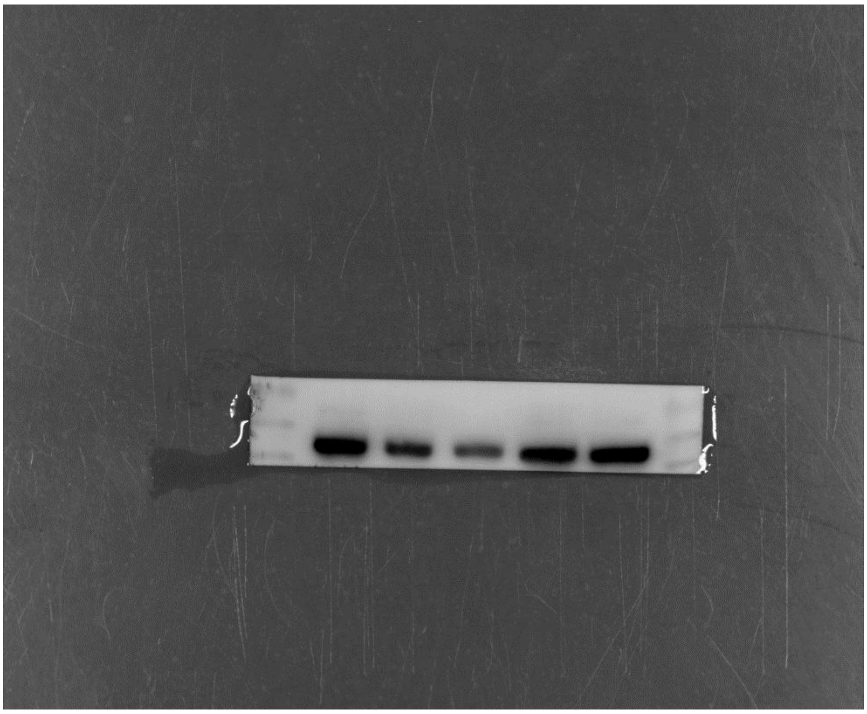

β-actin

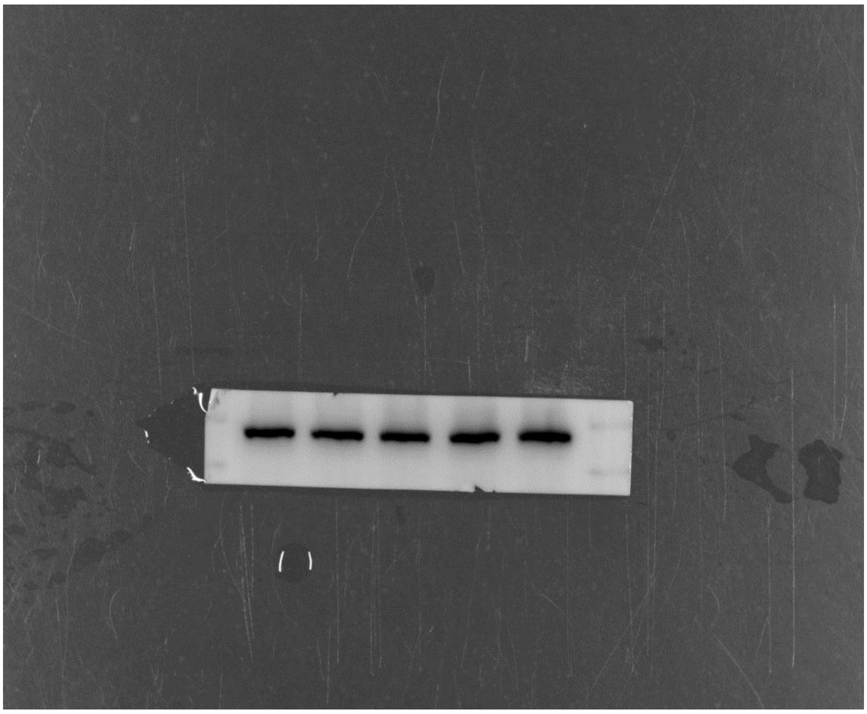

FigureS5B

RAD51

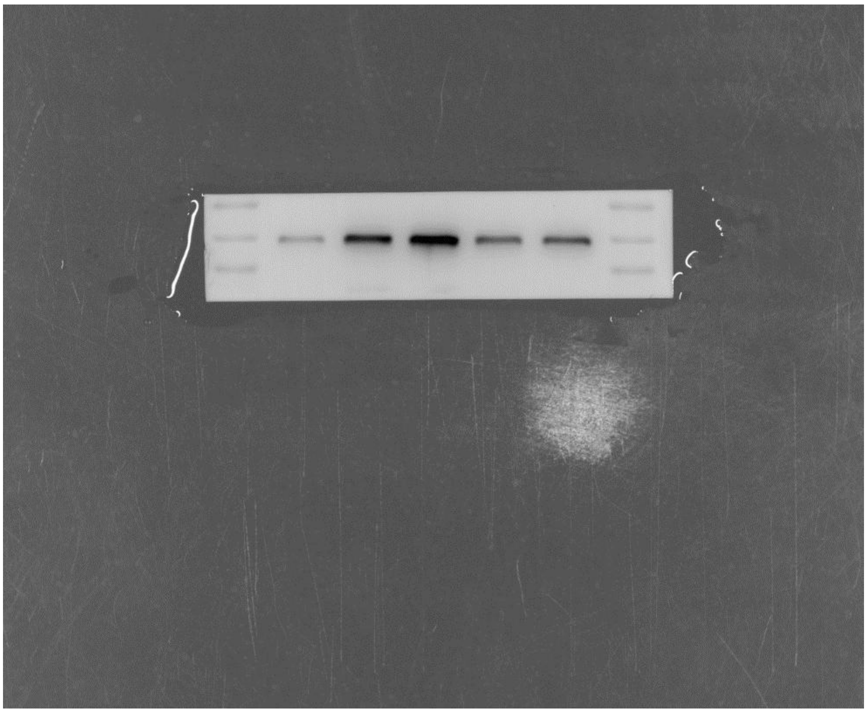

H3

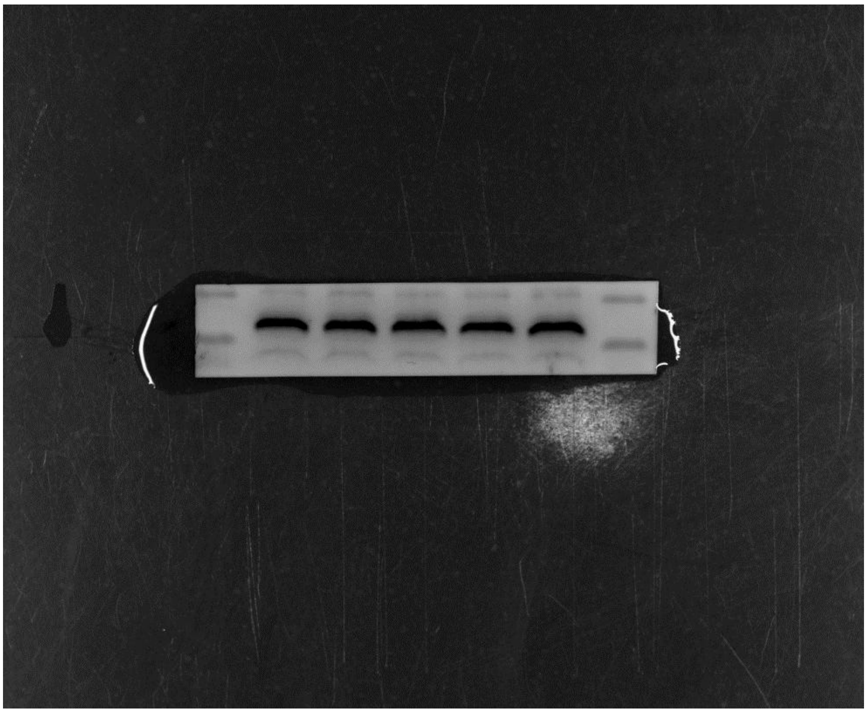

γH2AX

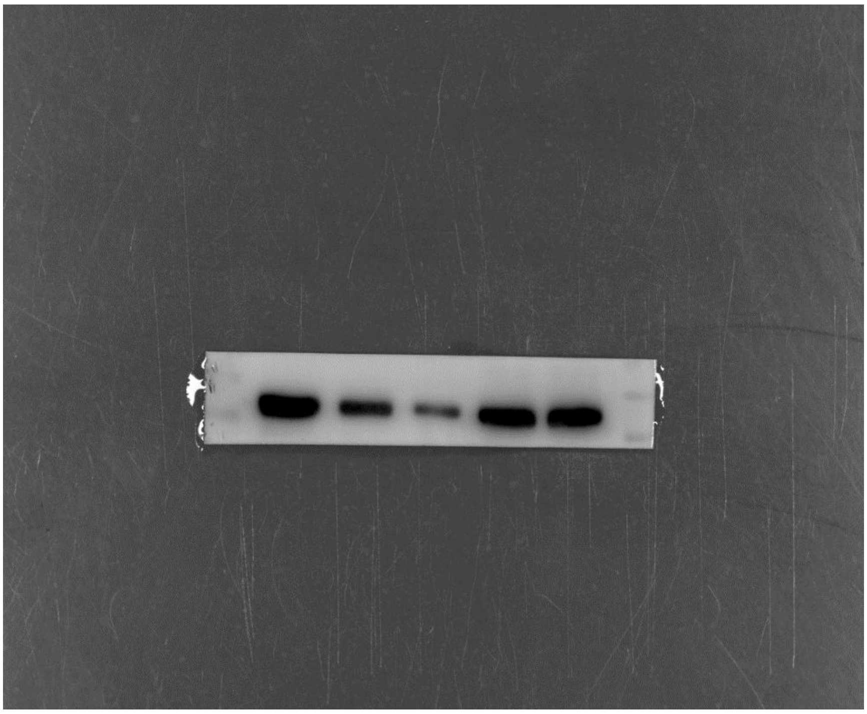

β-actin

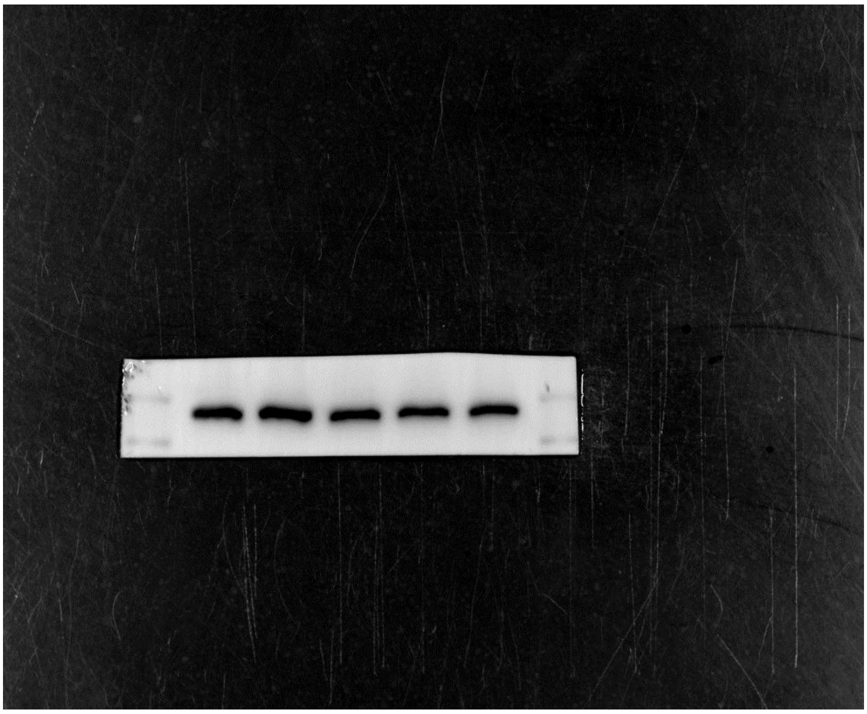

FigureS5F

IP-HA

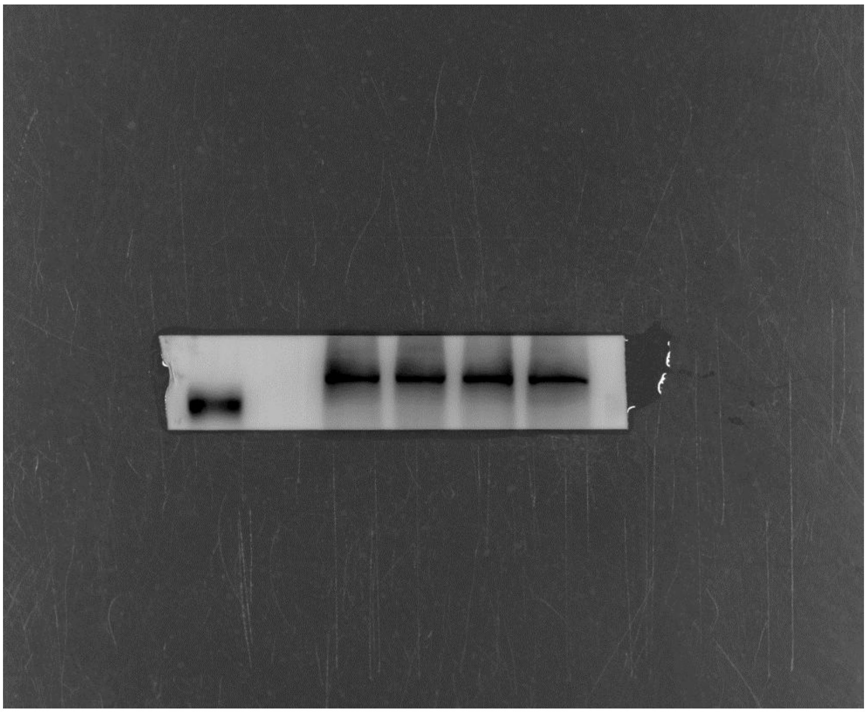

IP-TOPIIIA

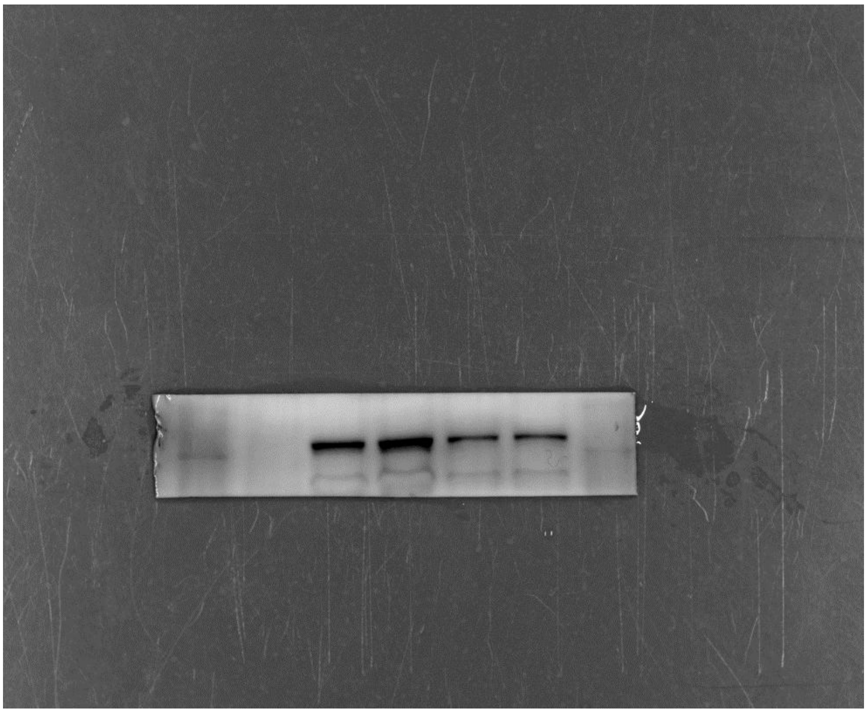

IP-RMI1

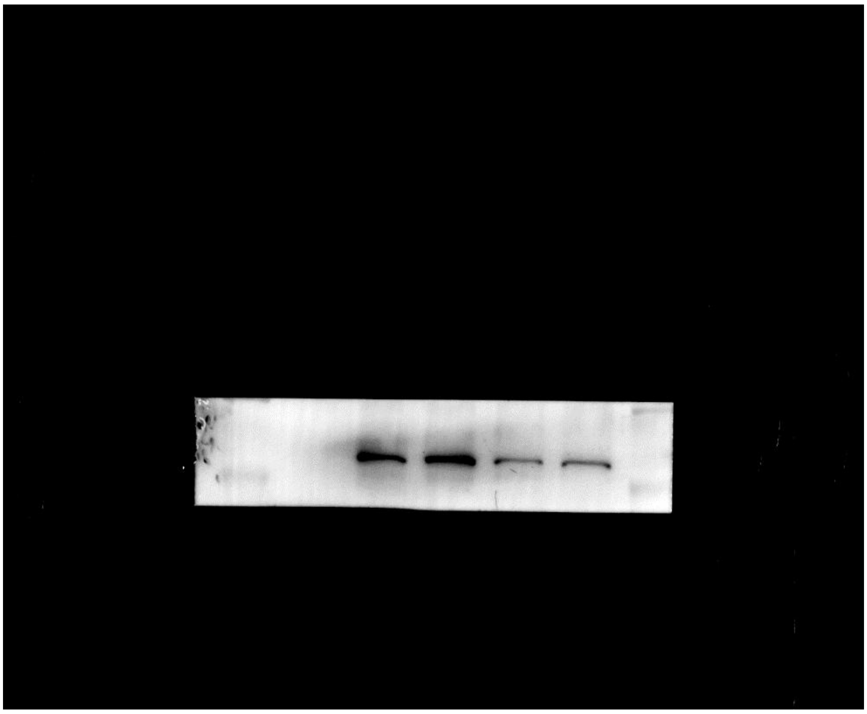

IP-WRN

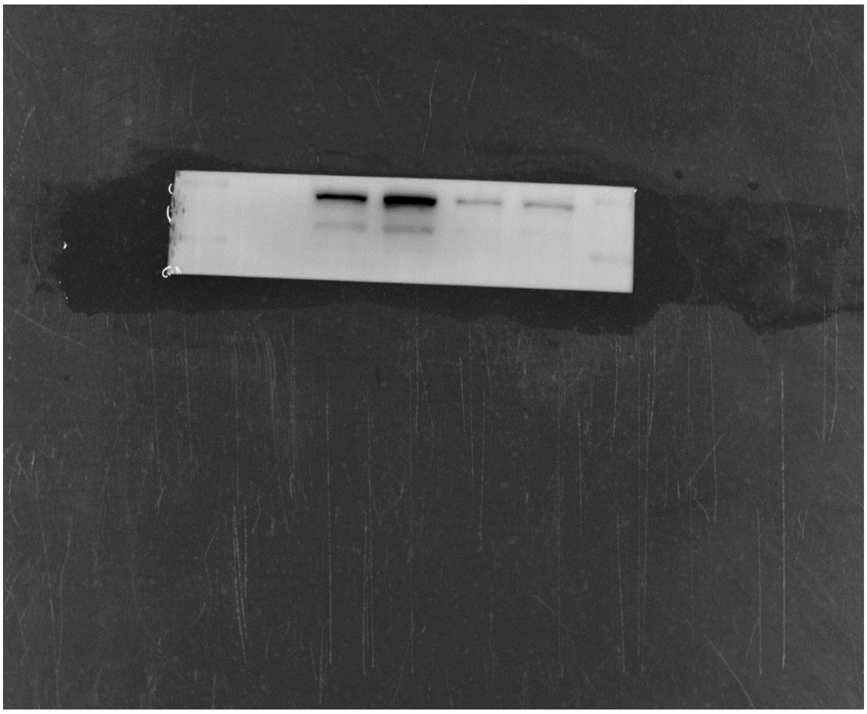

IP-RAD51

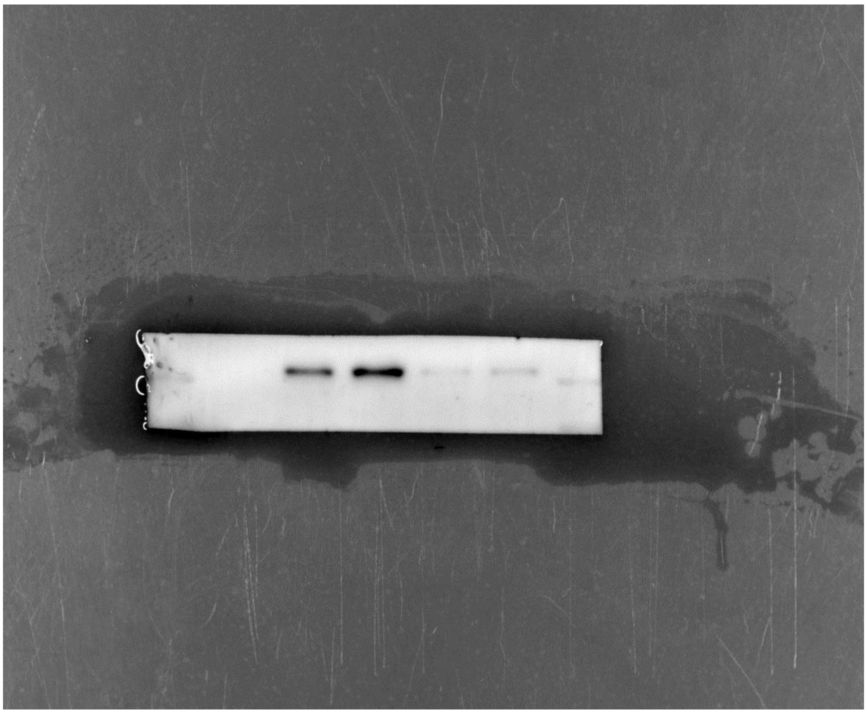

Input-HA

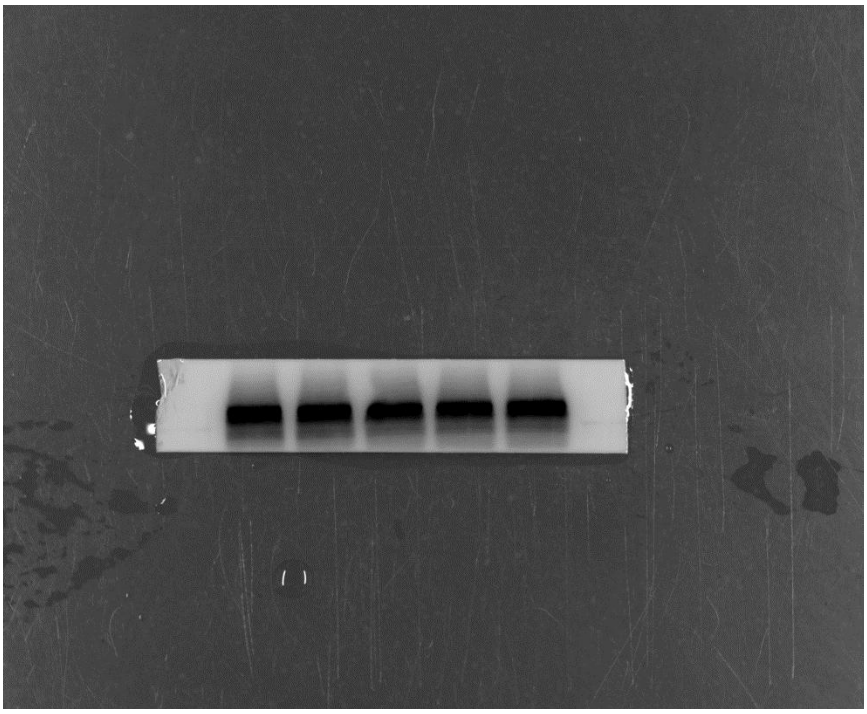

Input-TOPIIIA

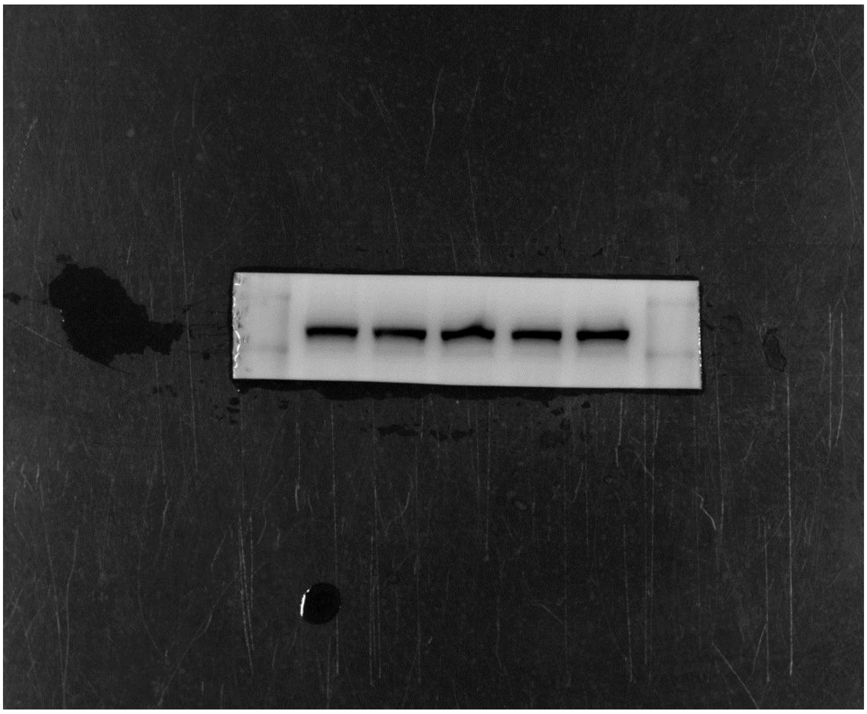

Input-RMI1

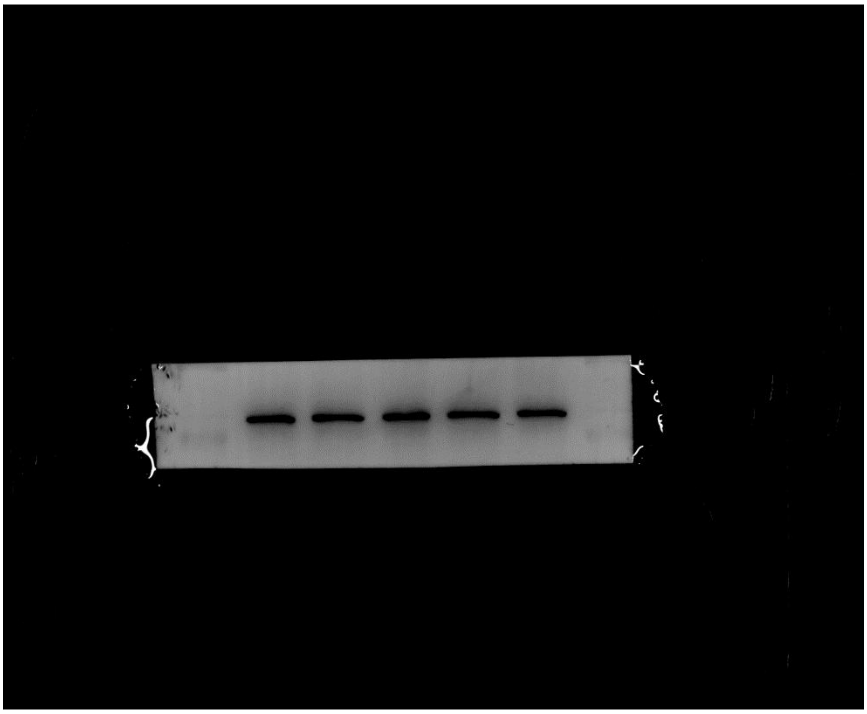

Input-WRN

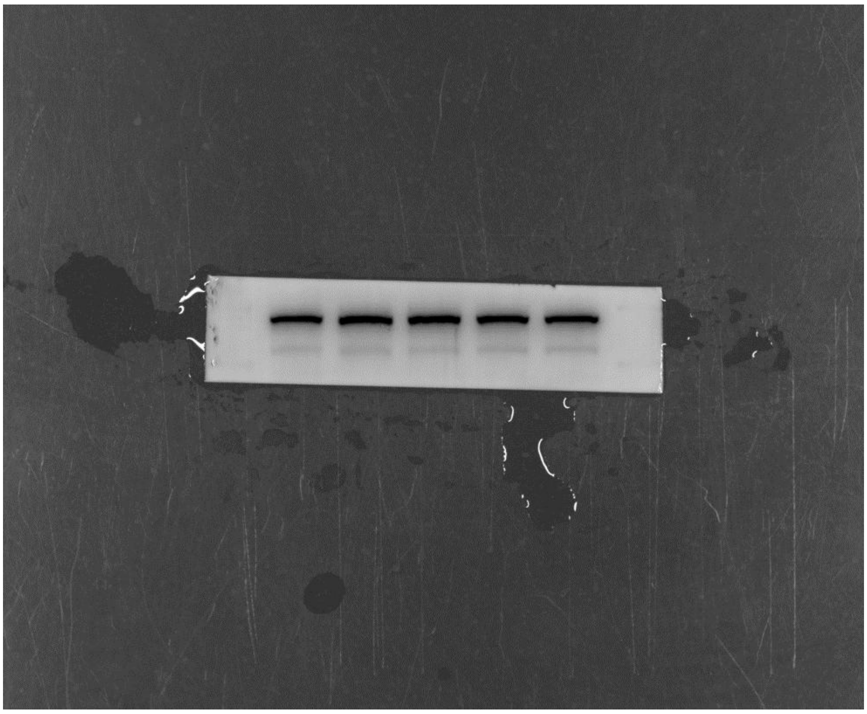

Input-RAD51

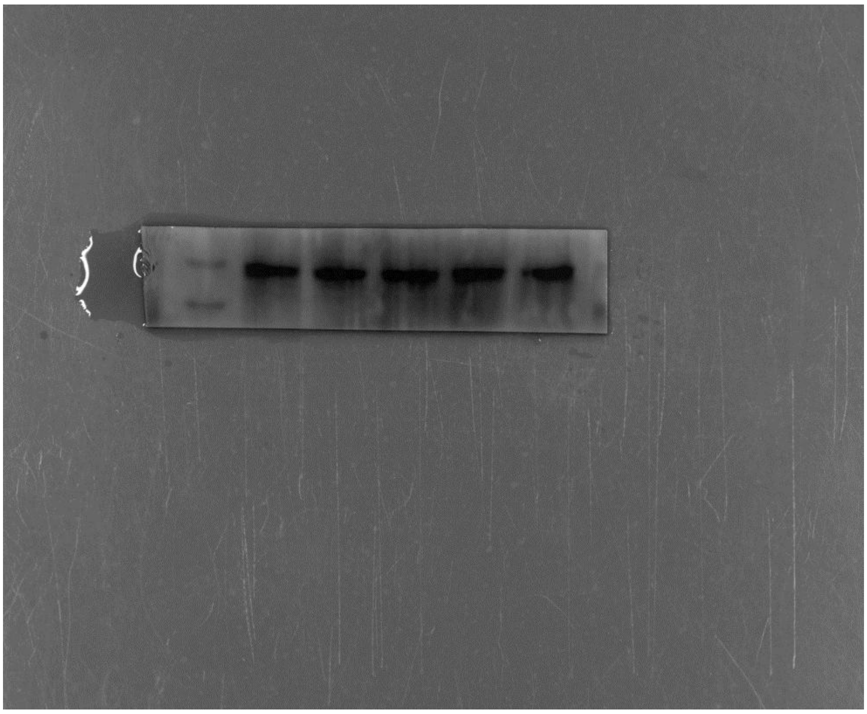

FigureS6A

HA

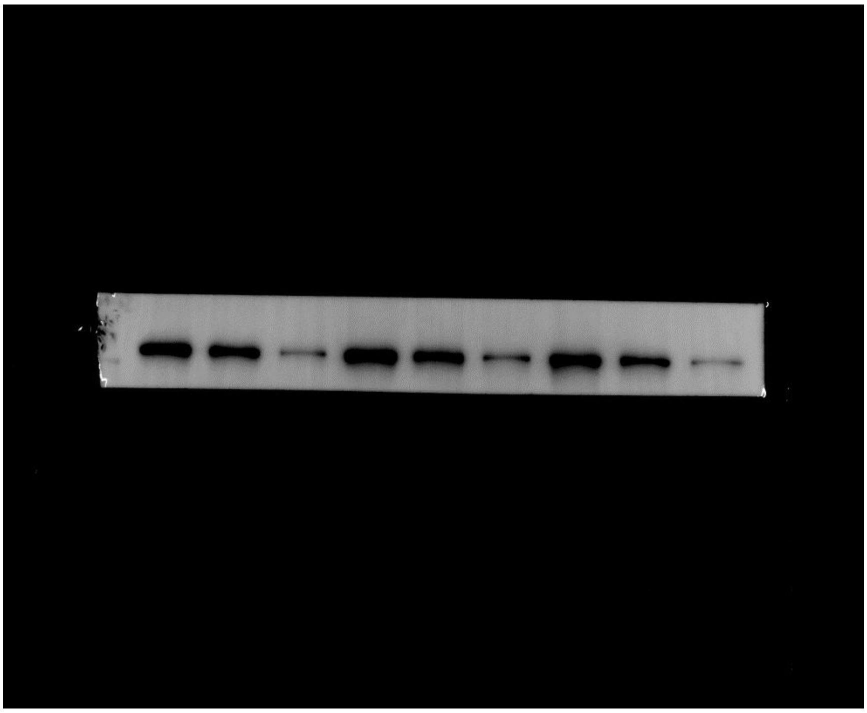

β-actin

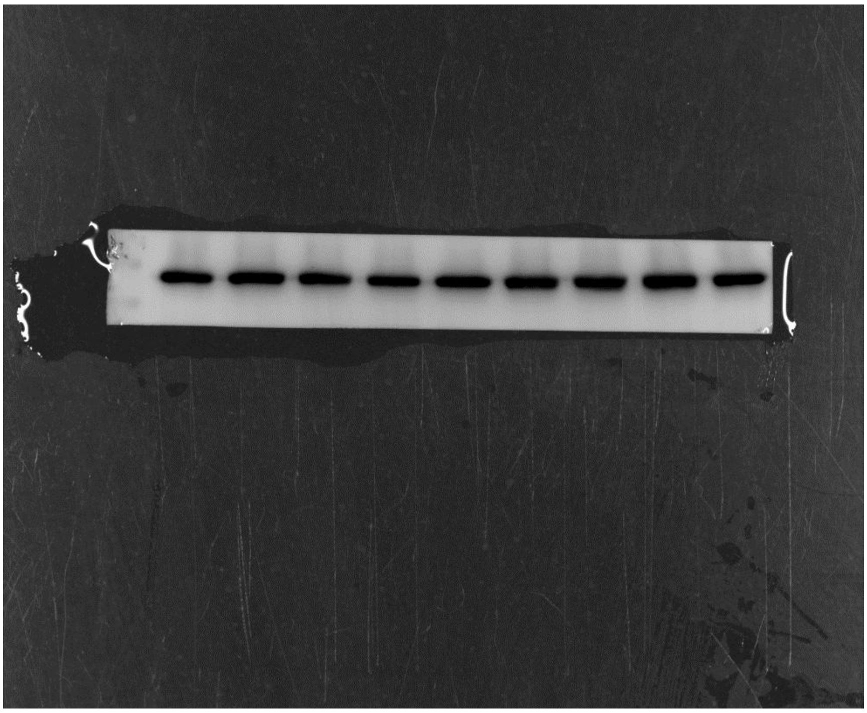

FigureS6C

IP-Flag

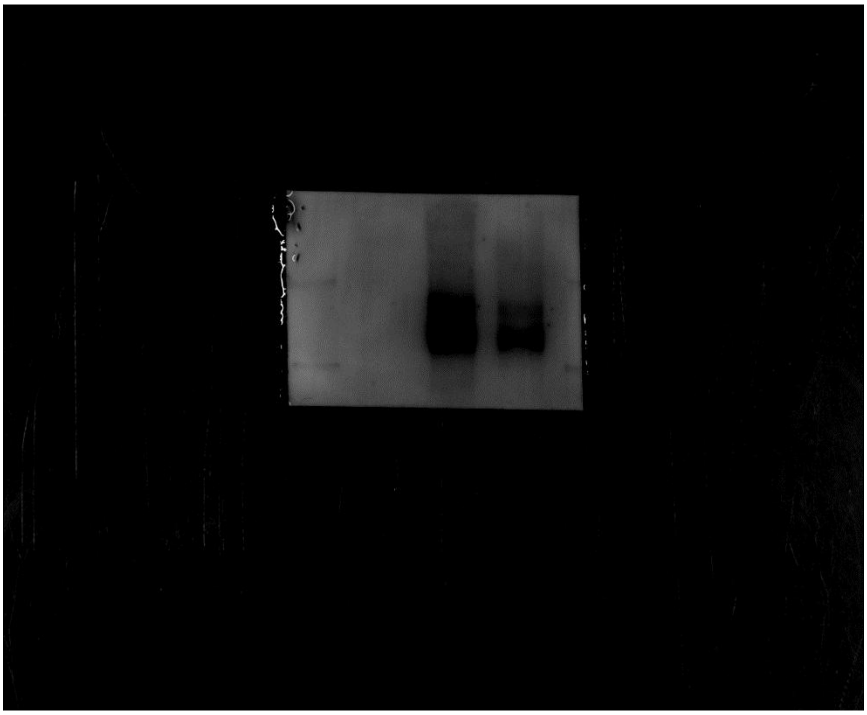

IP-HA

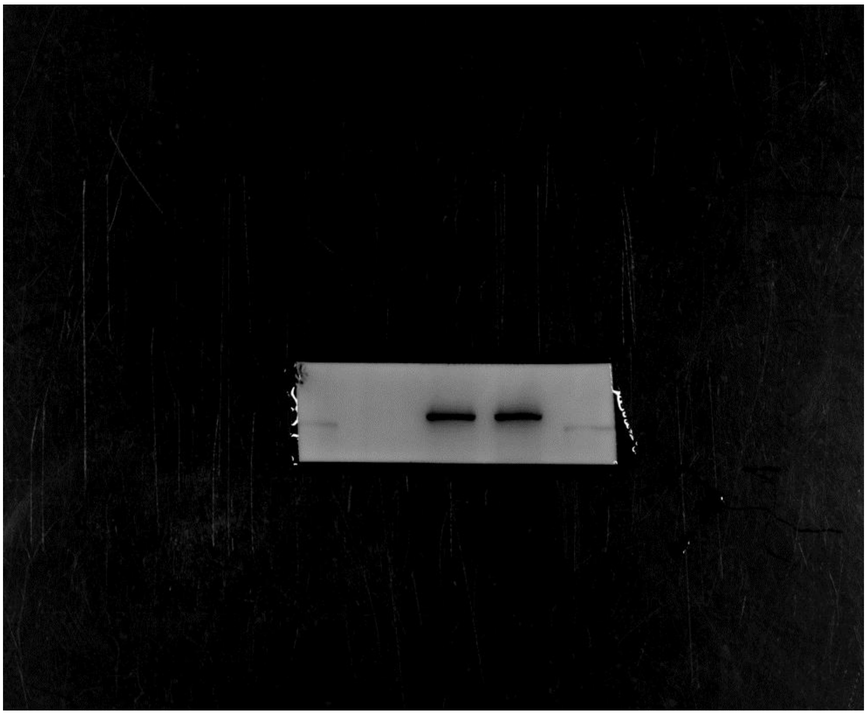

Input-HA

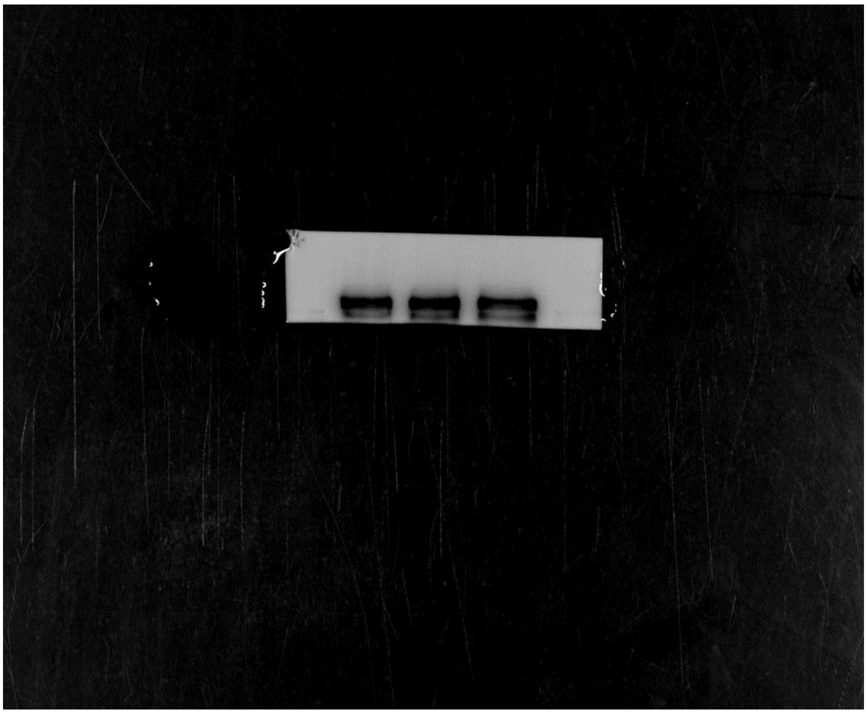

FigureS6D

IP-Flag

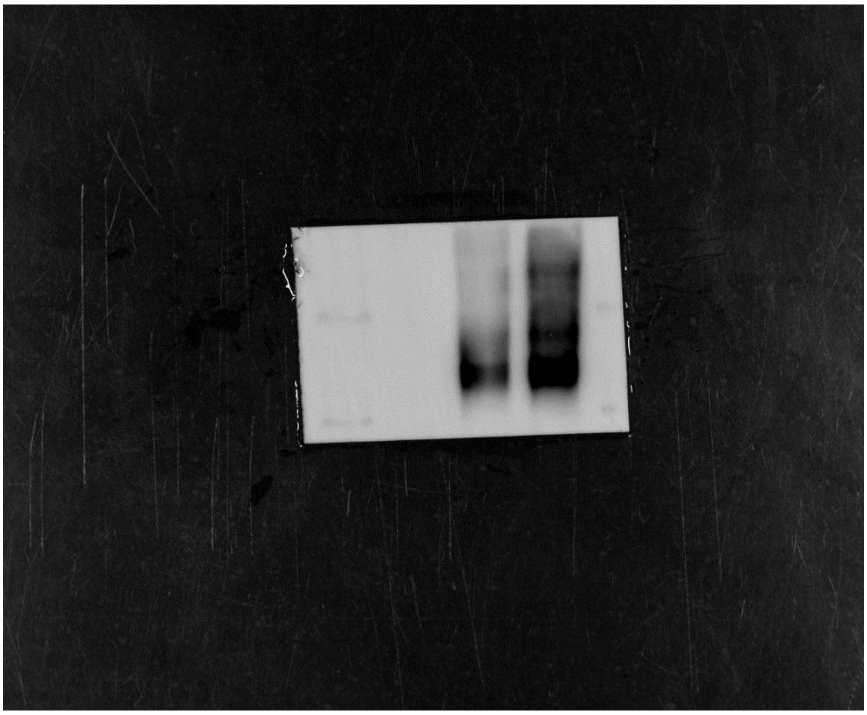

IP-HA

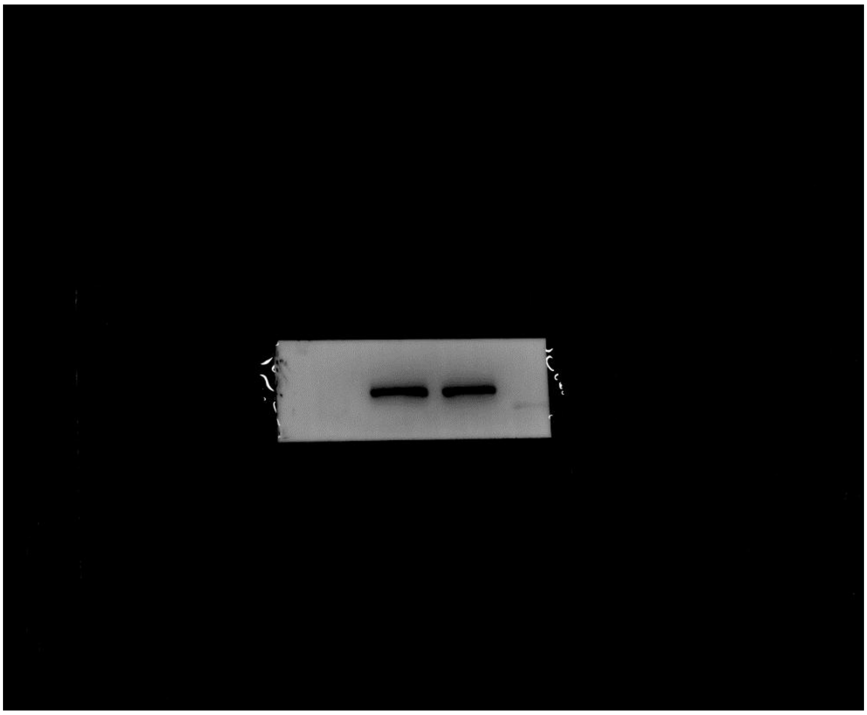

IP-His

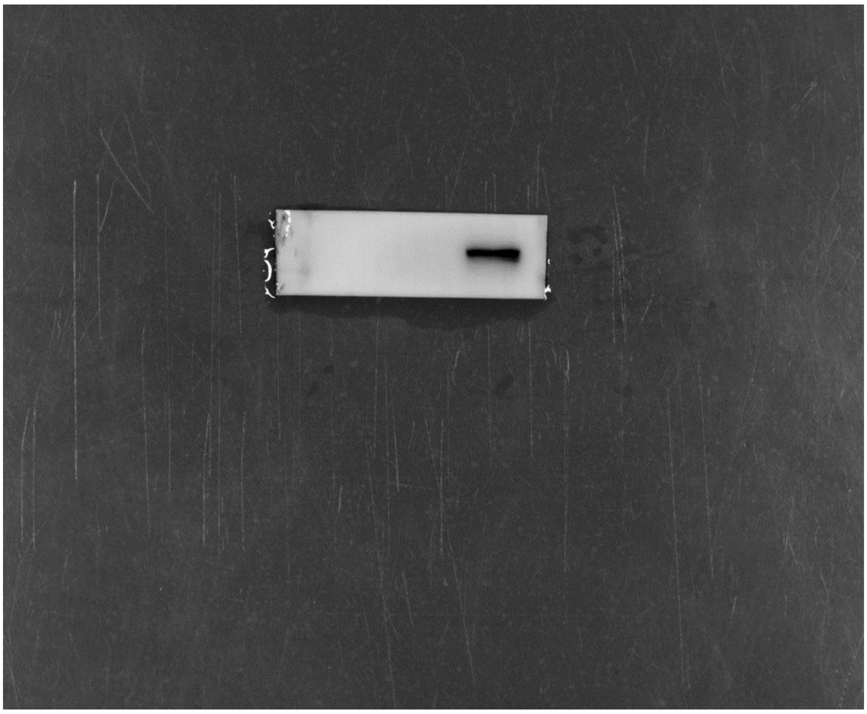

Input-HA

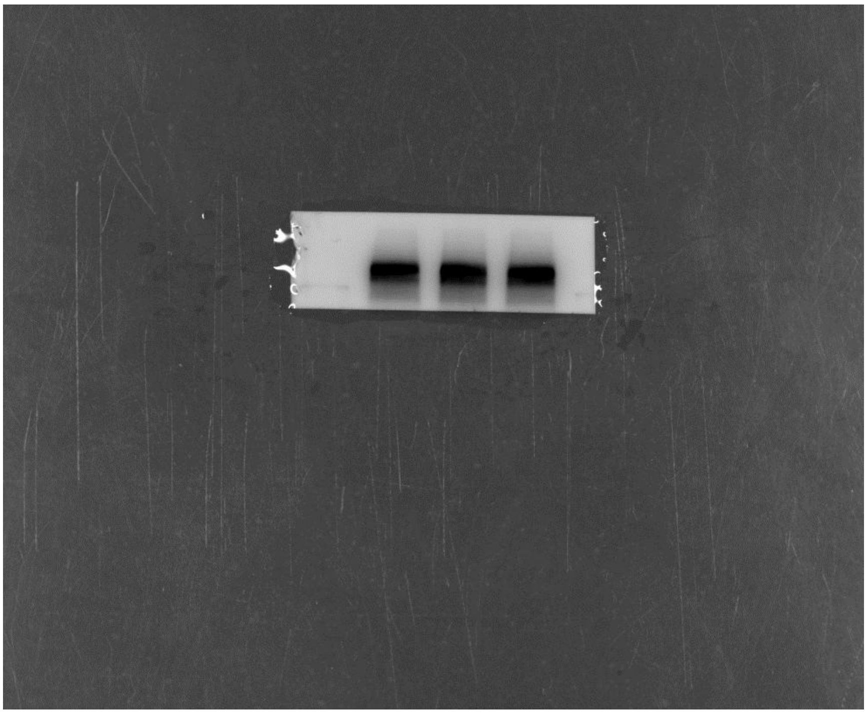

Input-His

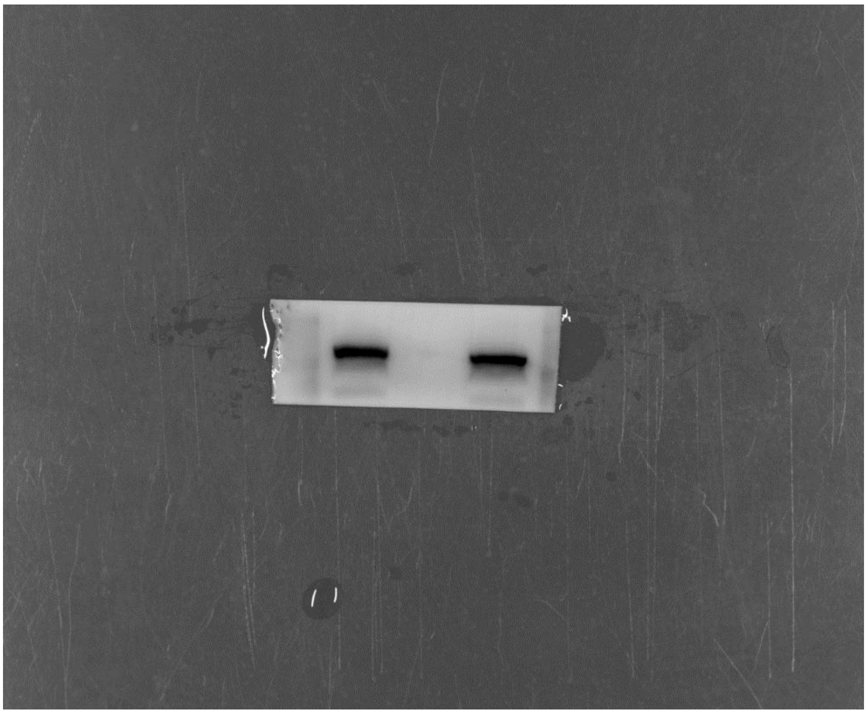

FigureS6G

HA

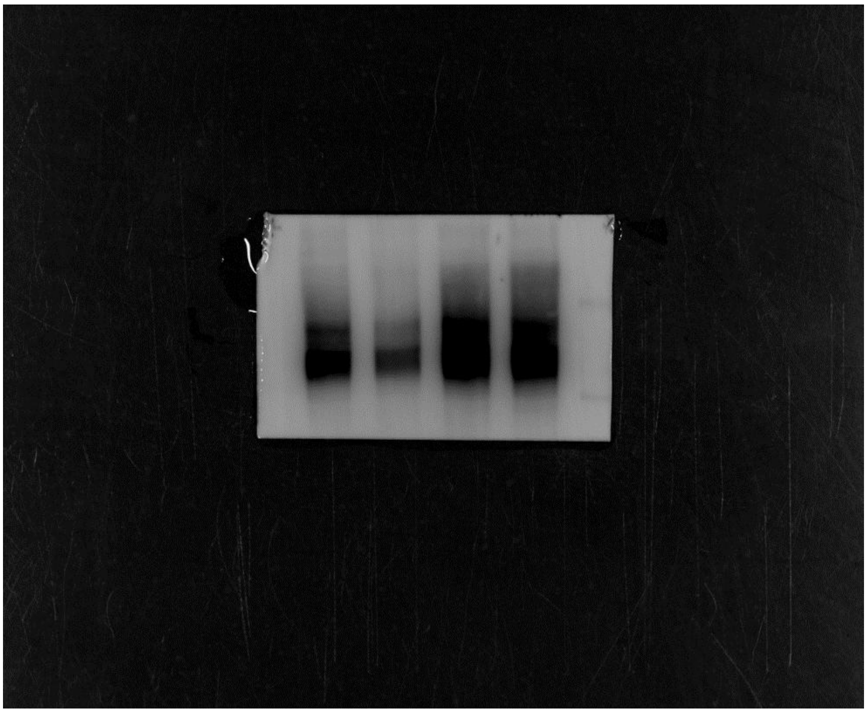

His

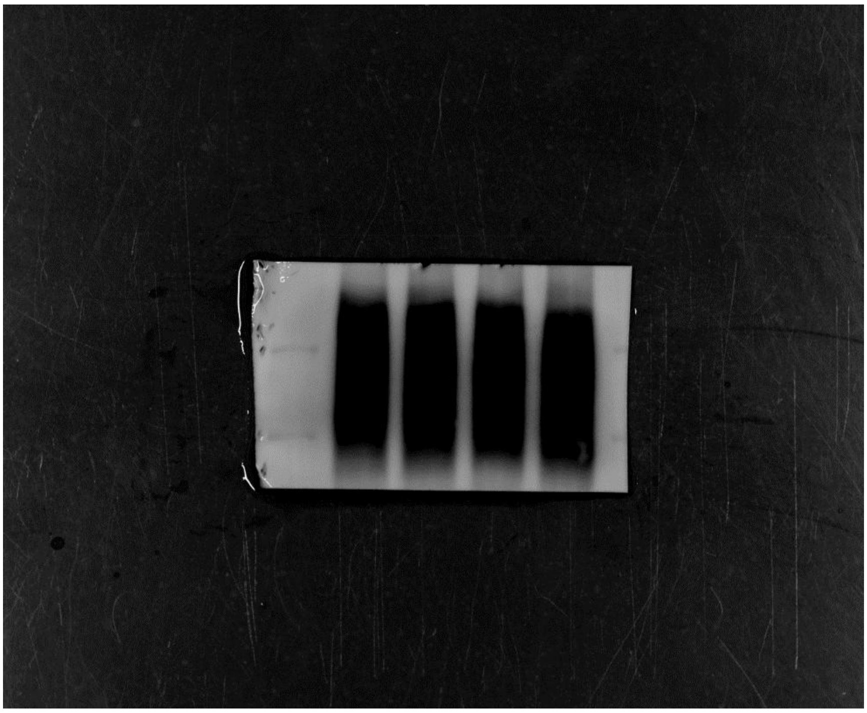

Input-HA

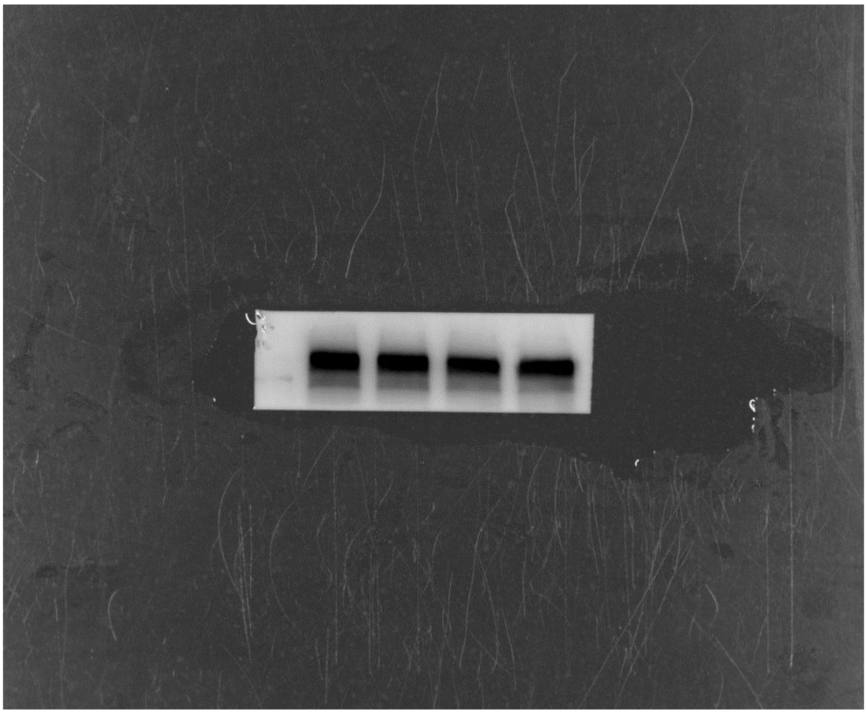

FigureS6H

IP-Flag

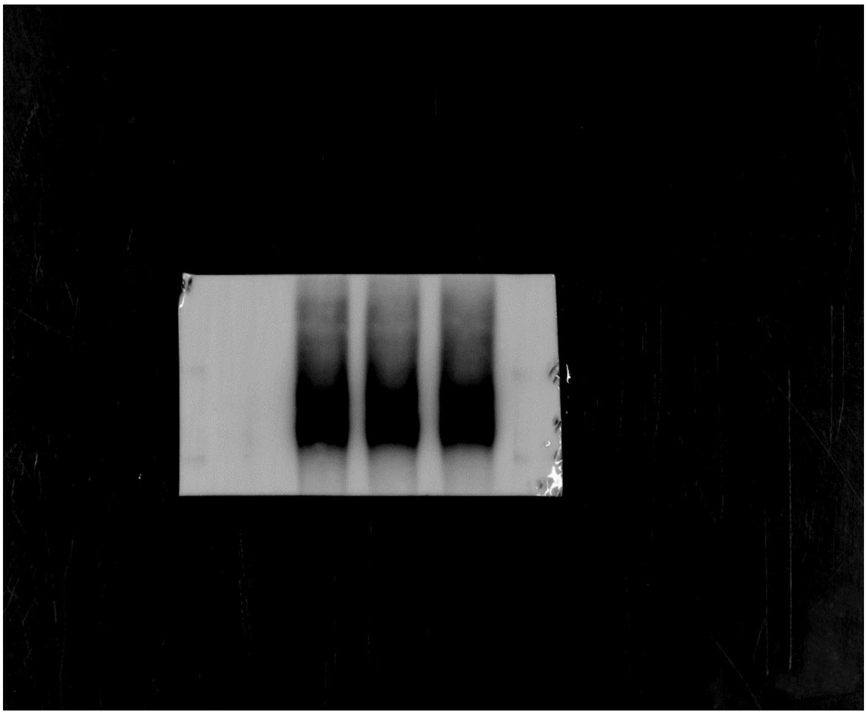

IP-His

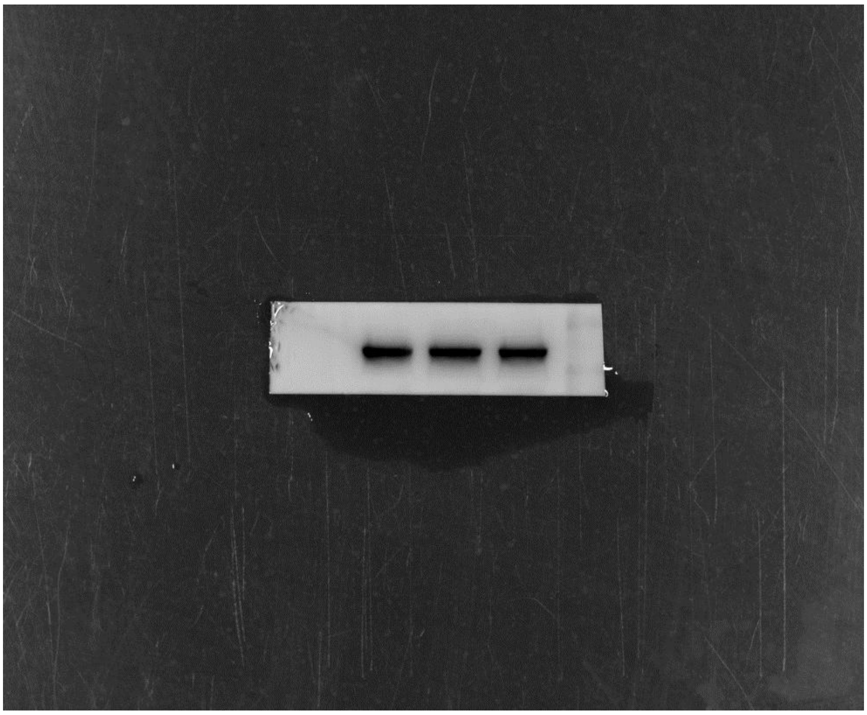

IP-HA

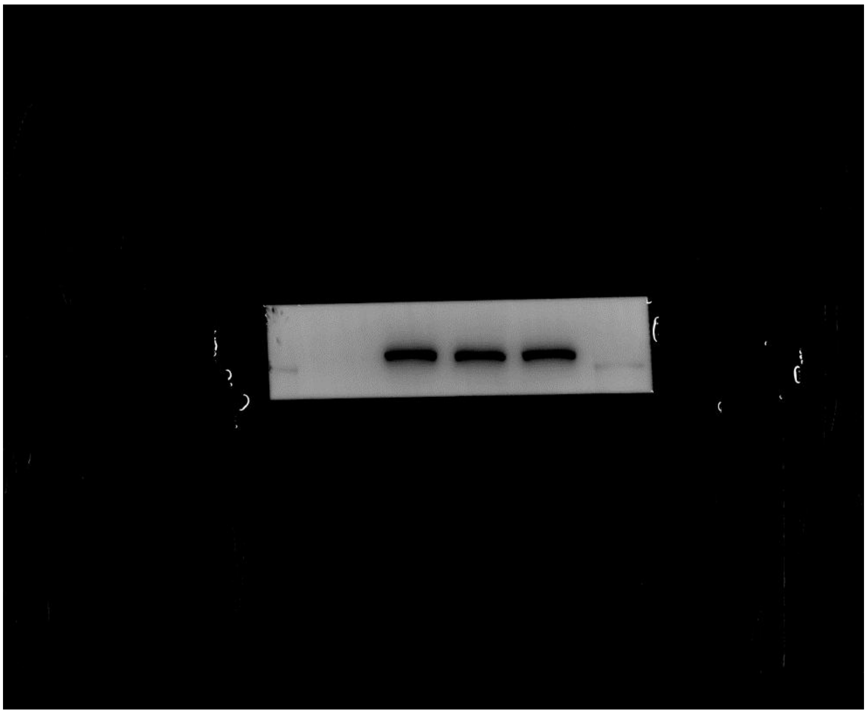

Input-His

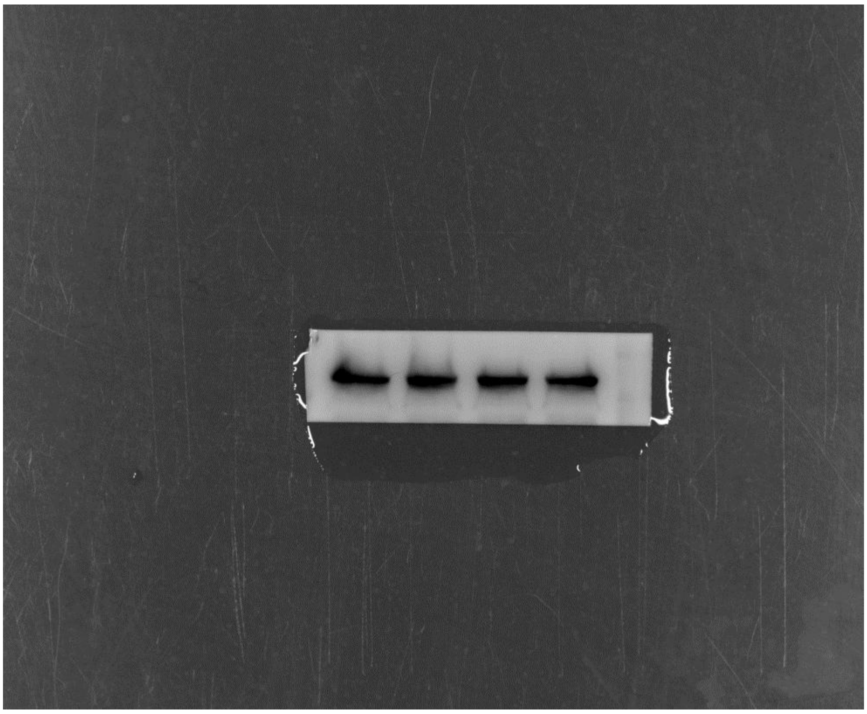

Input-HA

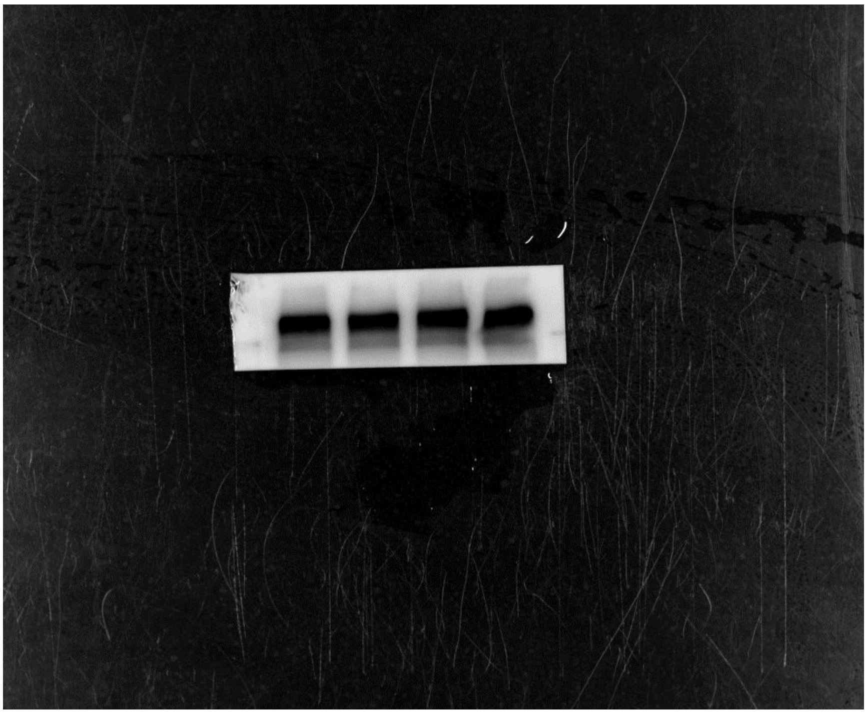

FigureS7A

TIP60

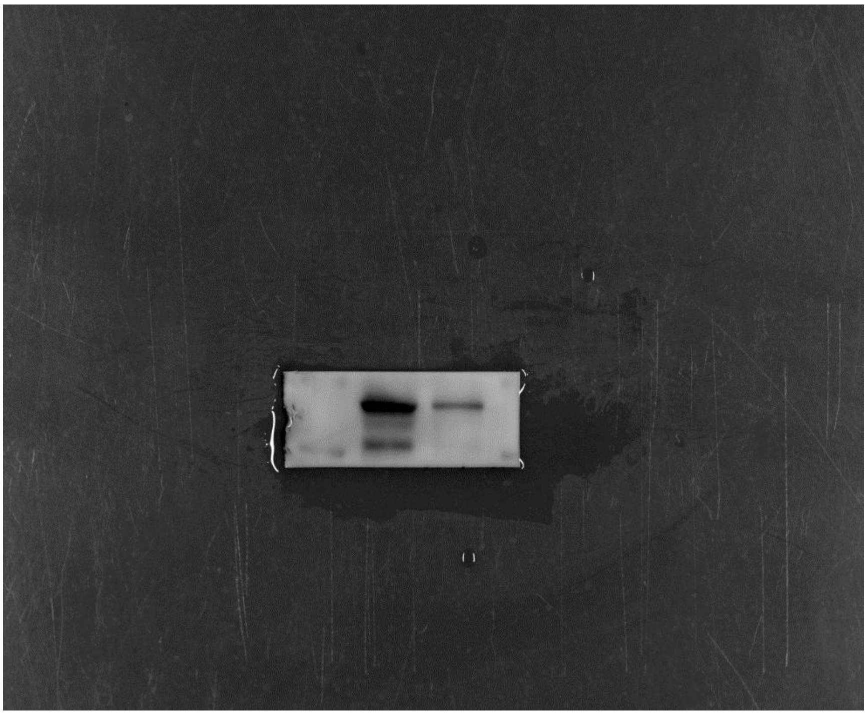

β-actin

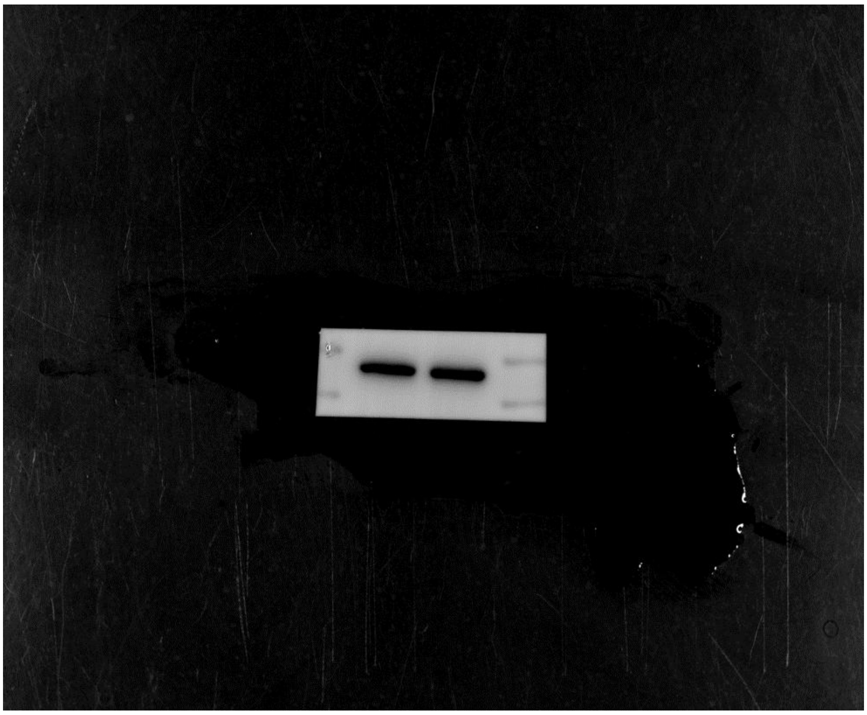

P300

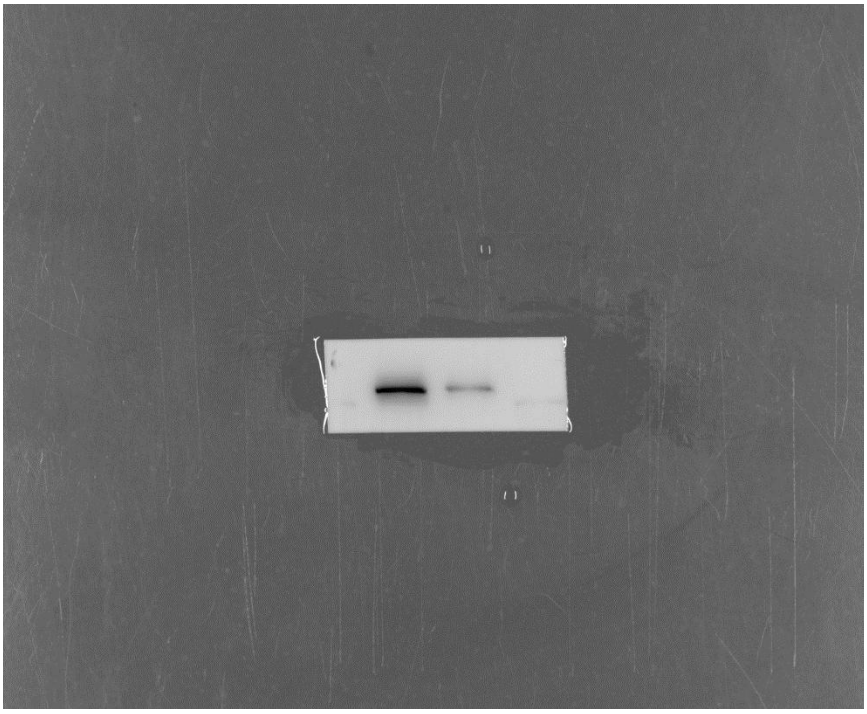

β-actin

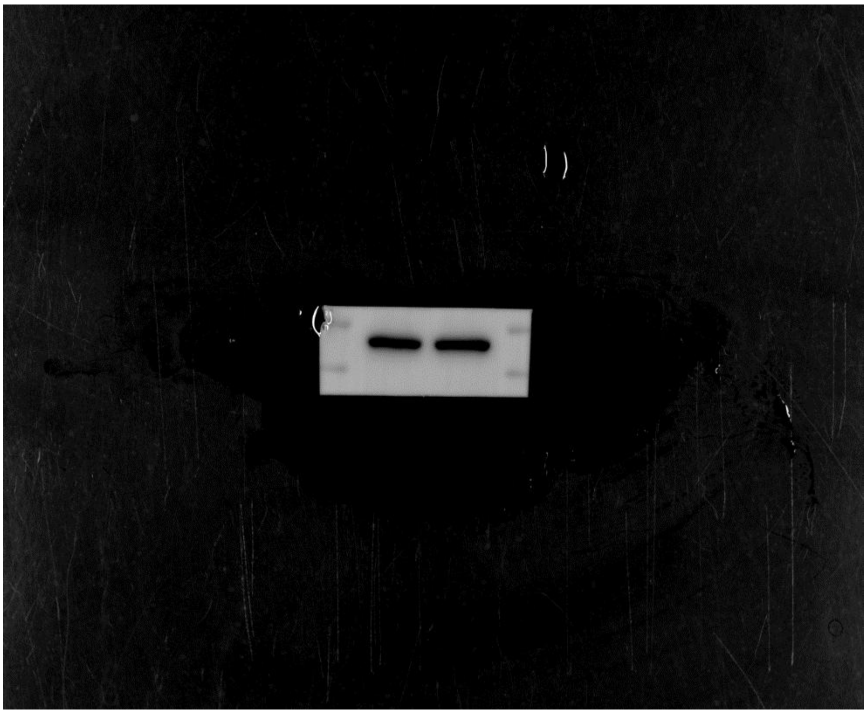

CBP

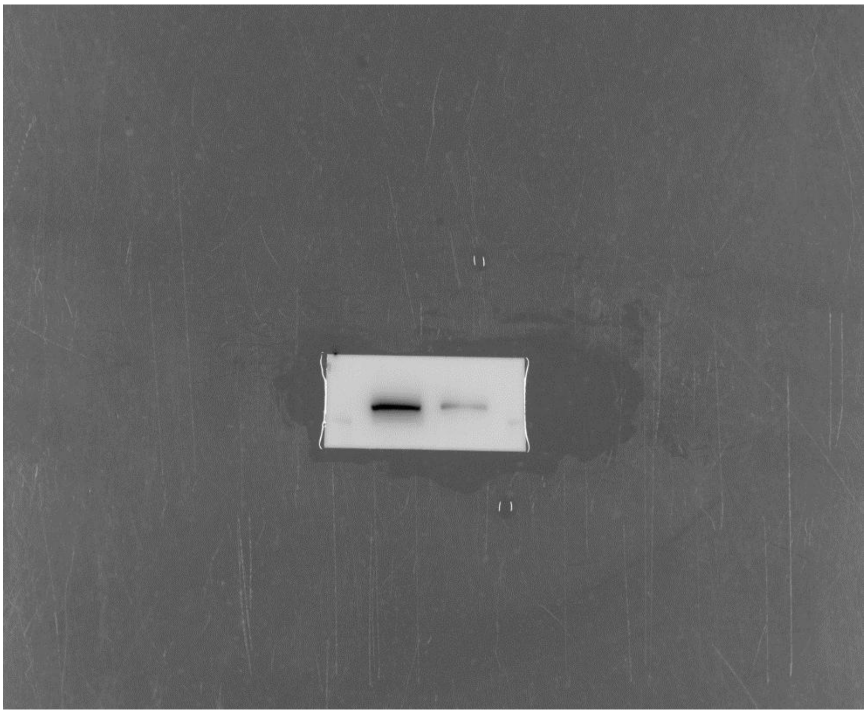

β-actin

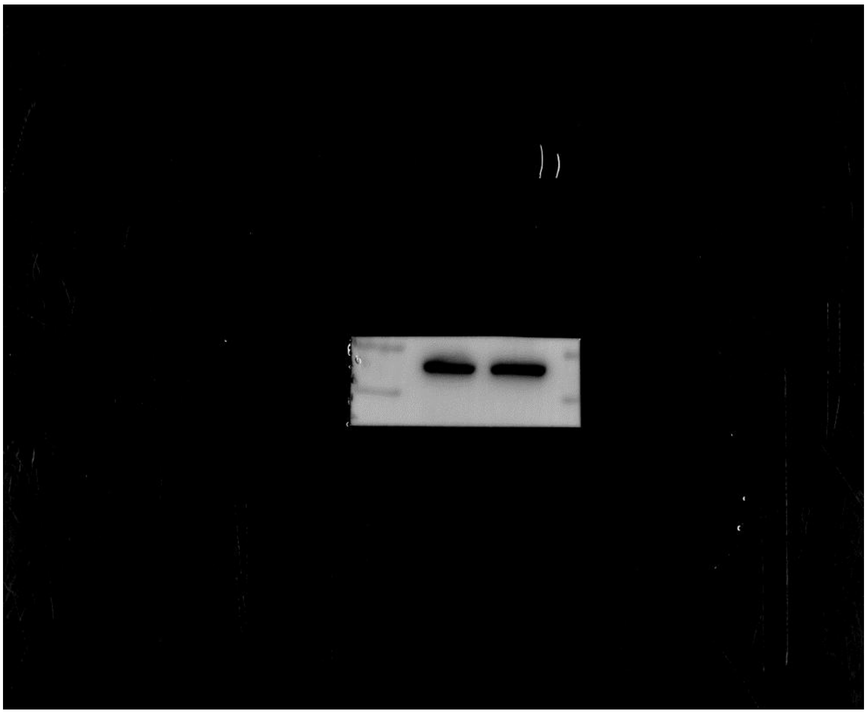

AARS1

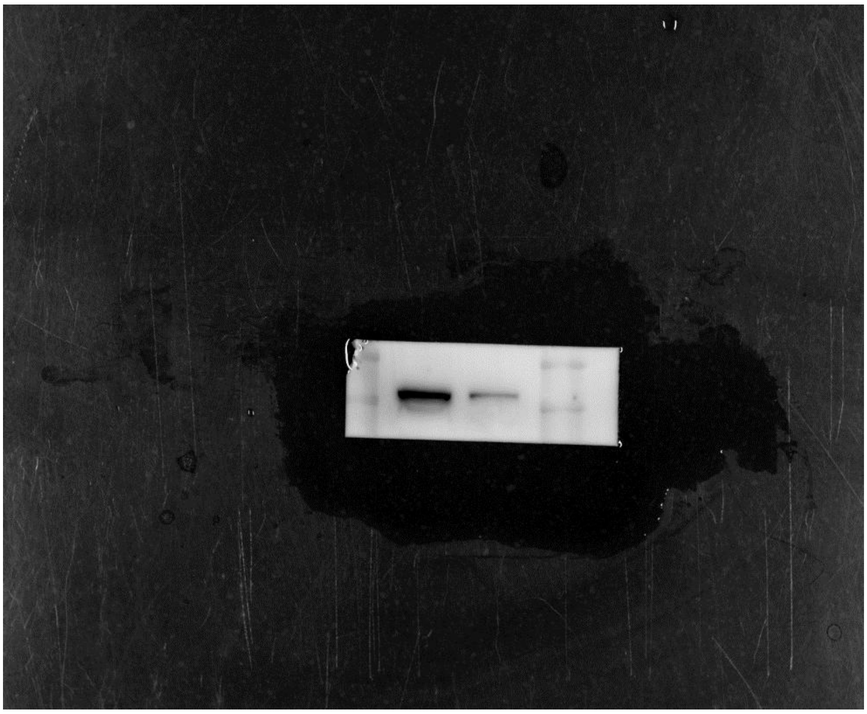

β-actin

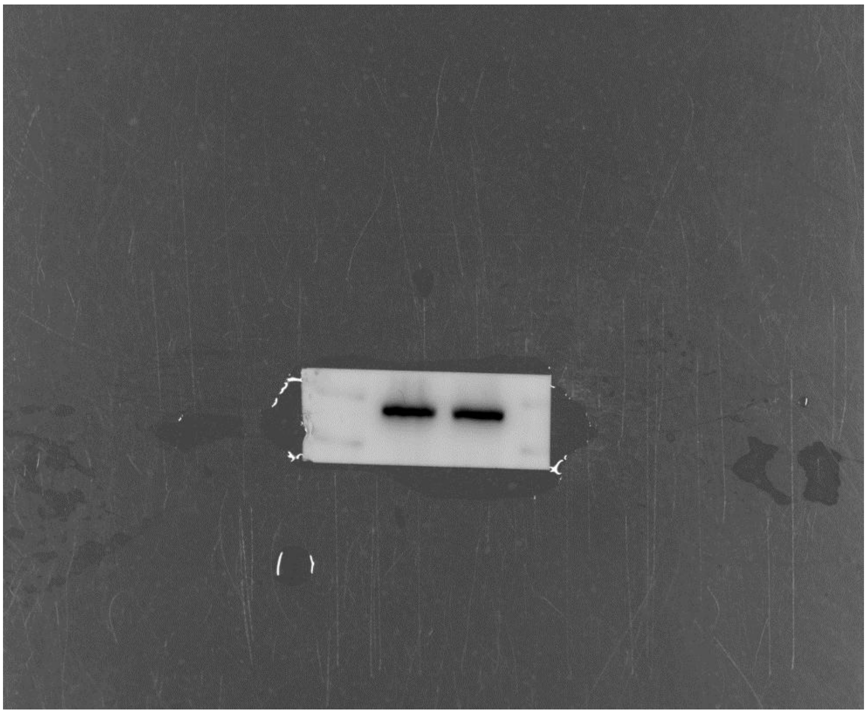

AARS2

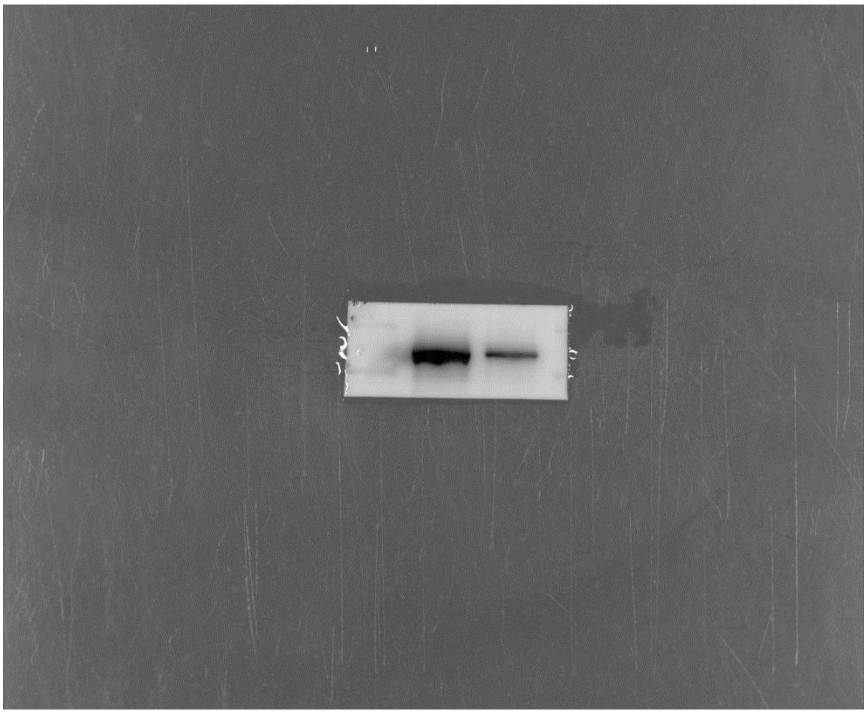

β-actin

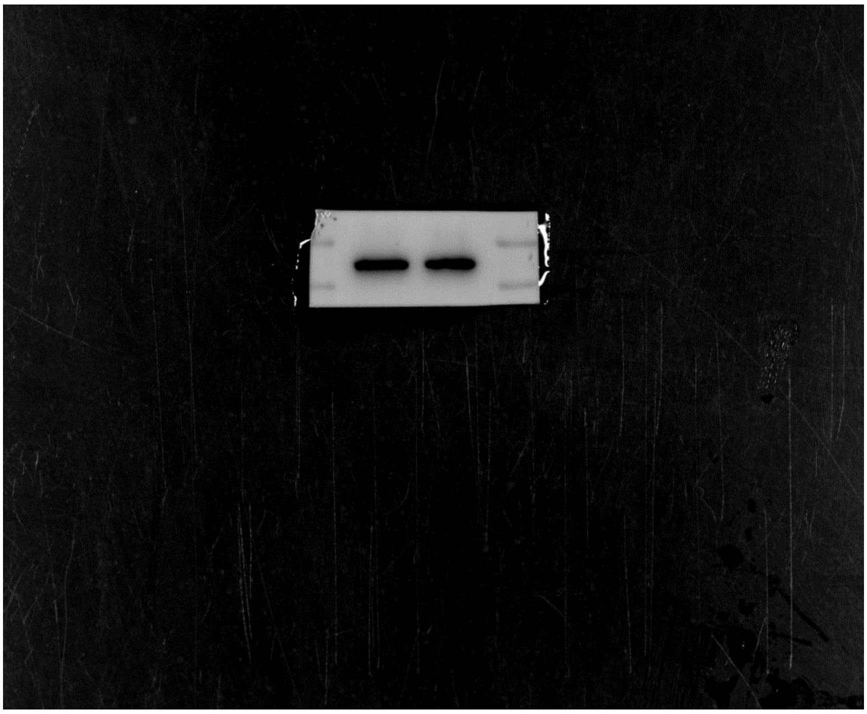

FigureS7C

AARS1

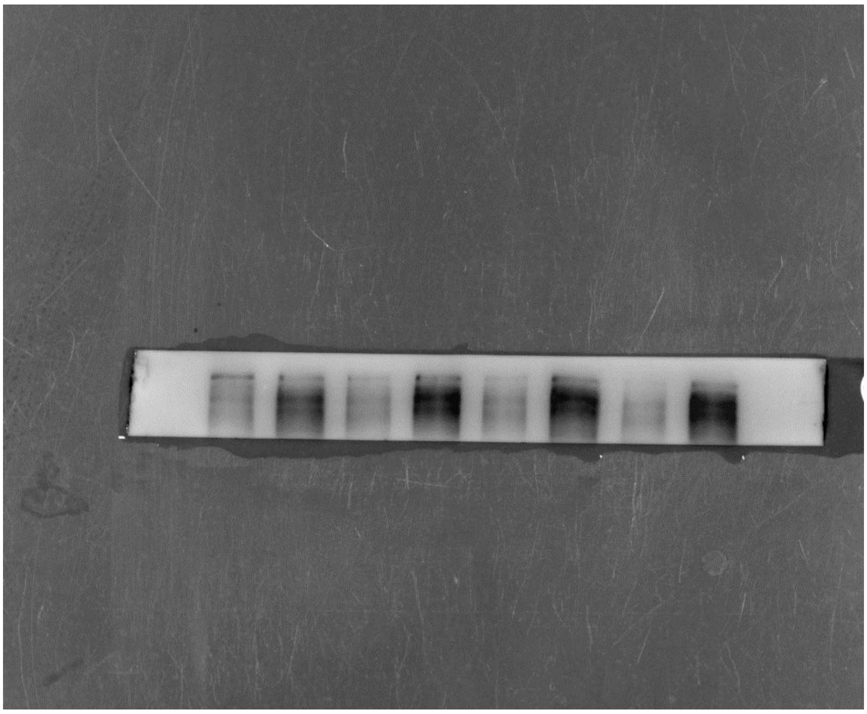

β-actin

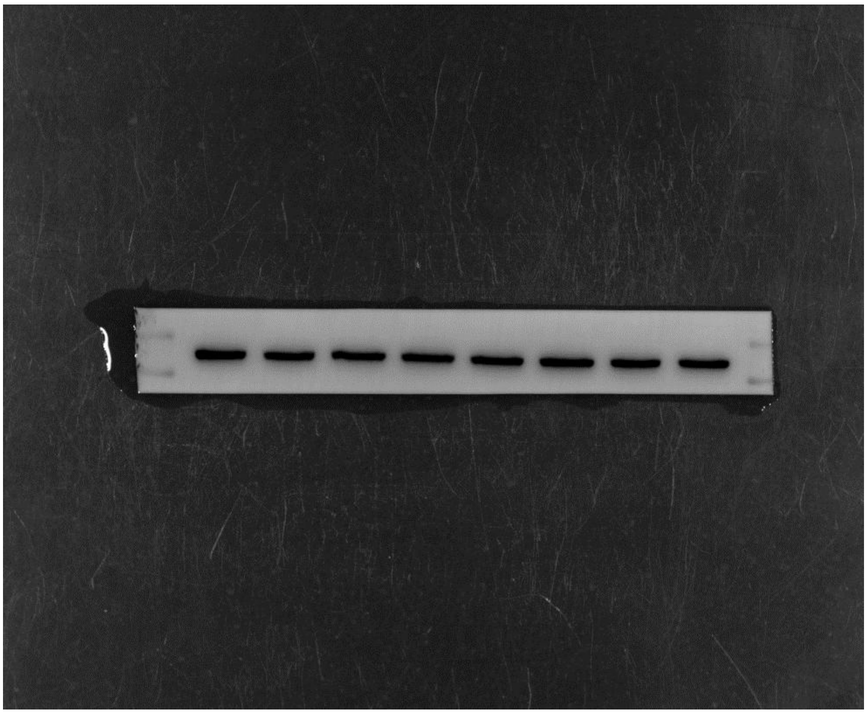

FigureS7D

AARS1

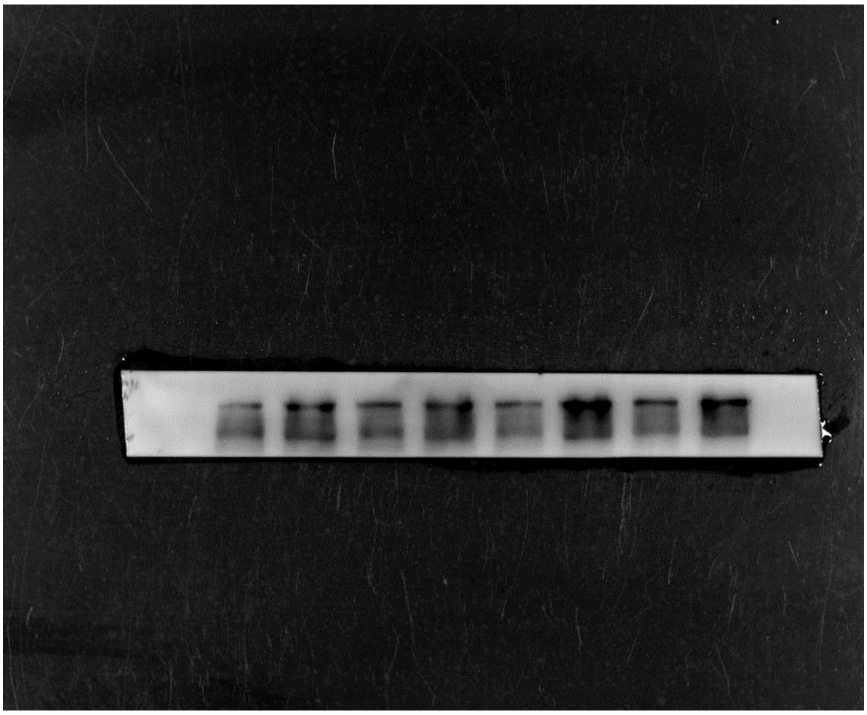

β-actin

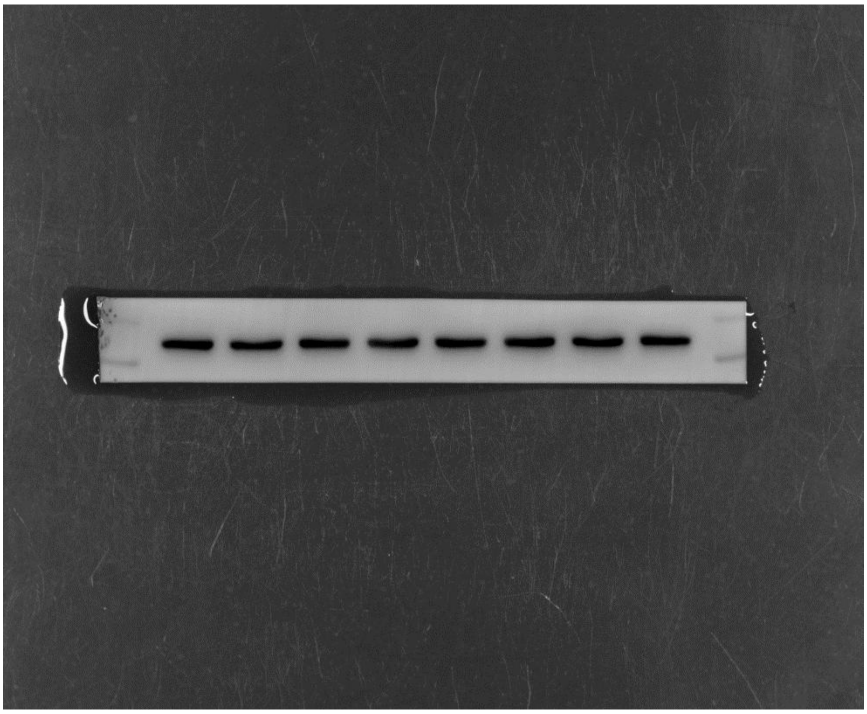

FigureS7G

AARS1

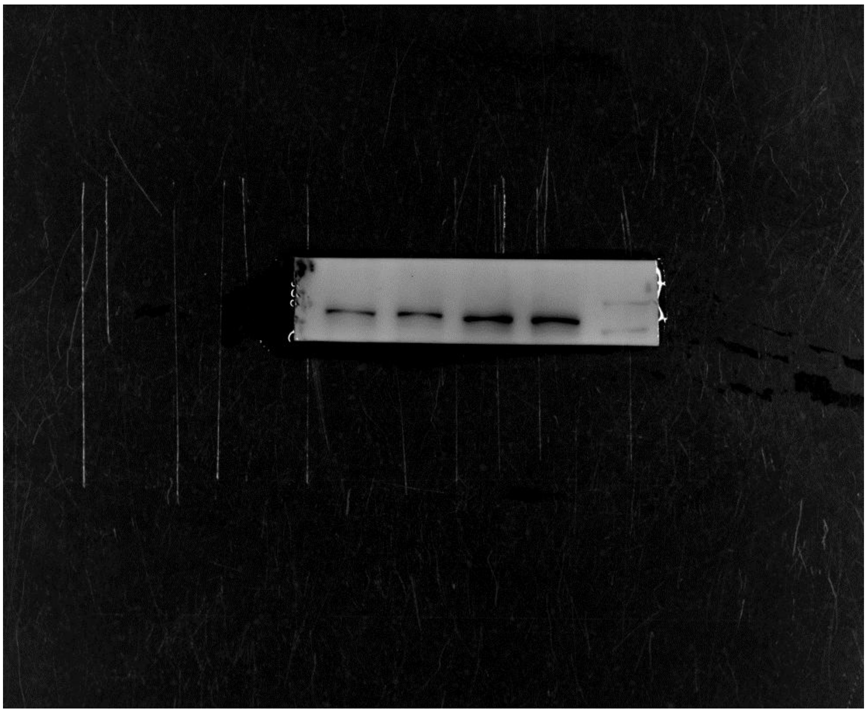

β-actin

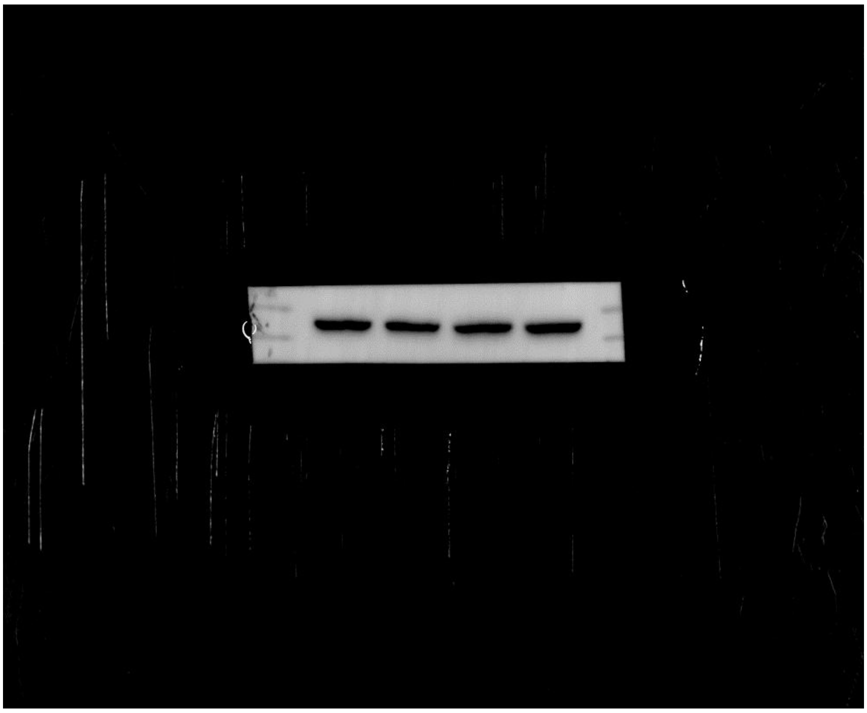

FigureS7H

IP-Flag

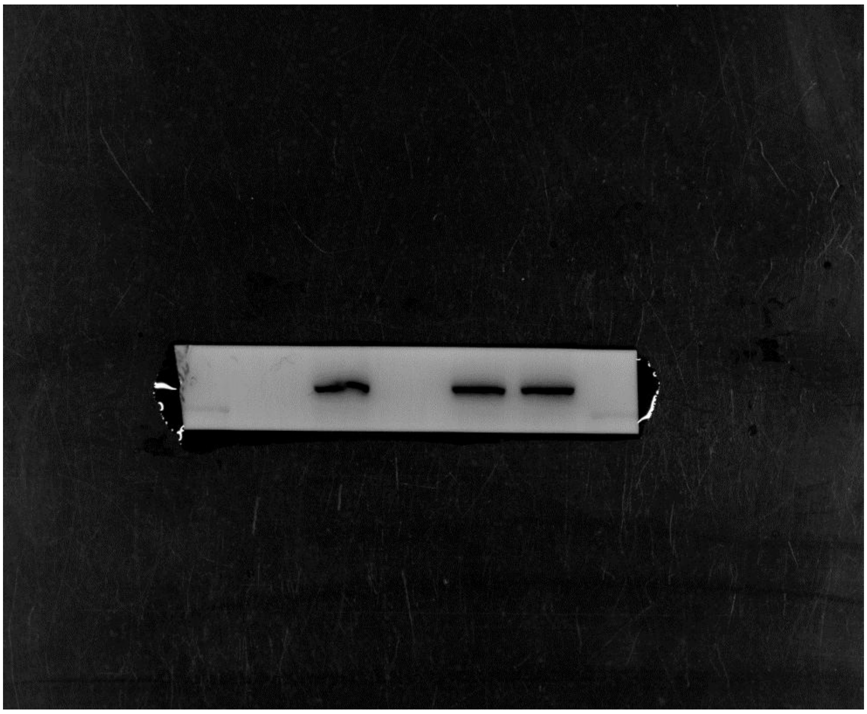

IP-HA

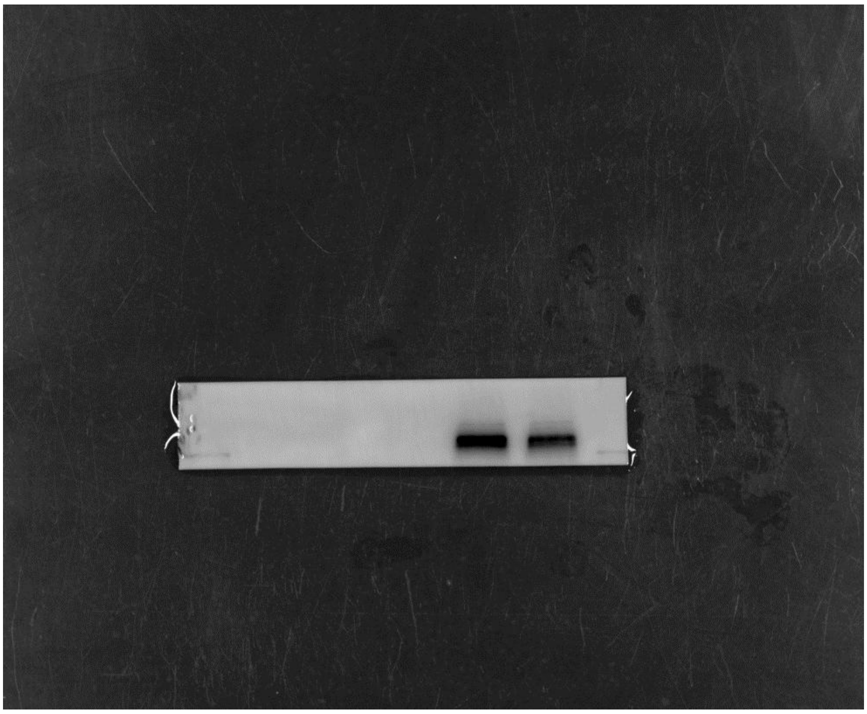

Input-Flag

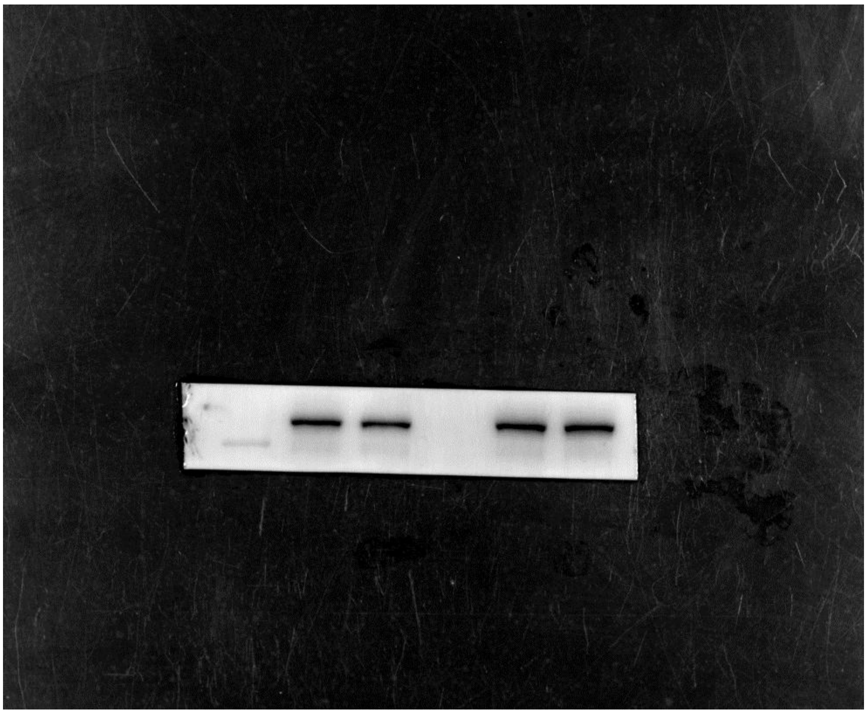

Input-HA

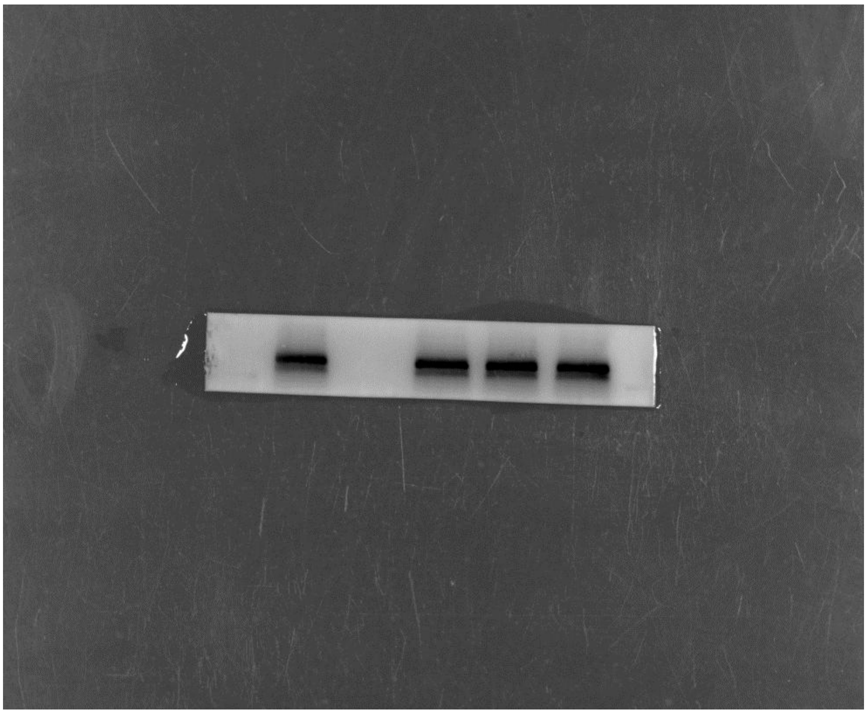

FigureS7L

IP-HA

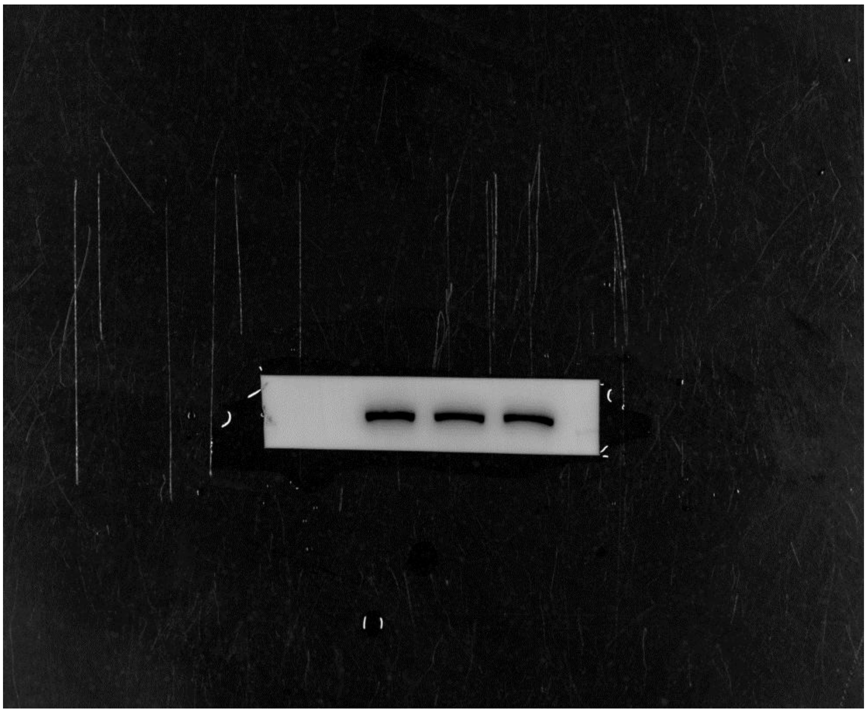

IP-Kla

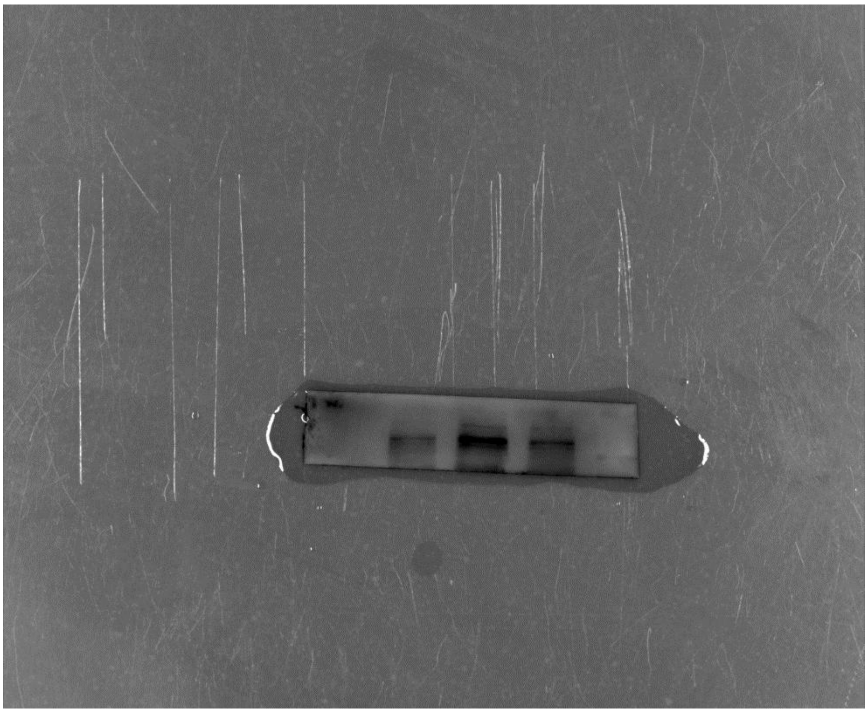

Input-HA

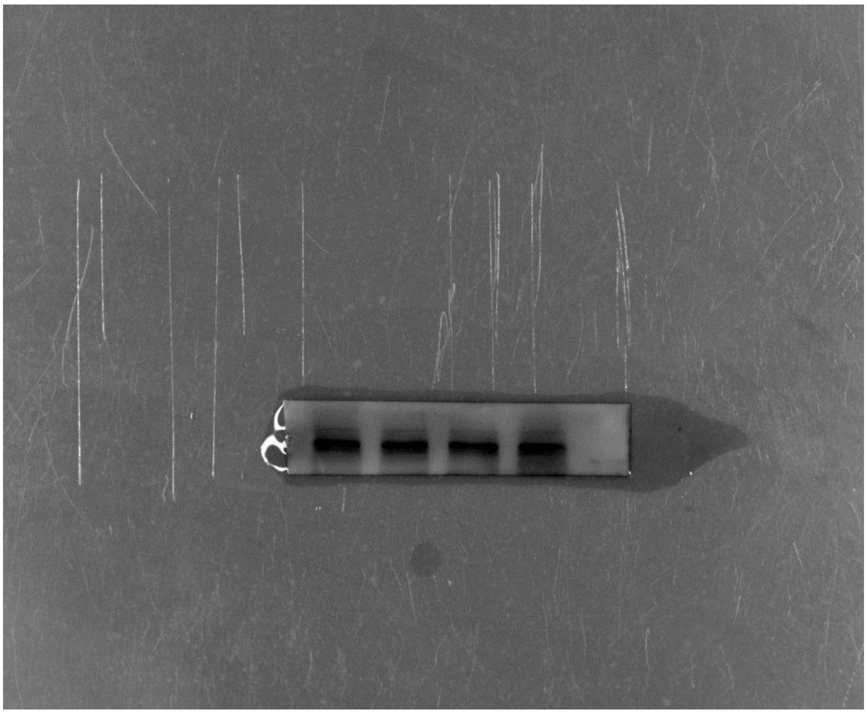

Input-Flag

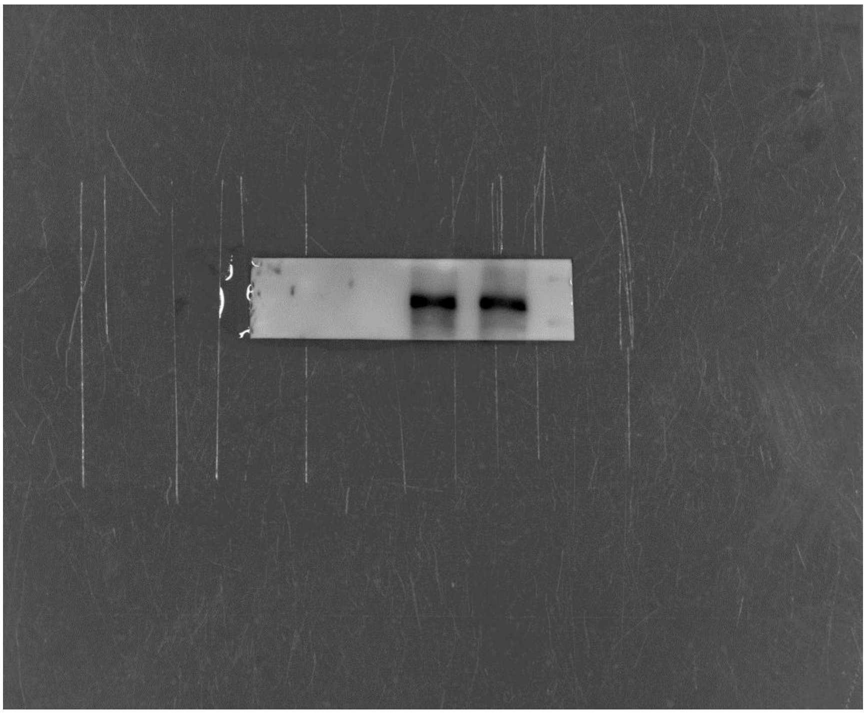

FigureS7M

IP-HA

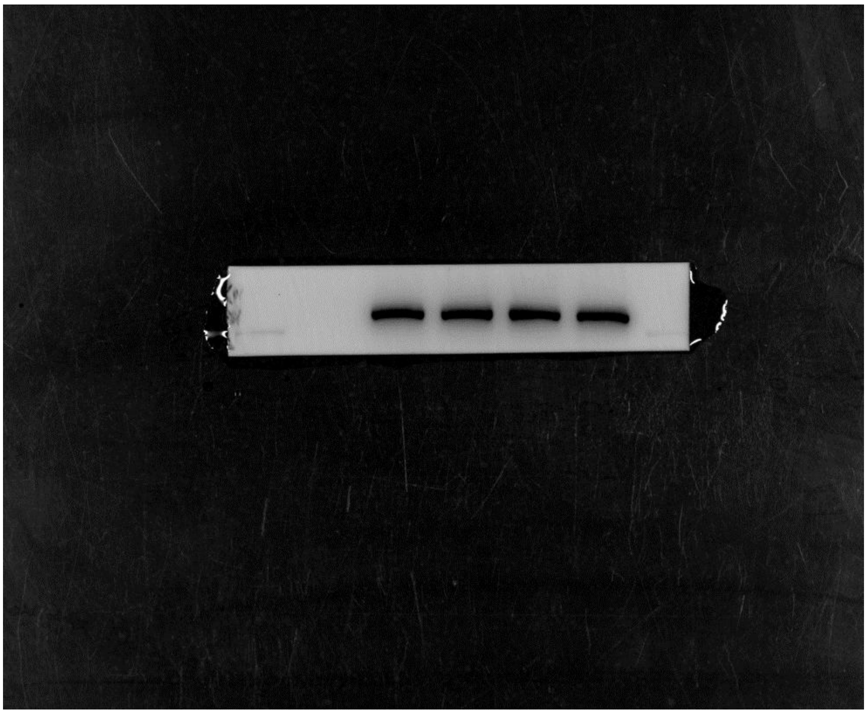

IP-DNA2

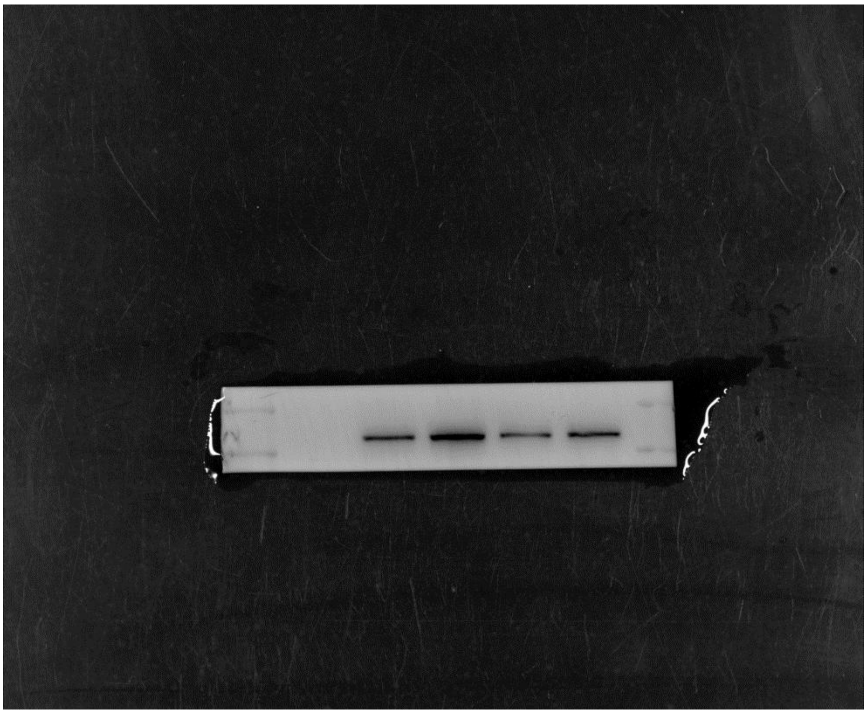

IP-TOPIIA

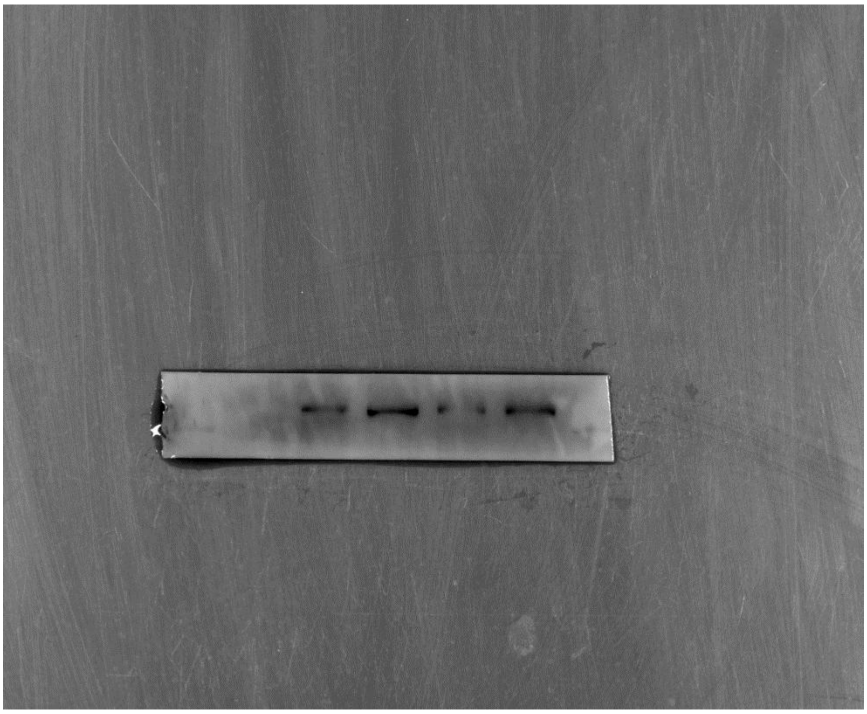

IP-RPA

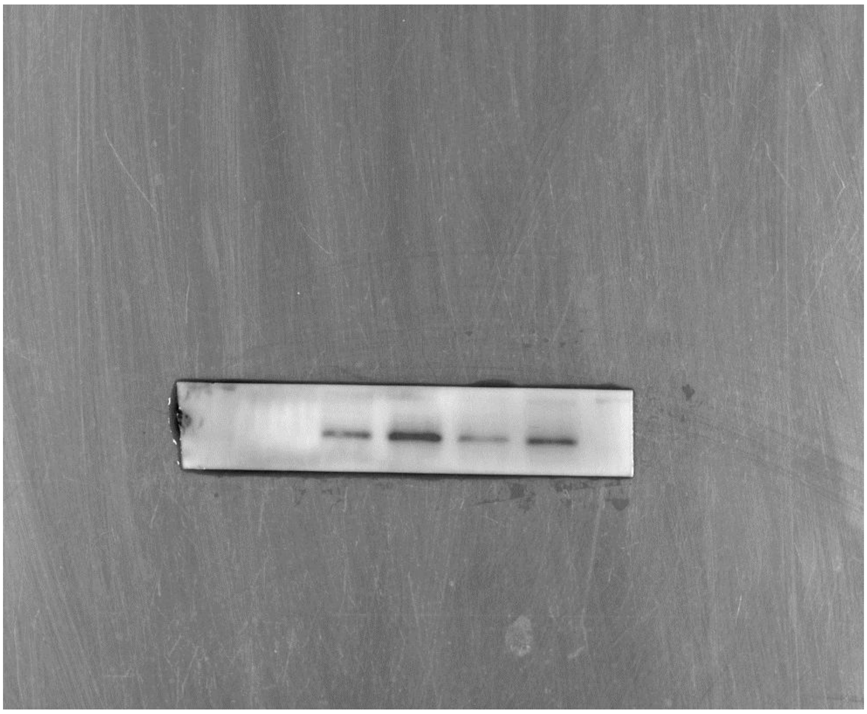

Input-HA

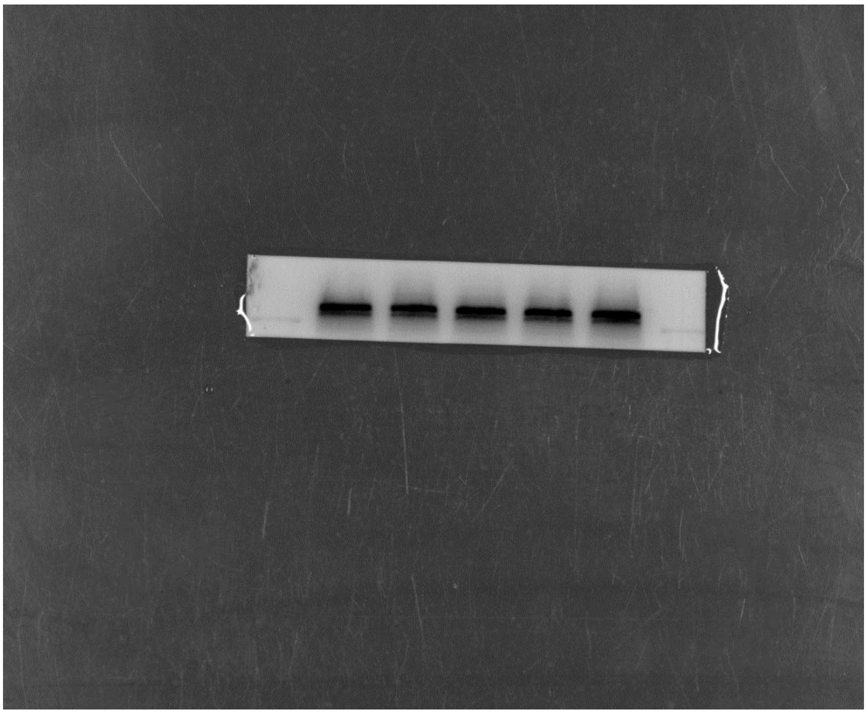

Input-DNA2

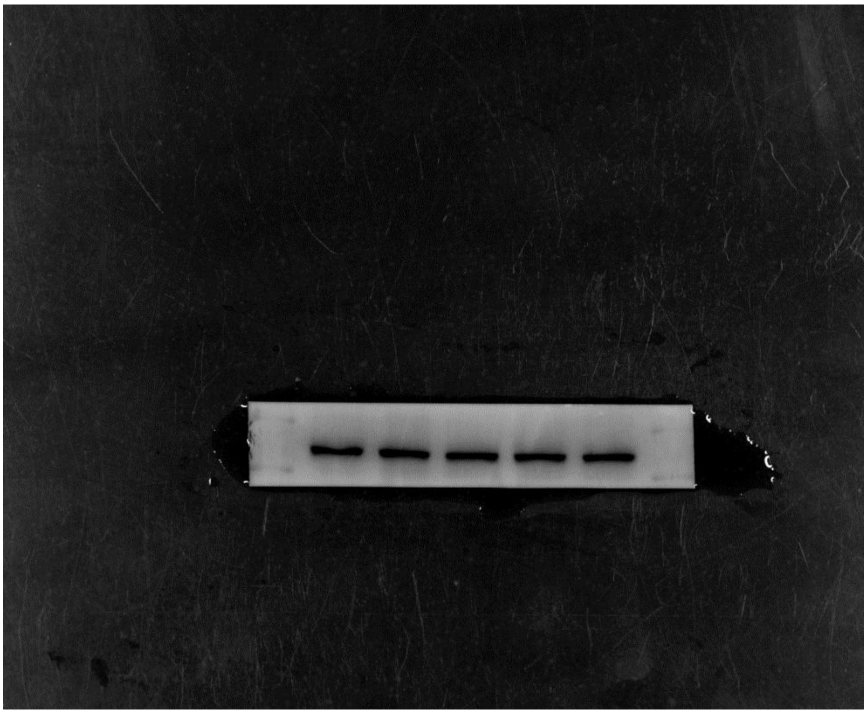

Input-TOPIIA

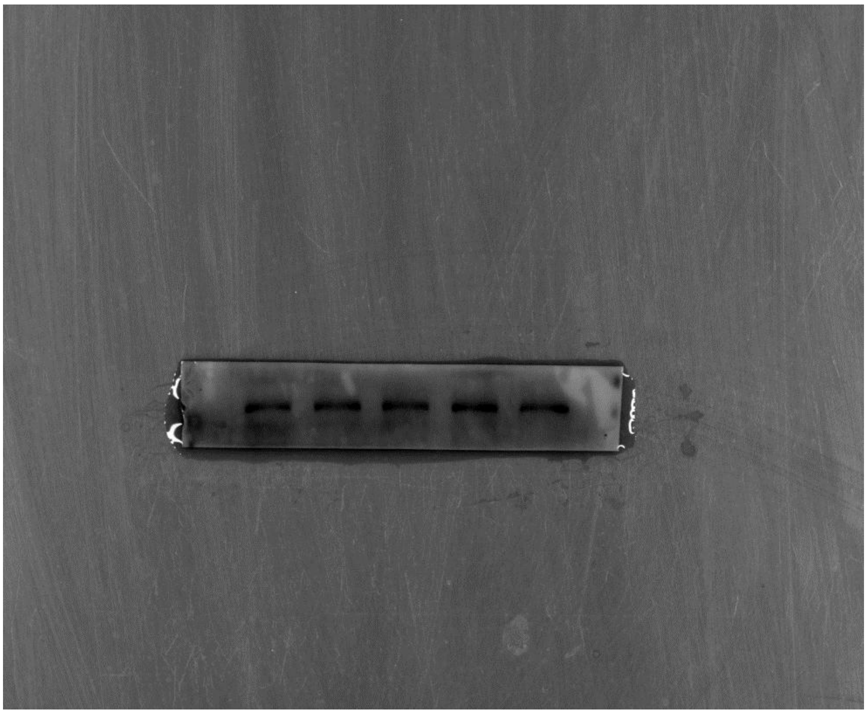

Input-RPA

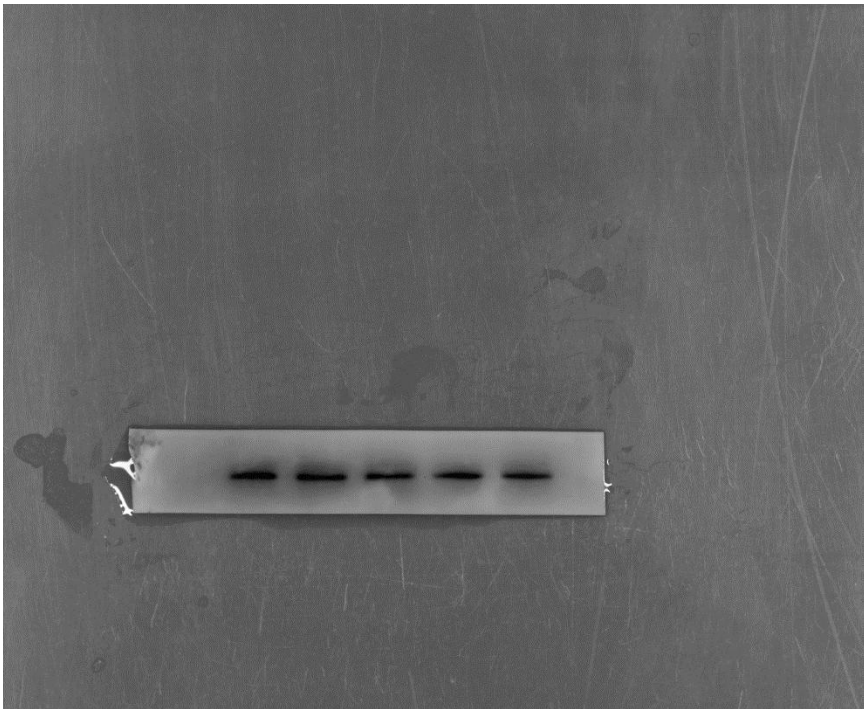

FigureS7N

IP-HA

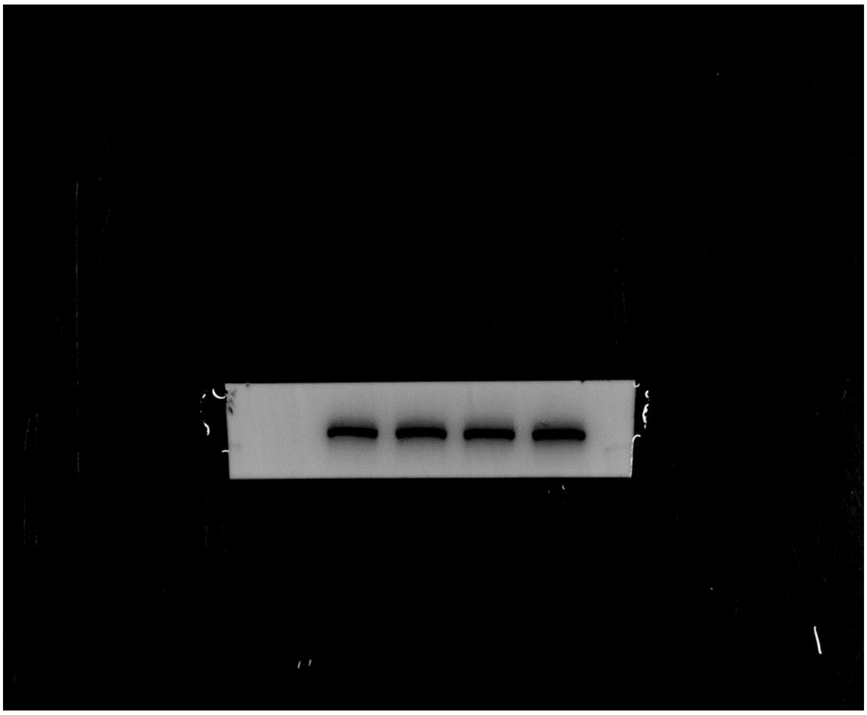

IP-Flag

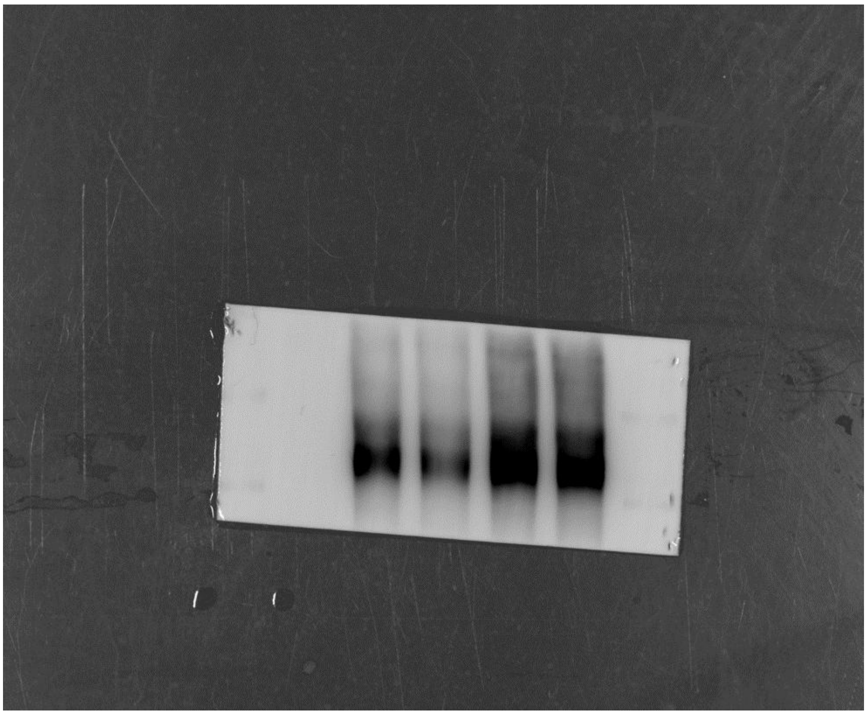

Input-HA

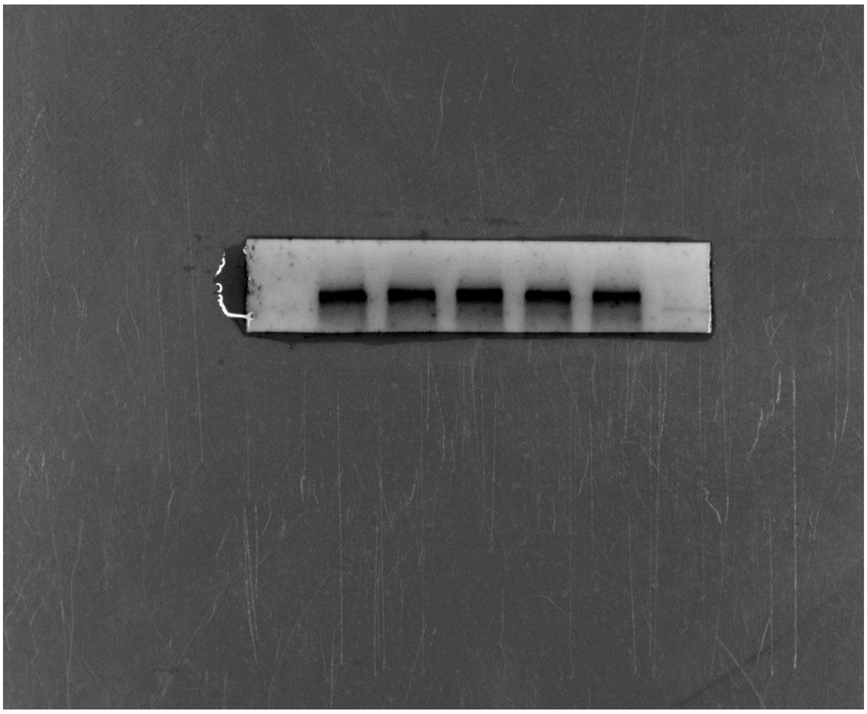

FigureS8F

BLM-K24la

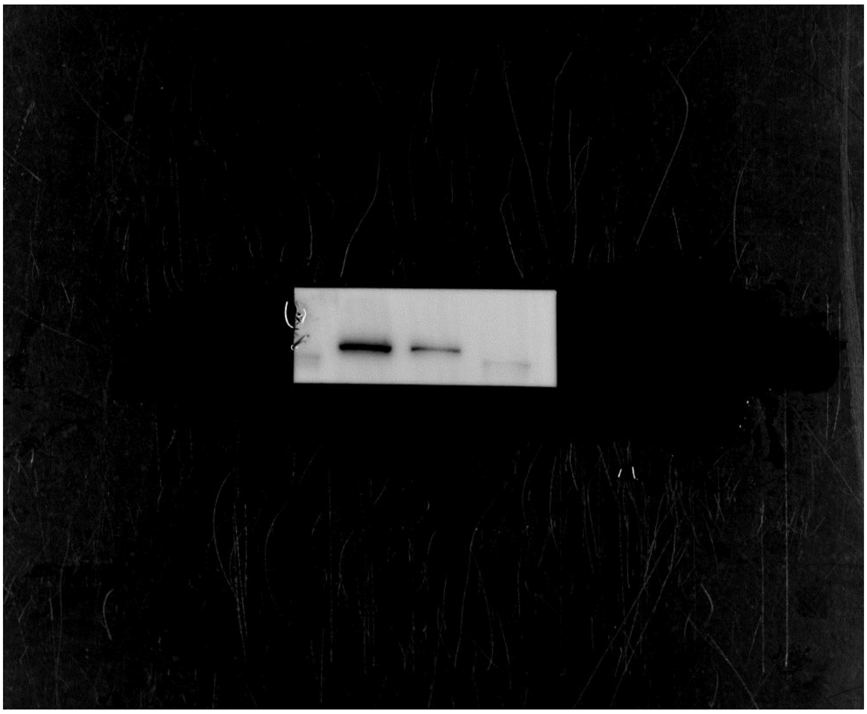

β-actin

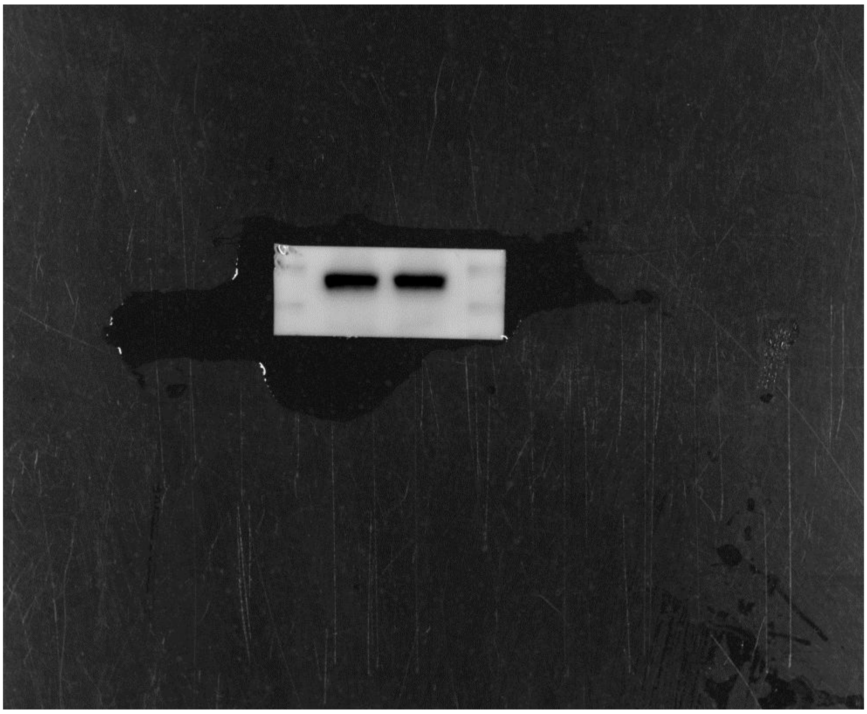

FigureS8G

BLM-K24la

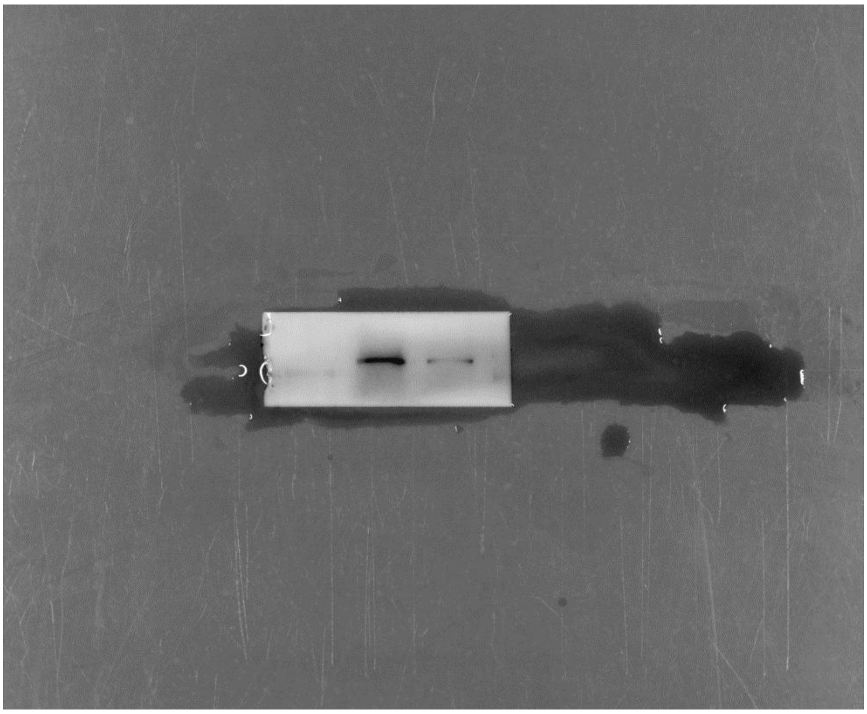

β-actin

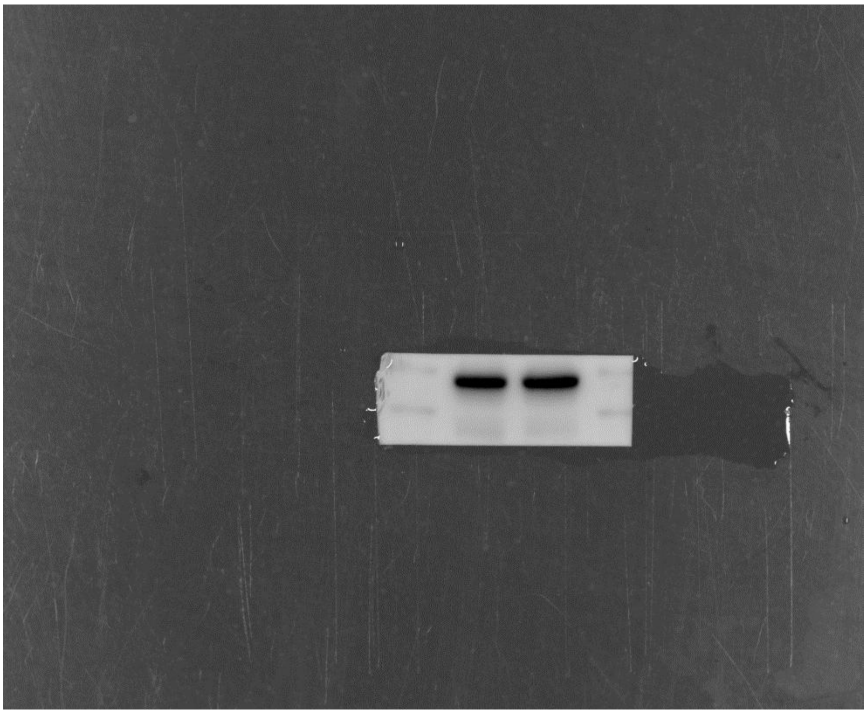

FigureS8L

TOPI

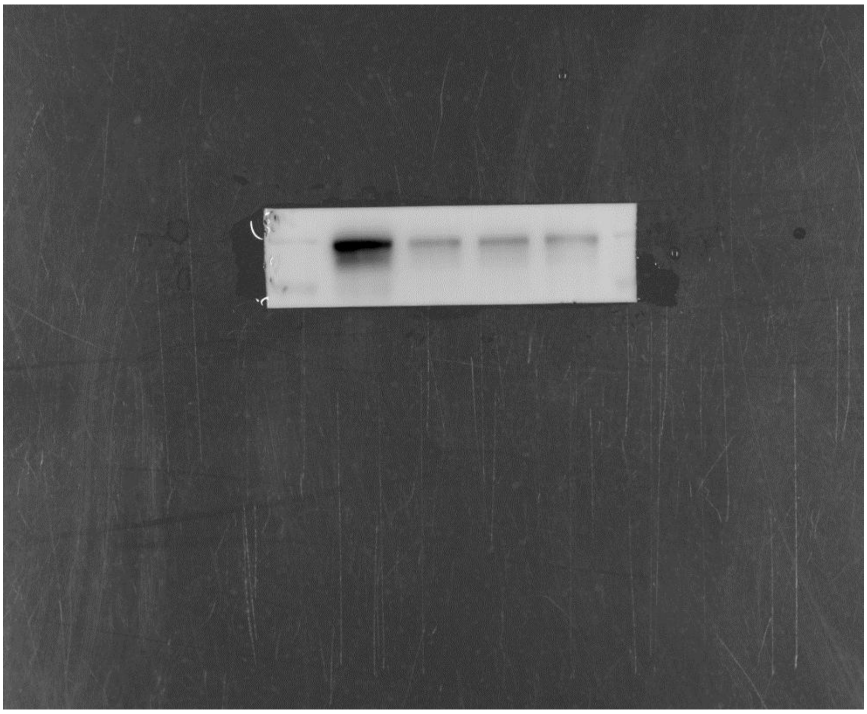

β-actin

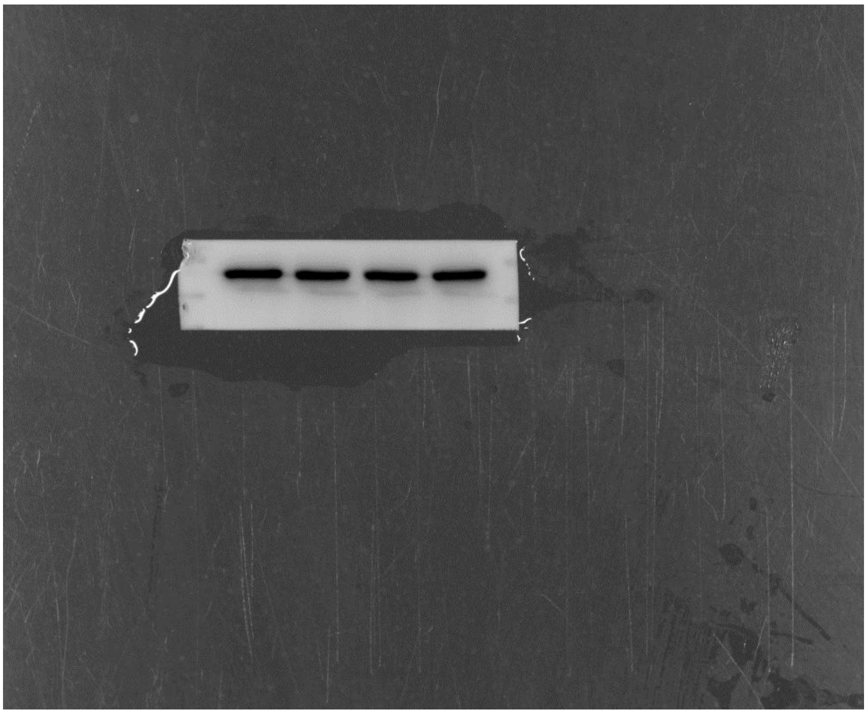

FigureS8M

IP-HA

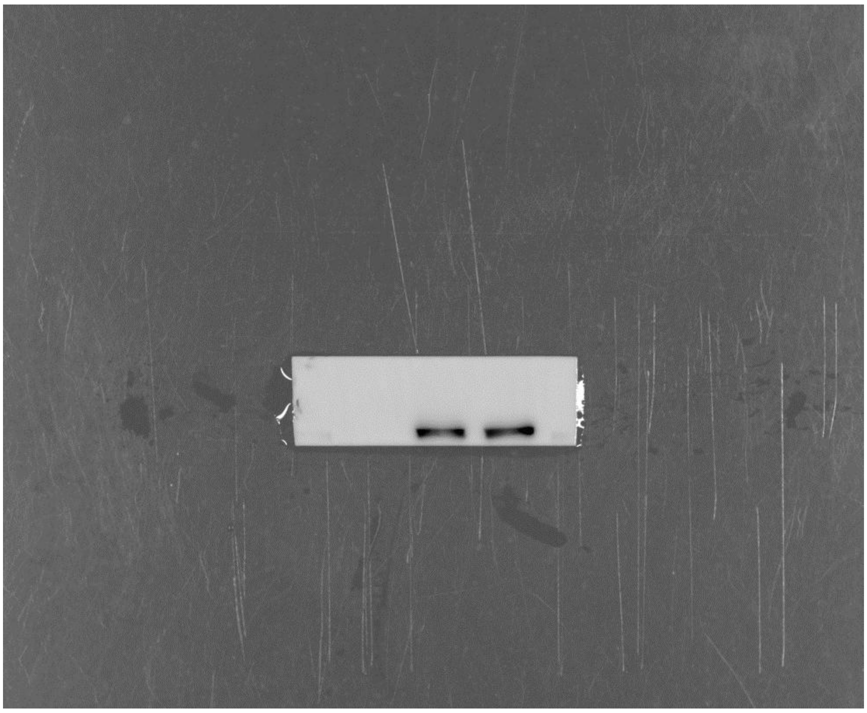

IP-Kla

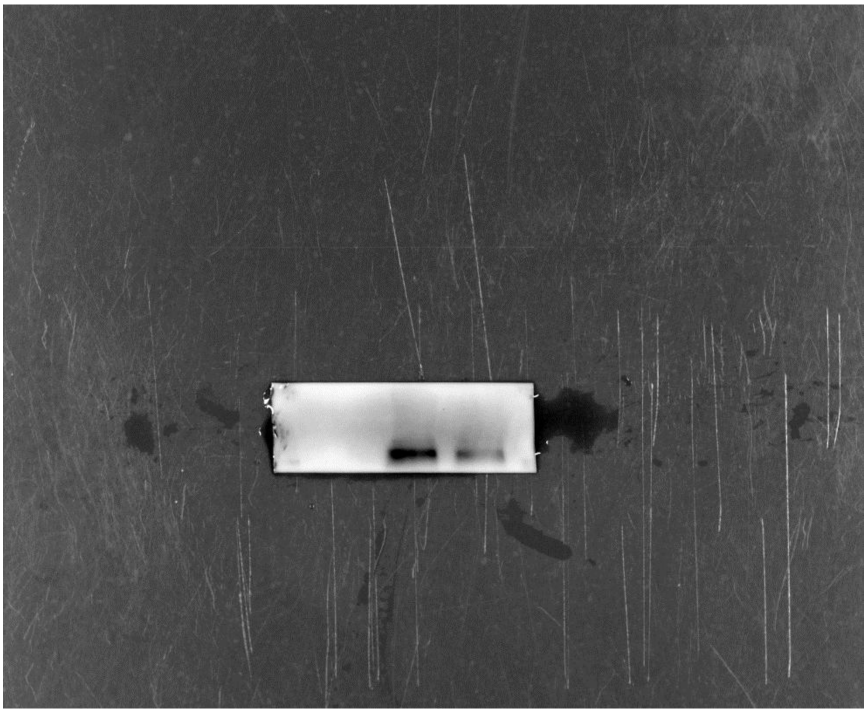

Input-HA

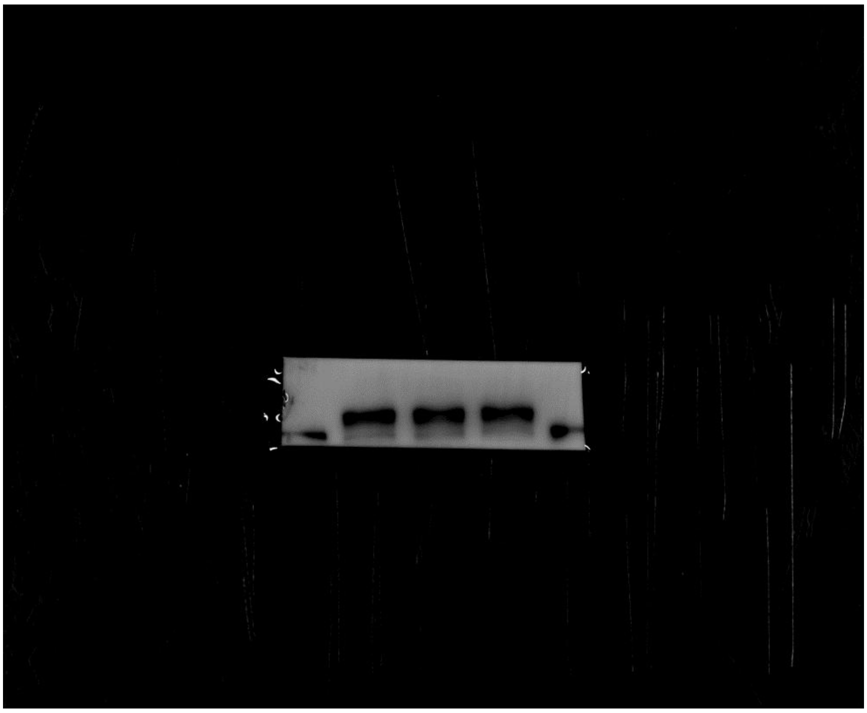

FigureS8O

HA

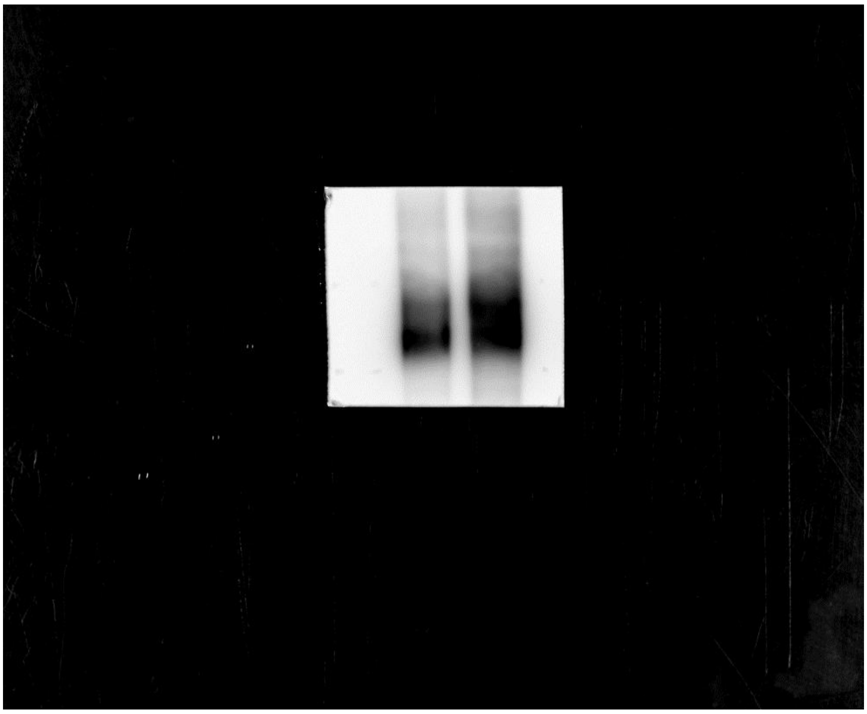

His

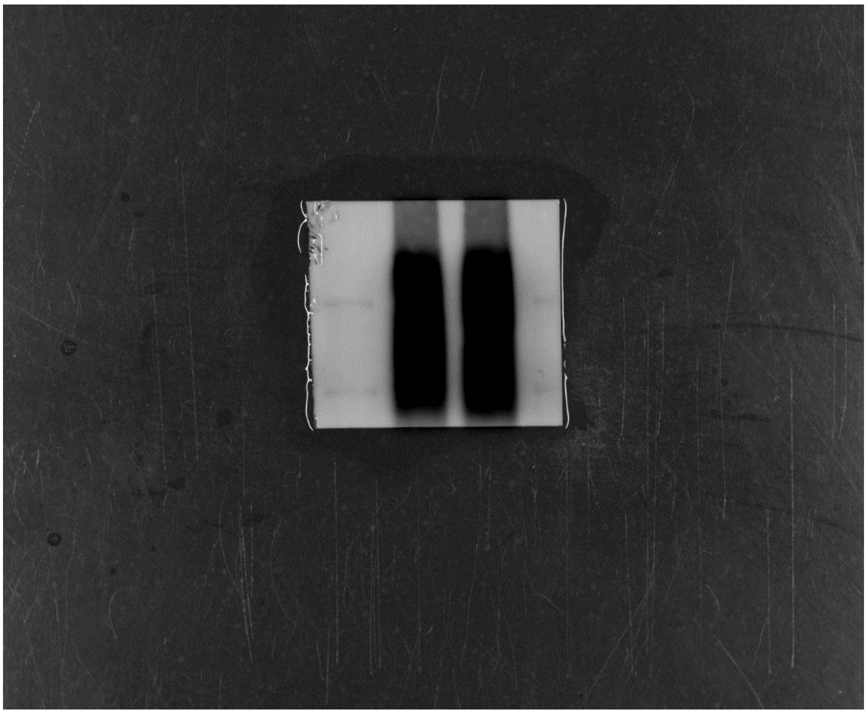

Input-HA

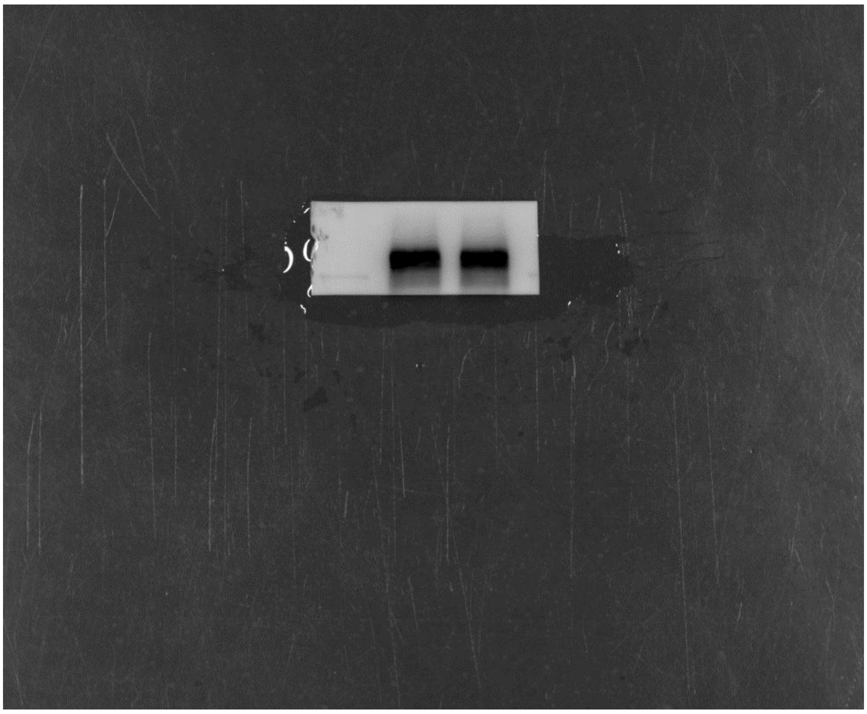

FigureS9D

RAD51

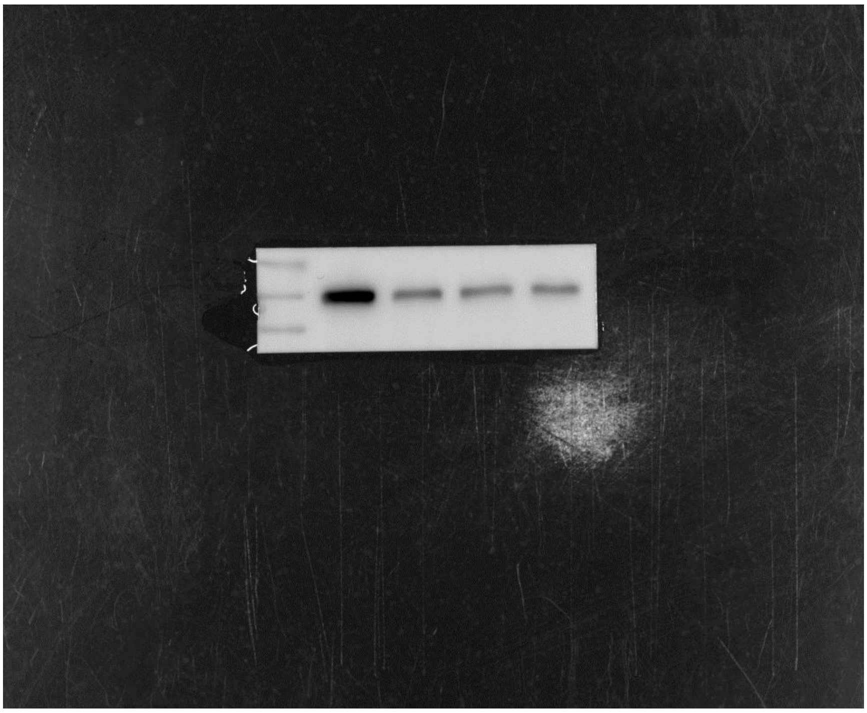

H3

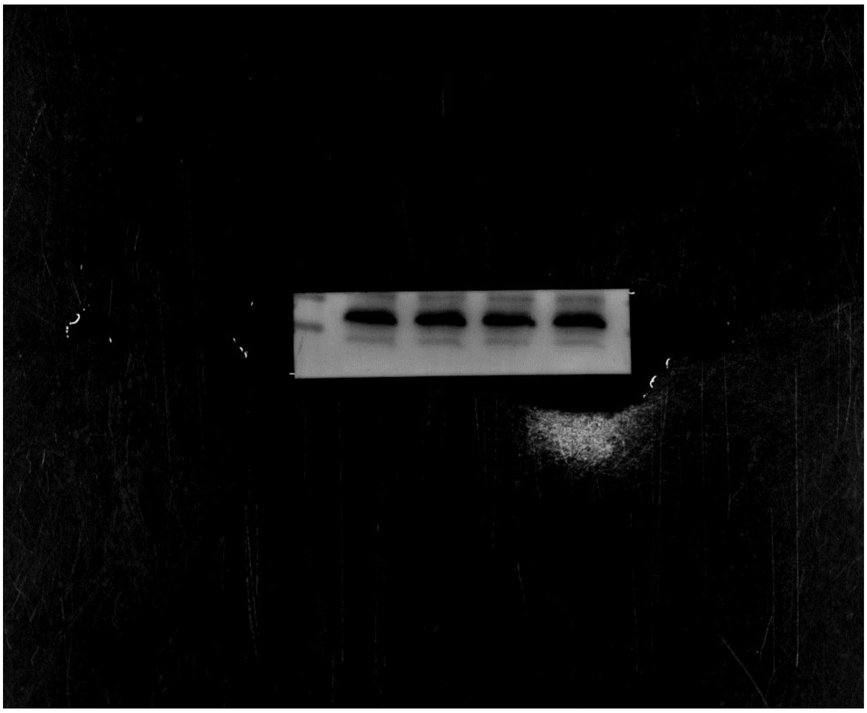

γH2AX

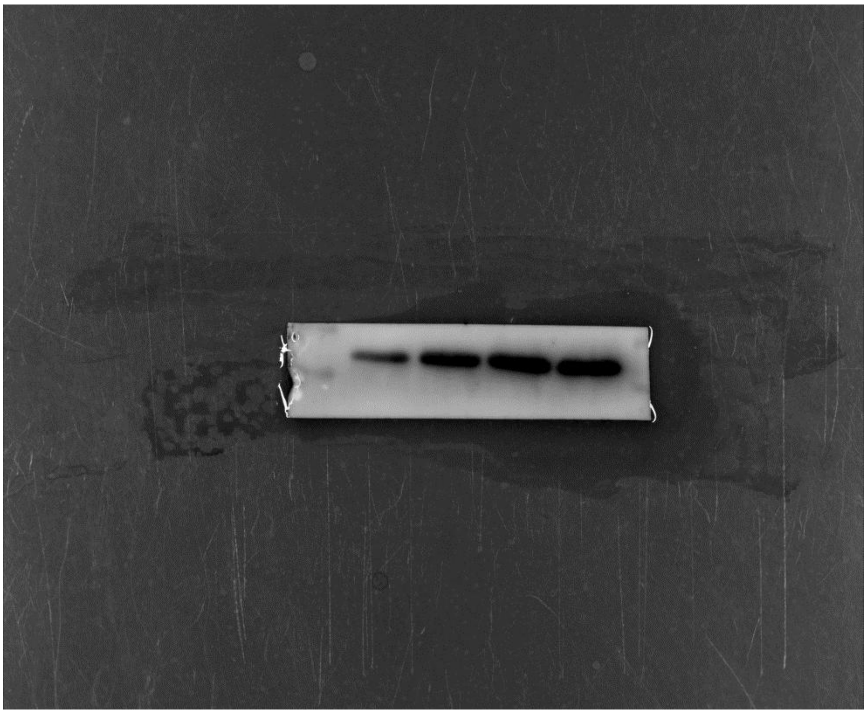

β-actin

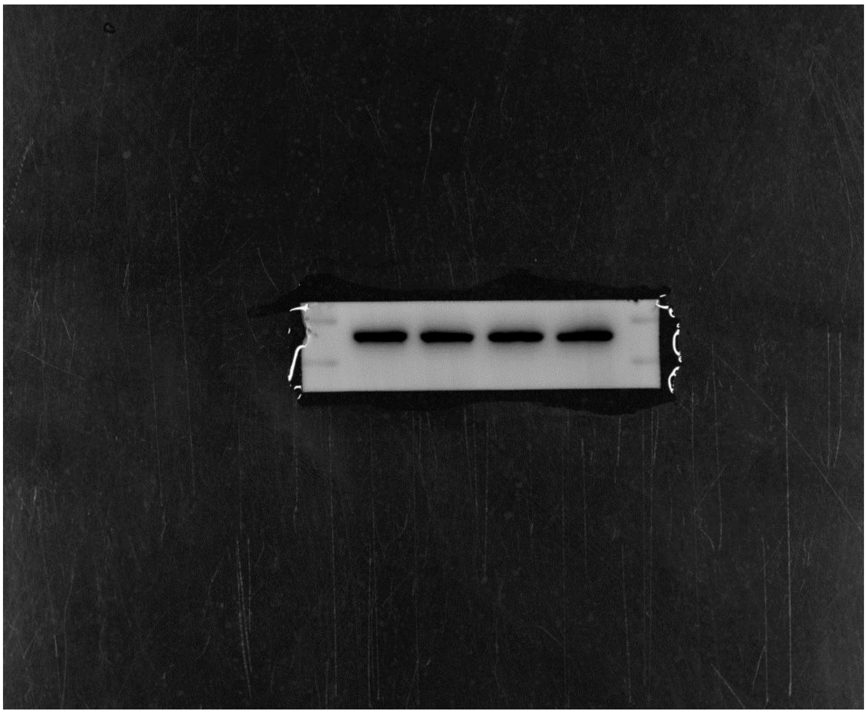

Supplement: Supplementary file 2 — Data S1 [file 41392_2025_2302_MOESM2_ESM.pdf]
