# Supplementary material for: Irinotecan alleviates chemoresistance to anthracyclines through the inhibition of AARS1-mediated BLM lactylation and homologous recombination repair
Source: Signal Transduct Target Ther. 2025 Jul 10;10:214. doi: 10.1038/s41392-025-02302-y (PMC12241633; doi:10.1038/s41392-025-02302-y)
Supplement: Supplementary file 4 — Study protocol [file 41392_2025_2302_MOESM4_ESM.docx]

**A Phase I, Open-Label Clinical Study of Irinotecan Liposomes Combined with Epirubicin in Recurrent Non-Muscle Invasive Bladder Urothelium Carcinoma after Anthracyclines Treatment**

**Clinical Study Protocol**

**Protocol ID:** 2024-549-02

**Version Number:** 1.0

**Version Date:** December 17, 2024

**Principal Investigator:** Prof. Xin Gou

**Sponsor:** The First Affiliated Hospital of Chongqing Medical University

**Protocol Signing Page**

**Statement of the Principal Investigator**

I will conscientiously perform my duties as an investigator according to guidelines for Good Clinical Practice (GCP) in China, and participate in or directly guide this clinical study. We have read and confirmed the protocol (Protocol ID: 2024-549-02; version number: 1.0; version date: December 17, 2024). I agree to perform my duties in accordance with Chinese law, the Declaration of Helsinki, the Chinese GCP, and the study protocol. I will make modifications to the protocol only after informing the sponsor and with the approval of the ethics committee unless measures are necessary to protect the safety, rights, and interests of the subjects.

| **Unit of Clinical Study:** The First Affiliated Hospital of Chongqing Medical University | | |
| --- | --- | --- |
|  | | |
|  |  |  |
| Principal Investigator | Unit of Clinical Study | Signature Date |

**Contents**

[**List of abbreviations 4**](#_Toc200664868)

[**Protocol synopsis 7**](#_Toc200664869)

[**1. Background 11**](#_Toc200664870)

[**1.1 Overview of Bladder Cancer 11**](#_Toc200664871)

[**1.2 Therapeutic Challenges in NMIBC 11**](#_Toc200664872)

[**1.3 Irinotecan Liposome 12**](#_Toc200664873)

[**2. Study Objective and Anticipated Outcomes 13**](#_Toc200664874)

[**2.1 Study Objective 13**](#_Toc200664875)

[**2.2 Anticipated Outcomes 13**](#_Toc200664876)

[**3. Study Endpoints 14**](#_Toc200664877)

[**3.1 Primary Endpoints 14**](#_Toc200664878)

[**3.2 Secondary Endpoints 14**](#_Toc200664879)

[**4. Study design 14**](#_Toc200664880)

[**4.1 Overall design 14**](#_Toc200664881)

[**4.2 Dose Escalation Regimen 15**](#_Toc200664882)

[**4.3 Dose Escalation Principles 15**](#_Toc200664883)

[**4.4 Treatment Period Follow-up 15**](#_Toc200664884)

[**4.5 Safety Evaluation 16**](#_Toc200664885)

[**5. Sample Size 16**](#_Toc200664886)

[**6. Eligibility Criteria 16**](#_Toc200664887)

[**6.1 Inclusion Criteria 16**](#_Toc200664888)

[**6.2 Exclusion Criteria 16**](#_Toc200664889)

[**6.3 Discontinuation Criteria 16**](#_Toc200664890)

[**6.4 Withdrawal Criteria 17**](#_Toc200664891)

[**6.5 Termination Criteria 17**](#_Toc200664892)

[**7. Recruitment Method 17**](#_Toc200664893)

[**8. Overview of drugs 17**](#_Toc200664894)

[**9. Special Adverse Reaction Management 18**](#_Toc200664895)

[**9.1 Infusion-related reactions 18**](#_Toc200664896)

[**9.2 Diarrhea 19**](#_Toc200664897)

[**10. Concomitant Medications 20**](#_Toc200664898)

[**10.1 Prohibited Medications During the Study 20**](#_Toc200664899)

[**10.2 Medications Requiring Cautious Use During the Study 20**](#_Toc200664900)

[**10.3 Supportive Care 20**](#_Toc200664901)

[**10.4 Nausea and vomiting treatment 20**](#_Toc200664902)

[**11. Administration, distribution and recycling of drugs 21**](#_Toc200664903)

[**12. Study Procedure 21**](#_Toc200664904)

[**12.1 Screening period 21**](#_Toc200664905)

[**12.2 Treatment period 22**](#_Toc200664906)

[**12.3 End-of-treatment and withdrawal study 22**](#_Toc200664907)

[**12.4 Safety Follow-up 23**](#_Toc200664908)

[**12.5 Survival Follow-up 23**](#_Toc200664909)

[**12.6 Unscheduled follow-up 23**](#_Toc200664910)

[**13. Study Assessments 24**](#_Toc200664911)

[**13.1 Efficacy assessment 24**](#_Toc200664912)

[**13.2 Safety Assessment 24**](#_Toc200664913)

[**14. Reporting of Adverse Events 24**](#_Toc200664914)

[**14.1 AEs 25**](#_Toc200664915)

[**14.1.1 Definitions of AEs 25**](#_Toc200664916)

[**14.1.2 AEs grade 25**](#_Toc200664917)

[**14.1.3 Assessment of AE Relationship to Investigational Product 25**](#_Toc200664918)

[**14.2 SAE 26**](#_Toc200664919)

[**14.2.1 Definitions of SAE 26**](#_Toc200664920)

[**14.2.2 Hospitalization Criteria for SAE Reporting 26**](#_Toc200664921)

[**14.3 Collection/follow-up of AE/ SAEs 27**](#_Toc200664922)

[**14.3.1 Collection of AE/SAE 27**](#_Toc200664923)

[**14.3.2 Follow-up of AE/SAE 27**](#_Toc200664924)

[**14.4 Abnormal liver function 27**](#_Toc200664925)

[**14.5 SAE Reporting Procedure 28**](#_Toc200664926)

[**14.6 Disease Progression and Death 28**](#_Toc200664927)

[**14.7 Pregnancy 29**](#_Toc200664928)

[**15. Data Management and Statistical Analysis 29**](#_Toc200664929)

[**15. 1 Documentation of Source Record 29**](#_Toc200664930)

[**15. 2 CRF Design and Completion 29**](#_Toc200664931)

[**15. 3 Monitoring and Auditing of Data 29**](#_Toc200664932)

[**15.4 Recording and Retention of Research Data 29**](#_Toc200664933)

[**15.5 Statistical Analysis 29**](#_Toc200664934)

[**15.5.1 Analysis datasets 29**](#_Toc200664935)

[**15.5.2 Analysis Variables 30**](#_Toc200664936)

[**15.5.3 Statistical Method 30**](#_Toc200664937)

[**16. Quality Control and Quality Assurance 30**](#_Toc200664938)

[**17. Ethical Standards and Informed Consent 31**](#_Toc200664939)

[**References 32**](#_Toc200664940)

List of abbreviations

| **Abbreviations** | **Full term** |
| --- | --- |
| NMIBC | Non-muscle-invasive bladder cancer |
| MIBC | Muscle-invasive bladder cancer |
| DLT | Dose-Limiting Toxicity |
| RFS | Recurrence-Free Survival |
| DCR | Disease Control Rate |
| CR | Complete Response |
| PR | Partial Response |
| SD | Stable Disease |
| PD | Progressive Disease |
| TURBT | Transurethral Resection of Bladder Tumor |
| EPI | Epirubicin |
| ECOG | Eastern Cooperative Oncology Group |
| RECIST | Response Evaluation Criteria in Solid Tumors |
| AE | Adverse event |
| DAS | DLT Analysis Set |
| CMH | Cochran-Mantel-Haenszel |
| DDR | DNA damage repair |
| OS | Overall survival |
| SAE | Serious adverse event |
| TEAE | Treatment-emergent adverse event |
| CRF | Case Report Form |
| NMPA | National Medical Products Administration |
| ASCO | American Society of Clinical Oncology |
| ICF | Informed Consent Form |
| BP | Blood pressure |
| ANC | Neutrophil count |
| LYM | Lymphocyte count |
| RBC | Red blood cell count |
| Hb | Hemoglobin |
| PLT | Platelet count |
| TBIL | Total bilirubin |
| DBIL | Direct bilirubin |
| ALT | Alanine aminotransferase |
| AST | Aspartate aminotransferase |
| AKP | Alkaline phosphatase |
| TP | Total protein |
| ALB | Albumin |
| BUN | Urea/urea nitrogen |
| Cr | Creatinine |
| Ccr | Endogenous creatinine clearance rate |
| GLU | Fasting blood glucose |
| K | Potassium |
| Na | Sodium |
| Cl | Chlorine |
| Ca | Calcium |
| P | Phosphorus |
| CK | Creatine Kinase |
| CK-MB | Creatine Kinase-MB |
| cTnT | Troponin |
| INR | International Normalized Ratio |
| APTT | Activated Partial Thromboplastin Time |
| PT | Prothrombin Time |
| FIB | Fibrinogen |
| ULN | Upper limit of normal |
| SDV | Source Data Verification |
| FAS | Full Analysis Set |
| PPS | Per-protocol set |
| SS | Safety analysis set |

Protocol synopsis

| Study Drug | Irinotecan Liposomes |
| --- | --- |
| Protocol Title | A Phase I, Open-Label Clinical Study of Irinotecan Liposomes Combined with Epirubicin in Recurrent Non-Muscle Invasive Bladder Urothelium Carcinoma after Anthracyclines Treatment |
| Protocol ID | 2024-549-02 |
| Version Number | 1.0 |
| Version Date | December 17, 2024 |
| Sponsor | The First Affiliated Hospital of Chongqing Medical University |
| Study Design | A Prospective, single-center, single-arm Phase I clinical study |
| Study Nature | An investigator-initiated clinical study |
| Study Population | Patients with recurrence of bladder urothelial carcinoma after anthracycline treatment |
| Sample Size | An estimated 6-9 participants will be recruited per arm, yielding a total target enrollment of 12-18 participants across both arms. |
| Objective | 1. To explore the safety profile (including dose-limiting toxicity), efficacy, and tolerability of neoadjuvant Irinotecan liposome combined with Epirubicin in non-muscle invasive bladder cancer (NMIBC) patients with anthracycline-resistant recurrent disease. 2. To explore the epigenetic mechanism by which Irinotecan liposome target and inhibit DNA homologous recombination repair to reverse anthracycline chemotherapy resistance in bladder cancer |
| Endpoints | 1. Primary Endpoints:  Incidence and frequency of Dose-Limiting Toxicity (DLT)  2. Secondary Endpoints:  (1) Adverse drug reactions;  (2) Recurrence-Free Survival (RFS);  (3) Disease Control Rate (DCR);  3. Tumor Response Evaluation (per RECIST 1.1 criteria):   - Complete Response (CR): Disappearance of all target lesions; no new lesions. - Partial Response (PR): ≥30% decrease in the sum of diameters of target lesions. - Stable Disease (SD): Neither sufficient shrinkage to qualify for PR nor sufficient increase to qualify for PD. - Progressive Disease (PD): ≥20% increase in the sum of diameters of target lesions or appearance of new lesions. |
| Study Procedures | 1. Arm A: TURBT unresectable  If the recurrent tumor can't be excised by TURBT, participants will be assigned to this arm. Participants in this arm will complete the neoadjuvant therapy with Irinotecan liposome (intravenous injection, once every two weeks for 1 month, dose increasing regimen: 37.6 mg/m^2^ and 56.5mg/m^2^) and Epirubicin (intravesical instillation, once a week for 1 month, 50 mg), and the therapeutic effect was evaluated by pelvic enhanced MRI and cystoscopy. Judged by two senior urologists, patients with complete response and partial response were treated with TURBT and intravesical therapy. Patients with stable disease or progressive disease received subsequent therapy at the investigator's discretion. Participants will visit the clinic once every 2 weeks for checkups and tests.  2. Arm B: TURBT resectable  If the recurrent tumor can be excised by TURBT, participants will be assigned to this arm (Arm B). Participants in this arm will complete TURBT with immediate intravesical instillation of Epirubicin (50 mg) within 24 hours after surgery. During the induction phase (the first 1 month after surgery), participants will receive the combined therapy with irinotecan liposome (intravenous injection, once every two weeks for 1 month, dose increasing regimen: 37.6 mg/m^2^ and 56.5mg/m^2^) and Epirubicin (intravesical instillation, once a week for 1 month, 50 mg). In the next 6 months, participants will continue the intravesical instillation treatment with Epirubicin (once a month, 50 mg). Participants will visit the clinic once every 2 weeks for checkups and tests in the first month, and once every 3 months after the first month. |
| Inclusion Criteria | Subjects can participate in the study only if all the following criteria are met:   1. Patients with cTa-cT1N0M0 non-muscle invasive bladder cancer (NMIBC); 2. Disease recurrence following intravesical anthracycline-based chemotherapy; 3. Recurrent tumors confirmed as cTa-cT1N0M0 bladder urothelial carcinoma; 4. No prior systemic chemotherapy exposure; 5. Measurable lesions per Response Evaluation Criteria in Solid Tumors (RECIST); 6. Eastern Cooperative Oncology Group (ECOG) performance status 0-1. |
| Exclusion Criteria | Subjects who meet any of the following criteria are not eligible to enter the study:   - 1. Age <18 years;   2. Subjects with severe cardiac, cerebral, hepatic, or renal dysfunction;   3. Subjects with severe malnutrition;   4. Subjects with psychiatric disorders or those lacking insight/unable to provide accurate expression;   5. Concurrent malignancies in other organs;   6. Presence of systemic infectious diseases. |
| Discontinuation Criteria | Discontinuation of study treatment does not equate to study withdrawal. Participants who discontinue study treatment must continue to complete all remaining study visits as required by the protocol. Study treatment must be discontinued if a participant meets any of the following criteria:  1. The study participant withdraws informed consent and refuses to continue receiving the investigational drug treatment;  2. The investigator determines that the participant's clinical symptoms have worsened/performance status has deteriorated;  3. Disease progression is confirmed per RECIST v1.1 criteria;  4. Despite dose modification, the participant remains unable to tolerate adverse events, including any clinical AEs, laboratory abnormalities, or other medical conditions;  5. The participant commits a major protocol violation or demonstrates non-compliance with medical instructions;  6. Occurrence of pregnancy, loss to follow-up, or death of the participant;  7. Other circumstances where the investigator deems it necessary to discontinue the investigational drug treatment. |
| Withdrawal Criteria | Reasons for Study Withdrawal:  1. Withdrawal of informed consent - Participant refuses further study participation and follow-up.  2. Lost to follow-up  3. Death of participant  4. Study termination  5. Other investigator-determined reasons for withdrawal |
| Termination Criteria | The study may be prematurely terminated or temporarily suspended based on the following criteria:  1. Identification of unexpected, significant, or unacceptable risks to study participants  2. Discovery of critical protocol flaws during trial execution |
| Safety | Any adverse events (AEs) that occurred in all subjects during the clinical study period will be observed, including abnormal clinical symptoms and vital signs, and abnormalities in laboratory tests. The clinical characteristics, severity, time of occurrence, duration, treatment methods, and prognosis of adverse events will be recorded; meanwhile, the correlation between adverse events and the study drugs will be determined. The safety of drugs is evaluated according to National Cancer Institute's Common Terminology Criteria for Adverse Events (NCI-CTC AE) version 5.0. |
| Statistical Analysis | For the primary endpoint, the DLT Analysis Set (DAS) included all participants who were in the DLT evaluation period and either completed the DLT assessment or experienced a DLT during the evaluation period. This analysis set will be used for the analysis and summary of DLT events.  For Secondary Endpoints, such as RFS, the median value and 95% CI are estimated and survival curves are obtained using the Kaplan-Meier method. The log-rank test is used to compare the survival between the two groups. DCR with 95% CI are calculated, and comparisons between treatment groups are performed using a Cochran-Mantel-Haenszel (CMH) test or chi-square test. Two independent samples will be compared for the differences between the two groups in QoL scores by the t-test or Wilcoxon rank sum test. |
| Study Schedule | Enrollment date of the first subject: January 2025  Estimated date of the primary completion date: April 2025  Estimated date of the end of the study: December 2025 |

1. Background

## 1.1 Overview of Bladder Cancer

Bladder cancer has emerged as one of the most common genitourinary malignancies in China, currently ranking as the eighth most frequently diagnosed cancer among Chinese male populations. With its characteristically high incidence, frequent recurrence patterns, and substantial mortality burden, this disease represents a major public health challenge in contemporary China [1]. Among histological subtypes, non-muscle invasive bladder cancer (NMIBC) constitutes approximately 75% of incident cases at initial diagnosis, representing the predominant clinical presentation. This form demonstrates particularly aggressive recurrence patterns, with published 5-year recurrence rates ranging from 31% to 78% across various clinical series [2]. The substantial recurrence risk, coupled with potential progression to muscle-invasive disease, underscores the critical need for effective therapeutic interventions. To mitigate recurrence and disease progression, adjuvant intravesical instillation therapy following transurethral resection of bladder tumor (TURBT) has been established as the standard treatment regimen for NMIBC.

## 1.2 Therapeutic Challenges in NMIBC

The standard care for NMIBC involves Transurethral resection (TURBT) followed by adjuvant intravesical therapy. Among chemotherapeutic options, anthracycline-based chemotherapeutic agents, particularly Epirubicin, have emerged as a first-line intravesical chemotherapy drugs for bladder cancer due to their potent antitumor activity and favorable safety profile. These agents are also widely used in treating other malignancies such as breast cancer and gastric cancer [3]. The mechanism is to inhibit topoisomerase II activity, thereby blocking DNA replication and transcription to suppress tumor cell proliferation. In addition, Epirubicin can intercalate between DNA base pairs, inducing DNA double-strand breaks and promoting tumor cell apoptosis [4]. Although bladder cancer cells demonstrate initial sensitivity to first-line chemotherapeutic agents like Epirubicin, the majority of patients develop varying degrees of acquired resistance within several years, resulting in elevated postoperative recurrence rates, increased tumor aggressiveness upon recurrence and the greater technical difficulty in surgical resection of recurrent lesions. Therefore, addressing this acquired chemoresistance represents a critical therapeutic challenge in NMIBC management.

Tumor heterogeneity represents one of the greatest challenges in contemporary cancer therapeutics. Emerging evidence from multiple studies has demonstrated that epigenetic alterations in tumors serve as a pivotal mechanism underlying therapy resistance and poor clinical outcomes in cancer patients [5-7]. Under drug selection pressure, tumor cells adapt to their microenvironment through a fundamental remodeling process spanning multiple molecular levels---including DNA, RNA, and protein networks. For instance, tumor subclones harboring specific driver mutations may acquire selective advantages over neighboring cells, ultimately driving clonal evolution and conferring therapeutic resistance. Multiple post-hoc analyses of tissue specimens from prior bladder cancer chemotherapy trials have demonstrated that mutations in DNA damage repair (DDR) pathway genes and other molecular markers exhibit predictive value for treatment efficacy in muscle-invasive bladder cancer (MIBC) [8-10].

Interestingly, in the bladder cancer cohort treated with Epirubicin infusion chemotherapy initiated by the collaborating unit, we conducted multi-omics testing on tissue specimens before and after treatment. Whole-exome sequencing results showed that the mutation rate of DDR-related genes was significantly increased in the tumor tissues of drug-resistant patients. Further modification omics analysis suggested that the lactylation modification levels of several key enzymes for DNA homologous recombination repair, such as BLM, RAD51, and MER11, were significantly up-regulated in drug-resistant patient tumor cells. Transcriptome sequencing analysis found that differentially expressed genes between tumors of patients without recurrence within 1 year and those of recurrent patients were enriched in multiple DDR-related signaling pathways such as homologous recombination repair and DNA metabolism. We further conducted a retrospective analysis of NMIBC patients resistant to anthracycline chemotherapy, and found that activation of homologous recombination repair also indicated anthracycline resistance. Then, clinically, can the reversal of Epirubicin chemotherapy resistance be achieved by inhibiting the homologous recombination repair pathway in these patients?

In our preliminary experiments, we observed significantly elevated levels of lactylation modification on BLM, a crucial helicase involved in DNA homologous recombination repair, in both EPI-resistant bladder cancer tissues and in vitro induced EPI-resistant bladder cancer cell lines. Inhibition of lactylation modification at the K24 site of BLM effectively reversed EPI resistance. Through molecular docking and high-throughput small molecule screening, we identified that irinotecan can bind to the BLM-K24 drug-binding pocket, thereby reducing BLM lactylation and enhancing EPI chemosensitivity. Further validation using EPI-resistant bladder cancer and breast cancer PDX models and organoid models demonstrated that the combination of irinotecan and EPI successfully reversed EPI resistance without inducing additional drug toxicity in mouse models.

## 1.3 Irinotecan Liposome

Irinotecan is a semi-synthetic derivative of camptothecin that specifically binds to topoisomerase I, inducing reversible single-strand breaks and consequently unwinding the DNA double-helix structure. Both irinotecan hydrochloride and its active metabolite SN-38 can bind to the topoisomerase I-DNA complex, thereby preventing the religation of the broken single strands [11, 12]. Recent structure-activity relationship studies have demonstrated that the lactone ring in the structure of camptothecin drugs such as irinotecan is an essential group for antitumor activity. However, under physiological conditions, it rapidly hydrolyzes into an inactive open-ring form, thereby limiting the antitumor efficacy [13, 14]. In contrast, liposome-based microvesicular drug delivery systems offer unique advantages, including enhanced drug solubility, controlled release properties, improved stability, and superior tumor-targeting capabilities. These features are of significant importance in potentiating the antitumor effects, reducing toxicity, and improving the bioavailability of chemotherapeutic agents [15, 16]. Irinotecan hydrochloride liposomal injection is a novel formulation developed by modifying the existing marketed irinotecan hydrochloride injection and its lyophilized powder. This reformulation enables passive tumor targeting, protecting irinotecan from premature conversion to its active metabolite, SN-38. Consequently, it prolongs the drug’s circulation time, enhances its accumulation and retention within tumor tissues, and strengthens its tumor growth inhibitory effects. This mechanism of action has been evaluated and validated in clinical studies. The global multicenter, randomized controlled phase III NAPOLI-1 trial published in The Lancet and its Asian subgroup analysis reported in Cancer Science demonstrated that for metastatic pancreatic cancer patients who failed prior gemcitabine-based chemotherapy, liposomal irinotecan combined with 5-FU/LV significantly improved overall survival (OS) with manageable toxicity, showing particularly enhanced survival benefits in the Asian population [13, 15, 16]. These preclinical findings suggest liposomal irinotecan may emerge as a key intervention for overcoming chemotherapy resistance in bladder cancer by inhibiting homologous recombination repair.

Building upon this scientific foundation and preliminary research findings, the current project proposes to conduct a prospective single-arm Phase I clinical study investigating combination therapy with Irinotecan Liposome and Epirubicin for NMIBC patients with anthracycline-resistant recurrent disease. This study is expected to provide a novel, highly effective and low-toxicity therapeutic option for reducing post-anthracycline chemotherapy recurrence and improving treatment outcomes in recurrent tumors, thereby offering a new precision medicine approach for bladder cancer.

# 2. Study Objective and Anticipated Outcomes

## 2.1 Study Objective

(1) To explore the safety profile (including dose-limiting toxicity (DLT)), efficacy, and tolerability of neoadjuvant liposomal irinotecan combined with Epirubicin in NMIBC patients with anthracycline-resistant recurrent disease.

(2) Building upon prior clinical and mechanistic investigations, this study aims to elucidate the epigenetic mechanisms by which liposomal irinotecan reverses anthracycline chemotherapy resistance in bladder cancer through targeted inhibition of DNA homologous recombination repair.

## 2.2 Anticipated Outcomes

(1) Establish DLT and characterize the safety profile, tolerability, and antitumor efficacy of this combination regimen.

(2) Publish research findings in a peer-reviewed journal with an impact factor >5.

(3) Secure provincial/ministerial-level research funding (1 grant application).

(4) Train 2-3 graduate students to clinical/translational research competency.

# 3. Study Endpoints

## 3.1 Primary Endpoints

Incidence and frequency of DLT

## 3.2 Secondary Endpoints

(1) Adverse drug reactions: Any noxious, unintended response to the investigational drug(s) (Irinotecan liposome + Epirubicin) at any dose, causally related to treatment (per WHO-UMC causality assessment criteria).

(2) Recurrence-Free Survival (RFS): Time from treatment initiation to histologically confirmed tumor recurrence, radiologic progression or death from any cause.

(3) Disease Control Rate (DCR): Proportion of patients achieving complete response (CR), partial response (PR) and stable disease (SD).

Note: Tumor Response Evaluation (per RECIST 1.1 criteria):

- CR: Disappearance of all target lesions; no new lesions.
- PR: ≥30% decrease in the sum of diameters of target lesions.
- SD: Neither sufficient shrinkage to qualify for PR nor sufficient increase to qualify for progressive disease (PD).
- PD: ≥20% increase in the sum of diameters of target lesions or appearance of new lesions.

# 4. Study design

This is a prospective, single-center, single-arm Phase I clinical study designed to evaluate the safety profile (including dose-limiting toxicities), efficacy, and tolerability of neoadjuvant irinotecan liposome in combination with Epirubicin.

## 4.1 Overall design

Enrolled patients with anthracycline-resistant recurrent NMIBC will first undergo enhanced pelvic-abdominal MRI and cystoscopy to collect imaging and pathological data. Two senior urologists will determine whether the tumor is resectable via TURBT. If unresectable (Arm A), patients will receive neoadjuvant irinotecan liposome combined with Epirubicin per the dose-escalation protocol. Post-treatment efficacy will be assessed by contrast-enhanced pelvic-abdominal MRI. For patients with CR and PR, TURBT with intravesical therapy was performed following confirmation by two independent senior urologists. For patients with SD or PD, subsequent therapies were determined by the investigator’s discretion. If resectable (Arm B), TURBT will be performed followed by immediate intravesical Epirubicin (50 mg) instillation within 24 hours postoperatively. During the induction phase (weeks 1-4 post-TURBT), the treatment with irinotecan liposome combined with Epirubicin will be completed according to the dose - escalation regimen. During the maintenance phase (months 1-6 post-TURBT), intravesical maintenance instillation of Epirubicin will be conducted once a month for a total of 6 cycles. The safety and adverse reactions will be followed up from the induction phase, and the tumor recurrence will be assessed by pelvic and abdominal enhanced MRI and cystoscopy after combination treatment.

## 4.2 Dose Escalation Regimen

The dose-escalation scheme is designed based on a comprehensive evaluation of non-clinical in vitro pharmacodynamic data, existing clinical efficacy evidence and Safety profiles.

Arm A: Participants in this arm will complete the neoadjuvant therapy with irinotecan liposome (intravenous injection, once every two weeks for 1 month, dose increasing regimen: 37.6 mg/m2 and 56.5mg/m2) and epirubicin (intravesical instillation, once a week for 1 month, 50 mg).

Arm B: In the induction phase, the first three participants received a reduced dose of irinotecan liposomes (37.6 mg/m2, once every two weeks) in combination with standard intravesical EPI chemotherapy (50 mg, once a week) during the first cycle. If the combination was well tolerated, the irinotecan liposome dose was escalated to the full dose (56.5 mg/m2, once every two weeks) in the second cycle. The remaining three participants received the full dose of irinotecan liposomes (56.5 mg/m2, once every two weeks) along with standard EPI (50 mg, once a week) in both treatment cycles. Epirubicin was given at a fixed dose of 50 mg q1w for 4 cycles. In the maintenance phase, Epirubicin 50 mg q1m was intravesical instillation for a total of six cycles.

## 4.3 Dose Escalation Principles

(1) Following the "3+3 principle", that is, three subjects were enrolled in each group, and the safety and tolerability of the first cycle were observed after administration. If no DLTS occurred, the trial proceeded to the next dose level according to the established dose-escalation protocol. If one DLT occurred, three more subjects were added at that dose level. When no new DLT occurred, the trial was continued at the next dose level. If ≥ 2 DLTS occurred, the dose level preceding that dose level was defined as MTD.

(2) The subjects with DLT were treated by the investigators according to the clinical diagnosis and treatment standards, and the dose could be delayed for no more than 2 weeks, the original dose could be maintained, or the dose level could be lowered to continue the next cycle. If no DLT occurred in the three patients enrolled in the first dose group during the observation period, the subsequent treatment was escalated to the second dose level.

(3) If more than one patient experienced DLT at the prespecified lowest dose, the lower dose was explored by the investigator.

## 4.4 Treatment Period Follow-up

Arm A: routine blood test, liver and kidney function, coagulation function and adverse reactions were followed up every cycle after the first cycle of Irinotecan liposomal treatment. Pelvic and abdominal enhanced MRI and cystoscopy were performed after the second cycle of irinotecan liposomal treatment. The patients who achieved CR and PR were treated with TURBT combined with intravesical therapy after judged by two senior urologists. Patients with SD or PD received subsequent therapy at the investigator's discretion.

Arm B: Routine blood test, liver and kidney function, coagulation function and adverse reactions were followed up every cycle after the first cycle of Irinotecan liposomal treatment. Pelvic and abdominal enhanced MRI and cystoscopy were performed after combination treatment.

## 4.5 Safety Evaluation

The safety analysis will summarize drug exposure (including duration and dose) and adverse events (AEs) based on the safety analysis set population. The investigator is responsible for monitoring the safety of all subjects enrolled in this study, documenting any AEs, including: Serious adverse events (SAEs), Treatment-emergent adverse events (TEAEs) of Grade ≥3, TEAEs leading to treatment discontinuation or dose interruption/reduction/delay.

# 5. Sample Size

Both arms are expected to enroll 6-9 subjects each, with a total planned enrollment of 12-18 subjects.

# 6. Eligibility Criteria

## 6.1 Inclusion Criteria

Subjects can participate in the study only if all the following criteria are met:

(1) Patients with cTa-cT1N0M0 non-muscle invasive bladder cancer (NMIBC);

(2) Disease recurrence following intravesical anthracycline-based chemotherapy;

(3) Recurrent tumors confirmed as cTa-cT1N0M0 bladder urothelial carcinoma;

(4) No prior systemic chemotherapy exposure;

(5) Measurable lesions per Response Evaluation Criteria in Solid Tumors (RECIST);

(6) Eastern Cooperative Oncology Group (ECOG) performance status 0-1.

## 6.2 Exclusion Criteria

Subjects who meet any of the following criteria are not eligible to enter the study:

(1) Age <18 years;

(2) Subjects with severe cardiac, cerebral, hepatic, or renal dysfunction;

(3) Subjects with severe malnutrition;

(4) Subjects with psychiatric disorders or those lacking insight/unable to provide accurate expression;

(5) Concurrent malignancies in other organs;

(6) Presence of systemic infectious diseases.

## 6.3 Discontinuation Criteria

Discontinuation of study treatment does not equate to study withdrawal. Participants who discontinue study treatment must continue to complete all remaining study visits as required by the protocol. Study treatment must be discontinued if a participant meets any of the following criteria:

(1) The study participant withdraws informed consent and refuses to continue receiving the investigational drug treatment;

(2) The investigator determines that the participant's clinical symptoms have worsened/performance status has deteriorated;

(3) Disease progression is confirmed per RECIST v1.1 criteria;

(4) Despite dose modification, the participant remains unable to tolerate adverse events, including any clinical AEs, laboratory abnormalities, or other medical conditions;

(5) The participant commits a major protocol violation or demonstrates non-compliance with medical instructions;

(6) Occurrence of pregnancy, loss to follow-up, or death of the participant;

(7) Other circumstances where the investigator deems it necessary to discontinue the investigational drug treatment.

## 6.4 Withdrawal Criteria

(1) Withdrawal of informed consent - Participant refuses further study participation and follow-up.

(2) Lost to follow-up

(3) Death of participant

(4) Study termination

(5) Other investigator-determined reasons for withdrawal

## 6.5 Termination Criteria

The study may be prematurely terminated or temporarily suspended based on the following criteria:

(1) Identification of unexpected, significant, or unacceptable risks to study participants;

(2) Discovery of critical protocol flaws during trial execution.

# 7. Recruitment Method

This study is an exploratory clinical trial, and participants should be enrolled consecutively based on their willingness to participate. For potential participants, the investigator should discuss the possibility of enrollment. If the participant agrees, the investigator must verify that they meet all eligibility criteria. Eligible participants must sign the Informed Consent Form (ICF).

# 8. Overview of drugs

**Irinotecan Hydrochloride Liposome Injection:**

Specification: 8ml: 37.66mg (as C₃₃H₃₈N₄O₆)

Manufacturer: Jiangsu Hengrui Pharmaceuticals Co., Ltd.

Dosage Form: Injection

Route of Administration: Intravenous infusion

# 9. Special Adverse Reaction Management

In the event of the following special circumstances, appropriate measures for the investigational drugs shall be implemented.

## 9.1 Infusion-related reactions

This section is based on the infusion reactions and allergic reactions described in CTCAE 5.0, with some differences. Relevant handling measures suggestions have also been formulated for reference. Please refer to the table below for details. If infusion reactions or allergic reactions occur, the time needs to be recorded at least to the minute precision.

Table 1. Treatment measures for infusion reactions and allergic reactions

| **Infusion reactions and allergic reactions** | **Treatment measure** | **Subsequent administration** |
| --- | --- | --- |
| The reaction was transient and mild. No need to interrupt the infusion;  No treatment required | Reduce the infusion rate by 50%;  Monitor the research participants every 15 minutes to prevent the deterioration of their condition; | Reduce the infusion rate |
| The treatment or infusion needs to be interrupted and appropriate symptomatic treatment (such as antihistamines, non-steroidal anti-inflammatory drugs, anesthetics, intravenous administration) should be carried out promptly; preventive medication for no more than 24 hours is required. | Stop the administration;  50mg of diphenhydramine hydrochloride is intravenously infused, 650mg of acetaminophen is taken orally, and oxygen therapy is provided;  After the symptoms are relieved, the administration is resumed at a 50% infusion rate;  Monitor the study participants every 15 minutes to prevent the deterioration of the condition; | Reduce the infusion rate;  Preventively administer 25-50mg of diphenhydramine hydrochloride, intravenously, 10mg of dexamethasone, intravenously, and 650mg of acetaminophen, orally before administration. |
| Symptomatic bronchospasm with or without urticaria; requires parenteral treatment;  allergic-related edema/hemolytic anaphylaxis; hypotension | Stop the medication and remove the infusion tube;  50mg of Diphenhydramine hydrochloride is infused intravenously, 10mg of Dexamethasone is also infused intravenously. If bronchospasm occurs, use bronchodilators. Other drugs or oxygen therapy can be used as needed. | Prohibition of subsequent dosing cycles; |
| Life-threatening; requires urgent treatment | Stop the medication and remove the infusion tube; Administer epinephrine. If bronchospasm occurs, administer bronchodilators or oxygen therapy;  Intravenously infuse 50mg of diphenhydramine hydrochloride and 10mg of dexamethasone; Consider hospital observation. | Prohibition of subsequent dosing cycles; |

## 9.2 Diarrhea

Diarrhea caused by irinotecan liposome injection usually consists of both early-onset and late-onset diarrhea. Cholinergic action can result in early-onset diarrhea (occurring during or shortly after administration of the drug). It may be accompanied by rhinitis, increased salivation, miosis, tearing, diaphoresis, flushing, bradycardia, and hyperperistalsis that can cause abdominal cramps. If cholinergic syndrome occurs, atropine 0.25 to 1mg (total dose ≤1mg/ day) is recommended for treatment. Study participants who had acute cholinergic symptoms during previous treatment could receive atropine prophylactically until the next dose, at the investigator's discretion.

late-onset diarrhea (usually occurring after 24 hours after administration of irinotecan liposome injection, beginning with the first loose stool) may be of long duration and may result in dehydration, electrolyte disturbances, or infection, even fatal.

Once late-onset diarrhea occurs, it is recommended to give imodil treatment in time. Study participants were instructed to have imodium available and to start treatment if they had irregular or loose stools or more frequent bowel movements than previously reported. The imodil regimen was administered with an initial dose of 4mg, followed by 2mg every 2 hours until 12 hours after the study participants had stopped having diarrhea. In the evening, study participants could take Imodium 4mg every 4 hours. Continuous use of the above doses of imodil for more than 48 hours is not recommended because of the risk of paralytic ileus, nor is it recommended for less than 12 hours. Prophylactic administration of imodil is not recommended.

When diarrhea causes electrolyte disturbance and induces QTc interval prolongation, electrolyte supplementation is needed in time. When the underlying abnormality is recovered and the electrocardiogram is abnormal, the treatment can be continued with intensive monitoring, and the dose can be carefully adjusted.

If diarrhoea is present, it needs to be recorded at least to the hour.

# 10. Concomitant Medications

All concomitant medications must be recorded in the Case Report Form (CRF), and the concomitant medications/treatments should be recorded from 28 days before the first dose to 30 days after the last dose. If a new antineoplastic therapy was initiated within 30 days after the last dose of the study drug, only concomitant medications/treatments for adverse events related to the study drug were recorded. After 30 days of the last dose, only concomitant medications/treatments used for follow-up for adverse events were recorded.

## 10.1 Prohibited Medications During the Study

The following systemic anti-tumor therapies are strictly prohibited during the study period, including but not limited to chemotherapy and immunotherapy not specified in the protocol, targeted therapy, modern traditional Chinese medicine preparations approved by the National Medical Products Administration (NMPA) for anti-tumor therapy, and immunomodulatory agents with adjuvant anti-tumor effects (such as thymosin, lentin, interleukin-12, etc.). These drugs may interact with Irinotecan and affect the plasma concentration of irinotecan.

## 10.2 Medications Requiring Cautious Use During the Study

Strong inducers/inhibitors of CYP3A4 and CYP2C8 or strong inhibitors of UGT1A1 should be used with caution during treatment. The above drugs may interact with irinotecan and should be used with caution. When combined with other drugs, it is recommended to choose alternative drugs that have no inhibition or induction of CYP3A4 and CYP2C8 enzymes. If they must be used at the same time with CYP3A4 and CYP2C8 enzyme inducers or inhibitors, it is necessary to consider whether to adjust the dose combined with clinical observation.

## 10.3 Supportive Care

Investigators should provide study participants with optimal supportive care when adverse events occur. The use of palliative and supportive care for disease-related symptoms will depend on the judgment of the investigator and relevant guidelines (e.g., the American Society of Clinical Oncology (ASCO) guidelines).

## 10.4 Nausea and vomiting treatment

Primary prevention of nausea and vomiting was permitted. The choice of prophylactic agent was left to the discretion of the investigator. The use of prophylaxis was approved if it was not included in the chapter on concomitant medications and there were no known or predictable drug interactions during treatment.

# 11. Administration, distribution and recycling of drugs

Personnel were responsible for the administration, distribution, and recycling of the study drugs, and investigators were required to ensure that all the study drugs were used only for the study participants who were enrolled in the study and that the doses and administration were in accordance with the study protocol. The remainder of the study drug was returned to the sponsor. The remaining liquid medicine was directly destroyed according to the standard of medical waste. Study drugs were stored according to the instructions.

Study monitors were responsible for monitoring the supply, use, storage, and management of the surplus of study drugs.

# 12. Study Procedure

Before study initiation, all participants must read and sign the current Ethics Committee approved ICF. All examinations and trial procedures shall be conducted according to the schedule specified in the study flowchart, irrespective of the duration of any treatment discontinuation. However, adjustments within the predefined visit windows are permitted due to holidays, festivals, or other administrative reasons. All examinations are recommended procedures and may be modified based on actual clinical circumstances.

## 12.1 Screening period

First, confirm whether the study participant has signed the ICF. Except for pre-existing tumor imaging examinations and pathological diagnoses conducted within the specified timeframe before initial treatment, written informed consent must be obtained prior to performing any clinical study procedures.

The screening period begins upon signing the ICF and ends either at randomization or upon screening failure. Study participants who withdraw from the study after signing the ICF but before randomization will be considered "screening failures." This study allows previously screened participants who failed screening to re-enter the screening process, provided they re-sign the ICF and are assigned a new participant identification number.

The interval between signing the ICF and the first treatment must not exceed 21 days. Final eligibility for study participation will be assessed within the designated timeframes outlined in the visit schedule.

During the screening period (Days -28 to -1), the following study procedures must be completed to confirm participant eligibility:

- Signing of ICF
- Verification of inclusion/exclusion criteria
- Documentation of demographic data, medical history, and prior medications
- Recording of vital signs, height, and weight
- Physical examination
- ECOG PS assessment
- Complete blood count/blood chemistry (within 7 days prior to first dose)
- Coagulation function tests (within 7 days prior to first dose)
- Myocardial enzymes
- Pregnancy test (within 72 hours prior to first dose)
- Adverse event assessment
- Concomitant medications and treatments
- Tumor imaging evaluations: Pelvic MRI (plain + contrast-enhanced);

## 12.2 Treatment period

- Vital signs: Heart rate, respiratory rate, temperature, and blood pressure;
- Physical examination: Facial features, integumentary system, lymph nodes, eyes, ears, nose, throat, oral, respiratory system, cardiovascular system, abdomen, urinary tract system, musculoskeletal system, nervous system, and spiritual state;
- Blood pressure (BP) monitoring: BP is measured by the subject himself/herself and recorded in the patient diary card. BP is tested at least 3 times per week for the first 2 cycles and followed up each day if the blood pressure was abnormal. In addition, BP is measured again by the investigator at each follow-up visit, coffee and tobacco intake are prohibited within 30 minutes before each BP is measured and the measurement is taken in the sitting position with the arm at heart level after at least 10 minutes of quiet sitting, and each BP measurement is taken on the same side;
- Hemanalysis: Hemoglobin, red blood cell, white blood cells, absolute neutrophil count, lymphocyte count, and platelet count;
- Blood biochemistry: TBil, ALT, AST, ALP, r-GT, total protein, Cr, uric acid (UA), blood glucose, triglyceride, cholesterol, potassium, sodium, chlorine, calcium, urea, and phosphonium;
- Coagulation tests: PT, APTT, TT, Fbg, and INR;
- Myocardial enzymes: Creatine Kinase, Myoglobin, CK-MB, Troponin T;
- ECOG PS;
- Adverse event assessment;
- Documentation of concomitant medications and treatments;
- Tumor imaging evaluation;
- Study drug administration (for visits requiring tumor imaging evaluation, the assessment must be completed prior to drug administration).

## 12.3 End-of-treatment and withdrawal study

To be completed within 3 days after confirmed treatment discontinuation, including the following:

- Vital signs: Heart rate, respiratory rate, temperature, and BP;
- Physical examination: Facial features, integumentary system, lymph nodes, eyes, ears, nose, throat, oral, respiratory system, cardiovascular system, abdomen, urinary tract system, musculoskeletal system, nervous system, and spiritual state;
- ECOG PS;
- Hemanalysis / Blood biochemistry;
- Coagulation tests: PT, APTT, TT, Fbg, and INR;
- Myocardial enzymes;
- Tumor imaging evaluation;
- Adverse event assessment;
- Documentation of concomitant medications and treatments;
- Subsequent anti-tumor therapy.

## 12.4 Safety Follow-up

The follow-up shall be conducted at 7 days (±2 days) after the last dose of study medication and includes the following:

- Adverse event assessment;
- Documentation of concomitant medications and concomitant therapies;
- Recording of subsequent anti-tumor therapies.

Collection of adverse events begins from the signing of the ICF and continues until the end of the safety follow-up period (i.e., 7 days after the last administration of the study drug or upon screening failure).If a study participant initiates new anti-tumor therapy before the end of the safety follow-up period, only AEs/SAEs suspected to be related to the study drug will be collected from the start of the new anti-tumor therapy until the end of the safety follow-up period. Any death occurring during the safety follow-up period must be reported as an SAE, regardless of whether the participant received other treatments.

After the safety follow-up period, only SAEs considered to be related to the investigational drug will be collected. For participants following the last dose of Irinotecan liposome, clinical follow-up or telephone visits will be conducted to collect information on survival status, AEs, concomitant medications, and concomitant therapies. Investigators may schedule additional visits as needed to follow up on the resolution of AEs.

## 12.5 Survival Follow-up

RFS follow-up was conducted every 2 months from the last dose of medication to collect RFS information and post-study treatment information (including subsequent antineoplastic therapy and efficacy) until death or loss of follow-up or discontinuation of the study.

## 12.6 Unscheduled follow-up

Unscheduled follow-up may be conducted at the request of either the study participant or the investigator. Based on the participant's condition, the investigator will perform relevant examinations including, but not limited to:

- Vital signs;
- Targeted physical examinations;
- ECOG PS assessment;
- Hemanalysis / Blood biochemistry;
- Myocardial enzymes;
- Tumor imaging evaluations.

All examination results from unscheduled visits must be documented in the eCRF.

# 13. Study Assessments

## 13.1 Efficacy assessment

The primary analysis of this study utilizes tumor assessments conducted per RECIST v1.1 criteria. All study-related decisions during trial execution will be based on local investigators' assessments of imaging results, participants' clinical status and relevant laboratory/ diagnostic findings.

## 13.2 Safety Assessment

Investigators or qualified designated personnel shall evaluate each study participant to assess potential new or worsening AEs in accordance with the trial flowchart schedule, with more frequent evaluations permitted when clinically indicated. During the study process and follow-up period, adverse events will be graded and recorded according to CTCAE 5.0. The toxicity characteristics will be determined based on several aspects: severity, causality, toxicity classification, and the measures taken regarding the trial treatment. All adverse events associated with study treatment exposure and with unknown causes should be evaluated to determine whether they may be events with potential immunological causes.

Table 2. Laboratory Safety Evaluation

| Hemanalysis | Neutrophil count (ANC), lymphocyte count (LYM), red blood cell count (RBC), hemoglobin (Hb), platelet count (PLT) |
| --- | --- |
| Blood biochemistry | Total bilirubin (TBIL), direct bilirubin (DBIL), alanine aminotransferase (ALT), aspartate aminotransferase (AST), alkaline phosphatase (AKP), total protein (TP), albumin (ALB), urea/urea nitrogen (BUN), creatinine (Cr), endogenous creatinine clearance rate (Ccr), fasting blood glucose (GLU), amylase (When blood amylase is abnormal and has clinical significance, blood lipase should also be tested), potassium (K), sodium (Na), chlorine (Cl), calcium (Ca), phosphorus (P) |
| Myocardial enzymes | Creatine Kinase (CK), Myoglobin, Creatine Kinase-MB (CK-MB), Troponin T(cTnT); |
| Coagulation function | International Normalized Ratio (INR), Activated Partial Thromboplastin Time (APTT), Prothrombin Time (PT), Fibrinogen (FIB) |

# 14. Reporting of Adverse Events

## 14.1 AEs

### 14.1.1 Definitions of AEs

An AE refers to any unfavorable medical occurrence in a clinical trial participant administered a medicinal product, which does not necessarily have a causal relationship with the treatment. An AE may be any untoward or unintended sign (including an abnormal laboratory finding), symptom, or disease temporally associated with the use of the investigational product, and must include at least the following circumstances:

(1) Pre-existing conditions: A medical condition/disease present prior to trial participation shall only be recorded as an AE if it worsens after initiation of the investigational product (including deterioration of symptoms, signs, or laboratory abnormalities);

(2) New-onset events: Any newly emergent adverse medical condition (including symptoms, signs, or newly diagnosed diseases);

(3) Clinically significant laboratory abnormalities.

Diagnostic or therapeutic procedures (invasive [e.g., surgery] or non-invasive) should not be reported as AEs. However, the underlying medical condition necessitating the procedure that meets AE criteria shall be reported (e.g., acute appendicitis occurring during the AE reporting period should be reported as an AE, while the subsequent appendectomy should be recorded as the treatment for that AE).

Any administration exceeding the protocol-specified dose by >10% constitutes an overdose. All overdose incidents must be documented in the eCRF.

### 14.1.2 AEs grade

According to the grading criteria for adverse drug events in the CTCAE 5.0 version, if an adverse event not listed in the CTCAE 5.0 table occurs, the following standards can be referred to:

Grade 1: Mild symptoms; asymptomatic or mild symptoms; clinical or diagnostic observations only; intervention not indicated;

Grade 2: Moderate symptoms; minimal, local, or noninvasive intervention indicated; limiting age-appropriate instrumental activities of daily living;

Grade 3: Severe or medically significant but not immediately life-threatening symptoms; hospitalization or prolongation of hospitalization indicated; disabling; limiting self-care ADL;

Grade 4: Life-threatening consequences; urgent intervention required;

Grade 5: Death related to AE.

Note: Distinguish between the severity and seriousness of adverse events. For example, "severe headache" may be intense in severity (e.g., Grade 3), but it does not qualify as a SAE unless it meets SAE criteria (e.g., results in hospitalization, persistent disability, or life-threatening consequences).

### 14.1.3 Assessment of AE Relationship to Investigational Product

The investigator shall determine whether there is a reasonable possibility that the investigational product caused or contributed to an AE through comprehensive evaluation. The assessment factors include:

- Temporal relationship between AE onset and investigational product administration;
- Known characteristics of the investigational product;
- Toxicological and pharmacological effects of the investigational product;
- Use of concomitant medications;
- Participant's underlying medical conditions;
- Medical history and family history;
- Dechallenge and rechallenge responses.

The causality assessment between the AE and investigational product shall be classified using the following five-category scale: Definitely related, probably related, possibly unrelated, definitely unrelated and unassessable.

## 14.2 SAE

### 14.2.1 Definitions of SAE

An SAE refers to any adverse event occurring during clinical research that meets one or more of the following criteria:

- Results in death;
- Is life-threatening (The term "life-threatening" refers to an event/reaction that places the participant at immediate risk of death at the time of occurrence (not a hypothetical risk if the condition worsened));
- Requires hospitalization or prolongs existing hospitalization;
- Causes persistent or significant disability/incapacity (Substantial disruption of the ability to perform normal life functions);
- Congenital anomaly or birth defect;
- Other medically significant events (referring to these events which, although not immediately life-threatening or causing death or hospitalization, based on reasonable medical and scientific judgment, may pose risks to the research participants, or may require intervention [such as medication or surgery] to prevent the serious consequences listed in the above definition).

### 14.2.2 Hospitalization Criteria for SAE Reporting

- In clinical studies, any adverse event leading to hospitalization (even if <24 hours) or prolonged hospitalization shall be considered an SAE. However, the following hospitalization scenarios do not require SAE reporting:
- Non-acute care facilities: Rehabilitation centers, Nursing homes;
- Routine emergency care: Emergency room visits lasting <24 hours without admission;
- Ambulatory procedures: Same-day/short-stay surgeries (outpatient/non-ambulatory settings);
- Non-medical reasons: Social/financial factors (e.g., insurance reimbursement);
- Hospitalizations or prolonged hospital stays unrelated to AE are not SAEs.

## 14.3 Collection/follow-up of AE/ SAEs

### 14.3.1 Collection of AE/SAE

The collection of AE/SAE information shall begin from the signing of the ICF and continue until the end of the safety follow-up period (defined as 30 days after the last administration of the investigational product) or Screening failure, whichever occurs first.

If a study participant initiates new anti-tumor therapy prior to the completion of the safety follow-up period, only AE/SAEs suspected to be related to the investigational product shall be collected from the start of the new anti-tumor therapy until the end of the safety follow-up period.

Any death occurring during the safety follow-up period must be reported as an SAE, irrespective of whether the participant received other treatments.

For all AEs occurring in study participants, investigators must maintain detailed documentation including: AE name, Start and end dates, Severity grade, Relationship to the investigational product, Duration, Actions taken regarding the investigational product, Outcome and Whether the event meets SAE criteria.

### 14.3.2 Follow-up of AE/SAE

Investigators shall inquire about all AE/SAEs occurring since the previous visit during each scheduled study visit and promptly provide follow-up information as required by any data queries received.

All AE/ SAEs should be followed until symptoms resolve, or clinically relevant changes in laboratory values return to baseline and/or ≤ grade 1, or have a reasonable explanation (e.g., loss to follow-up, death), or until the event is conclusively confirmed to be unrelated to the study drug or study procedure at the end of the safety follow-up period.

## 14.4 Abnormal liver function

If AST and/or ALT levels are abnormal and the TBIL level is abnormally increased, and the following conditions are met and there are no other reasons for the abnormality, the SAE reporting procedure should be followed.

| conditions | criteria |
| --- | --- |
| (1) Abnormal ALT or AST | Normal baseline: ALT or AST > 3× upper limit of normal (ULN) during treatment;  Abnormal baseline: ALT or AST > 2× baseline level and value > 3× ULN during treatment; Or > 8× ULN; |
| (2) Abnormal TBIL | Normal baseline: TBIL > 2×ULN during treatment;  Abnormal baseline: TBIL increased > 1×ULN or its value > 3×ULN during treatment; |
| (3) No hemolysis and alkaline phosphatase < 2×ULN (or information not available) | |

Participants who had an abnormal AST and/or ALT level combined with an abnormally elevated total bilirubin level during the safety follow-up period were advised to return to the study center for evaluation and confirmation as soon as the abnormal result was known, preferably within 48 hours.

## 14.5 SAE Reporting Procedure

Upon occurrence of an SAE, whether for initial reporting or follow-up reporting, the investigator must immediately complete, sign, and date a Serious Adverse Event/Adverse Event Report Form of special Interest and report it to the appropriate authorities

All SAEs should be recorded in detail, including symptoms, severity (according to CTCAE 5.0), correlation with each study drug, occurrence time, treatment time, measures taken for each study drug due to SAEs, follow-up time and methods, and outcome. If an SAE is considered by the investigator to be unrelated to the study drug but potentially related to study conditions (e.g., discontinuation of the original treatment, or coexisting conditions during the course of the trial), this relationship should be detailed in the narrative section of the SAE report form. If the intensity of an ongoing SAE or its relationship to the study drug changes, a follow-up report should be submitted immediately. If investigators believed that the previously reported SAEs were misreported, they could provide corrections, retractions, or de-escalation instructions in the follow-up report and report them according to SAE reporting procedures.

## 14.6 Disease Progression and Death

Disease progression is defined as the worsening of a participant's condition due to the studied indication, including radiological progression as well as progression of clinical symptoms and signs. The appearance of new metastatic lesions from the primary tumor or progression of existing metastases is considered disease progression.

In the study population of this trial, "disease progression" is an expected outcome and should not be reported as an AE term. When disease progression occurs, the events used to confirm progression should be reported as AEs. For example, if a participant experiences seizures determined to be related to brain metastases, the AE term should be recorded as "seizures" rather than "disease progression" or "brain metastases."

Similarly, events leading to the diagnosis of disease progression—even if they meet criteria such as being life-threatening, requiring hospitalization or prolonging hospitalization, or causing permanent or severe disability/functional impairment (significant limitation in the ability to perform normal life functions)—should not be reported as SAEs. However, if it is uncertain whether the AE is due to disease progression, it should be reported as an SAE.

During the safety follow-up period, deaths assessed by the investigator as potentially caused by symptoms or signs of disease progression must be reported as SAEs.

The term "death" should not be used as an SAE term but rather as an outcome. The adverse event that caused or led to death should be recorded as the SAE term. If the cause of death cannot be determined at the time of reporting, the SAE term should be recorded as "death of unknown cause."

## 14.7 Pregnancy

If the pregnancy is an ectopic pregnancy, spontaneous abortion, intrauterine fetal death, neonatal death, or congenital abnormality, it is considered to be an SAE and needs to be reported according to the reporting procedures of the SAE.

# 15. Data Management and Statistical Analysis

15. 1 Documentation of Source Record**s**

As the original documentation for clinical trials, source medical records and data files shall be completely preserved. The investigator is responsible for completing and maintaining these records. Prior to each entry, the participant information on the medical record cover must be verified. All entries shall be written legibly in clear and readable handwriting.

## 15. 2 CRF Design and Completion

The CRF is designed based on the clinical trial protocol and undergoes joint review by data managers, statisticians, investigators, and medical personnel. The final version of the CRF must be approved by the investigator prior to use. The CRF design must comply with the protocol and adhere to relevant laws and regulations to ensure the collection of all clinical data required by the trial protocol.

The CRF is to be completed by the investigator, and a CRF must be fully filled out for each enrolled participant.

## 15. 3 Monitoring and Auditing of Data

Monitoring Content:

- Compliance with the trial protocol;
- Identification and correction of any data errors or omissions;
- Verification of data authenticity and completeness, a process also referred to as Source Data Verification (SDV).

## 15.4 Recording and Retention of Research Data

To facilitate subsequent evaluation and oversight by relevant authorities, investigators shall retain all study documentation, including verification records for all study participants (enabling effective cross-checking of all records, such as original hospital documentation). The retention period shall be 5 years after study completion or until the investigator determines the materials may be destroyed, whichever occurs later.

## 15.5 Statistical Analysis

### 15.5.1 Analysis datasets

(1) Full Analysis Set (FAS):

The Full Analysis Set (FAS) consists of all study participants who signed the ICF, were enrolled in the study, and received at least one dose of the investigational drug. The FAS serves as the primary analysis set for efficacy endpoints in this study. For cases where the full treatment course was not observed, the last observation carried forward (LOCF) method will be applied to impute missing data for the final analysis.

(2) Per-protocol set (PPS):

PPS will be defined as all subjects who met the trial protocol, did not take prohibited drugs, with favorable compliance, and completed CRF. No imputation of missing data. Efficacy analysis was conducted based on PPS populations.

(3) Safety analysis set (SS):

The SS population will serve as the population for the safety analysis. All randomized subjects have received at least one dose of the research drug and have a post-administration safety record.

### 15.5.2 Analysis Variables

Primary Endpoint: DLT

Secondary Endpoint: Adverse drug reactions, RFS, DCR,

### 15.5.3 Statistical Method

For the primary endpoint, the DLT Analysis Set (DAS) included all participants who were in the DLT evaluation period and either completed the DLT assessment or experienced a DLT during the evaluation period. This analysis set will be used for the analysis and summary of DLT events. DLT Rate = ( Number of participants with ≥1 DLT / Total evaluable participants in DAS)×100%. 95% CI will be computed using the Clopper-Pearson method.

For Secondary Endpoints, such as RFS, the median value and 95% CI are estimated and survival curves are obtained using the Kaplan-Meier method. The log-rank test is used to compare the survival between the two groups. DCR with 95% CI are calculated, and comparisons between treatment groups are performed using a Cochran-Mantel-Haenszel (CMH) test or chi-square test. Two independent samples will be compared for the differences between the two groups in QoL scores by the t-test or Wilcoxon rank sum test.

# 16. Quality Control and Quality Assurance

All investigators must be physicians trained in clinical trials and work under the supervision of senior professionals. Clinical wards must be inspected prior to the trial to ensure compliance with standardized requirements, including the availability of complete emergency equipment. It is recommended that specialized nursing staff administer investigational drugs to participants, with thorough documentation of medication administration to ensure participant compliance. All study sites must strictly adhere to the research protocol and maintain timely and accurate records.

Monitors shall follow standard operating procedures to oversee trial conduct, verifying that all data are recorded and reported accurately and completely. Electronic case report forms must be entered promptly and correctly, with consistency maintained between electronic records and source documentation to ensure protocol compliance.

In the event of a SAE, the study activities may be temporarily suspended if necessary. The trial site shall be subject to audits by the sponsor and drug regulatory authorities. Most critically, investigators and relevant personnel must provide the necessary access and time for monitoring and audit activities.

# 17. Ethical Standards and Informed Consent

This clinical trial shall be conducted in strict compliance with the Declaration of Helsinki (2008 edition) and applicable Chinese clinical trial regulations and guidelines. Prior to trial initiation, the study protocol shall be jointly developed and agreed upon by the investigator and sponsor, duly signed, and submitted to the Institutional Ethics Committee for review and approval before implementation. During trial execution, any proposed protocol amendments shall require resubmission to the Ethics Committee for approval prior to implementation. Should significant new information regarding the investigational product emerge, a revised ICF must be prepared, submitted for Ethics Committee approval, and subsequently re-obtained from study participants.

Prior to trial commencement, the investigator must provide participants with comprehensive details regarding the clinical trial, including but not limited to: the nature of the study, its objectives, potential benefits and risks, as well as participants' rights and obligations. Clinical trial procedures may only commence after participants have fully understood this information, voluntarily expressed their consent, and signed the informed consent document.

# References

[1] Lobo N, Afferi L, Moschini M, Mostafid H, Porten S, Psutka S P et al. Epidemiology, Screening, and Prevention of Bladder Cancer. European urology oncology 5, 628-639 (2022).

[2] Teoh J Y, Kamat A M, Black P C, Grivas P, Shariat S F & Babjuk M. Recurrence mechanisms of non-muscle-invasive bladder cancer - a clinical perspective. Nature reviews. Urology 19, 280-294 (2022).

[3] Laukhtina E, Abufaraj M, Al-Ani A, Ali M R, Mori K, Moschini M et al. Intravesical Therapy in Patients with Intermediate-risk Non-muscle-invasive Bladder Cancer: A Systematic Review and Network Meta-analysis of Disease Recurrence. European urology focus 8, 447-456 (2022).

[4] Chora A F, Pedroso D, Kyriakou E, Pejanovic N, Colaço H, Gozzelino R et al. DNA damage independent inhibition of NF-κB transcription by anthracyclines. eLife 11 (2022).

[5] Mazor T, Pankov A, Song J S & Costello J F. Intratumoral Heterogeneity of the Epigenome. Cancer cell 2016,29, 440-451.

[6] Song H, Liu D, Dong S, Zeng L, Wu Z, Zhao P et al. Epitranscriptomics and epiproteomics in cancer drug resistance: therapeutic implications. Signal transduction and targeted therapy 2020,5, 193.

[7] Topper M J, Vaz M, Marrone K A, Brahmer J R & Baylin S B. The emerging role of epigenetic therapeutics in immuno-oncology. Nature reviews. Clinical oncology 2020,17, 75-90.

[8] Necchi, A. et al. Pembrolizumab as Neoadjuvant Therapy Before Radical Cystectomy in Patients With Muscle-Invasive Urothelial Bladder Carcinoma (PURE-01): An Open-Label, Single-Arm, Phase II Study. J Clin Oncol 36, 3353–3360 (2018).

[9] Powles, T. et al. Clinical efficacy and biomarker analysis of neoadjuvant atezolizumab in operable urothelial carcinoma in the ABACUS trial. Nat Med 25, 1706–1714 (2019).

[10] Van Dijk, N. et al. Preoperative ipilimumab plus nivolumab in locoregionally advanced urothelial cancer: the NABUCCO trial. Nat Med 26, 1839–1844 (2020).

[11] Defachelles A S, Bogart E, Casanova M, Merks J H M, Bisogno G, Calareso G et al. Randomized Phase II Trial of Vincristine-Irinotecan With or Without Temozolomide, in Children and Adults With Relapsed or Refractory Rhabdomyosarcoma: A European Paediatric Soft Tissue Sarcoma Study Group and Innovative Therapies for Children With Cancer Trial. Journal of clinical oncology : official journal of the American Society of Clinical Oncology 2021,39, 2979-2990.

[12] Li Y, Zhao D, Zhang W, Yang M, Wu Z, Shi W et al. A novel camptothecin derivative, ZBH-01, exhibits superior antitumor efficacy than irinotecan by regulating the cell cycle. Journal of translational medicine 2023,21, 422.

[13] Hyung J, Kim I, Kim K P, Ryoo B Y, Jeong J H, Kang M J et al. Treatment With Liposomal Irinotecan Plus Fluorouracil and Leucovorin for Patients With Previously Treated Metastatic Biliary Tract Cancer: The Phase 2b NIFTY Randomized Clinical Trial. JAMA oncology 2023,9, 692-699.

[14] Spigel D R, Dowlati A, Chen Y, Navarro A, Yang J C, Stojanovic G et al. RESILIENT Part 2: A Randomized, Open-Label Phase III Study of Liposomal Irinotecan Versus Topotecan in Adults With Relapsed Small Cell Lung Cancer. Journal of clinical oncology : official journal of the American Society of Clinical Oncology 2024,42, 2317-2326.

[15] Wainberg Z A, Melisi D, Macarulla T, Pazo Cid R, Chandana S R, De La Fouchardière C et al. NALIRIFOX versus nab-paclitaxel and gemcitabine in treatment-naive patients with metastatic pancreatic ductal adenocarcinoma (NAPOLI 3): a randomised, open-label, phase 3 trial. Lancet (London, England) 2023,402, 1272-1281.

[16] Yoo C, Kim K P, Jeong J H, Kim I, Kang M J, Cheon J et al. Liposomal irinotecan plus fluorouracil and leucovorin versus fluorouracil and leucovorin for metastatic biliary tract cancer after progression on gemcitabine plus cisplatin (NIFTY): a multicentre, open-label, randomised, phase 2b study. The Lancet. Oncology 2021,22, 1560-1572.
